# Biomimetic sulfur-catalyzed carbonyl transfer enables the carbonylative difunctionalization of unactivated alkenes

**DOI:** 10.1039/d5sc09889k

**Published:** 2026-01-02

**Authors:** Yuanrui Wang, Xiao-Feng Wu

**Affiliations:** a Dalian National Laboratory for Clean Energy, Dalian Institute of Chemical Physics, Chinese Academy of Sciences Dalian China xwu2020@dicp.ac.cn; b University of Chinese Academy of Sciences Beijing 100049 China; c Leibniz-Institut für Katalyse e.V. Rostock Germany

## Abstract

Novel organic synthesis platforms inspired by enzyme-promoted biochemical transformations often demonstrate unexpected efficacy in solving challenging problems in organic synthesis. Drawing inspiration from the acyl transfer process catalyzed by coenzyme A (CoA) and acetyl–CoA in living organisms, we have developed a biomimetic acyl transfer strategy catalyzed by organic sulfur compounds. By activating the inert C–F bonds of trifluoromethyl aromatics, a multi-component radical relay strategy enables the efficient construction of fluorine-modified γ-aryl carboxylic acid derivatives. Sulfur compounds serve dual roles in the catalytic cycle, acting as electron-donor catalysts to activate inert C–F bonds and forming thioester intermediates to transfer acyl groups to nucleophiles. Building upon this platform, we have for the first time extended the scope of fluoroalkyl carbon radical precursors in cascade carbonylation of alkenes from reactive species to unactivated trifluoromethylarenes.

## Abstract 2

We have developed a biomimetic, sulfur-catalyzed carbonyl transfer strategy for the activation and transformation of aryl trifluoromethyl groups.

## Graphical abstract

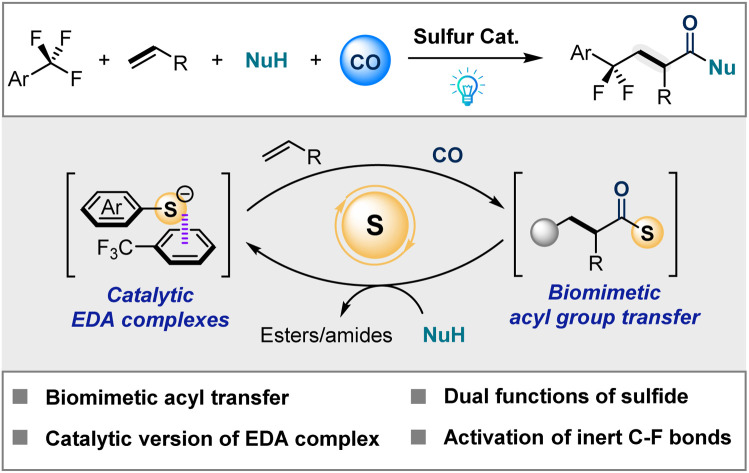

## Introduction

**Fig. 1:**
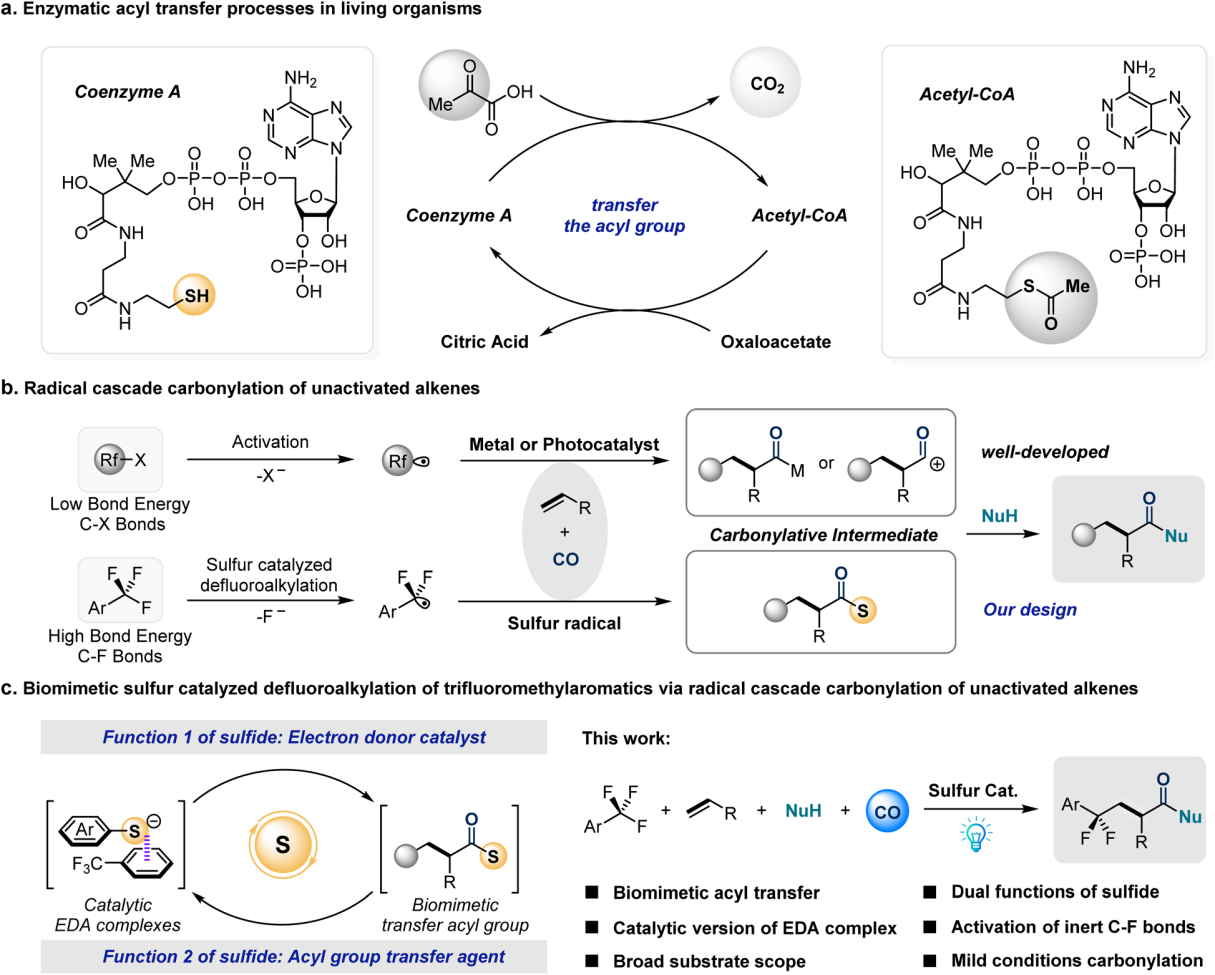
(a) Enzymatic acyl transfer processes in living organisms. (b) Radical cascade carbonylation of unactivated olefins. (c) Biomimetic sulfur catalyzed defluoroalkylation of trifluoromethylaromatics *via* radical cascade carbonylation of unactivated olefins.

The metabolism of sugars and fats in living organisms provides a constant source of energy for cellular activities, and coenzyme A plays an important role in catalyzing this complex biochemical process.^1^ Coenzyme A (CoA) is an intracellular enzyme consisting of three components: mercaptoethylamine, pantothenic acid (vitamin B5), and adenosine 3′-phosphate.^2^ Despite its complex structure, coenzyme A is functionally a simple molecule. The enzymatic reaction of coenzyme A involves only thiol groups. The thiol group of CoA could bind the acetyl group produced by pyruvate decarboxylation, producing acetyl–CoA. Acetyl–CoA then transfers the acetyl group to oxaloacetate,^3^ hydrolyzing the thioester bond to synthesize citrate. CoA is released to complete the catalytic cycle, a process that is irreversible and releases a substantial amount of energy (Fig. 1a).^4^ This sustainable catalytic process has triggered our thinking about catalytic carbonylation coupling reactions in organic synthetic chemistry.

In existing catalytic carbonylative coupling strategies between nucleophiles and CO, the bond formation between nucleophiles and active acyl intermediates primarily occurs through two mechanisms. The first relies on transition-metal catalysis, where nucleophiles generate acyl–metal intermediates *in situ via* ligand exchange, followed by reductive elimination to couple with the acyl group.^5^ The second mechanism involves oxidation of the acyl radical to an acyl cation, which then directly couples with the nucleophile.^6^ This pathway is commonly observed in photocatalytic carbonylation systems.^7^ Inspired by the acyl transfer mechanism of CoA, we aimed to develop a thioester intermediate-mediated acyl transfer strategy for the cross-coupling of nucleophiles with CO (Fig. 1b). To the best of our knowledge, such sulfide-catalyzed carbonyl transfer processes remain underdeveloped in carbonylation. However, thiophenols are often used as hydrogen atom transfer catalysts because the bond dissociation energy of the S–H bond (BDE_S–H_ ∼80–85 kcal mol^−1^) precisely meets the requirements for them to act as catalyst-H species.^8^

Although organic sulfides have shown remarkable performance in catalytic hydrogen atom transfer reactions, their application as electron donors to co-construct electron donor–acceptor (EDA) complexes with electron acceptors has been relatively limited, especially in catalytic EDA systems.^9,10^ In recent years, catalytic EDA systems have achieved exciting advancements in the field of transition-metal-free organic synthesis.^11^ Electron donor catalysts such as amines and phosphines have successfully enabled the efficient activation of radical precursors,^12^ including fluoroalkyl reagents,^13^ aryl sulfonium salts,^14^ sulfonyl chlorides,^15^ and so on. As electron donor catalysts, sulfides also exhibit unique effects in activating the inert C–F bonds of trifluoromethylarenes. Compared with the single-electron reduction catalyzed by metals or photocatalysts, the activation of C–F bonds with higher reduction potentials (trifluoromethyl aromatics without electron-withdrawing groups) *via* sulfide-based electron donor–acceptor (EDA) complexes offers irreplaceable advantages.^16^

The activation of C–F bonds in trifluoromethyl aromatics has received continuous attention and research.^17^ Single-electron transfer (SET) can enable defluorination to generate difluorobenzylic radical species that are difficult to obtain by other methods.^18^ While the use of such radical species in cascade functionalization reactions of activated alkenes has been well-developed,^19^ their application in difunctionalization of unactivated alkenes remains scarcely explored.^20^ Compared with common trifluoromethyl and difluoroacetate carbon radicals, the addition ability of difluorobenzylic radicals to unactivated alkenes is limited. The reason may be that the presence of an aryl group reduces the electrophilicity of the difluoromethyl carbon radical. However, if the carbon radical after addition can be rapidly transformed, it will effectively promote the forward movement of the reaction equilibrium.^21^ We propose that the generation of acyl radicals from alkyl carbon radicals under a CO atmosphere could enable efficient radical–radical coupling with sulfur radicals. This process would effectively promote the addition of difluorobenzylic carbon radicals to unactivated alkenes, while the introduction of carbonyl functional groups would further expand the scope of difunctionalization of unactivated alkenes involving *gem*-difluorobenzylic radicals.^22^

We envisage combining the activation process of trifluoromethylarenes by sulfides with the thioester intermediate-mediated carbonyl transfer process (Fig. 1c). In a catalytic electron donor–acceptor (EDA) system, catalytic amounts of sulfides form complexes with trifluorotoluene. Upon photoexcitation, single-electron transfer (SET) activates the C–F bond to generate sulfur radicals and difluorobenzylic radicals. The latter undergo cascade carbonylation reactions with alkenes to capture CO, forming acyl radicals that couple with sulfur radicals *in situ* to generate reactive thioester intermediates. Subsequently, the thioester will mimic the biochemical process of acetyl–CoA, transferring the acyl group from the thioester to the nucleophile *via* nucleophilic substitution, thereby completing the sulfide-catalyzed carbonylative transformation.

In this study, we developed a biomimetic, sulfur-catalyzed carbonyl transfer strategy. Combined with the activation of aryl trifluoromethyl groups by catalytic electron donor–acceptor (EDA) complexes, we successfully achieved radical cascade carbonylative difunctionalization of unactivated alkenes. The fluoroalkyl carbon radical precursors in radical cascade carbonylation reactions of alkenes have been expanded for the first time from reactive species such as Togni's reagent (II), perfluoroalkyl iodides, and bromodifluoroacetates to unactivated trifluoromethylarenes.^23–25^ Various nucleophiles,^26^ including alcohols, amines, and even phenols, can serve as acceptors for acyl group transfer. A series of γ-*gem*-difluoroalkyl carboxylic acid derivatives were synthesized under mild transition-metal-free conditions.

## Results and discussion

Gratifyingly, our mechanistic hypothesis did lead to a successful protocol for the defluorination cascade functionalization of trifluoromethyl aromatics. Table 1 shows the optimal reaction conditions for the carbonylation of benzyl alcohol as a model nucleophile. Under an atmosphere of 30 bar CO and 10 bar ethylene gas, trifluorotoluene 1a reacts with benzyl alcohol in the presence of 30 mol% disulfide to produce γ-*gem*-difluoroalkyl ester 3a with an 84% yield. This process requires irradiation at 390 nm with 30 W LEDs. We hypothesized that a low concentration of sulfur radicals disfavors efficient capture of acyl radicals. Reducing the amount of disulfide to 10 mol% leads to a decrease in the yield of 3a to 30% (entry 2). The absence of bis(4-methoxyphenyl) disulfide S13 completely prevents the formation of the desired target product 3a, and no conversion of 1a is observed, confirming the critical role of sulfides in generating photoactive species (entry 3). After screening a series of electron donor catalysts, we found that S13 exhibited the highest reaction efficiency. Other thiophenol derivatives can also serve as electron donor catalysts. For example, both 4-fluorothiophenol (S2) and 4-methoxythiophenol (S3) can afford the target product with yields exceeding 60%. When diphenyl sulfide S10 was used as the electron donor catalyst, no 3a was detected and there was no conversion of 1a. We speculate that this is because S10 fails to form an EDA complex with trifluorotoluene, thus making it difficult to activate the C–F bond to trigger the reaction. The reaction can only proceed in polar solvents and is sensitive to bases. Control experiments indicate that the organic strong base DBU is indispensable for this transformation. Reducing the gas pressure will slow down the reaction rate and decrease the conversion of starting materials. For detailed information, please refer to SI Tables S1–S7.

**Table 1:** Optimization of the reaction conditions^a^^,^^b^

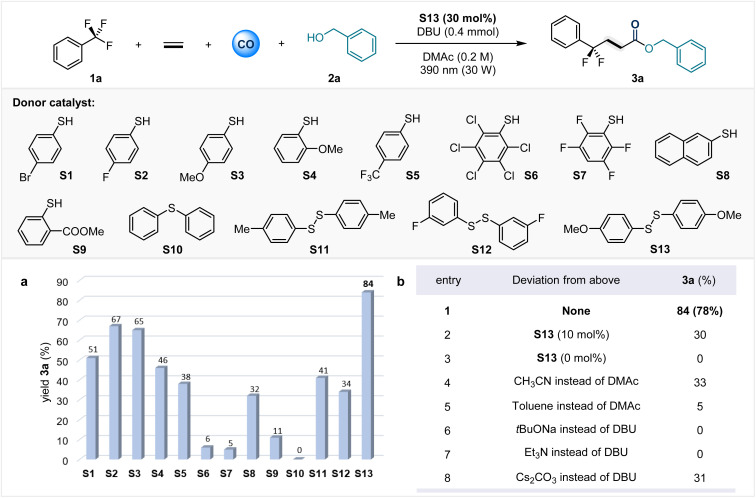

a Reaction conditions: 1a (3.0 equiv), 2a (0.3 mmol), donor catalyst (30 mol%), base (1.3 equiv), solvent (1.5 mL), CO (30 bar), ethylene (10 bar), 30 W 390 nm Kessil lamp, rt, 36 h.

b Effect of other species as donor catalysts on sulfur-catalyzed carbonylation; yields were determined by GC-FID analysis using *n*-hexadecane as an internal standard. Isolated yields are given in brackets.

After optimizing the reaction conditions, we first explored the scope of various *O*-nucleophiles. Overall, a variety of alcohols and phenols containing diverse functional groups could smoothly participate in this multicomponent carbonylation reaction (Table 2). Substituted benzyl alcohols, including –*t*Bu (3b), –COOMe (3c), –Br (3d and 3f), and 2-Me (3e), afforded the target compounds in isolated yields ranging from 51% to 86%. Phenols with electron-donating groups showed excellent adaptability (3g, 3h). Additionally, various alkyl alcohols were investigated, including cyclic secondary alcohols and primary alcohols, which afforded the corresponding carbonylation products in high yields (3i–3l). Notably, we tested some alcohols containing unsaturated bonds as nucleophiles (3m–3p), and the desired transformations were not affected by the presence of reactive double bonds. Even terminal alkynes could survive (3q). Substrates derived from amino alcohols afforded 3r and 3s in high yields of 81% and 82%, respectively. The glycerol derivative also provided 3t in 76% yield. Unfortunately, *tert*-butanol and trifluoroethanol did not yield the corresponding products due to steric and electronic constraints, respectively.

**Table 2:** Scope of nucleophiles^a^

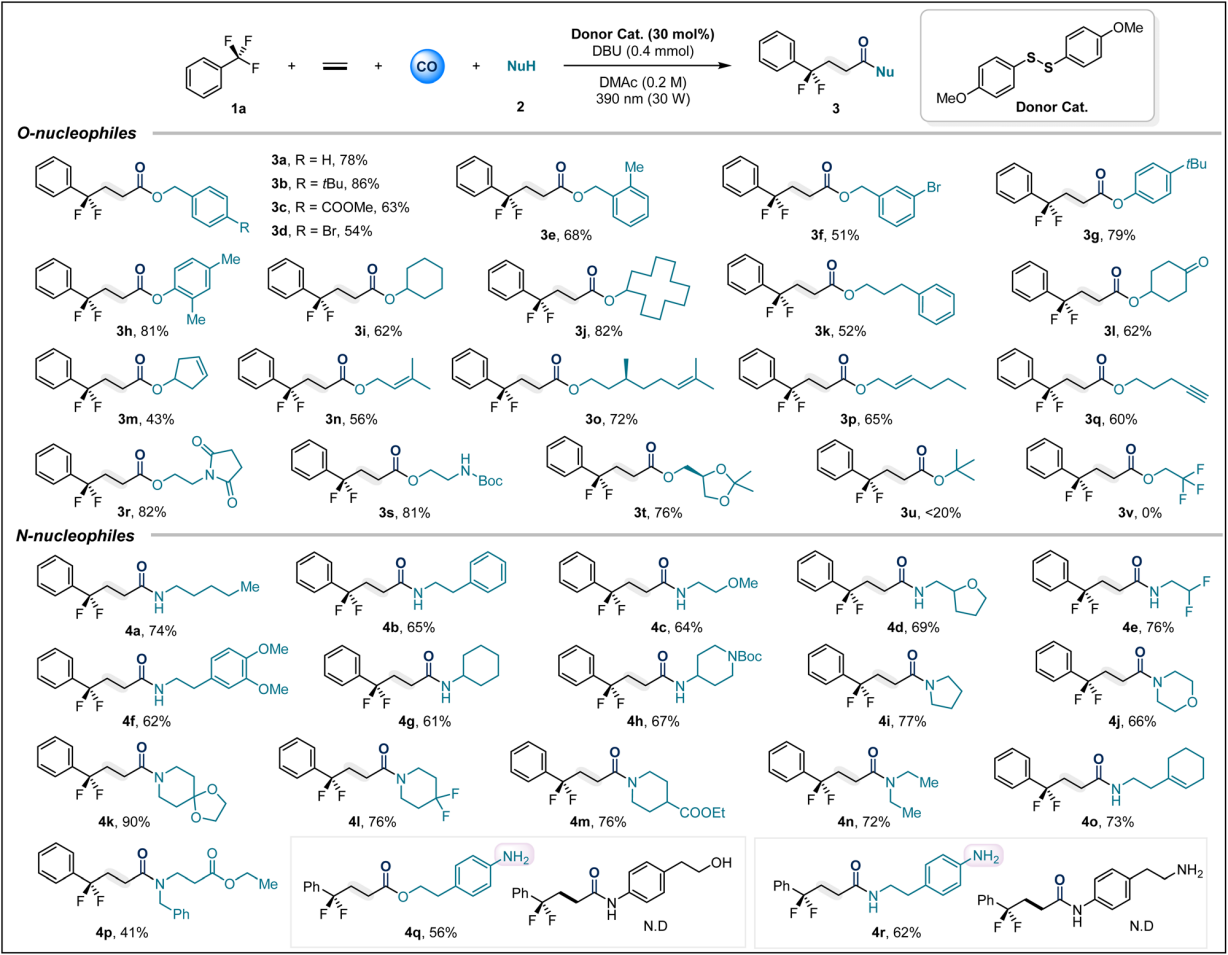

a Reaction conditions: 1a (3.0 equiv), 2 (0.3 mmol), donor catalyst (30 mol%), DBU (0.4 mmol), DMAc (1.5 mL), CO (30 bar), ethylene (10 bar), 30 W 390 nm Kessil lamp, rt, 36 h. All yields are isolated yields.

**Fig. 2:**
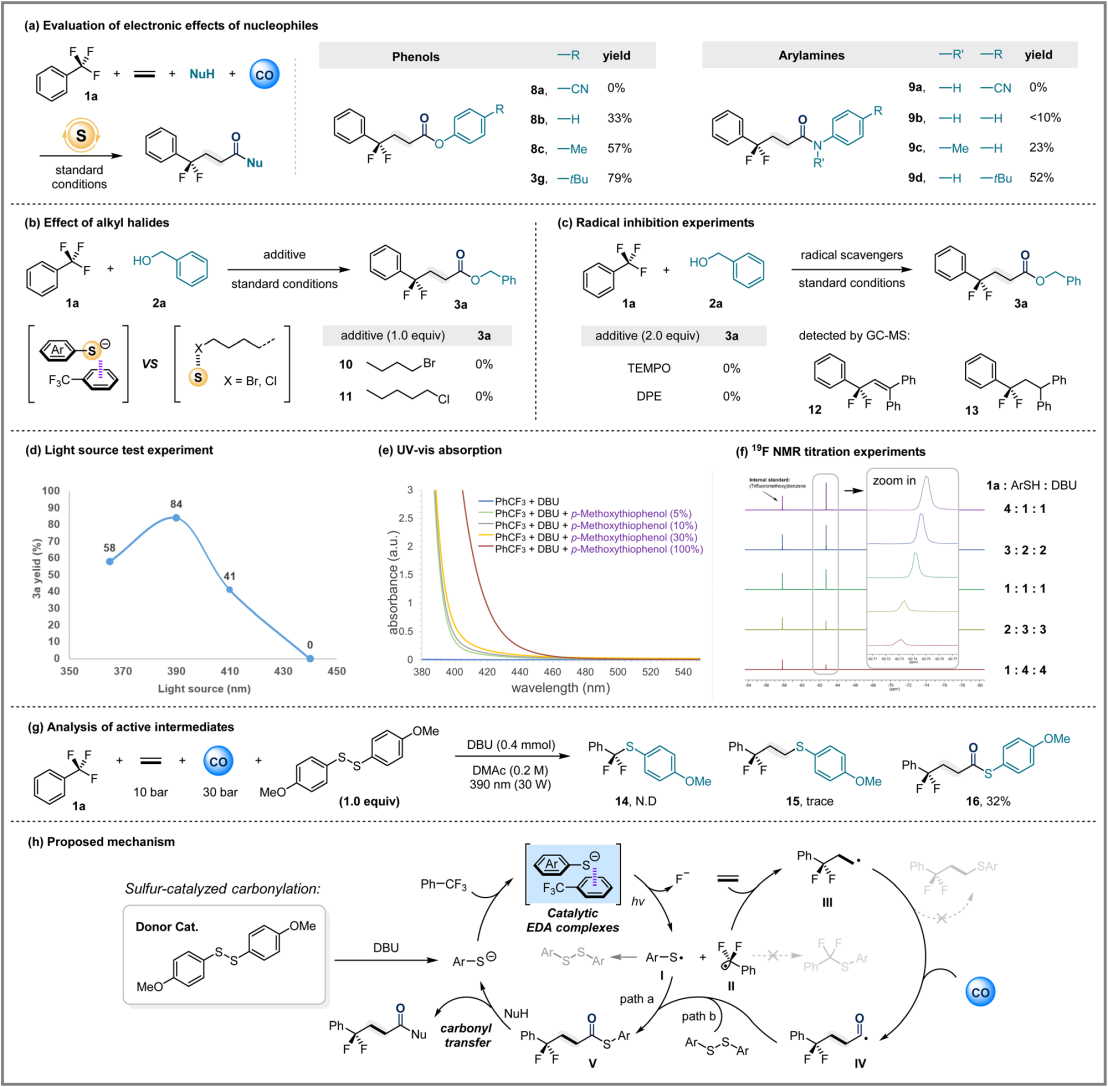
Mechanistic studies of sulfur catalyzed carbonylation. Standard conditions: 1 (3.0 equiv), 2 (0.3 mmol), donor catalyst (30 mol%), DBU (0.4 mmol), DMAc (1.5 mL), CO (30 bar), ethylene (10 bar), 30 W 390 nm Kessil lamp, rt, 36 h. (a) Evaluation of electronic effects of nucleophiles. (b) Effect of alkyl halides. (c) Radical inhibition experiments. (d) Light source test experiment. (e) UV-Vis absorption. (f) ^19^F NMR titration experiments. (g) Analysis of active intermediates. (h) Proposed mechanism.

Next, the applicability of this method for amide synthesis was further explored using various amines (Table 2). We first tested a series of primary alkyl amines, and the corresponding amide products (4a–4h) were isolated in satisfactory yields. Cyclic secondary amines, possibly due to their stronger nucleophilicity, provided products (4i–4m) in excellent yields under optimized conditions. Amino acid derivatives and alkylamines containing unsaturated bonds were also applicable, delivering 4o and 4p in 73% and 41% yields, respectively. Nucleophiles derived from aniline with dual nucleophilic sites were tested, where alkyl hydroxyl and alkyl amino groups preferentially underwent carbonylative coupling over aniline (4q–4r). It is valuable to note that anilines can serve as nucleophiles, provided no more nucleophilic groups are present (Fig. 2a). This precise selectivity for nucleophilic sites highlights the unique properties of the thioester intermediate-mediated acyl group transfer process.

Subsequently, our attention turned to trifluoromethyl aryl compound bearing substituents (Table 3). It is gratifying that in this system, the *ortho*-, *meta*- and *para*-positions of trifluorotoluene can all tolerate the presence of substituents, and the target products 5a–5d are obtained in good yields. Unfortunately, the trifluoromethyl-substituted pyridine compound only gives trace amounts of product 5e. Trifluoromethylarenes substituted with electron-withdrawing groups are also difficult to adapt to this reaction system. These results may be due to the reduced formation of EDA complexes or mismatched redox properties of this type of substrate. It should be noted that compared with trifluorotoluenes substituted with electron-withdrawing groups, those containing electron donating groups generally exhibit higher reduction potentials. But trifluoromethylarenes substituted with electron-withdrawing groups are difficult to adapt to this reaction system. A possible reason for this limitation is mismatched redox properties.

**Table 3:** Scope of trifluoromethyl aryl compounds and unactivated alkenes^a^

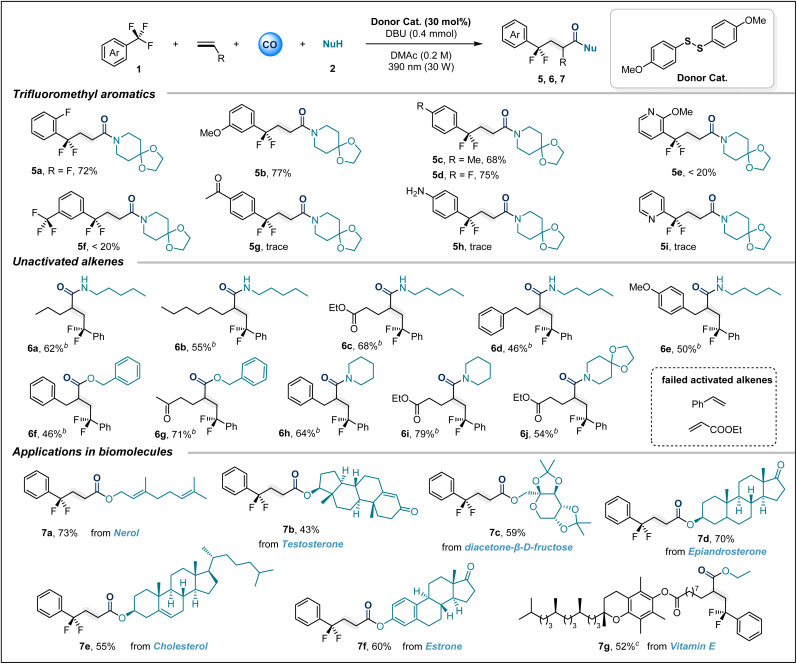

a Reaction conditions: 1 (3.0 equiv), 2 (0.3 mmol), donor catalyst (30 mol%), DBU (0.4 mmol), DMAc (1.5 mL), CO (30 bar), ethylene (10 bar), 30 W 390 nm Kessil lamp, rt, 36 h.

b 1a (3.0 equiv), alkenes (3.0 equiv), 2 (0.3 mmol), donor catalyst (30 mol%), DBU (0.4 mmol), DMAc (1.5 mL), CO (40 bar), 30 W 390 nm Kessil lamp, rt, 36 h.

c 1a (3.0 equiv), alkenes (0.2 mmol), ethanol (5.0 equiv), donor catalyst (30 mol%), DBU (0.4 mmol), DMAc (1.5 mL), CO (40 bar), 30 W 390 nm Kessil lamp, rt, 48 h. All yields are isolated yields.

Additionally, this sulfur-catalyzed four-component carbonylation is not limited to ethylene; various unactivated alkenes also serve as suitable acceptors for radical cascade reactions. The system allows for the smooth transformation of diverse coupling partners, including alcohols and amines, into products 6a–6g under a CO (40 bar) atmosphere. To further verify the practical applicability of this sulfur-catalyzed carbonylation transformation, we attempted to perform carbonylation modification on various bioactive molecules. Satisfactorily, when nerol (7a), testosterone (7b), diacetone-β-d-fructose (7c), epiandrosterone (7d), cholesterol (7e) and estrone (7f) were subjected to the optimized conditions, the corresponding esters were obtained in moderate to good yields. The unactivated olefin derived from vitamin E can also participate in this carbonylative difunctionalization. When ethanol is used as the nucleophile, the expected product 7g is obtained in a moderate yield of 52%.

Although the generality survey of nucleophiles demonstrates the broad compatibility of this system, including amines, alcohols, and phenols, several intriguing results have prompted us to conduct a more in-depth investigation into the detailed mechanism of this transformation. For example, *para*-substituted arylamine and phenol substrates seem to exhibit a strong correlation between yield and nucleophilicity. Therefore, we conducted a systematic investigation on *para*-substituted anilines and phenols. When the *para*-substituents were changed from electron-withdrawing groups to electron-donating groups, the yields of the target products showed a significant upward trend. Upon comparison of the yields between the target products 8a and 3g, or between 9a and 9d, respectively, it is evident that when anilines and phenols are substituted with electron-withdrawing groups, the corresponding amides and esters are difficult to obtain. In contrast, the electron-donating *tert*-butyl group demonstrates good applicability (Fig. 2a). This feature is clearly distinct from carbonylation coupling reactions involving acyl metal species or acyl cation intermediates. The presence of electron-donating groups enhances the nucleophilicity of phenols and anilines, which is crucial for the transfer of the carbonyl group from sulfur atoms to *O*- and *N*-nucleophiles. Based on this, we hypothesize that the reaction intermediate is a thioester, and this reactive species is highly sensitive to the electronic properties of the nucleophile.

Additionally, when using halogen-substituted alkyl alcohols as nucleophiles or halogenated alkyl alkenes as substrates, the expected target products were completely undetectable. Thus, we added alkyl halides to the template reaction under standard conditions to determine whether the presence of halogens would inhibit the target reaction. The results of this functional group sensitivity test confirmed that the addition of alkyl halides indeed completely suppressed the reaction (Fig. 2b). We speculate that the anion–π interaction between sulfides and trifluoromethylarenes is crucial for initiating the reaction. Alkyl chloride/bromide interactions with sulfides are more pronounced than the complexation of trifluorotoluene with sulfides. Therefore, the addition of alkyl halides will completely disrupt the formation of EDA complexes between trifluorotoluene and arylthio anions. Radical inhibition experiments suggested that trifluorotoluene could generate difluorobenzylic radical species in this system (Fig. 2c).

To gain insight into the effect of light irradiation on this transformation, we tested the template reaction under different wavelengths of light.^27^ The results showed that when the wavelength of the light source was changed from 390 nm to 410 nm, the yield of 3a decreased to 41% (Fig. 2d). When irradiated with 440 nm blue light, no 3a was detected at all. The UV-Vis spectra of the reactants and their mixtures showed that the absorption of the thioanion and trifluorotoluene mixture caused a significant redshift compared to the individual reactants (Fig. 2e). This is consistent with the previous light source test results. Additionally, ^19^F NMR titration experiments also provided evidence for the possible interaction between the thioanion and trifluorotoluene (Fig. 2f).

To further confirm the existence of the thioester intermediate, we removed the nucleophile from the template reaction mixture and increased the amount of the electron donor catalyst bis(4-methoxyphenyl) disulfide S13 to one equivalent. After the reaction, rapid column chromatography separation was performed, and thioester 16 was obtained in 32% yield (Fig. 2g). It is worth noting that the actual formation amount of thioester produced may be greater than the isolated yield, as thioester is highly prone to hydrolysis in the presence of organic base DBU, which may lead to low isolated yield. Additionally, only trace amounts of byproducts 14 and 15 (resulting from coupling of alkyl carbon radicals and sulfur radicals) were detected in the reaction system. This indicates that the coupling rate of acyl radicals with sulfur radicals is significantly faster than that of other alkyl radical species, which is the main reason why this reaction system can effectively avoid other coupling pathways. The CO-free control experiment showed that only trace amounts of sulfur coupling product could be detected, and the benzyl alcohol coupling product could not be obtained. This result shows that the difluorobenzylic radicals typically display limited reactivity toward unactivated alkenes, so the presence of CO is essential.

Based on the above results, we propose a reaction mechanism for the catalytic electron donor–acceptor (EDA) system (Fig. 2h). The initial interaction between trifluorotoluene and the arylthio anion in solution forms an EDA complex. Under irradiation with 390 nm LEDs, single-electron transfer occurs within the EDA complex, generating a trifluorotoluene radical anion and a sulfur radical I. Subsequently, the departure of a fluoride anion produces difluorobenzylic radical species II, which could add to alkenes to form alkyl radical intermediate III. Under a CO atmosphere, III captures CO to generate acyl radical species IV. There are two possible pathways for IV to generate the key intermediate V. Path a undergoes efficient and specific cross-coupling with the previously generated sulfur radical I to afford V, and the other possible path b involves radical substitution of the disulfide by the acyl radical. Finally, nucleophilic substitution transfers the acyl group to the nucleophile, resulting in the formation of the target carboxylic acid derivative and regeneration of the electron-donor catalyst thioanion, which then participates in the next catalytic cycle.

## Conclusions

In summary, inspired by the acyl transfer process catalyzed by coenzyme A and combined with the catalytic EDA strategy, we have successfully developed a transition-metal-free radical cascade carbonylation platform under light irradiation. By activating the inert C–F bond of trifluoromethyl arenes, defluorination generates difluorobenzyl radicals to trigger the carbonylative difunctionalization of unactivated alkenes. The thioester intermediate is crucial for the successful transfer of the acyl group to the *O*- and *N*-nucleophile. Relying on this method, various fluorine-modified γ-aryl carboxylic acid derivatives have been efficiently synthesized. This work establishes a precedent for biomimetic catalytic carbonylation by leveraging the inherent metastable properties of thioester intermediates to create a more flexible platform for synthesizing carboxylic acid derivatives through carbonylation.

## Author contributions

Y. W. designed and carried out the reactions and analyzed the data. X.-F. W. designed and supervised the project. X.-F. W. and Y. W. wrote and revised the manuscript.

## Conflicts of interest

There are no conflicts to declare.

## Supplementary Material

SC-OLF-D5SC09889K-s001

## Data Availability

The data supporting this article have been included as part of the supplementary information (SI). Supplementary information: general comments, general procedure, analytic data, and NMR spectra. See DOI: https://doi.org/10.1039/d5sc09889k.
