# Supplementary material for: Biomimetic sulfur-catalyzed carbonyl transfer enables the carbonylative difunctionalization of unactivated alkenes
Source: Chem Sci. 2026 Jan 2;17(7):3683–90. doi: 10.1039/d5sc09889k (PMC12757789; doi:10.1039/d5sc09889k)
Supplement: SC-017-D5SC09889K-s001 [file SC-017-D5SC09889K-s001.pdf]

Supplementary Information (SI) for Chemical Science.
This journal is © The Royal Society of Chemistry 2025

Supporting Information

Table of Contents

1. General information....S2
2. Reaction optimization....S3
3. UV/vis absorption....S7
4. NMR titration experiments....S8
5. Radical-trapping experiment....S9
6. Characterization of Products....S10
7. Copy of NMR Spectrum....S24
8. References....S152

1. General Information

Unless otherwise noted, all reactions were carried out under a carbon monoxide or nitrogen atmosphere. All reagents were from commercial sources, all solvents are extra dry solvents and used as received without further purification. Column chromatography was performed on silica gel (200-300 meshes) using petroleum ether (b.p. 60-90 °C) and ethyl acetate as the eluents. ^{1}H and ^{13}C NMR spectra were taken on Bruker AVANCE III 400 MHz or 700 MHz spectrometers and spectral data were reported in ppm relative to tetramethylsilane (TMS) as the internal standard and $CDCl_3$ or DMSO-D_6 as solvent. All coupling constants (J) are reported in Hz with the following abbreviations: s = singlet, d = doublet, dd = double doublet, t = triplet, dt = double triplet, q = quartet, m = multiplet. Gas chromatography (GC) analyses were performed on an Agilent HP-7890A instrument with a FID detector and HP-5 capillary column (polydimethylsiloxane with 5% phenyl groups, 30 m, 0.32 mm i.d. 0.25 μm film thickness) using argon as carrier gas. Gas chromatography mass spectrometer (GC-MS) analyses were performed on a Shimadzu QP2020 NX instrument. High resolution mass spectra (HRMS) were recorded on Agilent 8890-7250 and Agilent Q-TOF 6540.

Because of the high toxicity of carbon monoxide, all the reactions should be performed in an autoclave. The laboratory should be well-equipped with a CO detector and alarm system.

2. Reaction Optimization

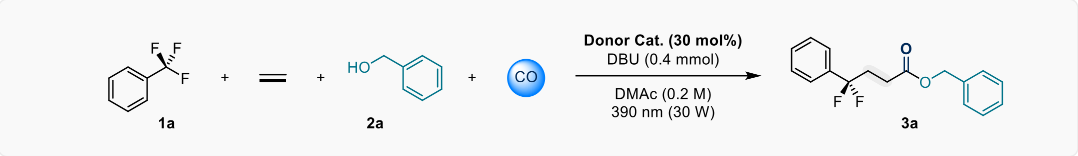

A 4 mL screw-cap vial was charged with **1a** (0.9 mmol), **2a** (0.3 mmol), catalyst (30 mol%), base (0.4 mmol), and solvent (1.5 mL) under N_2 atmosphere. The vial was closed with a Teflon septum and cap and connected to the atmosphere via a needle. The closed autoclave was flushed two times with N_2 (~ 5 bar) and two times with CO (~ 5 bar), and a pressure of 10 bar ethylene and 40 bar CO were charged. The autoclave was then placed on a magnetic stirrer. The reaction mixture was stirred while being irradiated with 30 W 390 nm LEDs at room temperature for 36 h. After irradiation, the pressure was released carefully. Yields were determined by GC-FID analysis using n-hexadecane as internal standard or the mixture was extracted with a saturated NH_4Cl solution three times. The combined organic layer was dried over anhydrous sodium sulfate was concentrated under vacuum. The crude product was purified by column chromatography (PE/EA =50/1 to 2/1) on silica gel to afford the corresponding products. Note: Because of the high toxicity of carbon monoxide, all the reactions should be performed in an autoclave. The laboratory should be well-equipped with a CO detector and alarm system.

Supplementary Table S1. Screening of the amount of donor catalyst

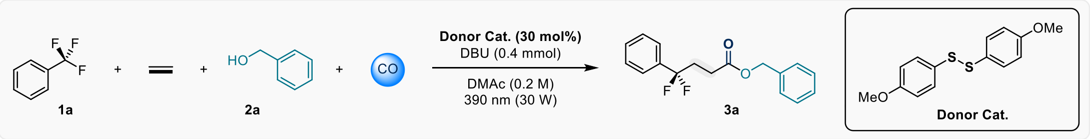

entry	Donor Cat.	base	solvent	**3a** (%)[a]
1	(0 mol%)	DBU	DMAc	0
2	(5 mol%)	DBU	DMAc	21
3	(10 mol%)	DBU	DMAc	30
4	(12.5 mol%)	DBU	DMAc	41
5	(15 mol%)	DBU	DMAc	55
6	(17.5 mol%)	DBU	DMAc	60
7	(20 mol%)	DBU	DMAc	64
8	(22.5 mol%)	DBU	DMAc	71
9	(25 mol%)	DBU	DMAc	74
10	(27.5 mol%)	DBU	DMAc	78
**11**	**(30 mol%)**	**DBU**	**DMAc**	**84**
12	(32.5 mol%)	DBU	DMAc	81
13	(35 mol%)	DBU	DMAc	80

[a] yields were determined by GC-FID analysis using *n*-hexadecane as internal standard. Isolated yields given in brackets.

Supplementary Table S2. Screening of donor catalyst

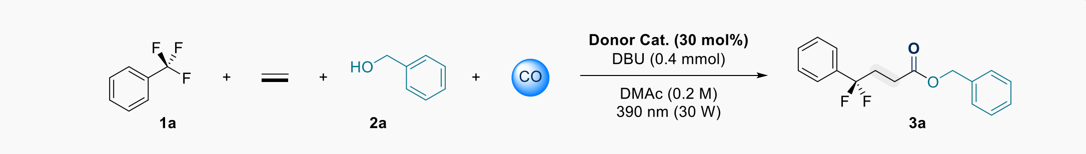

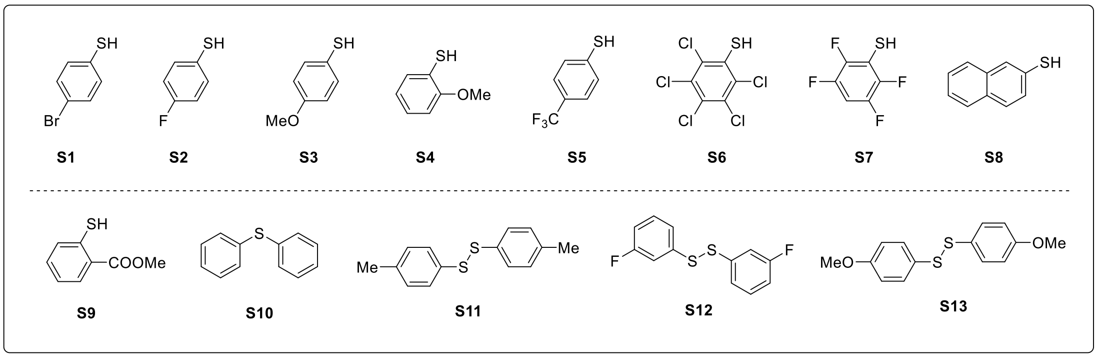

entry	Cat.	base	solvent	**3a** (%)[a]
1	**S1** (30 mol%)	DBU	DMAc	51
2	**S2** (30 mol%)	DBU	DMAc	67
3	**S3** (30 mol%)	DBU	DMAc	65
4	**S4** (30 mol%)	DBU	DMAc	46
5	**S5** (30 mol%)	DBU	DMAc	38
6	**S6** (30 mol%)	DBU	DMAc	6
7	**S7** (30 mol%)	DBU	DMAc	5
8	**S8** (30 mol%)	DBU	DMAc	32
9	**S9** (30 mol%)	DBU	DMAc	11
10	**S10** (30 mol%)	DBU	DMAc	0
11	**S11** (30 mol%)	DBU	DMAc	41
12	**S12** (30 mol%)	DBU	DMAc	34
**13**	**S13 (30 mol%)**	**DBU**	**DMAc**	**84**

[a] yields were determined by GC-FID analysis using *n*-hexadecane as internal standard.

Supplementary Table S3. Screening of solvents

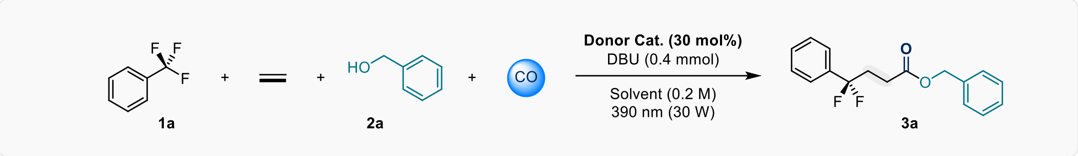

entry	Cat.	base	solvent	**3a** (%)[a]
1	**S13** (30 mol%)	DBU	DMF	60
2	**S13** (30 mol%)	DBU	DMSO	34
3	**S13** (30 mol%)	DBU	CH_3CN	33
4	**S13** (30 mol%)	DBU	THF	11
5	**S13** (30 mol%)	DBU	Toluene	5
6	**S13** (30 mol%)	DBU	DMAc (1 mL)	72
7	**S13** (30 mol%)	DBU	DMAc (1.5 mL)	84
8	**S13** (30 mol%)	DBU	DMAc (2 mL)	80
9	**S13** (30 mol%)	DBU	DMAc (1.5 mL) + H_2O (2 μL)	74

[a] yields were determined by GC-FID analysis using *n*-hexadecane as internal standard.

Supplementary Table S4. Screening of bases

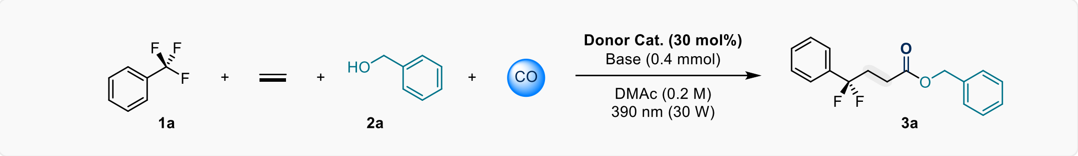

entry	Cat.	base	solvent	**3a** (%)[a]
1	**S13** (30 mol%)	K_2CO_3	DMAc	18
2	**S13** (30 mol%)	Cs_2CO_3	DMAc	31
3	**S13** (30 mol%)	KOMe	DMAc	0
4	**S13** (30 mol%)	*t*BuONa	DMAc	0
5	**S13** (30 mol%)	KOH	DMAc	5
6	**S13** (30 mol%)	KF	DMAc	0
7	**S13** (30 mol%)	Et_3N	DMAc	0
8	**S13** (30 mol%)	DIPEA	DMAc	0
9	**S13** (30 mol%)	Quinuclidine	DMAc	0
10	**S13** (30 mol%)	DBN	DMAc	55

[a] yields were determined by GC-FID analysis using *n*-hexadecane as internal standard.

Supplementary Table S5. Screening of the amount of base

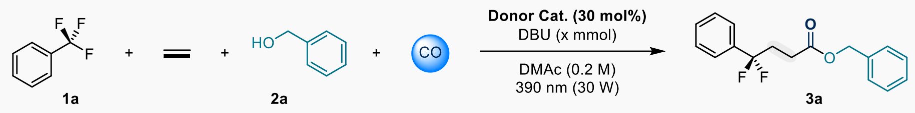

entry	Cat.	base	solvent	**3a** (%)[a]
1	**S13** (30 mol%)	-	DMAc	0
2	**S13** (30 mol%)	DBU (0.3 mmol)	DMAc	48
**3**	**S13 (30 mol%)**	**DBU (0.4 mmol)**	**DMAc**	**84**
4	**S13** (30 mol%)	DBU (0.5 mmol)	DMAc	82
5	**S13** (30 mol%)	DBU (0.6 mmol)	DMAc	61

[a] yields were determined by GC-FID analysis using *n*-hexadecane as internal standard.

Supplementary Table S6. Screening of pressure

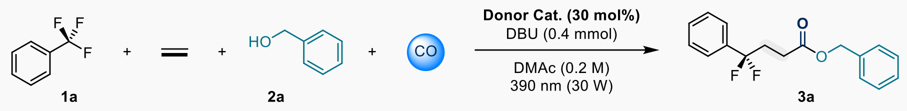

entry	Cat.	base	solvent	pressure of CO/ Ethylene	**3a** (%)[a]
**1**	**S13 (30 mol%)**	**DBU (0.4 mmol)**	**DMAc**	**30 bar + 10 bar**	**84**
2	**S13** (30 mol%)	DBU (0.4 mmol)	DMAc	20 bar + 20 bar	74
3	**S13** (30 mol%)	DBU (0.4 mmol)	DMAc	10 bar + 5 bar	32

[a] yields were determined by GC-FID analysis using *n*-hexadecane as internal standard.

Supplementary Table S7. Screening of light source

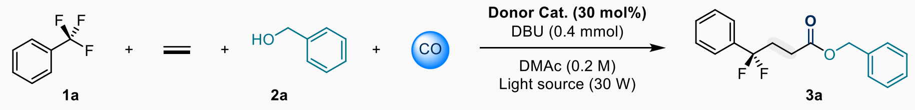

entry	Cat.	base	solvent	Light source	**3a** (%)[a]
1	**S13** (30 mol%)	DBU (0.4 mmol)	DMAc	365 nm	58
**2**	**S13 (30 mol%)**	**DBU (0.4 mmol)**	**DMAc**	**390 nm**	**84**
3	**S13** (30 mol%)	DBU (0.4 mmol)	DMAc	410 nm	41
4	**S13** (30 mol%)	DBU (0.4 mmol)	DMAc	440 nm	0

[a] yields were determined by GC-FID analysis using *n*-hexadecane as internal standard.

3. UV/vis absorption

UV/vis absorption spectra between *p*-methoxythiophenol, DBU and $PhCF_3$ in DMAc were recorded in path quartz cuvettes using a Lambda 950 UV/Vis spectrometer.[1-2] The absorbance of a constant concentration of $PhCF_3$ (0.05M) with DBU (0.05 M), and an increasing concentration of *p*-methoxythiophenol was recorded. The absorption spectra shown in Figures S1.

2.0 mL mixed solution ($PhCF_3$ and DBU mixed in equal proportions (0.05 M)) in cuvettes were added *p*-methoxythiophenol (5%, 10%, 30% and 100%) and to prepare four samples. Three samples were prepared by placing the solution in cuvettes. The absorption spectrum is as follows:

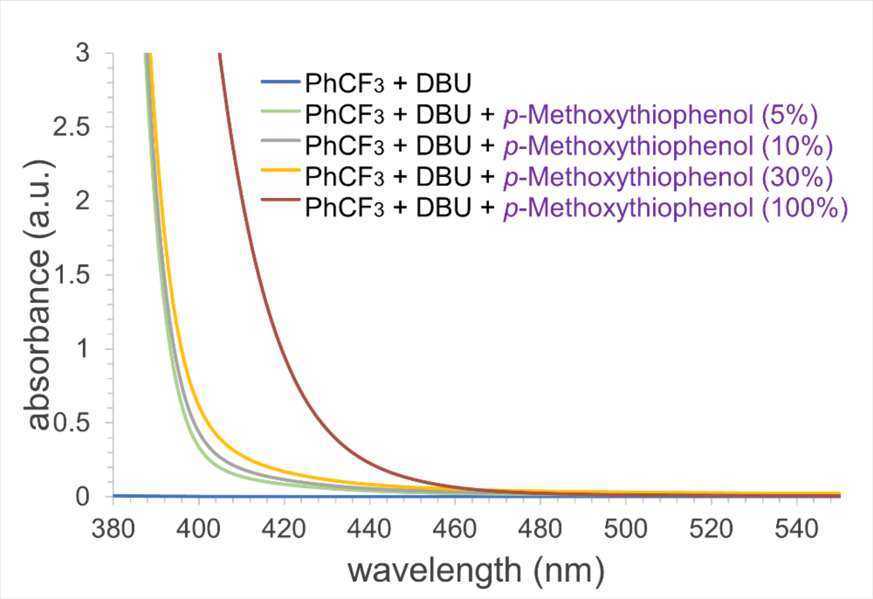

Supplementary Figure S1. UV-vis absorption spectra of $PhCF_3$ (0.05 M in DMAc) and DBU (0.05 M in DMAc) in combination with *p*-methoxythiophenol.

4. NMR titration experiments

^{19}F NMR titration between sulfur anion and $PhCF_3$: Solutions containing equal molar concentrations of the donor (freshly prepared in situ by the deprotonation of *p*-methoxythiophenol with DBU, 0.1 M in $CDCl_3$) and the acceptor ($PhCF_3$, 0.1 M in $CDCl_3$) were prepared and mixed to cover donor/acceptor ratio from 0% to 100% donor.[3-4] (Trifluoromethoxy)benzene was used as an internal standard.

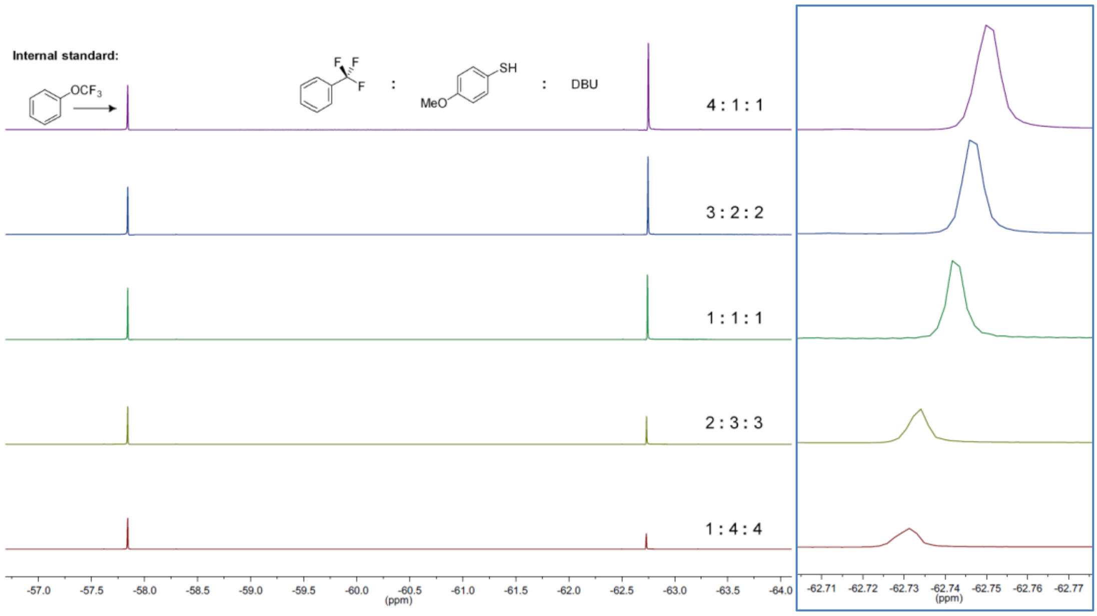

Supplementary Figure S2. The interaction between $PhCF_3$ and sulfur anion.

5. Radical trapping experiments

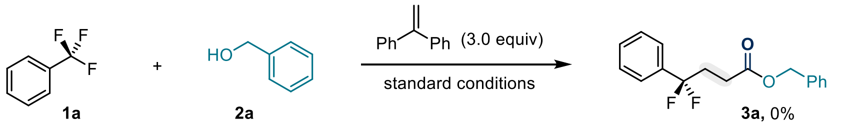

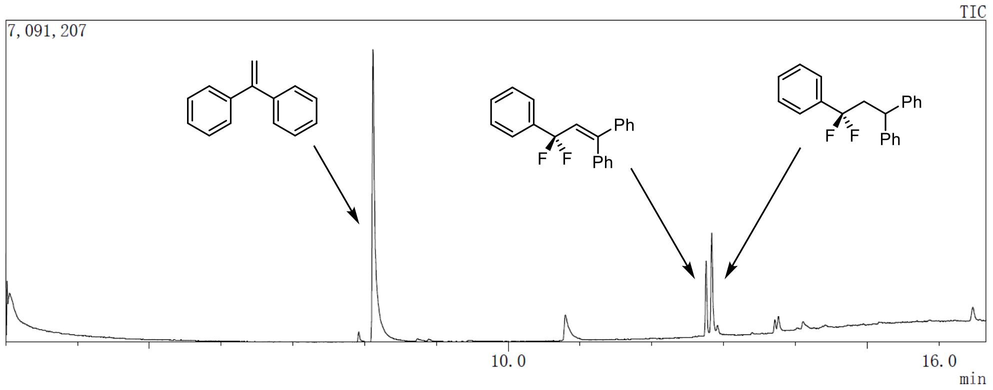

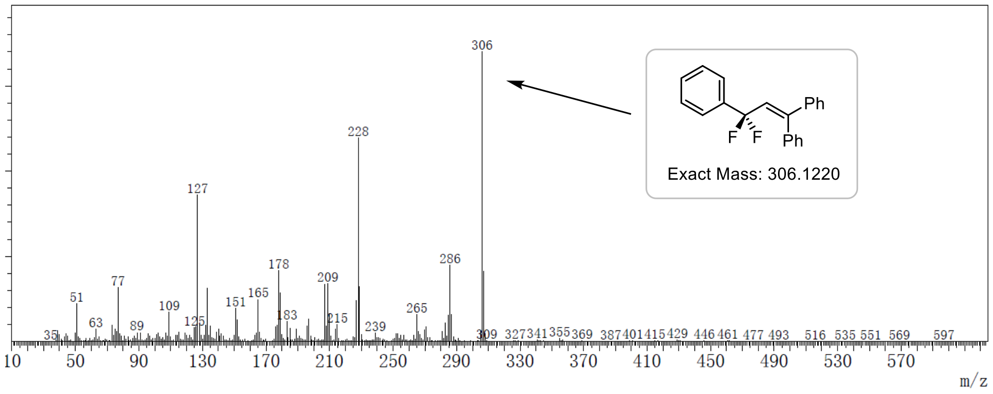

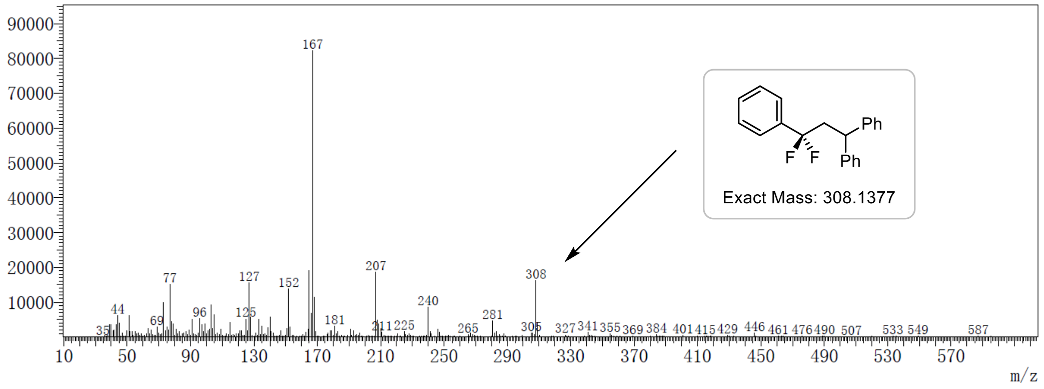

6. Characterization of Products.

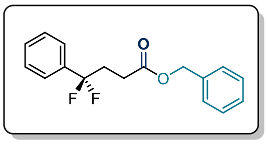

Benzyl 4,4-difluoro-4-phenylbutanoate (3a): 68.2 mg, colorless oil, yield: 78%. Eluent: pentane/ethyl acetate = 50/1; **^{1}H NMR (400 MHz, $CDCl_3$)** δ 7.52 – 7.28 (m, 10H), 5.09 (s, 2H), 2.61 – 2.42 (m, 4H); **^{13}C NMR (101 MHz, $CDCl_3$)** δ 172.0, 136.59 (t, *J* = 26.3 Hz), 135.7, 130.0, 128.6, 128.5, 128.4, 128.3, 124.9 (t, *J* = 6.2 Hz), 122.2 (t, *J* = 242.7 Hz), 66.6, 34.4 (t, *J* = 28.5 Hz), 27.8 (t, *J* = 4.3 Hz); **^{19}F NMR (376 MHz, $CDCl_3$)** δ -96.9; **HRMS (ESI-TOF) m/z:** $[M+H]^+$ Calcd. for $C_{17}H_{17}F_2O_2$ 291.1191; found: 291.1190.

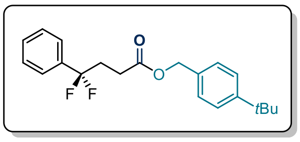

4-(*tert*-Butyl)Benzyl 4,4-difluoro-4-phenylbutanoate (3b): 89.1 mg, colorless oil, yield: 86%. Eluent: pentane/ethyl acetate = 50/1; **^{1}H NMR (400 MHz, $CDCl_3$)** δ 7.49 – 7.33 (m, 7H), 7.30 – 7.23 (m, 2H), 5.06 (s, 2H), 2.58 – 2.43 (m, 4H), 1.31 (s, 9H); **^{13}C NMR (101 MHz, $CDCl_3$)** δ 172.0, 151.5, 136.6 (t, *J* = 26.4 Hz), 132.7, 130.0, 128.6, 128.3, 125.6, 124.9 (t, *J* = 6.2 Hz), 122.2 (t, *J* = 242.6 Hz), 66.5, 34.6, 34.4 (t, *J* = 28.5 Hz), 31.3, 27.8 (t, *J* = 4.3 Hz); **^{19}F NMR (376 MHz, $CDCl_3$)** δ -96.9; **HRMS (ESI-TOF) m/z:** $[M+H]^+$ Calcd. for $C_{21}H_{25}F_2O_2$ 347.1817; found: 347.1812.

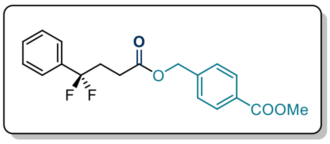

Methyl-4-(((4,4-difluoro-4-phenylbutanoyl)oxy)methyl)benzoate (3c): 65.7 mg, colorless oil, yield: 63% (98% purity). Eluent: pentane/ethyl acetate = 20/1; **^{1}H NMR (400 MHz, $CDCl_3$)** δ 7.47 – 7.38 (m, 5H), 7.27 (d, *J* = 8.6 Hz, 2H), 6.90 – 6.86 (m, 2H), 5.02 (s, 2H), 3.79 (s, 3H), 2.56 – 2.42 (m, 4H); **^{13}C NMR (101 MHz, $CDCl_3$)** δ 172.0, 159.7, 136.6 (t, *J* = 26.4 Hz), 130.2, 130.0, 128.5, 127.8, 124.9 (t, *J* = 6.2 Hz), 122.2 (t, *J* = 242.6 Hz), 114.0, 66.5, 55.3. 34.4 (t, *J* = 28.5 Hz), 27.9 (t, *J* = 4.3 Hz); **^{19}F NMR (376 MHz, $CDCl_3$)** δ -96.9; **HRMS (ESI-TOF) m/z:** $[M+H]^+$ Calcd. for $C_{19}H_{19}F_2O_4$ 349.1246; found: 349.1241.

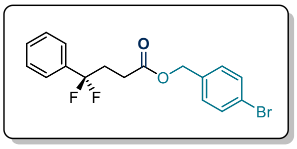

4-Bromobenzyl 4,4-difluoro-4-phenylbutanoate (3d): 59.7 mg, colorless oil, yield: 54%. Eluent: pentane/ethyl acetate = 50/1; **^{1}H NMR (400 MHz, $CDCl_3$)** δ 4.39 – 4.30 (m, 2H), 3.73 (s, 3H), 3.54 (t, *J* = 5.5 Hz, 1H), 3.42 – 3.35 (m, 1H), 3.27 – 3.22 (m, 1H), 2.75 – 2.58 (m, 1H), 2.45 – 2.27 (m, 1H), 1.58 – 1.47 (m, 2H), 1.43 – 1.29 (m, 5H); **^{13}C NMR (101 MHz, $CDCl_3$)** δ 171.8, 136.6 (t, *J* = 26.3 Hz), 134.7, 131.8, 130.0, 128.6, 124.9 (t, *J* = 6.2 Hz) 122.4, 122.1 (t, *J* = 242.6 Hz), 34.3 (t, *J* = 28.5 Hz), 27.7 (t, *J* = 4.2 Hz), 65.8; **^{19}F NMR (376 MHz, $CDCl_3$)** δ -97.0; **HRMS (ESI-TOF) m/z:** $[M+H]^+$ Calcd. for $C_{17}H_{16}BrF_2O_2$ 369.0296; found: 369.0293.

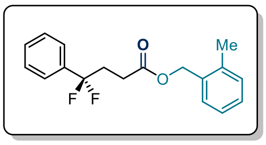

2-Methylbenzyl 4,4-difluoro-4-phenylbutanoate (3e): 62.2 mg, colorless oil, yield: 68%. Eluent: pentane/ethyl acetate = 50/1; **^{1}H NMR (400 MHz, $CDCl_3$)** δ 7.50 – 7.35 (m, 5H), 7.31 – 7.16 (m, 4H), 5.11 (s, 2H), 2.59 – 2.43 (m, 4H), 2.33 (s, 3H); **^{13}C NMR (101 MHz, $CDCl_3$)** δ 172.0, 137.050, 136.6 (t, *J* = 26.3 Hz), 133.6, 130.4, 130.0, 129.4, 128.7, 128.5, 126.1, 124.9 (t, *J* = 6.2 Hz), 122.2 (t, *J* = 242.6 Hz), 65.1, 34.4 (t, *J* = 28.5 Hz), 27.8 (t, *J* = 4.2 Hz), 18.9; **^{19}F NMR (376 MHz, $CDCl_3$)** δ -96.9; **HRMS (ESI-TOF) m/z:** $[M+H]^+$ Calcd. for $C_{18}H_{19}F_2O_2$ 305.1348; found: 305.1342.

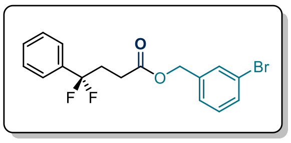

3-Bromobenzyl 4,4-difluoro-4-phenylbutanoate (3f): 56.7 mg, colorless oil, yield: 51% (97% purity). Eluent: pentane/ethyl acetate = 50/1; **^{1}H NMR (400 MHz, $CDCl_3$)** δ 7.53 – 7.34 (m, 7H), 7.27 – 7.20 (m, 2H), 5.05 (s, 2H), 2.62 – 2.43 (m, 4H); **^{13}C NMR (101 MHz, $CDCl_3$)** δ 171.8, 137.9, 136.5 (t, *J* = 26.3 Hz), 131.4, 131.2, 130.2, 130.0, 128.6, 126.7, 124.9 (t, *J* = 6.2 Hz), 122.6, 122.1 (t, *J* = 242.7 Hz), 65.6, 34.3 (t, *J* = 28.6 Hz), 27.7 (t, *J* = 4.2 Hz); **^{19}F NMR (376 MHz, $CDCl_3$)** δ -97.0; **HRMS (ESI-TOF) m/z:** $[M+H]^+$ Calcd. for $C_{17}H_{16}BrF_2O_2$ 369.0296; found: 369.0294.

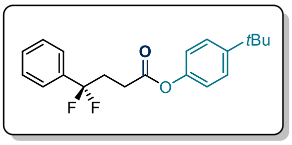

4-(*tert*-Butyl)phenyl 4,4-difluoro-4-phenylbutanoate (3g): 79.2 mg, colorless oil, yield: 79%. Eluent: pentane/ethyl acetate = 50/1; **^{1}H NMR (400 MHz, $CDCl_3$)** δ 7.55 – 7.47 (m, 2H), 7.47 – 7.40 (m, 3H), 7.37 (d, *J* = 8.7 Hz, 2H), 6.96 (d, *J* = 8.7 Hz, 2H), 2.80 – 2.74 (m, 2H), 2.68 – 2.51 (m, 2H), 1.30 (s, 9H); **^{13}C NMR (101 MHz, $CDCl_3$)** δ 170.9, 148.8, 148.2, 136.6 (t, *J* = 26.3 Hz), 130.0, 128.6, 126.4, 125.0 (t, *J* = 6.3 Hz), 122.2 (t, *J* = 242.7 Hz), 120.8, 34.50, 34.46 (t, *J* = 28.6 Hz), 31.4, 28.0 (t, *J* = 4.2 Hz); **^{19}F NMR (376 MHz, $CDCl_3$)** δ -96.9; **HRMS (ESI-TOF) m/z:** $[M+H]^+$ Calcd. for $C_{20}H_{23}F_2O_2$ 333.1661; found: 333.1665.

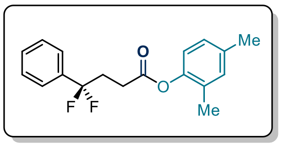

2,4-Dimethylphenyl 4,4-difluoro-4-phenylbutanoate (3h): 73.7 mg, colorless oil, yield: 81%. Eluent: pentane/ethyl acetate = 50/1; **^{1}H NMR (400 MHz, $CDCl_3$)** δ 7.56 – 7.48 (m, 2H), 7.48 – 7.38 (m, 3H), 7.07 – 6.94 (m, 2H), 6.83 (d, *J* = 8.1 Hz, 1H), 2.83 – 2.76 (m, 2H), 2.68 – 2.53 (m, 2H), 2.29 (s, 3H), 2.11 (s, 3H); **^{13}C NMR (101 MHz, $CDCl_3$)** δ 170.7, 147.0, 136.6 (t, *J* = 26.3 Hz), 135.8, 131.8, 130.1, 126.0, 128.6, 127.5, 124.9 (t, *J* = 6.2 Hz), 122.2 (t, *J* = 242.7 Hz), 121.4, 34.5 (t, *J* = 28.5 Hz), 27.7 (t, *J* = 4.2 Hz), 20.8, 16.1; **^{19}F NMR (376 MHz, $CDCl_3$)** δ -97.0; **HRMS (ESI-TOF) m/z:** $[M+H]^+$ Calcd. for $C_{18}H_{19}F_2O_2$ 305.1348; found: 305.1349.

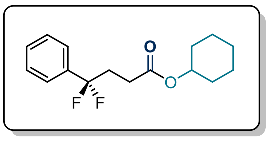

Cyclohexyl 4,4-difluoro-4-phenylbutanoate (3i): 52.5 mg, colorless oil, yield: 62% (96% purity). Eluent: pentane/ethyl acetate = 50/1; **^{1}H NMR (400 MHz, $CDCl_3$)** δ 7.53 – 7.37 (m, 5H), 4.81 – 4.66 (m, 1H), 2.59 – 2.39 (m, 4H), 1.88 – 1.77 (m, 2H), 1.76 – 1.65 (m, 2H), 1.57 – 1.49 (m, 1H), 1.44 – 1.24 (m, 5H); **^{13}C NMR (101 MHz, $CDCl_3$)** δ 171.6, 136.7 (t, *J* = 26.4 Hz), 129.9, 128.5, 124.9 (t, *J* = 6.3 Hz), 122.3 (t, *J* = 242.5 Hz), 73.07, 34.5 (t, *J* = 28.4 Hz), 31.6, 28.2 (t, *J* = 4.2 Hz). 25.4, 23.7; **^{19}F NMR (376 MHz, $CDCl_3$)** δ -96.8; **HRMS (ESI-TOF) m/z:** $[M+H]^+$ Calcd. for $C_{16}H_{21}F_2O_2$ 283.1504; found: 283.1507.

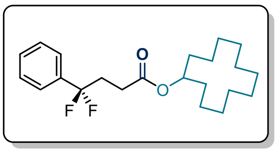

Cyclododecyl 4,4-difluoro-4-phenylbutanoate (3j): 90.7 mg, colorless oil, yield: 82%. Eluent: pentane/ethyl acetate = 50/1; **^{1}H NMR (400 MHz, $CDCl_3$)** δ 7.54 – 7.35 (m, 5H), 5.04 – 4.96 (m, 1H), 2.56 – 2.40 (m, 4H), 1.75 – 1.61 (m, 2H), 1.50 – 1.28 (m, 20H); **^{13}C NMR (101 MHz, $CDCl_3$)** δ 171.8, 136.7 (t, *J* = 26.3 Hz), 129.9, 128.5, 124.9 (t, *J* = 6.2 Hz), 122.3 (t, *J* = 242.5 Hz), 72.7, 34.4 (t, *J* = 28.4 Hz), 29.0, 28.1 (t, *J* = 4.2 Hz), 24.1, 23.8, 23.4, 23.2, 20.9; **^{19}F NMR (376 MHz, $CDCl_3$)** δ -96.8; **HRMS (ESI-TOF) m/z:** $[M+H]^+$ Calcd. for $C_{22}H_{33}F_2O_2$ 367.2443; found: 367.2446.

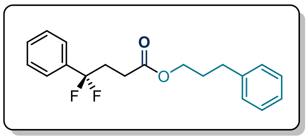

3-Phenylpropyl 4,4-difluoro-4-phenylbutanoate (3k): 50.2 mg, colorless oil, yield: 52%. Eluent: pentane/ethyl acetate = 50/1; **^{1}H NMR (400 MHz, CDCl$_3$)** δ 7.51 – 7.39 (m, 5H), 7.31 – 7.25 (m, 2H), 7.22 – 7.13 (m, 3H), 4.08 (t, *J* = 6.5 Hz, 2H), 2.73 – 2.61 (m, 2H), 2.58 – 2.38 (m, 4H), 2.01 – 1.87 (m, 2H); **^{13}C NMR (101 MHz, CDCl$_3$)** δ 172.2, 141.1, 136.7 (t, *J* = 26.4 Hz), 130.0, 128.5, 128.5, 128.4, 126.1, 124.9 (t, *J* = 6.2 Hz), 122.2 (t, *J* = 242.5 Hz), 64.2, 34.4 (t, *J* = 28.5 Hz), 32.2, 30.1, 27.7 (t, *J* = 4.2 Hz); **^{19}F NMR (376 MHz, CDCl$_3$)** δ -96.9; **HRMS (ESI-TOF) m/z:** $[M+H]^+$ Calcd. for $C_{19}H_{21}F_2O_2$ 319.1504; found: 319.1502.

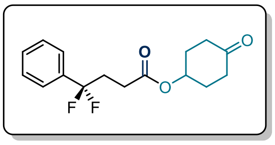

4-Oxocyclohexyl 4,4-difluoro-4-phenylbutanoate (3l): 54.7 mg, colorless oil, yield: 62% (98% purity). Eluent: pentane/ethyl acetate = 10/1; **^{1}H NMR (400 MHz, CDCl$_3$)** δ 7.53 – 7.36 (m, 5H), 5.25 – 5.06 (m, 1H), 2.61 – 2.44 (m, 6H), 2.39 – 2.31 (m, 2H), 2.08 – 2.02 (m, 4H); **^{13}C NMR (101 MHz, CDCl$_3$)** δ 209.6, 171.5, 136.6 (t, *J* = 26.3 Hz), 130.0, 128.6, 124.9 (t, *J* = 6.3 Hz), 122.2 (t, *J* = 242.7 Hz), 69.0, 37.2, 34.4 (t, *J* = 28.5 Hz), 30.3, 28.0 (t, *J* = 4.2 Hz); **^{19}F NMR (376 MHz, CDCl$_3$)** δ -96.9; **HRMS (ESI-TOF) m/z:** $[M+H]^+$ Calcd. for $C_{16}H_{19}F_2O_3$ 297.1297; found: 297.1296.

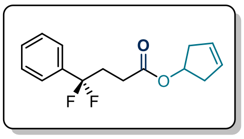

Cyclopent-3-en-1-yl 4,4-difluoro-4-phenylbutanoate (3m): 33.6 mg, colorless oil, yield: 43%. Eluent: pentane/ethyl acetate = 20/1; **^{1}H NMR (400 MHz, CDCl$_3$)** δ 7.51 – 7.36 (m, 5H), 5.70 (s, 2H), 5.38 – 5.30 (m, 1H), 2.71 (dd, *J* = 16.7, 6.9 Hz, 2H), 2.54 – 2.40 (m, 4H), 2.35 (dd, *J* = 16.5, 2.0 Hz, 2H); **^{13}C NMR (101 MHz, CDCl$_3$)** δ 172.0, 136.7 (t, *J* = 26.4 Hz), 129.9, 128.5, 128.2, 124.9 (t, *J* = 6.3 Hz), 122.2 (t, *J* = 242.6 Hz), 74.6, 39.6, 34.4 (t, *J* = 28.5 Hz), 28.0 (t, *J* = 4.2 Hz); **^{19}F NMR (376 MHz, CDCl$_3$)** δ -96.8; **HRMS (ESI-TOF) m/z:** $[M+Na]^+$ Calcd. for $C_{15}H_{17}F_2O_2$ 267.1191; found: 267.1198.

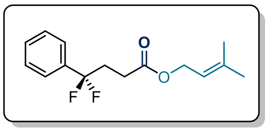

3-Methylbut-2-en-1-yl 4,4-difluoro-4-phenylbutanoate (3n): 33.4 mg, colorless oil, yield: 56%. Eluent: pentane/ethyl acetate = 30/1; **^{1}H NMR (400 MHz, CDCl$_3$)** δ 7.54 – 7.33 (m, 5H), 5.38 – 5.21 (m, 1H), 4.55 (d, *J* = 7.3 Hz, 2H), 2.55 – 2.42 (m, 4H), 1.75 (s, 3H), 1.70 (s, 3H); **^{13}C NMR (101 MHz, CDCl$_3$)** δ 172.1, 139.4, 136.7 (t, J = 26.3 Hz), 129.9, 128.5, 124.9 (t, J = 6.3 Hz), 122.2 (t, J = 242.5 Hz), 118.3, 61.7, 34.4 (t, J = 28.5 Hz), 27.8 (t, J = 4.2 Hz), 25.8, 18.0; **^{19}F NMR (376 MHz, CDCl$_3$)** δ -96.9; **HRMS (ESI-TOF) m/z:** $[M+H]^+$ Calcd. for $C_{15}H_{19}F_2O_2$ 269.1348; found: 269.1342.

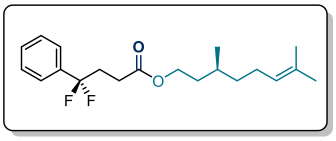

(*S*)-3,7-Dimethyloct-6-en-1-yl 4,4-difluoro-4-phenylbutanoate (3o): 72.7 mg, colorless oil, yield: 72%. Eluent: pentane/ethyl acetate = 50/1; **^{1}H NMR (400 MHz, CDCl$_3$)** δ 7.58 – 7.30 (m, 5H), 5.14 – 5.00 (m, 1H), 4.14 – 4.03 (m, 2H), 2.56 – 2.40 (m, 4H), 2.07 – 1.86 (m, 2H), 1.71 – 1.62 (m, 4H), 1.60 (s, 3H), 1.58 – 1.48 (m, 1H), 1.47 – 1.28 (m, 2H), 1.23 – 1.12 (m, 1H), 0.90 (d, *J* = 6.6 Hz, 3H); **^{13}C NMR (101 MHz, CDCl$_3$)** δ 172.2, 136.7 (t, J = 26.3 Hz), 131.4, 129.9, 128.5, 124.9 (t, J = 6.2 Hz), 124.5, 122.2 (t, J = 242.6 Hz), 63.4, 37.0, 35.4, 34.4 (t, J = 28.5 Hz), 29.4, 27.8 (t, J = 4.2 Hz), 25.7, 25.4, 19.4, 17.6; **^{19}F NMR (376 MHz, CDCl$_3$)** δ -96.9; **HRMS (ESI-TOF) m/z:** $[M+H]^+$ Calcd. for $C_{20}H_{29}F_2O_2$ 339.2130; found: 339.2135.

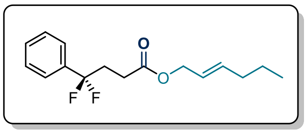

(*E*)-Hex-2-en-1-yl 4,4-difluoro-4-phenylbutanoate (3p): 53.8 mg, colorless oil, yield: 65%. Eluent: pentane/ethyl acetate = 50/1; **^{1}H NMR (400 MHz, CDCl$_3$)** δ 7.53 – 7.34 (m, 5H), 5.81 – 5.68 (m, 1H), 5.60 – 5.43 (m, 1H), 4.49 (d, *J* = 6.5 Hz, 2H), 2.57 – 2.40 (m, 4H), 2.03 (q, *J* = 7.1 Hz, 2H), 1.46 – 1.35 (m, 2H), 0.90 (t, *J* = 7.4 Hz, 3H); **^{13}C NMR (101 MHz, CDCl$_3$)** δ 171.9, 136.7, 136.7 (t, J = 26.4 Hz), 129.9, 128.5, 124.9 (t, J = 6.2 Hz), 123.7, 122.2 (t, J = 242.5 Hz), 65.6, 34.4 (t, J = 28.5 Hz), 34.3, 27.8 (t, J = 4.3 Hz), 22.0, 13.6; **^{19}F NMR (376 MHz, CDCl$_3$)** δ -96.9; **HRMS (ESI-TOF) m/z:** $[M+H]^+$ Calcd. for $C_{16}H_{21}F_2O_2$ 283.1504; found: 283.1509.

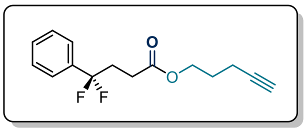

Pent-4-yn-1-yl 4,4-difluoro-4-phenylbutanoate (3q): 46.9 mg, colorless oil, yield: 60%. Eluent: pentane/ethyl acetate = 50/1; **^{1}H NMR (400 MHz, CDCl$_3$)** δ 7.52 – 7.38 (m, 5H), 4.17 (t, *J* = 6.3 Hz, 2H), 2.57 – 2.40 (m, 4H), 2.33 – 2.23 (m, 2H), 1.96 (t, *J* = 2.6 Hz, 1H), 1.89 – 1.78 (m, 2H); **^{13}C NMR (101 MHz, CDCl$_3$)** δ 172.1, 136.6 (t, J = 26.3 Hz), 130.0, 128.5, 124.9 (t, J = 6.2 Hz), 122.2 (t, J = 242.5 Hz), 82.9, 69.1, 63.3, 34.4 (t, J = 28.5 Hz), 27.7 (t, J = 4.3 Hz), 27.4, 15.2; **^{19}F NMR (376 MHz, CDCl$_3$)** δ -97.0; **HRMS (ESI-TOF) m/z:** $[M+H]^+$ Calcd. for $C_{15}H_{17}F_2O_2$ 267.1191; found: 267.1194.

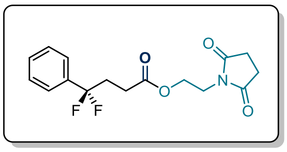

2-(2,5-Dioxopyrrolidin-1-yl)ethyl-4,4-difluoro-4-phenylbutanoate (3r): 78.7 mg, colorless oil, yield: 82% (97% purity). Eluent: pentane/ethyl acetate = 3/1; **^{1}H NMR (400 MHz, CDCl$_3$)** δ 7.51 – 7.37 (m, 5H), 4.25 – 4.21 (m, 2H), 3.77 (t, *J* = 5.2 Hz, 2H), 2.70 (s, 4H), 2.57 – 2.36 (m, 4H); **^{13}C NMR (101 MHz, CDCl$_3$)** δ 177.0, 172.0, 136.6 (t, *J* = 26.3 Hz), 130.0, 128.5, 128.4, 124.9 (t, *J* = 6.2 Hz), 122.2 (t, *J* = 242.5 Hz), 61.2, 37.8, 34.1 (t, *J* = 28.4 Hz), 28.1, 27.6 (t, *J* = 4.2 Hz); **^{19}F NMR (376 MHz, CDCl$_3$)** δ -96.9; **HRMS (ESI-TOF) m/z:** $[M+H]^+$ Calcd. for $C_{16}H_{18}F_2O_4$ 326.1198; found: 326.1193.

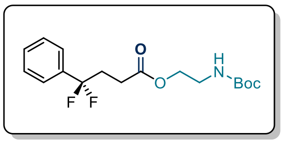

2-((*tert*-Butoxycarbonyl)amino)ethyl-4,4-difluoro-4-phenylbutanoate (3s): 82.8 mg, colorless oil, yield: 81% (96% purity). Eluent: pentane/ethyl acetate = 5/1; **^{1}H NMR (400 MHz, CDCl$_3$)** δ 7.56 – 7.34 (m, 5H), 4.77 (s, 1H), 4.11 (t, *J* = 5.2 Hz, 2H), 3.37 (d, *J* = 5.0 Hz, 2H), 2.62 – 2.38 (m, 4H), 1.44 (s, 9H); **^{13}C NMR (101 MHz, CDCl$_3$)** δ 172.0, 155.8, 136.6 (t, *J* = 26.3 Hz), 130.0, 128.6, 124.9 (t, *J* = 6.2 Hz), 122.2 (t, *J* = 242.6 Hz), 79.6, 64.0, 39.6, 34.3 (t, *J* = 28.5 Hz), 28.4, 27.6 (t, *J* = 4.2 Hz); **^{19}F NMR (376 MHz, CDCl$_3$)** δ -96.9; **HRMS (ESI-TOF) m/z:** $[M+H]^+$ Calcd. for $C_{17}H_{28}F_2NO_4$ 344.1668; found: 344.1663.

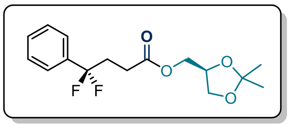

(*S*)-(2,2-Dimethyl-1,3-dioxolan-4-yl)methyl-4,4-difluoro-4-phenylbutanoate (3t): 71.7 mg, colorless oil, yield: 76% (97% purity). Eluent: pentane/ethyl acetate = 5/1; **^{1}H NMR (400 MHz, CDCl$_3$)** δ 7.54 – 7.34 (m, 5H), 4.32 – 4.25 (m, 1H), 4.14 (dd, *J* = 11.5, 4.6 Hz, 1H), 4.11 – 4.03 (m, 2H), 3.72 (dd, *J* = 8.5, 6.1 Hz, 1H), 2.61 – 2.41 (m, 4H), 1.42 (s, 3H), 1.36 (s, 3H); **^{13}C NMR (101 MHz, CDCl$_3$)** δ 171.91, 136.55 (t, *J* = 26.3 Hz), 129.97, 128.54, 124.90 (t, *J* = 6.2 Hz), 122.14 (t, *J* = 242.6 Hz), 109.89, 73.48, 66.27, 65.09, 34.32 (t, *J* = 28.6 Hz), 27.59 (t, *J* = 4.3 Hz), 26.69, 25.35; **^{19}F NMR (376

MHz, $CDCl_3$) δ -96.9; **HRMS (ESI-TOF) m/z:** $[M+H]^+$ Calcd. for $C_{16}H_{21}F_2O_4$ 315.1402; found: 315.1408.

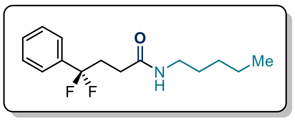

4,4-Difluoro-*N*-pentyl-4-phenylbutanamide (4a): 59.5 mg, colorless oil, yield: 74%. Eluent: pentane/ethyl acetate = 5/1; **^{1}H NMR (400 MHz, $CDCl_3$)** δ 7.52 – 7.38 (m, 5H), 5.67 (s, 1H), 3.20 (dd, *J* = 13.4, 6.8 Hz, 2H), 2.59 – 2.42 (m, 2H), 2.35 (dd, *J* = 9.7, 6.1 Hz, 2H), 1.53 – 1.40 (m, 2H), 1.35 – 1.24 (m, 4H), 0.88 (t, *J* = 6.9 Hz, 3H); **^{13}C NMR (101 MHz, $CDCl_3$)** δ 171.0, 136.8 (t, *J* = 26.4 Hz), 129.9, 128.5, 124.9 (t, *J* = 6.3 Hz), 122.5 (t, *J* = 242.2 Hz), 39.7, 34.8 (t, *J* = 28.0 Hz), 29.6 (t, *J* = 3.6 Hz), 29.2, 29.0, 22.3, 14.0; **^{19}F NMR (376 MHz, $CDCl_3$)** δ -96.6; **HRMS (ESI-TOF) m/z:** $[M+H]^+$ Calcd. for $C_{15}H_{22}F_2NO$ 270.1664; found: 270.1666.

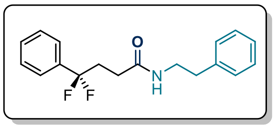

4,4-Difluoro-*N*-phenethyl-4-phenylbutanamide (4b): 59.2 mg, colorless oil, yield: 65%. Eluent: pentane/ethyl acetate = 5/1; **^{1}H NMR (400 MHz, $CDCl_3$)** δ 7.50 – 7.35 (m, 5H), 7.34 – 7.26 (m, 2H), 7.24 – 7.19 (m, 1H), 7.19 – 7.12 (m, 2H), 5.61 (s, 1H), 3.48 (dd, *J* = 13.0, 6.9 Hz, 2H), 2.78 (t, *J* = 7.0 Hz, 2H), 2.56 – 2.40 (m, 2H), 2.30 (dd, *J* = 9.5, 6.2 Hz, 2H); **^{13}C NMR (101 MHz, $CDCl_3$)** δ 171.1, 138.8, 136.8 (t, *J* = 26.4 Hz), 129.9, 128.8, 128.7, 128.5, 126.6, 124.9 (t, *J* = 6.2 Hz), 122.5 (t, *J* = 242.2 Hz), 40.7, 35.6, 34.7 (t, *J* = 28.0 Hz), 29.5 (t, *J* = 3.6 Hz); **^{19}F NMR (376 MHz, $CDCl_3$)** δ -96.6; **HRMS (ESI-TOF) m/z:** $[M+H]^+$ Calcd. for $C_{18}H_{20}F_2NO$ 304.1507; found: 304.1503.

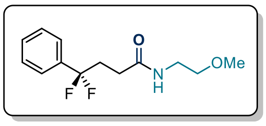

4,4-Difluoro-*N*-(2-methoxyethyl)-4-phenylbutanamide (4c): 49.4 mg, colorless oil, yield: 64%. Eluent: pentane/ethyl acetate = 50/1; **^{1}H NMR (400 MHz, $CDCl_3$)** δ 7.51 – 7.37 (m, 5H), 5.96 (s, 1H), 3.46 – 3.38 (m, 4H), 3.34 (s, 3H), 2.58 – 2.45 (m, 2H), 2.41 – 2.33 (m, 2H); **^{13}C NMR (101 MHz, $CDCl_3$)** δ 171.2, 136.8 (t, *J* = 26.4 Hz), 129.9, 128.5, 124.9 (t, *J* = 6.2 Hz), 122.5 (t, *J* = 242.3 Hz), 71.1, 58.7, 39.3, 34.7 (t, *J* = 28.1 Hz), 29.5 (t, *J* = 3.7 Hz); **^{19}F NMR (376 MHz, $CDCl_3$)** δ -96.6; **HRMS (ESI-TOF) m/z:** $[M+H]^+$ Calcd. for $C_{13}H_{18}F_2NO_2$ 258.1300; found: 258.1304.

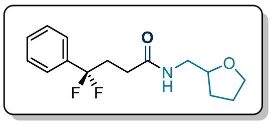

4,4-Difluoro-4-phenyl-*N*-((tetrahydrofuran-2-yl)methyl)butanamide (4d): 58.2 mg, colorless oil, yield: 69%. Eluent: pentane/ethyl acetate = 10/1; **^{1}H NMR (400 MHz, $CDCl_3$)** δ 7.54 – 7.36 (m, 5H), 5.94 (s, 1H), 3.97 – 3.88 (m, 1H), 3.87 – 3.80 (m, 1H), 3.73 (dd, *J* = 15.0, 6.9 Hz, 1H), 3.59 – 3.52 (m, 1H), 3.14 – 3.04 (m, 1H), 2.58 – 2.44 (m, 2H), 2.37 (dd, *J* = 9.6, 6.0 Hz, 2H), 2.01 – 1.83 (m, 3H), 1.56 – 1.46 (m, 1H); **^{13}C NMR (101 MHz, $CDCl_3$)** δ 171.1, 136.8 (t, *J* = 26.3 Hz),129.9, 128.5, 124.9 (t, *J* = 6.2 Hz), 122.5, (t, *J* = 241.4 Hz), 76.8, 68.0, 43.3, 34.7 (t, *J* = 28.1 Hz), 29.5 (t, *J* = 3.7 Hz), 28.6, 25.8; **^{19}F NMR (376 MHz, $CDCl_3$)** δ -96.7; **HRMS (ESI-TOF) m/z:** $[M+H]^+$ Calcd. for $C_{15}H_{20}F_2NO_2$ 284.1457; found: 284.1454.

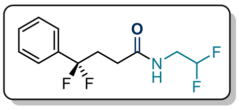

***N*-(2,2-Difluoroethyl)-4,4-difluoro-4-phenylbutanamide (4e):** 59.8 mg, colorless oil, yield: 76%. Eluent: pentane/ethyl acetate = 10/1; **^{1}H NMR (400 MHz, $CDCl_3$)** δ 7.52 – 7.35 (m, 5H), 6.08 (s, 1H), 5.79 (tt, *J* = 56.0, 4.0 Hz, 1H), 3.64 – 3.53 (m, 2H), 2.61 – 2.36 (m, 4H); **^{13}C NMR (101 MHz, $CDCl_3$)** δ 171.9, 136.6 (t, *J* = 26.3 Hz), 130.0, 128.6, 124.8 (t, *J* = 6.2 Hz), 122.4 (t, *J* = 241.4 Hz), 113.5 (t, *J* = 241.2 Hz), 41.8 (t, *J* = 26.3 Hz),

34.5 (t, J = 28.2 Hz), 29.2 (t, J = 3.7 Hz). **^{19}F NMR (376 MHz, CDCl$_3$)** δ -96.8 (s, 2F), -123.0 (s, 2F); **HRMS (ESI-TOF) m/z:** [M+H]$^+$ Calcd. for $C_{12}H_{14}F_4NO$ 264.1006; found: 264.1003.

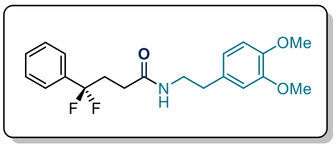

***N*-(3,4-Dimethoxyphenethyl)-4,4-difluoro-4-phenylbutanamide (4f):** 67.4 mg, colorless oil, yield: 62%. Eluent: pentane/ethyl acetate = 5/1; **^{1}H NMR (400 MHz, CDCl$_3$)** δ 7.50 – 7.36 (m, 5H), 6.80 (d, J = 8.0 Hz, 1H), 6.76 – 6.67 (m, 2H), 5.62 (s, 1H), 3.85 (s, 3H), 3.85 (s, 3H), 3.47 (dd, J = 13.0, 6.8 Hz, 2H), 2.73 (t, J = 7.0 Hz, 2H), 2.57 – 2.41 (m, 2H), 2.32 (dd, J = 9.4, 6.2 Hz, 2H); **^{13}C NMR (101 MHz, CDCl$_3$)** δ 171.1, 149.1, 147.7, 136.8 (t, J = 26.4 Hz), 131.3, 129.9, 128.5, 124.9 (t, J = 6.2 Hz), 122.5, (t, J = 241.4 Hz), 120.6, 111.9, 111.4, 55.9, 55.8, 40.8, 35.2, 34.7 (t, J = 28.0 Hz), 29.5 (t, J = 3.6 Hz); **^{19}F NMR (376 MHz, CDCl$_3$)** δ -96.6; **HRMS (ESI-TOF) m/z:** [M+H]$^+$ Calcd. for $C_{20}H_{24}F_2NO_3$ 364.1719; found: 364.1726.

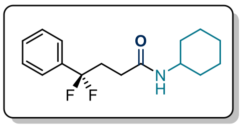

***N*-Cyclohexyl-4,4-difluoro-4-phenylbutanamide (4g):** 51.5 mg, colorless oil, yield: 61%. Eluent: pentane/ethyl acetate = 5/1; **^{1}H NMR (400 MHz, CDCl$_3$)** δ 7.51 – 7.36 (m, 5H), 5.48 (s, 1H), 3.79 – 3.67 (m, 1H), 2.58 – 2.42 (m, 2H), 2.37 – 2.29 (m, 2H), 1.92 – 1.84 (m, 2H), 1.74 – 1.64 (m, 2H), 1.64 – 1.55 (m, 1H), 1.40 – 1.27 (m, 2H), 1.20 – 1.03 (m, 3H); **^{13}C NMR (101 MHz, CDCl$_3$)** δ 170.0, 136.9 (t, J = 26.4 Hz), 129.9, 128.5, 124.9 (t, J = 6.2 Hz), 122.6 (t, J = 242.2 Hz), 48.3, 34.8 (t, J = 28.0 Hz), 33.1, 29.8 (t, J = 3.5 Hz), 25.5, 24.8; **^{19}F NMR (376 MHz, CDCl$_3$)** δ -96.5; **HRMS (ESI-TOF) m/z:** [M+H]$^+$ Calcd. for $C_{16}H_{22}F_2NO$ 282.1664; found: 282.1665.

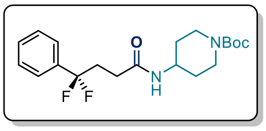

***tert*-Butyl-4-(4,4-difluoro-4-phenylbutanamido)piperidine-1-carboxylate (4h):** 77.3 mg, colorless oil, yield: 67%. Eluent: pentane/ethyl acetate = 2/1; **^{1}H NMR (400 MHz, CDCl$_3$)** δ 7.53 – 7.34 (m, 5H), 5.78 (s, 1H), 4.11 – 3.82 (m, 3H), 2.83 (t, J = 12.0 Hz, 2H), 2.58 – 2.42 (m, 2H), 2.40 – 2.32 (m, 2H), 1.86 (d, J = 12.3 Hz, 2H), 1.45 (s, 9H), 1.32 – 1.22 (m, 2H); **^{13}C NMR (101 MHz, CDCl$_3$)** δ 170.4, 154.7, 136.8 (t, J = 26.4 Hz), 129.9, 128.5, 124.9 (t, J = 6.3 Hz), 122.5 (t, J = 242.2 Hz), 79.7, 46.8, 42.6, 34.7 (t, J = 28.0 Hz), 32.0, 29.6, 28.4; **^{19}F NMR (376 MHz, CDCl$_3$)** δ -96.6; **HRMS (ESI-TOF) m/z:** [M+H]$^+$ Calcd. for $C_{20}H_{29}F_2N_2O_3$ 383.2141; found: 383.2146.

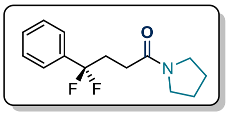

4,4-Difluoro-4-phenyl-1-(pyrrolidin-1-yl)butan-1-one (4i): 58.3 mg, colorless oil, yield: 77%. Eluent: pentane/ethyl acetate = 3/1; **^{1}H NMR (400 MHz, CDCl$_3$)** δ 7.53 – 7.45 (m, 2H), 7.45 – 7.36 (m, 3H), 3.43 (t, J = 6.8 Hz, 2H), 3.40 – 3.35 (m, 2H), 2.63 – 2.41 (m, 4H), 1.98 – 1.88 (m, 2H), 1.88 – 1.78 (m, 2H); **^{13}C NMR (101 MHz, CDCl$_3$)** δ 169.5, 137.0 (t, J = 26.4 Hz), 129.8, 128.4, 124.9 (t, J = 6.2 Hz), 122.8 (t, J = 241.9 Hz), 46.5, 45.8, 34.2 (t, J = 27.6 Hz), 27.7 (t, J = 3.5 Hz), 26.0, 24.4; **^{19}F NMR (376 MHz, CDCl$_3$)** δ -96.5; **HRMS (ESI-TOF) m/z:** [M+H]$^+$ Calcd. for $C_{14}H_{18}F_2NO$ 254.1351; found: 254.1353.

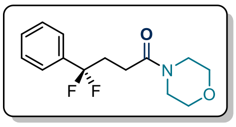

4,4-Difluoro-1-morpholino-4-phenylbutan-1-one (4j): 53.5 mg, colorless oil, yield: 66%. Eluent: pentane/ethyl acetate = 2/1; **^{1}H NMR (400 MHz, CDCl$_3$)** δ 7.53 – 7.46 (m, 2H), 7.46 – 7.39 (m, 3H), 3.69 – 3.63 (m, 4H), 3.62 – 3.54 (m, 2H), 3.47 – 3.41 (m, 2H), 2.61 – 2.45 (m, 4H); **^{13}C NMR (101 MHz, CDCl$_3$)** δ 169.8, 136.9 (t, J = 26.4 Hz), 129.9, 128.5, 124.9 (t, J = 6.2 Hz), 122.6 (t, J = 242.1 Hz), 66.8, 66.5, 45.8,

42.1, 34.4 (t, J = 27.5 Hz), 26.0 (t, J = 3.5 Hz); **^{19}F NMR (376 MHz, CDCl$_3$)** δ -96.6; **HRMS (ESI-TOF) m/z:** [M+H]$^+$ Calcd. for $C_{14}H_{18}F_2NO_2$ 270.1300; found: 270.1303.

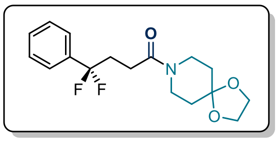

4,4-Difluoro-4-phenyl-1-(1,4-dioxa-8-azaspiro[4.5]decan-8-yl)butan-1-one (4k): 88.2 mg, colorless oil, yield: 90%. Eluent: pentane/ethyl acetate = 3/1; **^{1}H NMR (400 MHz, CDCl$_3$)** δ 7.52 – 7.46 (m, 2H), 7.45 – 7.38 (m, 3H), 3.96 (s, 4H), 3.71 – 3.64 (m, 2H), 3.54 – 3.47 (m, 2H), 2.61 – 2.44 (m, 4H), 1.72 – 1.61 (m, 4H); **^{13}C NMR (101 MHz, CDCl$_3$)** δ 169.3, 137.0 (t, J = 26.4 Hz), 129.9, 128.5, 124.9 (t, J = 6.2 Hz), 122.7 (t, J = 242.1 Hz), 106.8, 64.5, 43.4, 39.9, 35.5, 34.7, 34.6 (t, J = 27.4 Hz), 26.2 (t, J = 3.4 Hz); **^{19}F NMR (376 MHz, CDCl$_3$)** δ -96.6; **HRMS (ESI-TOF) m/z:** [M+H]$^+$ Calcd. for $C_{17}H_{22}F_2NO_3$ 326.1562; found: 326.1566.

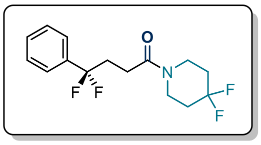

1-(4,4-Difluoropiperidin-1-yl)-4,4-difluoro-4-phenylbutan-1-one (4l): 69.1 mg, colorless oil, yield: 76%. Eluent: pentane/ethyl acetate = 5/1; **^{1}H NMR (400 MHz, CDCl$_3$)** δ 7.54 – 7.46 (m, 2H), 7.46 – 7.38 (m, 3H), 3.78 – 3.63 (m, 2H), 3.59 – 3.50 (m, 2H), 2.65 – 2.43 (m, 4H), 2.05 – 1.84 (m, 4H); **^{13}C NMR (101 MHz, CDCl$_3$)** δ 169.5, 136.8 (t, J = 26.3 Hz), 130.0, 128.6, 124.8 (t, J = 6.2 Hz), 122.6 (t, J = 242.1 Hz), 121.4 (t, J = 242.2 Hz), 42.1 (t, J = 5.4 Hz), 38.7 (t, J = 5.2 Hz), 34.52 (t, J = 27.4 Hz), 34.49 (t, J = 23.6 Hz), 33.7 (t, J = 23.1 Hz), 26.1 (t, J = 3.4 Hz); **^{19}F NMR (376 MHz, CDCl$_3$)** δ -96.7, -98.0. **HRMS (ESI-TOF) m/z:** [M+H]$^+$ Calcd. for $C_{15}H_{18}F_4NO$ 304.1319; found: 304.1317.

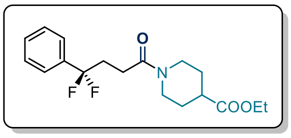

Ethyl-1-(4,4-difluoro-4-phenylbutanoyl)piperidine-4-carboxylate (4m): 76.9 mg, colorless oil, yield: 76%. Eluent: pentane/ethyl acetate = 3/1; **^{1}H NMR (400 MHz, CDCl$_3$)** δ 7.53 – 7.45 (m, 2H), 7.45 – 7.36 (m, 3H), 4.44 – 4.33 (m, 1H), 4.14 (q, J = 7.1 Hz, 2H), 3.79 (d, J = 13.6 Hz, 1H), 3.16 – 3.02 (m, 1H), 2.87 – 2.73 (m, 1H), 2.60 – 2.44 (m, 5H), 1.98 – 1.87 (m, 2H), 1.73 – 1.54 (m, 2H), 1.25 (t, J = 7.1 Hz, 3H); **^{13}C NMR (101 MHz, CDCl$_3$)** δ 174.11, 169.36, 136.94 (t, J = 26.3 Hz), 129.88, 128.50, 124.87 (t, J = 6.2 Hz), 122.69 (t, J = 242.1 Hz), 44.65, 41.15, 40.95, 34.56 (t, J = 27.4 Hz), 28.34, 27.79, 26.24 (t, J = 3.4 Hz), 14.19; **^{19}F NMR (376 MHz, CDCl$_3$)** δ -96.6; **HRMS (ESI-TOF) m/z:** [M+H]$^+$ Calcd. for $C_{18}H_{24}F_2NO_3$ 340.1719; found: 340.1722.

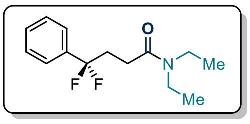

***N*,*N*-Diethyl-4,4-difluoro-4-phenylbutanamide (4n):** 54.5 mg, colorless oil, yield: 72%. Eluent: pentane/ethyl acetate = 3/1; **^{1}H NMR (400 MHz, CDCl$_3$)** δ 7.55 – 7.45 (m, 2H), 7.45 – 7.35 (m, 3H), 3.36 (q, J = 7.1 Hz, 2H), 3.28 (q, J = 7.1 Hz, 2H), 2.61 – 2.47 (m, 4H), 1.15 (t, J = 7.1 Hz, 3H), 1.09 (t, J = 7.1 Hz, 3H); **^{13}C NMR (101 MHz, CDCl$_3$)** δ 170.1, 137.1 (t, J = 26.4 Hz), 129.8, 128.5, 124.9 (t, J = 6.2 Hz), 122.8 (t, J = 242.0 Hz), 41.9, 40.3, 34.7 (t, J = 27.4 Hz), 26.1 (t, J = 3.4 Hz), 14.2, 13.0; **^{19}F NMR (376 MHz, CDCl$_3$)** δ -96.6; **HRMS (ESI-TOF) m/z:** [M+H]$^+$ Calcd. for $C_{14}H_{20}F_2NO$ 256.1507; found: 256.1511.

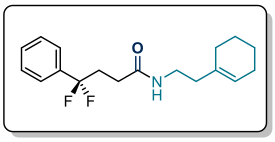

***N*-(2-(Cyclohex-1-en-1-yl)ethyl)-4,4-difluoro-4-phenylbutanamide (4o):** 65.9 mg, colorless oil. yield: 73%. Eluent: pentane/ethyl acetate = 5/1; **^{1}H NMR (400 MHz, CDCl$_3$)** δ 7.54 – 7.36 (m, 5H), 5.53 (s, 1H), 5.45 (s, 1H), 3.29 (dd, J = 12.4, 6.7 Hz, 2H), 2.59 – 2.42 (m, 2H), 2.34 (dd, J = 9.6, 6.1 Hz, 2H), 2.10 (t, J = 6.8 Hz, 2H), 2.04 – 1.94 (m, 2H), 1.90 (s, 2H), 1.66 – 1.49 (m, 4H); **^{13}C NMR (101 MHz,**

$CDCl_3$) δ 170.9, 136.8 (t, J = 26.4 Hz), 134.6, 129.9, 128.5, 124.9 (t, J = 6.2 Hz), 123.6, 122.5, 37.5, 37.3, 34.8 (t, J = 28.0 Hz), 29.6 (t, J = 3.6 Hz), 27.9, 25.2, 22.8, 22.3; **^{19}F NMR (376 MHz, $CDCl_3$)** δ -96.6; **HRMS (ESI-TOF) m/z:** $[M+H]^+$ Calcd. for $C_{18}H_{24}F_2NO$ 308.1820; found: 308.1826.

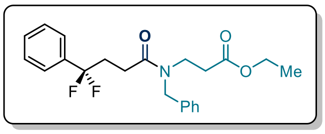

Ethyl-3-(N-benzyl-4,4-difluoro-4-phenylbutanamido)propanoate (4p): 48.3 mg, colorless oil. yield: 41%. Eluent: pentane/ethyl acetate = 2/1; **^1H NMR (400 MHz, $CDCl_3$)** δ 7.53 – 7.11 (m, 10H), 4.64 – 4.52 (m, 2H), 4.15 – 4.05 (m, 2H), 3.66 – 3.53 (m, 2H), 2.73 – 2.45 (m, 6H), 1.28 – 1.20 (m, 3H);**^{13}C NMR (101 MHz, $CDCl_3$)** δ 172.1, 171.7, 171.3, 170.9, 137.3, 136.9, 136.6, 129.8, 129.0, 128.7, 128.5, 128.5, 128.0, 127.7, 127.5, 126.3, 124.92 (t, J = 6.2 Hz), 124.86 (t, J = 6.2 Hz), 122.6 (t, J = 242.0 Hz), 61.0, 60.6, 52.1, 48.4, 43.1, 42.5, 34.7 (t, J = 27.2 Hz), 34.5 (t, J = 27.6 Hz), 33.4, 32.8, 26.4 (t, J = 3.2 Hz), 26.2 (t, J = 3.0 Hz), 14.2, 14.1;**^{19}F NMR (376 MHz, $CDCl_3$)** δ -96.6; **HRMS (ESI-TOF) m/z:** $[M+H]^+$ Calcd. for $C_{22}H_{26}F_2NO_3$ 390.1875; found: 390.1877.

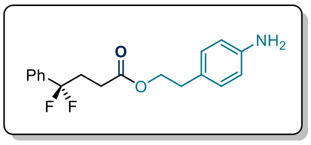

4-Aminophenethyl 4,4-difluoro-4-phenylbutanoate (4q): 53.6 mg, colorless oil, yield: 56% (96% purity). Eluent: pentane/ethyl acetate = 5/1; **^1H NMR (400 MHz, $CDCl_3$)** δ 7.52 – 7.34 (m, 5H), 6.98 (d, J = 8.3 Hz, 2H), 6.66 – 6.55 (m, 2H), 4.19 (t, J = 7.2 Hz, 2H), 3.57 (s, 2H), 2.79 (t, J = 7.1 Hz, 2H), 2.58 – 2.33 (m, 4H); **^{13}C NMR (101 MHz, $CDCl_3$)** δ 172.1, 145.0, 136.6 (t, J = 26.3 Hz), 129.9, 129.7, 128.5, 127.5, 124.9 (t, J = 6.2 Hz), 122.2 (t, J = 242.5 Hz), 115.3, 65.7, 34.4 (t, J = 28.4 Hz), 34.2, 27.8 (t, J = 4.2 Hz); **^{19}F NMR (376 MHz, $CDCl_3$)** δ -96.8; **HRMS (ESI-TOF) m/z:** $[M+H]^+$ Calcd. for $C_{18}H_{20}F_2NO_2$ 320.1457; found: 320.1461.

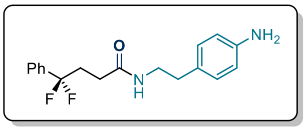

N-(4-Aminophenethyl)-4,4-difluoro-4-phenylbutanamide (4r): 59.1 mg, colorless oil, yield: 62%. Eluent: pentane/ethyl acetate = 2/1; **^1H NMR (400 MHz, $CDCl_3$)** δ 7.52 – 7.34 (m, 5H), 6.96 (d, J = 8.3 Hz, 2H), 6.63 (d, J = 8.3 Hz, 2H), 5.46 (s, 1H), 3.68 (s, 2H), 3.43 (dd, J = 12.9, 6.8 Hz, 2H), 2.67 (t, J = 6.9 Hz, 2H), 2.56 – 2.42 (m, 2H), 2.30 (dd, J = 9.5, 6.3 Hz, 2H); **^{13}C NMR (101 MHz, $CDCl_3$)** δ 171.0, 144.9, 136.8 (t, J = 26.4 Hz), 129.9, 129.6, 128.5, 124.9 (t, J = 6.2 Hz), 122.5 (t, J = 242.2 Hz), 115.4, 40.9, 34.7 (t, J = 28.0 Hz), 34.7, 29.6 (t, J = 3.7 Hz); **^{19}F NMR (376 MHz, $CDCl_3$)** δ -96.6; **HRMS (ESI-TOF) m/z:** $[M+H]^+$ Calcd. for $C_{18}H_{21}F_2N_2O$ 319.1616; found: 319.1615.

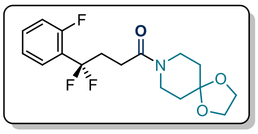

4,4-Difluoro-4-(2-fluorophenyl)-1-(1,4-dioxa-8-azaspiro[4.5]decan-8-yl)butan-1-one (5a): 74.2 mg, colorless oil, yield: 72%. Eluent: pentane/ethyl acetate = 3/1; **^1H NMR (400 MHz, $CDCl_3$)** δ 7.56 – 7.47 (m, 1H), 7.47 – 7.38 (m, 1H), 7.24 – 7.16 (m, 1H), 7.16 – 7.07 (m, 1H), 3.97 (s, 4H), 3.72 – 3.64 (m, 2H), 3.58 – 3.46 (m, 2H), 2.72 – 2.54 (m, 4H), 1.73 – 1.61 (m, 4H); **^{13}C NMR (101 MHz, $CDCl_3$)** δ 169.2, 116.7, 159.6 (d, J = 251.8 Hz), 132.0 (d, J = 8.4 Hz), 126.9 (td, J = 7.7, 2.5 Hz), 124.2 (t, J = 27.1 Hz), 124.0 (d, J = 3.7 Hz), 121.2 (t, J = 243.0 Hz), 116.6 (d, J = 21.7 Hz), 116.5, 106.8, 64.5, 43.4, 39.9, 35.5, 34.7, 33.7 (td, J = 26.3, 3.1 Hz), 26.0 (t, J = 3.4 Hz); **^{19}F NMR (376 MHz, $CDCl_3$)** δ -96.1, -114.47 (t, J = 11.9 Hz, 1F); **HRMS (ESI-TOF) m/z:** $[M+H]^+$ Calcd. for $C_{17}H_{21}F_3NO_3$ 344.1468; found: 344.1467.

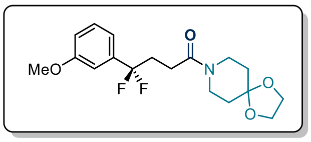

4,4-Difluoro-4-(3-methoxyphenyl)-1-(1,4-dioxa-8-azaspiro[4.5]decan-8-yl)butan-1-one (5b): 82.1 mg, colorless oil, yield: 77%. Eluent: pentane/ethyl acetate = 3/1; **^{1}H NMR (400 MHz, $CDCl_3$)** δ 7.37 – 7.29 (m, 1H), 7.06 (d, *J* = 7.7 Hz, 1H), 7.01 (s, 1H), 6.96 (d, *J* = 8.2 Hz, 1H), 3.97 (s, 4H), 3.82 (s, 3H), 3.74 – 3.61 (m, 2H), 3.56 – 3.45 (m, 2H), 2.63 – 2.41 (m, 4H), 1.75 – 1.58 (m, 4H); **^{13}C NMR (101 MHz, $CDCl_3$)** δ 169.3, 159.6, 138.4 (t, *J* = 26.4 Hz), 129.7, 122.5 (t, *J* = 242.4 Hz), 117.2 (t, *J* = 6.2 Hz), 115.5, 110.5 (t, *J* = 6.4 Hz), 106.8, 64.5, 55.4, 43.4, 39.9, 35.5, 34.7, 34.6 (t, *J* = 27.4 Hz), 26.2 (t, *J* = 3.4 Hz); **^{19}F NMR (376 MHz, $CDCl_3$)** δ -96.4; **HRMS (ESI-TOF) m/z:** $[M+H]^+$ Calcd. for $C_{18}H_{24}F_2NO_4$ 356.1668; found: 356.1674.

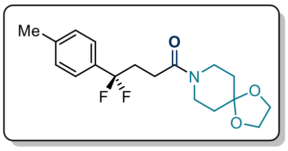

4,4-Difluoro-1-(1,4-dioxa-8-azaspiro[4.5]decan-8-yl)-4-(*p*-tolyl)butan-1-one (5c): 69.5 mg, colorless oil, yield: 68%. Eluent: pentane/ethyl acetate = 3/1; **^{1}H NMR (400 MHz, $CDCl_3$)** δ 7.37 (d, *J* = 8.1 Hz, 2H), 7.22 (d, *J* = 7.9 Hz, 2H), 3.97 (s, 4H), 3.71 – 3.65 (m, 2H), 3.57 – 3.44 (m, 2H), 2.59 – 2.44 (m, 4H), 2.38 (s, 3H), 1.72 – 1.62 (m, 4H); **^{13}C NMR (101 MHz, $CDCl_3$)** δ 169.40, 139.88, 134.08 (t, *J* = 26.5 Hz), 129.14, 124.83 (t, *J* = 6.1 Hz), 122.85 (t, *J* = 241.7 Hz), 106.86, 64.49, 43.38, 39.91, 35.49, 34.68, 34.55 (t, *J* = 27.7 Hz), 26.25 (t, *J* = 3.5 Hz), 21.25; **^{19}F NMR (376 MHz, $CDCl_3$)** δ -95.8; **HRMS (ESI-TOF) m/z:** $[M+H]^+$ Calcd. for $C_{18}H_{24}F_2NO_3$ 340.1719; found: 340.1714.

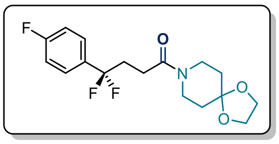

4,4-Difluoro-4-(4-fluorophenyl)-1-(1,4-dioxa-8-azaspiro[4.5]decan-8-yl)butan-1-one (5d): 77.0 mg, colorless oil, yield: 75%. Eluent: pentane/ethyl acetate = 3/1; **^{1}H NMR (400 MHz, $CDCl_3$)** δ 7.52 – 7.43 (m, 2H), 7.17 – 7.03 (m, 2H), 3.97 (s, 4H), 3.73 – 3.62 (m, 2H), 3.57 – 3.47 (m, 2H), 2.62 – 2.42 (m, 4H), 1.75 – 1.61 (m, 4H); **^{13}C NMR (101 MHz, $CDCl_3$)** δ 169.2, 163.5 (d, *J* = 249.2 Hz), 133.1 (t, *J* = 27.0 Hz), 127.0 (dt, *J* = 8.5, 6.2 Hz), 122.4 (t, *J* = 242.2 Hz), 115.6 (d, *J* = 21.9 Hz), 106.8, 64.5, 43.4, 39.9, 35.5, 34.7, 34.6 (t, *J* = 27.4 Hz), 26.1 (t, *J* = 3.3 Hz); **^{19}F NMR (376 MHz, $CDCl_3$)** δ -95.7, -111.1; **HRMS (ESI-TOF) m/z:** $[M+H]^+$ Calcd. for $C_{17}H_{21}F_3NO_3$ 344.1468; found: 344.1467.

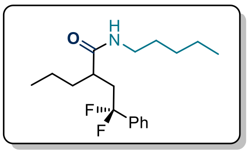

2-(2,2-Difluoro-2-phenylethyl)-*N*-pentylpentanamide (6a): 58.2 mg, colorless oil, yield: 62% (95% purity). Eluent: pentane/ethyl acetate = 5/1; **^{1}H NMR (400 MHz, $CDCl_3$)** δ 7.52 – 7.34 (m, 6H), 5.44 (s, 1H), 3.27 – 3.10 (m, 2H), 2.84 – 2.61 (m, 1H), 2.41 – 2.27 (m, 1H), 2.19 – 2.00 (m, 1H), 1.72 – 1.61 (m, 1H), 1.53 – 1.41 (m, 2H), 1.38 – 1.23 (m, 7H), 0.94 – 0.85 (m, 6H); **^{13}C NMR (101 MHz, $CDCl_3$)** δ 174.5, 137.2 (t, *J* = 26.3 Hz), 129.8, 128.4, 124.9 (t, *J* = 6.2 Hz), 122.5 (t, J = 241.3 Hz), 41.8 (t, *J* = 26.9 Hz), 41.6, 39.5, 36.0, 29.2, 29.0, 22.3, 20.5, 14.0, 13.9; **^{19}F NMR (376 MHz, $CDCl_3$)** δ -95.6; **HRMS (ESI-TOF) m/z:** $[M+H]^+$ Calcd. for $C_{18}H_{28}F_2NO$ 312.2133; found: 312.2133.

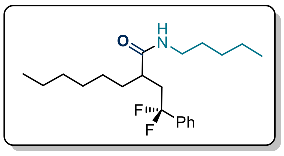

2-(2,2-Difluoro-2-phenylethyl)-*N*-pentyloctanamide (6b): 57.9 mg, colorless oil, yield: 55%. Eluent: pentane/ethyl acetate = 5/1; **^{1}H NMR (400 MHz, $CDCl_3$)** δ 7.50 – 7.37 (m, 5H), 5.44 (s, 1H), 3.31 – 3.06 (m, 2H), 2.83 – 2.59 (m, 1H), 2.38 – 2.28 (m, 1H), 2.21 – 1.98 (m, 1H), 1.78 – 1.62 (m, 1H), 1.53 – 1.38 (m, 3H), 1.34 – 1.21 (m, 12H), 0.92 – 0.83 (m, 6H); **^{13}C NMR (101 MHz, $CDCl_3$)** δ 174.6, 137.2 (t, *J* = 26.3 Hz), 129.8, 128.4, 124.9 (t, *J* = 6.2 Hz), 122.5 (t, J = 241.0 Hz), 41.8 (t, *J* = 26.7 Hz), 39.5, 33.9, 31.7, 29.2, 29.1, 29.1, 27.3, 22.6, 22.4, 14.05,

13.99; **^{19}F NMR (376 MHz, CDCl$_3$)** δ -95.6; **HRMS (ESI-TOF) m/z:** [M+H]$^+$ Calcd. for $C_{21}H_{34}F_2NO$ 354.2603; found: 354.2609.

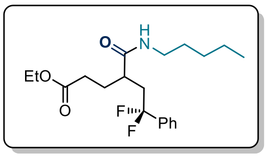

Ethyl 6,6-difluoro-4-(pentylcarbamoyl)-6-phenylhexanoate (6c): 75.4 mg, colorless oil, yield: 68%. Eluent: pentane/ethyl acetate = 5/1; **^{1}H NMR (400 MHz, CDCl$_3$)** δ 7.52 – 7.35 (m, 5H), 5.72 (s, 1H), 4.12 (q, J = 7.1 Hz, 2H), 3.26 – 3.11 (m, 2H), 2.84 – 2.65 (m, 1H), 2.58 – 2.47 (m, 1H), 2.42 – 2.21 (m, 2H), 2.18 – 2.02 (m, 1H), 2.00 – 1.89 (m, 1H), 1.85 – 1.73 (m, 1H), 1.55 – 1.40 (m, 2H), 1.36 – 1.22 (m, 7H), 0.89 (t, J = 6.9 Hz, 3H); **^{13}C NMR (101 MHz, CDCl$_3$)** δ 173.6, 173.1, 137.0 (t, J = 26.3 Hz), 129.9, 128.4, 124.8 (t, J = 6.2 Hz), 122.3 (t, J = 242.8 Hz), 60.5, 41.6 (t, J = 27.1 Hz), 40.3, 39.6, 31.6, 29.1, 29.0, 28.8, 22.3, 14.2, 14.0; **^{19}F NMR (376 MHz, CDCl$_3$)** δ -95.2 (d, J = 244.5 Hz, 1F), -96.1 (d, J = 244.3 Hz, 1F); **HRMS (ESI-TOF) m/z:** [M+H]$^+$ Calcd. for $C_{20}H_{30}F_2NO_3$ 370.2188; found: 370.2193.

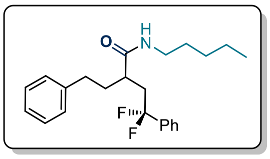

4,4-Difluoro-*N*-pentyl-2-phenethyl-4-phenylbutanamide (6d): 52.0 mg, colorless oil, yield: 46%. Eluent: pentane/ethyl acetate = 5/1; **^{1}H NMR (400 MHz, CDCl$_3$)** δ 7.48 – 7.36 (m, 5H), 7.31 – 7.24 (m, 2H), 7.23 – 7.09 (m, 3H), 5.39 (s, 1H), 3.29 – 3.11 (m, 2H), 2.84 – 2.60 (m, 2H), 2.59 – 2.49 (m, 1H), 2.42 – 2.33 (m, 1H), 2.22 – 1.96 (m, 2H), 1.82 – 1.71 (m, 1H), 1.53 – 1.43 (m, 2H), 1.35 – 1.25 (m, 4H), 0.90 (t, J = 6.9 Hz, 3H); **^{13}C NMR (101 MHz, CDCl$_3$)** δ 174.1, 141.2, 137.1 (t, J = 26.3 Hz), 129.9, 128.5, 128.4, 126.1, 124.9 (t, J = 6.2 Hz), 122.4 (t, J = 242.3 Hz), 41.8 (t, J = 27.1 Hz), 41.0, 39.6, 35.0, 33.3, 29.2, 29.1, 22.4, 14.0; **^{19}F NMR (376 MHz, CDCl$_3$)** δ -94.8 – -95.5 (m, 1F), -95.5 – -96.2 (m, 1F); **HRMS (ESI-TOF) m/z:** [M+H]$^+$ Calcd. for $C_{23}H_{30}F_2NO$ 374.2290; found: 374.2299.

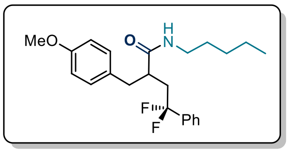

4,4-Difluoro-2-(4-methoxybenzyl)-N-pentyl-4-phenylbutanamide (6e): 57.4 mg, colorless oil, yield: 50%. Eluent: pentane/ethyl acetate = 3/1; **^{1}H NMR (400 MHz, CDCl$_3$)** δ 7.55 – 7.32 (m, 5H), 7.05 (d, J = 8.6 Hz, 2H), 6.89 – 6.67 (m, 2H), 5.09 (s, 1H), 3.77 (s, 3H), 3.17 – 2.93 (m, 2H), 2.92 – 2.63 (m, 3H), 2.61 – 2.48 (m, 1H), 2.28 – 2.08 (m, 1H), 1.32 – 1.17 (m, 4H), 1.14 – 1.04 (m, 2H), 0.85 (t, J = 7.3 Hz, 3H); **^{13}C NMR (101 MHz, CDCl$_3$)** δ 173.5, 158.3, 137.1 (t, J = 26.3 Hz), 131.0, 129.9, 129.9, 128.4, 124.9 (t, J = 6.2 Hz), 122.5 (t, J = 242.5 Hz), 113.8, 55.2, 44.4, 41.1 (t, J = 27.1 Hz), 39.4, 39.0, 29.0, 28.9, 22.3, 13.9; **^{19}F NMR (376 MHz, CDCl$_3$)** δ -95.0 (d, J = 244.5 Hz, 1F), -95.7 (d, J = 244.6 Hz, 1F); **HRMS (ESI-TOF) m/z:** [M+H]$^+$ Calcd. for $C_{23}H_{30}F_2NO_2$ 390.2239; found: 390.2243.

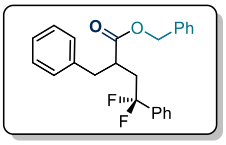

Benzyl 2-benzyl-4,4-difluoro-4-phenylbutanoate (6f): 52.2 mg, colorless oil, yield: 46%. Eluent: pentane/ethyl acetate = 50/1; **^{1}H NMR (400 MHz, CDCl$_3$)** δ 7.45 – 7.33 (m, 5H), 7.33 – 7.27 (m, 3H), 7.27 – 7.19 (m, 3H), 7.19 – 7.12 (m, 2H), 7.11 – 7.03 (m, 2H), 4.93 (q, J = 12.3 Hz, 2H), 3.10 – 2.90 (m, 2H), 2.85 – 2.60 (m, 2H), 2.30 – 2.16 (m, 1H); **^{13}C NMR (101 MHz, CDCl$_3$)** δ 174.4, 137.8, 136.6 (t, J = 26.2 Hz), 135.6, 129.9, 129.0, 128.6, 128.5, 128.4, 128.2, 128.2, 126.8, 125.0 (t, J = 6.3 Hz), 122.2 (t, J = 243.0 Hz), 66.5, 41.8 (t, J = 2.7 Hz), 40.6 (t, J = 27.8 Hz), 39.0; **^{19}F NMR (376 MHz, CDCl$_3$)** δ -94.4 (d, J = 246.5 Hz, 1F), -95.6 (d, J = 246.5 Hz, 1F); **HRMS (ESI-TOF) m/z:** [M+H]$^+$ Calcd. for $C_{24}H_{23}F_2O_2$ 381.1661; found: 381.1664.

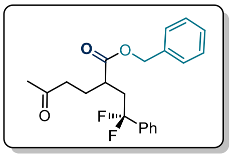

Benzyl 2-benzyl-4,4-difluoro-4-phenylbutanoate (6g): 76.9 mg, colorless oil, yield: 71%. Eluent: pentane/ethyl acetate = 10/1; **^{1}H NMR (400 MHz, $CDCl_3$)** δ 7.45 – 7.32 (m, 10H), 5.09 (d, *J* = 12.2 Hz, 1H), 4.99 (d, *J* = 12.2 Hz, 1H), 2.80 – 2.66 (m, 2H), 2.37 – 2.30 (m, 2H), 2.23 – 2.11 (m, 1H), 2.02 (s, 3H), 1.90 – 1.81 (m, 2H); **^{13}C NMR (101 MHz, $CDCl_3$)** δ 207.1, 174.4, 136.6 (t, *J* = 26.2 Hz), 135.7, 130.0, 128.6, 128.5, 128.4, 125.0 (t, *J* = 6.3 Hz), 122.0 (t, *J* = 243.0 Hz), 66.6, 41.4 (t, *J* = 27.8 Hz), 40.3, 39.1 (t, *J* = 2.9 Hz), 29.9, 26.8; **^{19}F NMR (376 MHz, $CDCl_3$)** δ -95.0 (d, *J* = 246.5 Hz, 1F), -95.7 (d, *J* = 246.4 Hz, 1F); **HRMS (ESI-TOF) m/z:** $[M+H]^+$ Calcd. for $C_{21}H_{23}F_2O_3$ 361.1610; found: 361.1615.

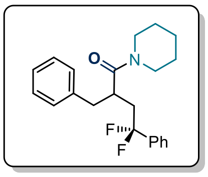

2-Benzyl-4,4-difluoro-4-phenyl-1-(piperidin-1-yl)butan-1-one (6h): 68.7 mg, colorless oil, yield: 64%. Eluent: pentane/ethyl acetate = 5/1; **^{1}H NMR (400 MHz, $CDCl_3$)** δ 7.51 – 7.42 (m, 2H), 7.42 – 7.32 (m, 3H), 7.28 – 7.23 (m, 2H), 7.22 – 7.10 (m, 3H), 3.59 – 3.48 (m, 1H), 3.41 – 3.31 (m, 1H), 3.27 – 3.18 (m, 1H), 3.13 – 2.82 (m, 4H), 2.73 (dd, *J* = 13.0, 5.7 Hz, 1H), 2.31 – 2.13 (m, 1H), 1.46 – 1.35 (m, 3H), 1.31 – 1.20 (m, 2H), 0.84 – 0.67 (m, 1H); **^{13}C NMR (101 MHz, $CDCl_3$)** δ 172.1, 139.0, 137.0 (t, *J* = 26.3 Hz), 129.8, 129.2, 128.4, 128.4, 126.5, 124.9 (t, *J* = 6.2 Hz), 122.5 (t, *J* = 243.5 Hz), 46.6, 42.9, 41.8 (t, *J* = 26.7 Hz), 40.4, 36.9, 25.7, 25.4, 24.4; **^{19}F NMR (376 MHz, $CDCl_3$)** δ -94.1 (d, *J* = 244.5 Hz, 1F), -96.4 (d, *J* = 244.5 Hz, 1F); **HRMS (ESI-TOF) m/z:** [M+H]+ Calcd. for $C_{22}H_{26}F_2NO$ 358.1977; found: 358.1979.

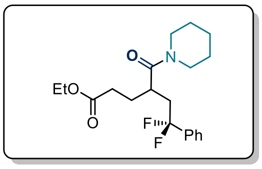

Ethyl 6,6-difluoro-6-phenyl-4-(piperidine-1-carbonyl)hexanoate (6i): 87.5 mg, colorless oil, yield: 79%. Eluent: pentane/ethyl acetate = 3/1; **^{1}H NMR (400 MHz, $CDCl_3$)** δ 7.50 – 7.36 (m, 5H), 4.19 – 4.05 (m, 2H), 3.58 – 3.38 (m, 4H), 3.31 – 3.19 (m, 1H), 2.92 – 2.73 (m, 1H), 2.39 – 2.21 (m, 2H), 2.19 – 2.05 (m, 1H), 2.01 – 1.94 (m, 1H), 1.84 – 1.74 (m, 1H), 1.66 – 1.47 (m, 6H), 1.25 (t, *J* = 7.1 Hz, 3H); **^{13}C NMR (101 MHz, $CDCl_3$)** δ 172.9, 172.4, 137.0 (t, *J* = 26.3 Hz), 129.8, 128.4, 124.9 (t, *J* = 6.2 Hz), 122.4 (t, *J* = 243.0 Hz), 60.4, 46.8, 43.2, 41.6 (t, *J* = 26.8 Hz), 33.5, 31.3, 28.7, 26.4, 25.6, 24.6, 14.2; **^{19}F NMR (376 MHz, $CDCl_3$)** δ -94.5 (d, *J* = 244.0 Hz, 1F), -96.7 (d, *J* = 243.9 Hz, 1F); **HRMS (ESI-TOF) m/z:** [M+H]+ Calcd. for $C_{20}H_{28}F_2NO_3$ 368.2032; found: 368.2041.

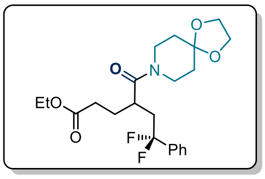

Ethyl-6,6-difluoro-6-phenyl-4-(1,4-dioxa-8-azaspiro[4.5]decane-8-carbonyl)hexanoate (6j): 69.3 mg, colorless oil, yield: 54%. Eluent: pentane/ethyl acetate = 3/1; **^{1}H NMR (400 MHz, $CDCl_3$)** δ 7.56 – 7.31 (m, 5H), 4.20 – 4.04 (m, 2H), 3.97 (s, 4H), 3.81 – 3.71 (m, 1H), 3.69 – 3.51 (m, 3H), 3.32 – 3.23 (m, 1H), 2.90 – 2.72 (m, 1H), 2.39 – 2.06 (m, 3H), 2.02 – 1.91 (m, 1H), 1.84 – 1.62 (m, 5H), 1.25 (t, *J* = 7.1 Hz, 3H); **^{13}C NMR (101 MHz, $CDCl_3$)** δ 172.9, 172.5, 136.9 (t, *J* = 26.3 Hz), 129.9, 128.4, 124.9 (t, *J* = 6.2 Hz), 122.4, 106.9, 64.4, 64.4, 60.5, 43.7, 41.7 (t, *J* = 26.7 Hz), 40.2, 35.4, 34.8, 33.5, 31.2, 28.7, 14.2; **^{19}F NMR (376 MHz, $CDCl_3$)** δ -94.6 (d, *J* = 243.9 Hz, 1F), -96.7 (d, *J* = 243.9 Hz, 1F); **HRMS (ESI-TOF) m/z:** [M+H]+ Calcd. for $C_{22}H_{30}F_2NO_5$ 426.2087; found: 426.2090.

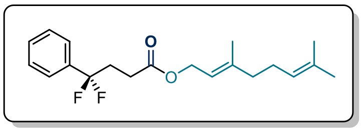

(*E*)-3,7-Dimethylocta-2,6-dien-1-yl-4,4-difluoro-4-phenylbutanoate (7a): 73.7 mg, colorless oil, yield: 73%. Eluent: pentane/ethyl acetate = 50/1; **^{1}H NMR (400 MHz, $CDCl_3$)** δ 7.56 – 7.34 (m, 5H), 5.32 (t, *J* = 7.2 Hz, 1H), 5.15 – 4.99 (m, 1H), 4.54 (d, *J* = 7.3 Hz, 2H), 2.57 – 2.41 (m, 4H), 2.16 – 2.01 (m, 4H), 1.76 (s, 3H), 1.67 (s, 3H), 1.59 (s, 3H); **^{13}C NMR (101 MHz, $CDCl_3$)** δ 172.1, 142.9, 136.7 (t, *J* = 26.4 Hz), 132.2, 129.9, 128.5, 124.9 (t, *J* = 6.3 Hz), 123.5, 122.2 (t, *J* = 242.6 Hz), 118.9, 61.4, 34.4 (t, *J* = 28.5 Hz), 32.2, 27.8 (t, *J* = 4.2 Hz), 26.6, 25.7, 23.5, 17.7; **^{19}F NMR (376 MHz, $CDCl_3$)** δ -96.9; **HRMS (ESI-TOF) m/z:** [M+H]+ Calcd. for $C_{20}H_{27}F_2O_2$ 337.1974; found: 337.1977.

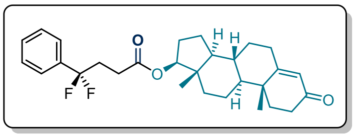

(8*R*,9*S*,10*R*,13*S*,14*S*,17*S*)-10,13-Dimethyl-3-oxo-2,3,6,7,8,9,10,11,12,13,14,15,16,17-tetradecahydro-1*H*-cyclopenta[a]phenanthren-17-yl 4,4-difluoro-4-phenylbutanoate (7b): 60.1 mg, colorless oil, yield: 43%. Eluent: pentane/ethyl acetate = 10/1; **^{1}H NMR (400 MHz, $CDCl_3$)** δ 7.52 – 7.37 (m, 5H), 5.73 (s, 1H), 4.60 (dd, *J* = 9.0, 8.0 Hz, 1H), 2.55 – 2.24 (m, 8H), 2.22 – 2.11 (m, 1H), 2.05 – 1.99 (m, 1H), 1.88 – 1.32 (m, 10H), 1.19 (s, 3H), 1.09 – 0.90 (m, 3H), 0.83 (s, 3H); **^{13}C NMR (101 MHz, $CDCl_3$)** δ 199.4, 172.1, 170.9, 136.7 (t, *J* = 26.3 Hz), 129.9, 128.5, 124.9 (t, *J* = 6.2 Hz), 124.0, 122.2 (t, *J* = 242.5 Hz), 82.8, 53.7, 50.2, 42.5, 38.6, 36.6, 35.7, 35.4, 34.4 (t, *J* = 28.4 Hz), 33.9, 32.7, 31.5, 27.9 (t, *J* = 4.2 Hz), 27.4, 23.5, 20.5, 17.4, 12.1; **^{19}F NMR (376 MHz, $CDCl_3$)** δ -96.8 (s, 2F); **HRMS (ESI-TOF) m/z:** [M+H]+ Calcd. for $C_{29}H_{37}F_2O_3$ 471.2705; found: 471.2703.

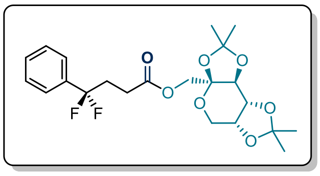

((3a*S*,5a*R*,8a*R*,8b*S*)-2,2,7,7-Tetramethyltetrahydro-3a*H*-bis([1,3]dioxolo)[4,5-*b*:4',5'-d]pyran-3a-yl)methyl-4,4-difluoro-4-phenylbutanoate (7c): 78.3 mg, colorless oil, yield: 59%. Eluent: pentane/ethyl acetate = 10/1; **^{1}H NMR (400 MHz, $CDCl_3$)** δ 7.52 – 7.35 (m, 5H), 4.40 (d, *J* = 11.7 Hz, 1H), 4.28 (d, *J* = 2.6 Hz, 1H), 4.23 (dd, *J* = 7.9, 1.0 Hz, 1H), 4.03 (d, *J* = 11.7 Hz, 1H), 3.90 (dd, *J* = 13.0, 1.8 Hz, 1H), 3.76 (d, *J* = 13.0 Hz, 1H), 2.65 – 2.41 (m, 4H), 1.54 (s, 3H), 1.46 (s, 3H), 1.38 (s, 3H), 1.34 (s, 3H); **^{13}C NMR (101 MHz, $CDCl_3$)** δ 171.5, 136.6 (t, *J* = 26.3 Hz), 130.0, 128.6, 124.9 (t, *J* = 6.2 Hz), 122.1 (t, *J* = 242.5 Hz), 109.1, 108.8, 101.4, 70.7, 70.6, 70.0, 65.7, 61.3, 34.3 (t, *J* = 28.5 Hz), 27.6 (t, *J* = 4.2 Hz), 26.4, 25.9, 25.2, 24.0; **^{19}F NMR (376 MHz, $CDCl_3$)** δ -90.1; **HRMS (ESI-TOF) m/z:** [M+H]+ Calcd. for $C_{22}H_{29}F_2O_7$ 443.1876; found: 443.1873.

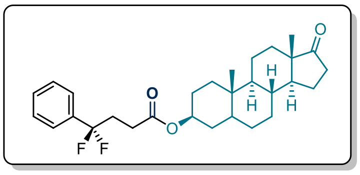

(3*S*,8*R*,9*S*,10*S*,13*S*,14*S*)-10,13-Dimethyl-17-oxohexadecahydro-1*H*-cyclopenta[a]phenanthren-3-yl 4,4-difluoro-4-phenylbutanoate (7d): 98.5 mg, colorless oil, yield: 70%. Eluent: pentane/ethyl acetate = 20/1; **^{1}H NMR (400 MHz, $CDCl_3$)** δ 7.52 – 7.34 (m, 5H), 4.73 – 4.62 (m, 1H), 2.59 – 2.32 (m, 5H), 2.11 – 2.00 (m, 1H), 1.96 – 1.87 (m, 1H), 1.82 – 1.70 (m, 4H), 1.66 – 1.44 (m, 5H), 1.40 – 1.17 (m, 7H), 1.07 – 0.91 (m, 2H), 0.84 (d, *J* = 4.4 Hz, 6H), 0.75 – 0.65 (m, 1H); **^{13}C NMR (101 MHz, $CDCl_3$)** δ 221.1, 171.6, 136.7 (t, *J* = 26.4 Hz), 129.9, 128.5, 124.9 (t, *J* = 6.2 Hz), 122.2 (t, *J* = 242.6 Hz), 73.9, 54.3, 51.3, 47.8, 44.6, 36.7, 35.8, 35.6, 35.0, 34.4 (t, *J* = 28.4 Hz), 33.9, 31.5, 30.8, 28.2,

28.1 (t, J = 4.1 Hz), 27.3, 21.8, 20.5, 13.8, 12.2; **^{19}F NMR (376 MHz, CDCl$_3$)** δ -96.8; **HRMS (ESI-TOF) m/z:** [M+H]+ Calcd. for $C_{29}H_{39}F_2O_3$ 473.2862; found: 473.2861.

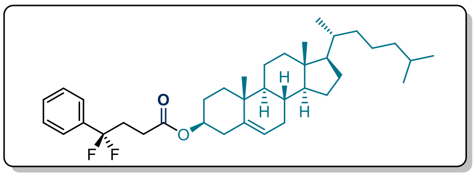

(3S,8S,9S,10R,13R,14S,17R)-10,13-Dimethyl-17-((R)-6-methylheptan-2-yl)-2,3,4,7,8,9,10,11,12,13,14,15,16,17-tetradecahydro-1H-cyclopenta[a]phenanthren-3-yl 4,4-difluoro-4-phenylbutanoate (7e): 94.6 mg, colorless oil, yield: 55%. Eluent: pentane/ethyl acetate = 30/1; **^{1}H NMR (400 MHz, CDCl$_3$)** δ 7.56 – 7.34 (m, 5H), 5.36 (d, J = 4.3 Hz, 1H), 4.65 – 4.54 (m, 1H), 2.55 – 2.40 (m, 4H), 2.29 (d, J = 7.8 Hz, 2H), 2.05 – 1.91 (m, 2H), 1.90 – 1.78 (m, 3H), 1.60 – 1.41 (m, 7H), 1.40 – 1.24 (m, 4H), 1.22 – 0.94 (m, 13H), 0.91 (d, J = 6.5 Hz, 3H), 0.88 – 0.84 (m, 6H), 0.67 (s, 3H); **^{13}C NMR (101 MHz, CDCl$_3$)** δ 171.5, 139.6, 136.7 (t, J = 26.4 Hz), 129.9, 128.5, 124.9 (t, J = 6.2 Hz), 122.8, 122.3 (t, J = 242.5 Hz), 74.4, 56.0, 56.2, 50.0, 42.3, 39.7, 39.5, 38.1, 37.0, 36.6, 36.2, 35.8, 34.5 (t, J = 28.4 Hz), 31.92, 31.87, 28.2, 28.1 (t, J = 4.1 Hz), 28.0, 27.7, 24.3, 23.8, 22.8, 22.6, 21.0, 19.3, 18.7, 11.9; **^{19}F NMR (376 MHz, CDCl$_3$)** δ -96.8; **HRMS (ESI-TOF) m/z:** [M+H]+ Calcd. for $C_{37}H_{55}F_2O_2$ 569.4165; found: 569.4169.

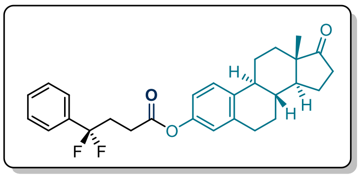

(8R,9S,13S,14S)-13-Methyl-17-oxo-7,8,9,11,12,13,14,15,16,17-Decahydro-6H-cyclopenta[a]phenanthren-3-yl 4,4-difluoro-4-phenylbutanoate (7f): 81.5 mg, colorless oil, yield: 60% (96% purity). Eluent: pentane/ethyl acetate = 10/1; **^{1}H NMR (400 MHz, CDCl$_3$)** δ 7.60 – 7.37 (m, 5H), 7.29 – 7.24 (m, 1H), 6.85 – 6.74 (m, 2H), 2.93 – 2.85 (m, 2H), 2.81 – 2.72 (m, 2H), 2.68 – 2.45 (m, 3H), 2.44 – 2.34 (m, 1H), 2.32 – 2.22 (m, 1H), 2.19 – 2.10 (m, 1H), 2.10 – 1.93 (m, 3H), 1.66 – 1.40 (m, 6H), 0.90 (s, 3H); **^{13}C NMR (101 MHz, CDCl$_3$)** δ 220.72, 171.00, 148.45, 138.08, 137.54, 136.57 (t, J = 26.3 Hz), 130.06, 128.62, 126.45, 124.95 (t, J = 6.2 Hz), 122.19 (t, J = 242.7 Hz), 121.47, 118.62, 77.41, 77.09, 76.77, 50.44, 47.95, 44.16, 38.00, 35.87, 34.45 (t, J = 28.5 Hz), 31.57, 29.41, 27.94 (t, J = 4.2 Hz), 26.34, 25.77, 21.60, 13.85; **^{19}F NMR (376 MHz, CDCl$_3$)** δ -96.8; **HRMS (ESI-TOF) m/z:** [M+H]+ Calcd. for $C_{28}H_{31}F_2O_3$ 453.2236; found: 453.2230.

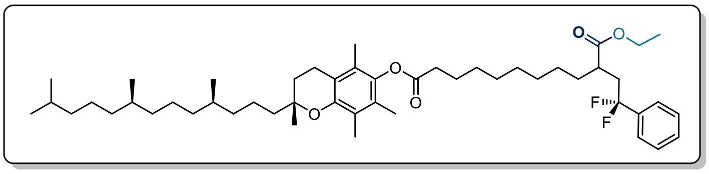

1-Ethyl-11-((R)-2,5,7,8-tetramethyl-2-((4R,8R)-4,8,12-trimethyltridecyl)chroman-6-yl) 2-(2,2-difluoro-2-phenylethyl)undecanedioate (7g): 82.6 mg, colorless oil, yield: 52%. Eluent: pentane/ethyl acetate = 20/1; **^{1}H NMR (400 MHz, CDCl$_3$)** δ 7.52 – 7.33 (m, 5H), 4.13 – 3.99 (m, 2H), 2.75 – 2.54 (m, 6H), 2.24 – 2.11 (m, 1H), 2.08 (s, 3H), 2.00 (s, 3H), 1.96 (s, 3H), 1.86 – 1.70 (m, 4H), 1.65 – 1.02 (m, 41H), 0.89 – 0.81 (m, 12H); **^{13}C NMR (101 MHz, CDCl$_3$)** δ 175.2, 172.4, 149.4, 140.5, 136.9 (t, J = 26.4 Hz), 129.8, 128.4, 126.7, 125.0 (t, J = 6.3 Hz), 124.9, 123.0, 122.3 (t, J = 242.9 Hz), 117.4, 75.0, 60.5, 41.4 (t, J = 27.6 Hz), 39.8, 39.4, 37.5, 37.4, 37.3, 34.1, 33.3, 32.8, 32.7, 29.3, 29.22, 29.18, 28.0, 26.9, 25.1, 24.8, 24.5, 22.8, 22.6, 21.0, 20.6, 19.8, 19.7, 14.2, 13.0, 12.1, 11.8; **^{19}F NMR (376 MHz, CDCl$_3$)** δ -95.3; **HRMS (ESI-TOF) m/z:** [M+H]+ Calcd. for $C_{50}H_{79}F_2O_5$ 797.5890; found: 797.5891.

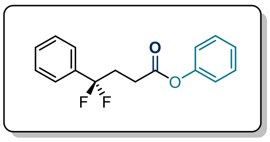

Phenyl 4,4-difluoro-4-phenylbutanoate (8b): 27.2 mg, colorless oil, yield: 33% (96% purity). Eluent: pentane/ethyl acetate = 50/1; **^{1}H NMR (400 MHz, $CDCl_3$)** δ 7.54 – 7.48 (m, 2H), 7.48 – 7.41 (m, 3H), 7.40 – 7.33 (m, 2H), 7.22 (t, *J* = 7.5 Hz, 1H), 7.04 (d, *J* = 7.8 Hz, 2H), 2.81 – 2.76 (m, 2H), 2.68 – 2.53 (m, 2H); **^{13}C NMR (101 MHz, $CDCl_3$)** δ 170.7, 150.6, 136.6 (t, *J* = 26.2 Hz), 130.1, 129.5, 128.6, 126.0, 125.0 (t, *J* = 6.2 Hz), 122.2 (t, *J* = 242.8 Hz), 121.5, 34.4 (t, *J* = 28.5 Hz), 28.0 (t, *J* = 4.2 Hz); **^{19}F NMR (376 MHz, $CDCl_3$)** δ -96.9 (t, *J* = 16.1 Hz, 2F); **HRMS (ESI-TOF) m/z:** [M+H]+ Calcd. for $C_{16}H_{15}F_2O_2$ 277.1035; found: 277.1031.

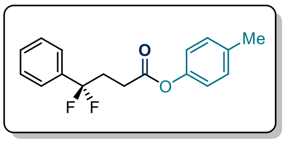

***p*-Tolyl 4,4-difluoro-4-phenylbutanoate (8c)**: 49.4 mg, colorless oil, yield: 57%. Eluent: pentane/ethyl acetate = 50/1; **^{1}H NMR (400 MHz, $CDCl_3$)** δ 7.55 – 7.47 (m, 2H), 7.47 – 7.39 (m, 3H), 7.15 (d, *J* = 8.2 Hz, 2H), 6.92 (d, *J* = 8.4 Hz, 2H), 2.80 – 2.73 (m, 2H), 2.67 – 2.51 (m, 2H), 2.33 (s, 3H); **^{13}C NMR (101 MHz, $CDCl_3$)** δ 170.94, 148.33, 136.58 (t, *J* = 26.3 Hz), 135.61, 130.05, 129.97, 128.62, 124.95 (t, *J* = 6.2 Hz), 122.19 (t, *J* = 242.7 Hz), 121.11, 34.44 (t, *J* = 28.6 Hz), 27.94 (t, *J* = 4.2 Hz), 20.88; **^{19}F NMR (376 MHz, $CDCl_3$)** δ -96.9; **HRMS (ESI-TOF) m/z:** [M+H]+ Calcd. for $C_{17}H_{17}F_2O_2$ 291.1191; found: 291.1197.

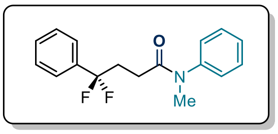

4,4-Difluoro-*N*-methyl-*N*,4-diphenylbutanamide (9c): 21.1 mg, colorless oil, yield: 23%. Eluent: pentane/ethyl acetate = 3/1; **^{1}H NMR (400 MHz, $CDCl_3$)** δ 7.44 – 7.32 (m, 8H), 7.15 (d, *J* = 7.4 Hz, 2H), 3.25 (s, 3H), 2.55 – 2.40 (m, 2H), 2.35 – 2.23 (m, 2H); **^{13}C NMR (101 MHz, $CDCl_3$)** δ 171.3, 143.7, 136.9 (t, *J* = 26.4 Hz), 129.9, 129.8, 128.4, 128.0, 127.2, 124.8 (t, *J* = 6.2 Hz), 122.5, 37.5, 34.7 (t, *J* = 27.6 Hz), 27.4 (t, *J* = 3.6 Hz); **^{19}F NMR (376 MHz, $CDCl_3$)** δ -96.9; **HRMS (ESI-TOF) m/z:** [M+H]+ Calcd. for $C_{17}H_{18}F_2NO$ 290.1351; found: 290.1354.

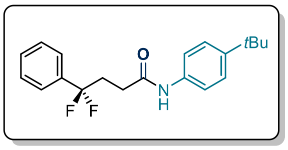

***N*-(4-(*tert*-Butyl)phenyl)-4,4-difluoro-4-phenylbutanamide (9d)**: 50.7 mg, colorless oil, yield: 52%. Eluent: pentane/ethyl acetate = 5/1; **^{1}H NMR (400 MHz, $CDCl_3$)** δ 7.52 – 7.28 (m, 10H), 2.66 – 2.48 (m, 4H), 1.29 (s, 9H); **^{13}C NMR (101 MHz, $CDCl_3$)** δ 169.4, 147.5, 136.8 (t, *J* = 26.3 Hz), 135.0, 130.0, 128.6, 125.8, 124.9 (t, *J* = 6.2 Hz), 122.3 (t, *J* = 212.9 Hz), 119.9, 34.4, 31.4, 30.5, 34.6 (t, *J* = 28.1 Hz); **^{19}F NMR (376 MHz, $CDCl_3$)** δ -96.6; **HRMS (ESI-TOF) m/z:** [M+H]+ Calcd. for $C_{20}H_{24}F_2NO$ 332.1820; found: 332.1823.

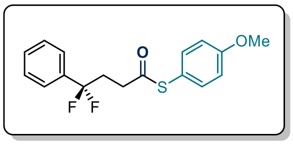

***S*-(4-Methoxyphenyl) 4,4-difluoro-4-phenylbutanethioate (16)**: 30.7 mg, colorless oil, yield: 32%. Eluent: pentane/ethyl acetate = 30/1; **^{1}H NMR (400 MHz, $CDCl_3$)** δ 7.53 – 7.37 (m, 5H), 7.31 – 7.25 (m, 2H), 6.98 – 6.88 (m, 2H), 3.81 (s, 3H), 2.85 (dd, *J* = 8.9, 6.7 Hz, 2H), 2.61 – 2.43 (m, 2H); **^{13}C NMR (101 MHz, $CDCl_3$)** δ 196.8, 160.8, 136.5 (t, *J* = 26.3 Hz), 136.1, 130.0, 128.6, 124.9 (t, *J* = 6.2 Hz), 122.1 (t, *J* = 242.8 Hz), 118.0, 114.9, 55.4, 36.4 (t, *J* = 3.5 Hz), 34.4 (t, *J* = 28.7 Hz); **^{19}F NMR (376 MHz, $CDCl_3$)** δ -96.6; **HRMS (ESI-TOF) m/z:** [M+H]+ Calcd. for $C_{17}H_{17}F_2O_2S$ 323.0912; found: 323.0917.

7. Copy of NMR Spectrum.

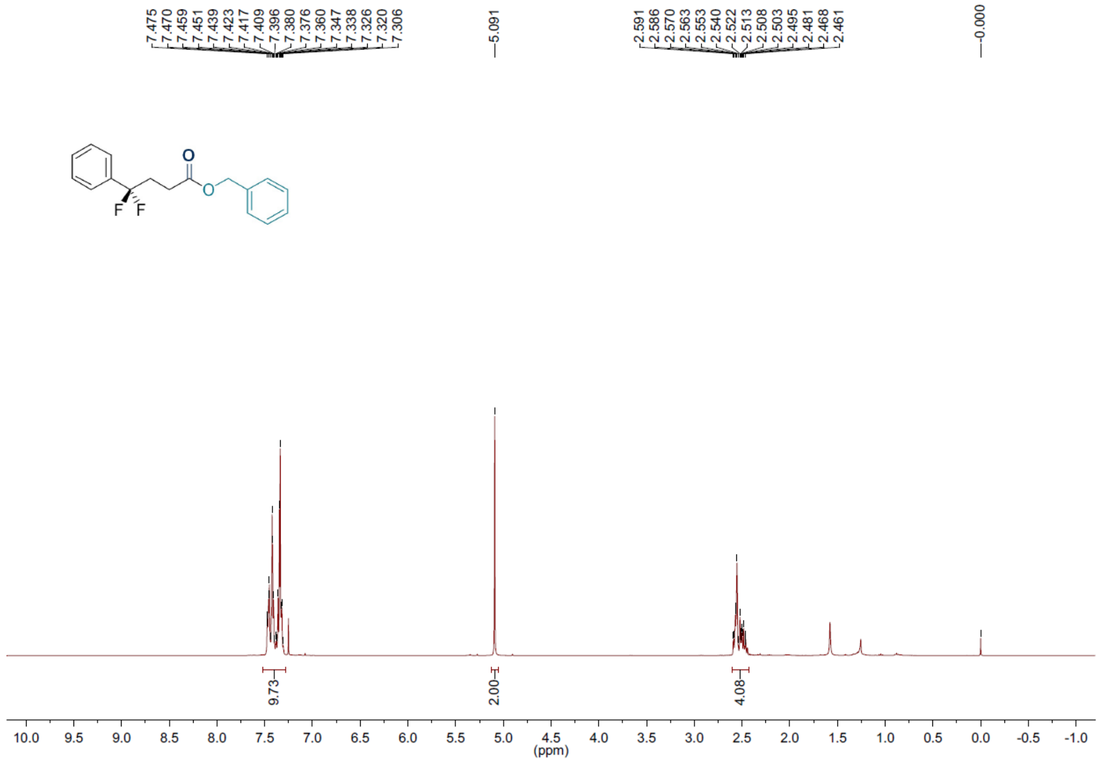

^{1}H NMR spectrum of **3a** in $CDCl_3$ (400 MHz)

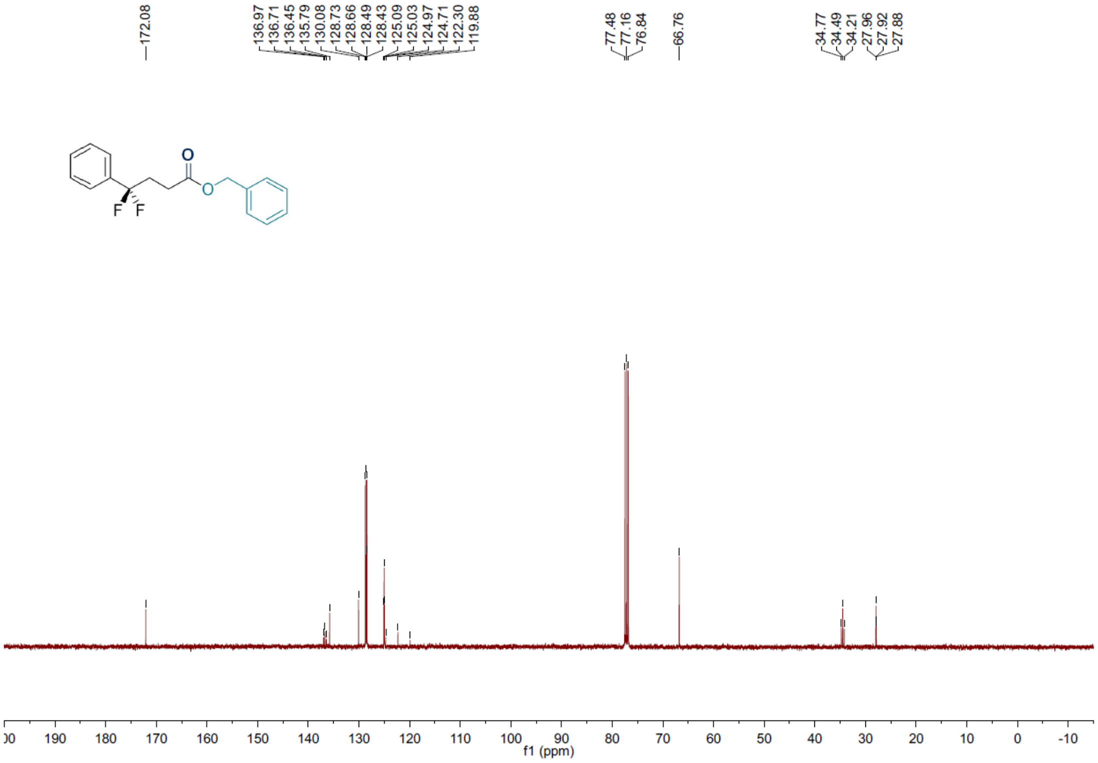

^{13}C NMR spectrum of **3a** in $CDCl_3$ (101 MHz)

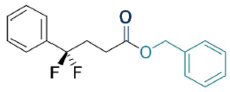

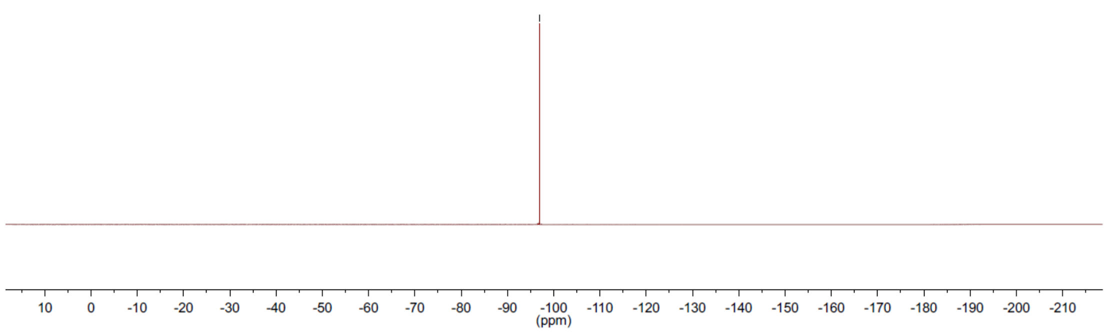

^{19}F NMR spectrum of **3a** in $CDCl_3$ (376 MHz)

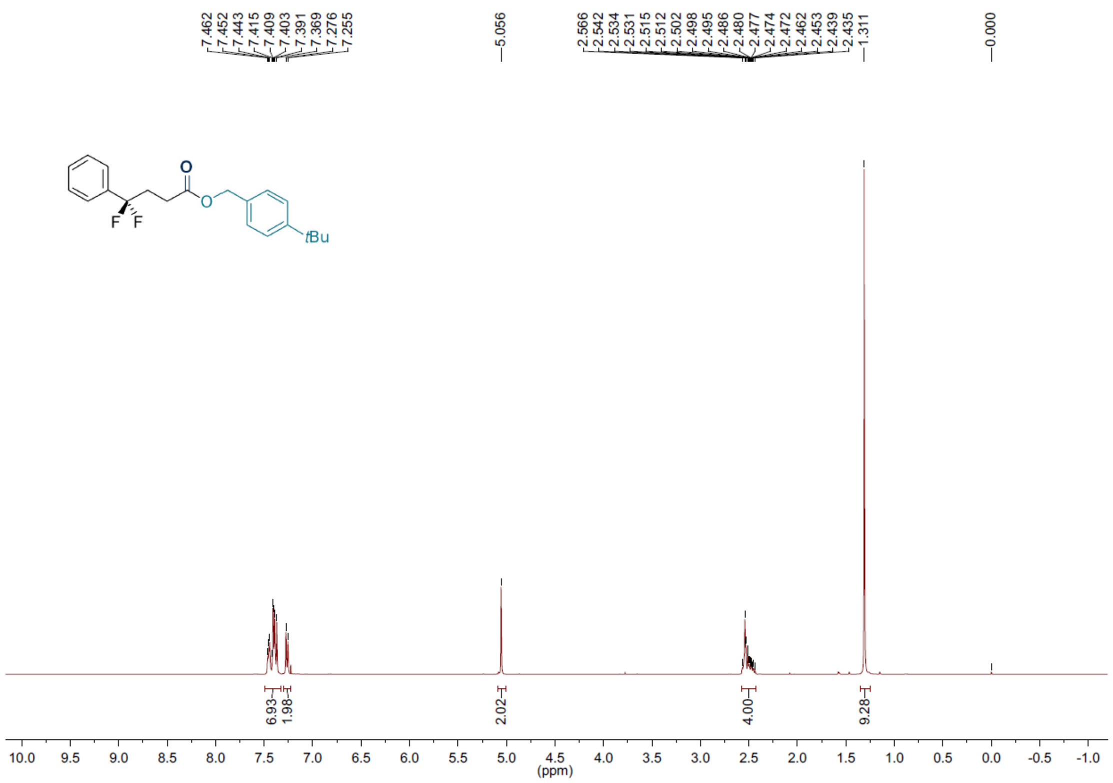

^1H NMR spectrum of **3b** in $CDCl_3$ (400 MHz)

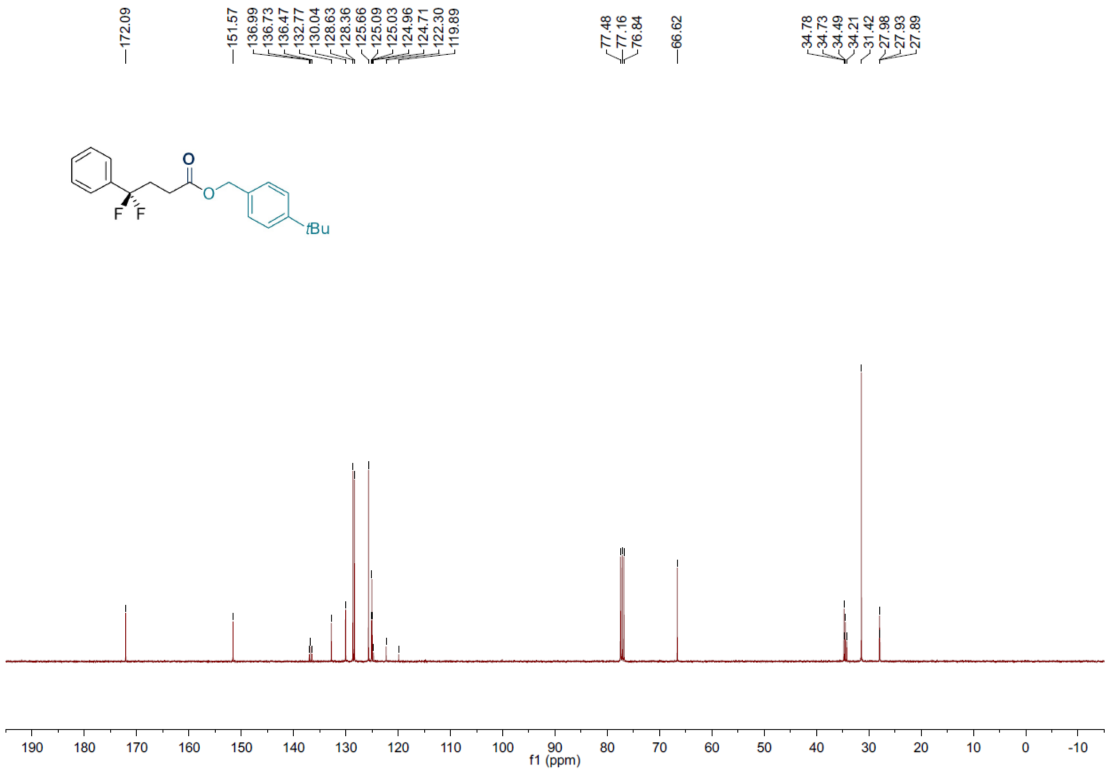

^{13}C NMR spectrum of **3b** in $CDCl_3$ (101 MHz)

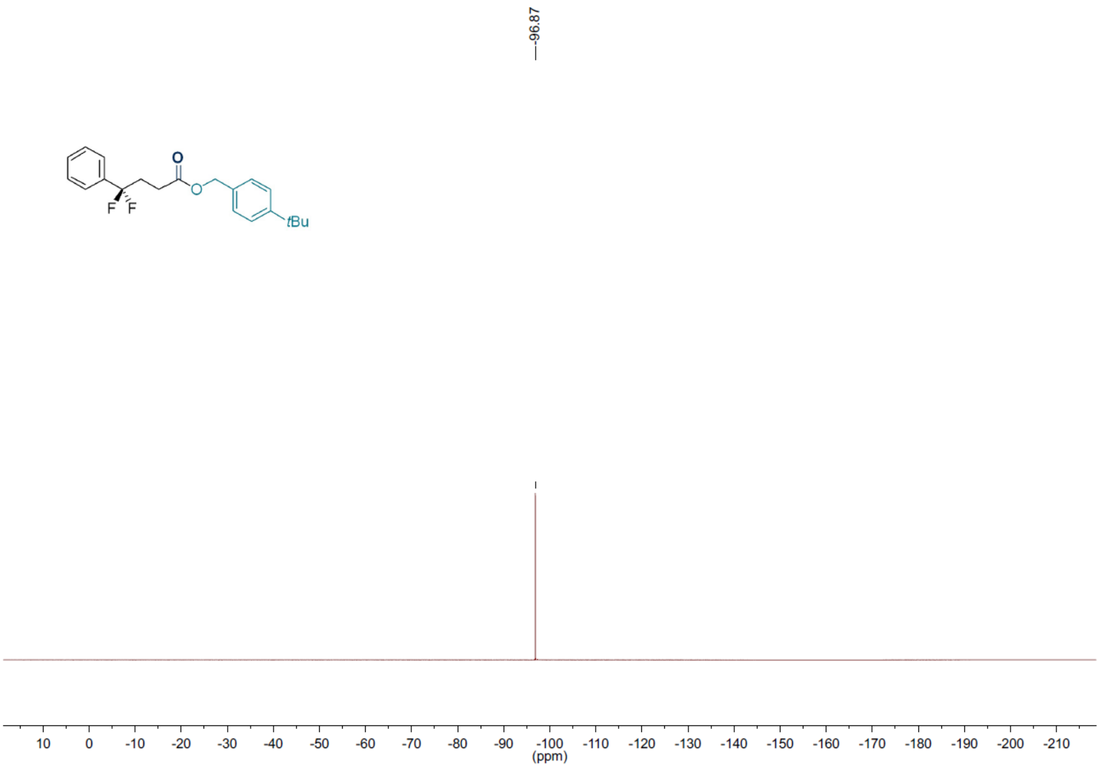

^{19}F NMR spectrum of **3b** in $CDCl_3$ (376 MHz)

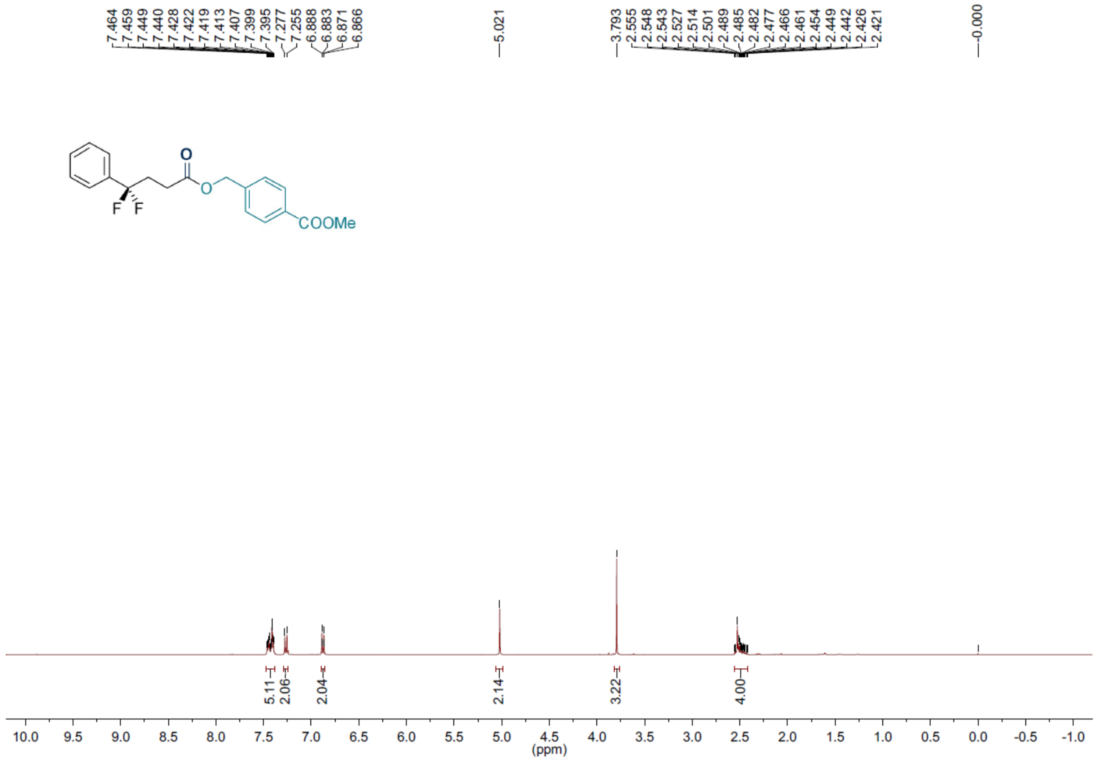

^{1}H NMR spectrum of **3c** in $CDCl_3$ (400 MHz)

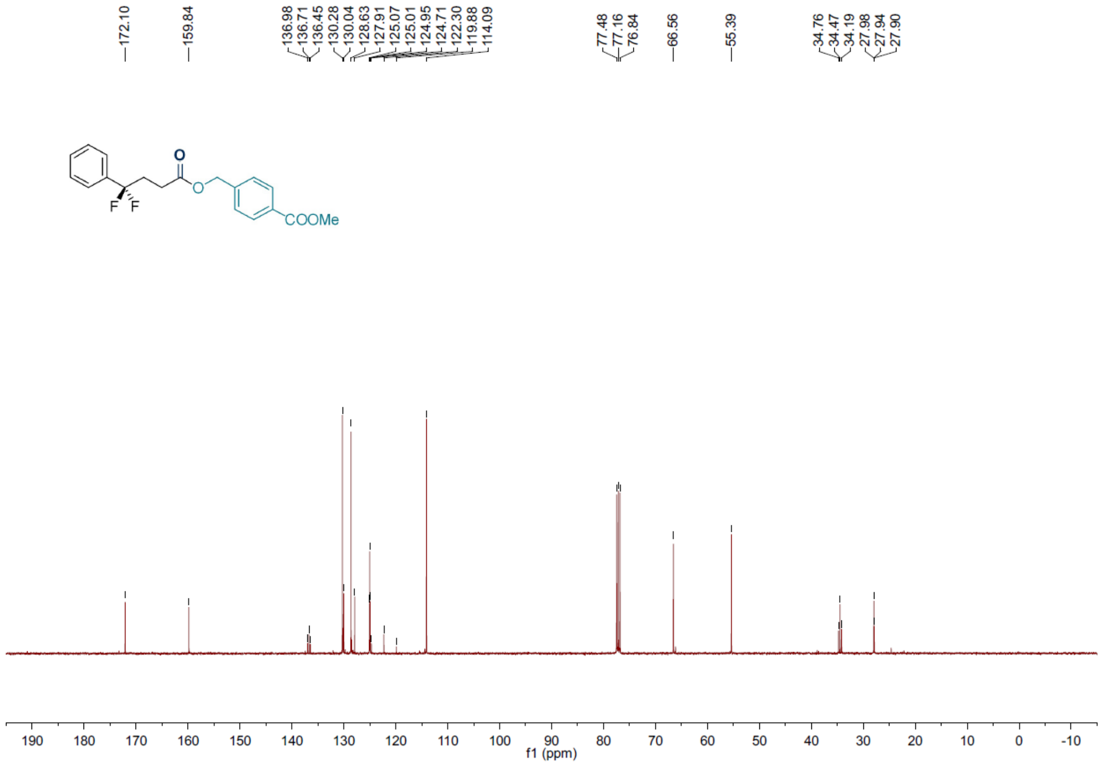

^{13}C NMR spectrum of **3c** in $CDCl_3$ (101 MHz)

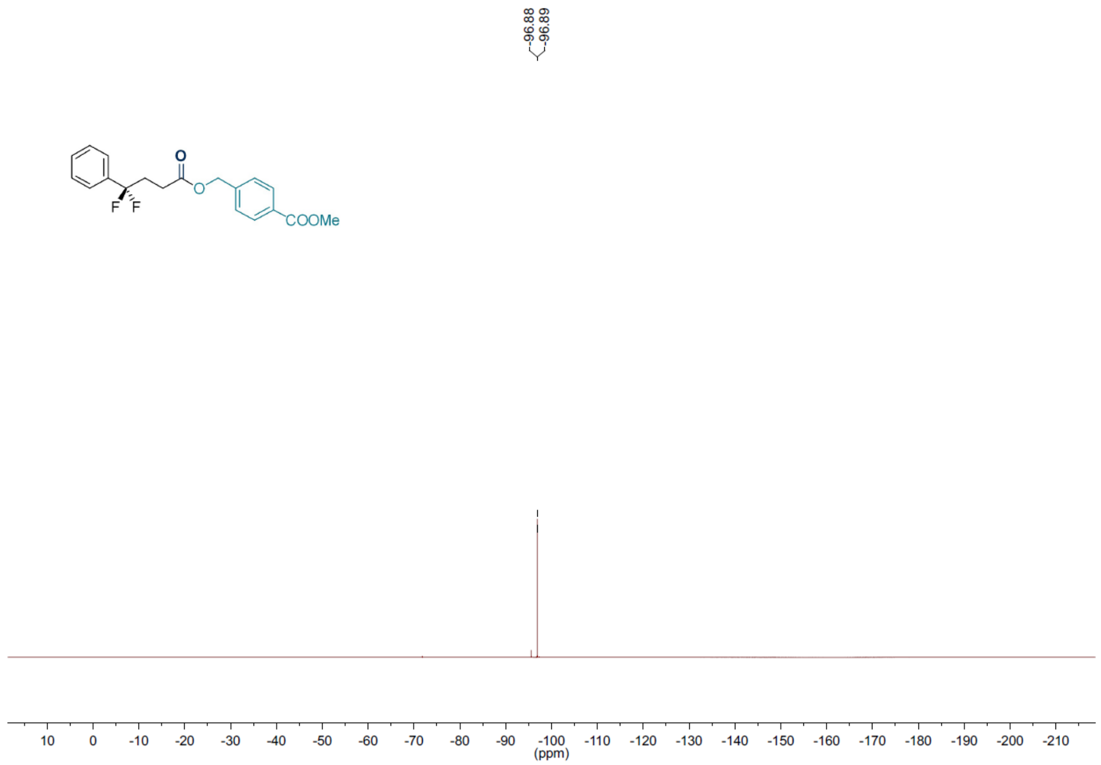

^{19}F NMR spectrum of **3c** in $CDCl_3$ (376 MHz)

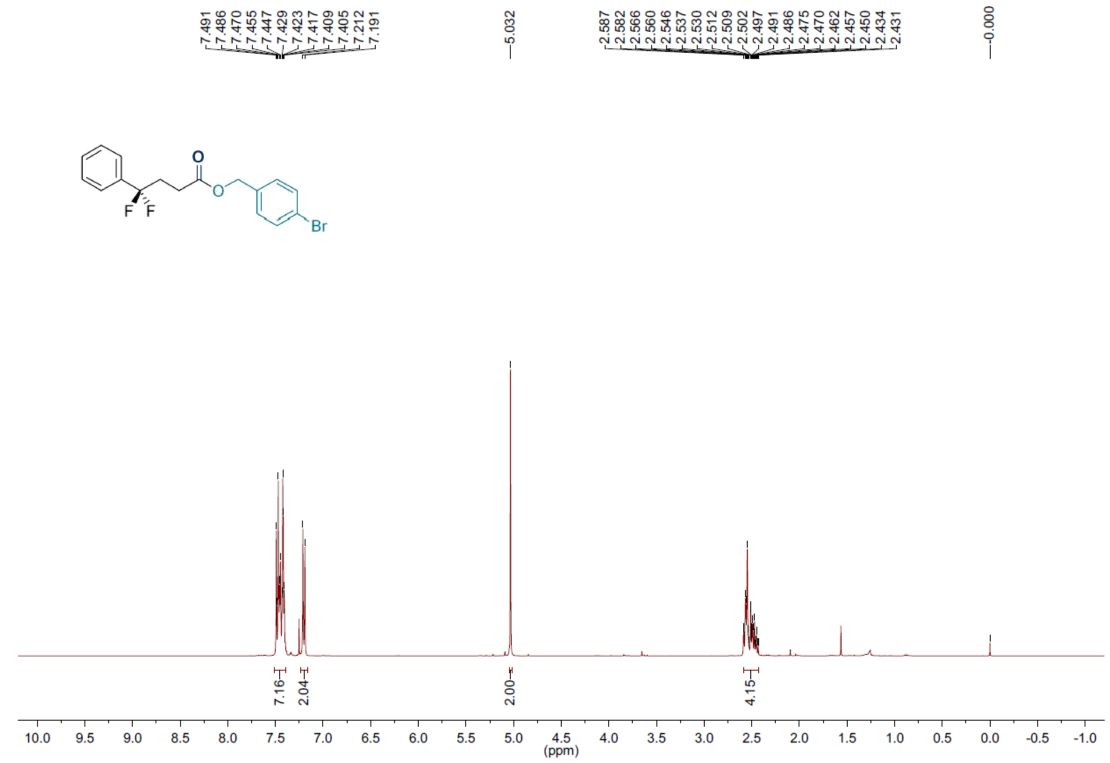

^{1}H NMR spectrum of **3d** in $CDCl_3$ (400 MHz)

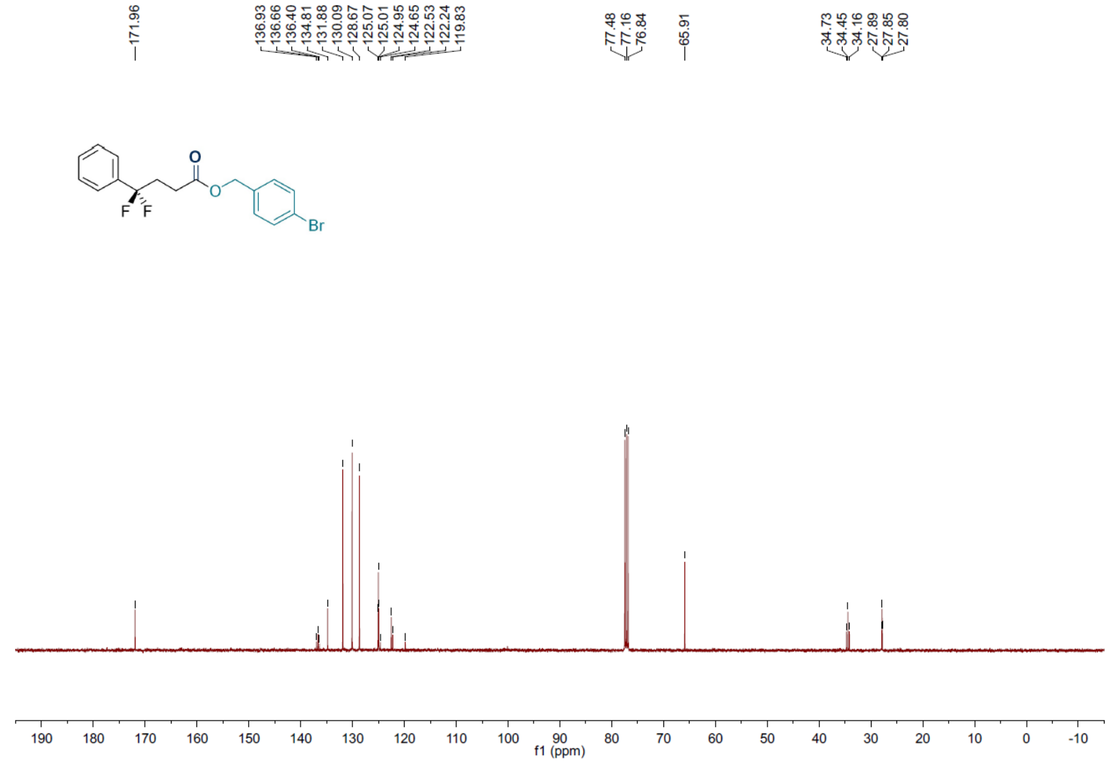

^{13}C NMR spectrum of **3d** in $CDCl_3$ (101 MHz)

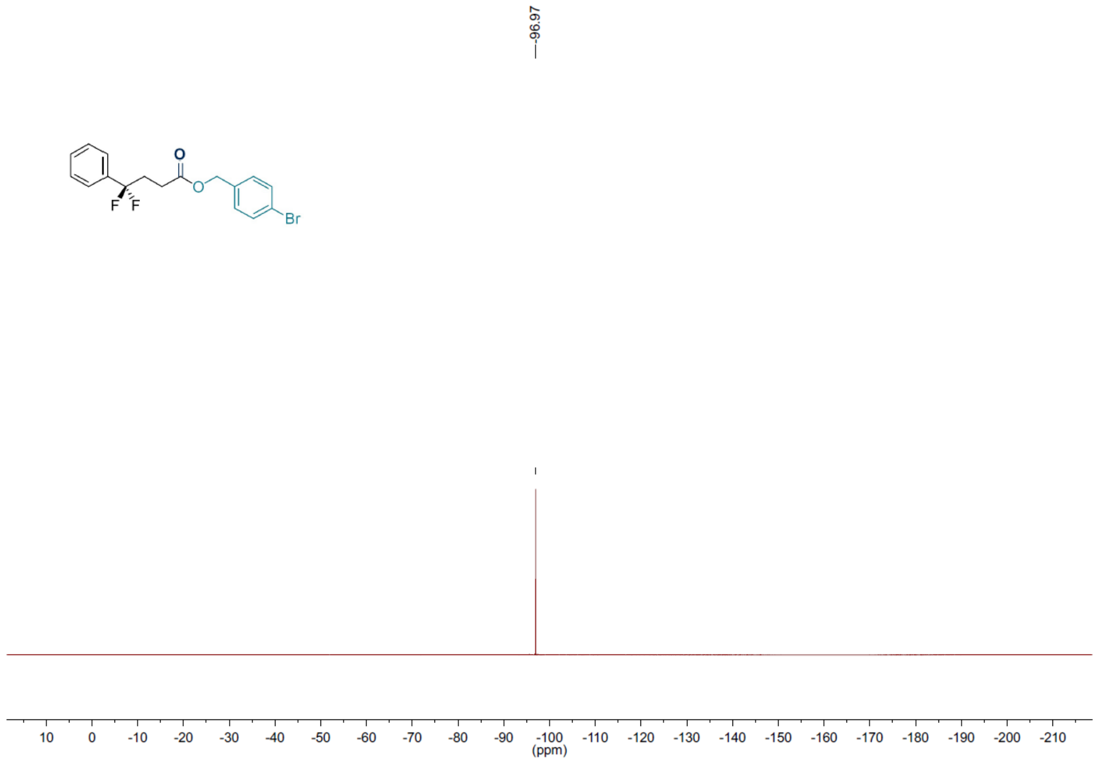

^{19}F NMR spectrum of **3d** in $CDCl_3$ (376 MHz)

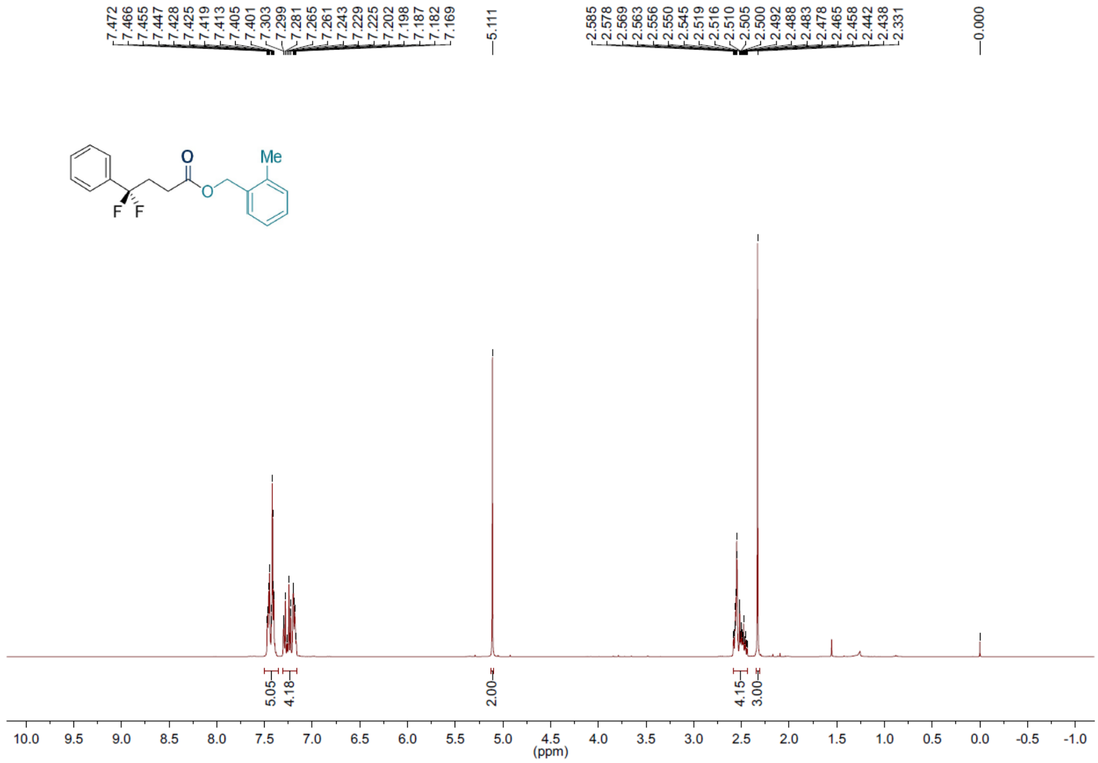

^{1}H NMR spectrum of **3e** in $CDCl_3$ (400 MHz)

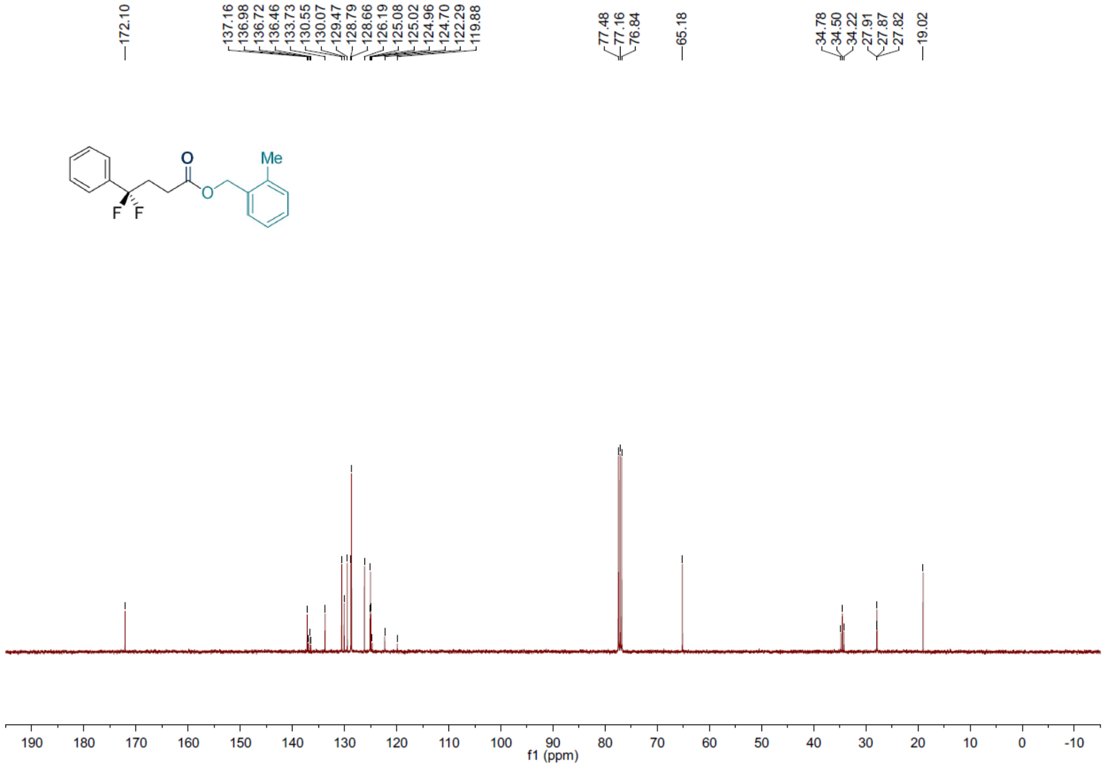

^{13}C NMR spectrum of **3e** in $CDCl_3$ (101 MHz)

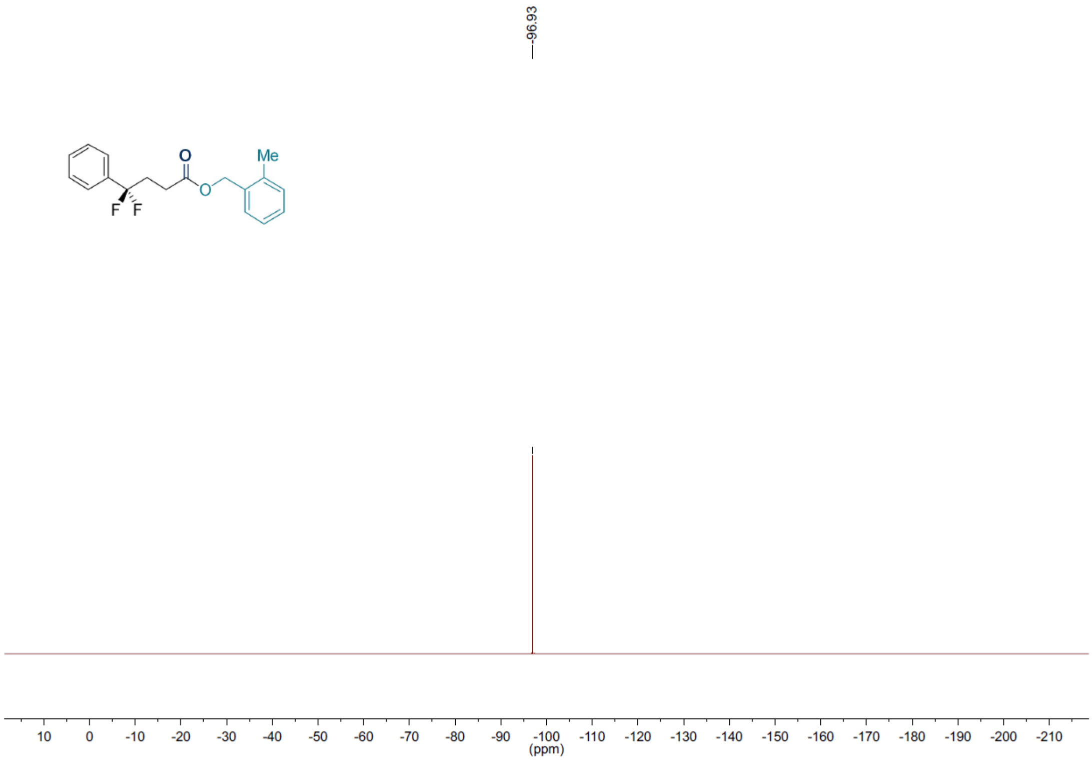

^{19}F NMR spectrum of **3e** in $CDCl_3$ (376 MHz)

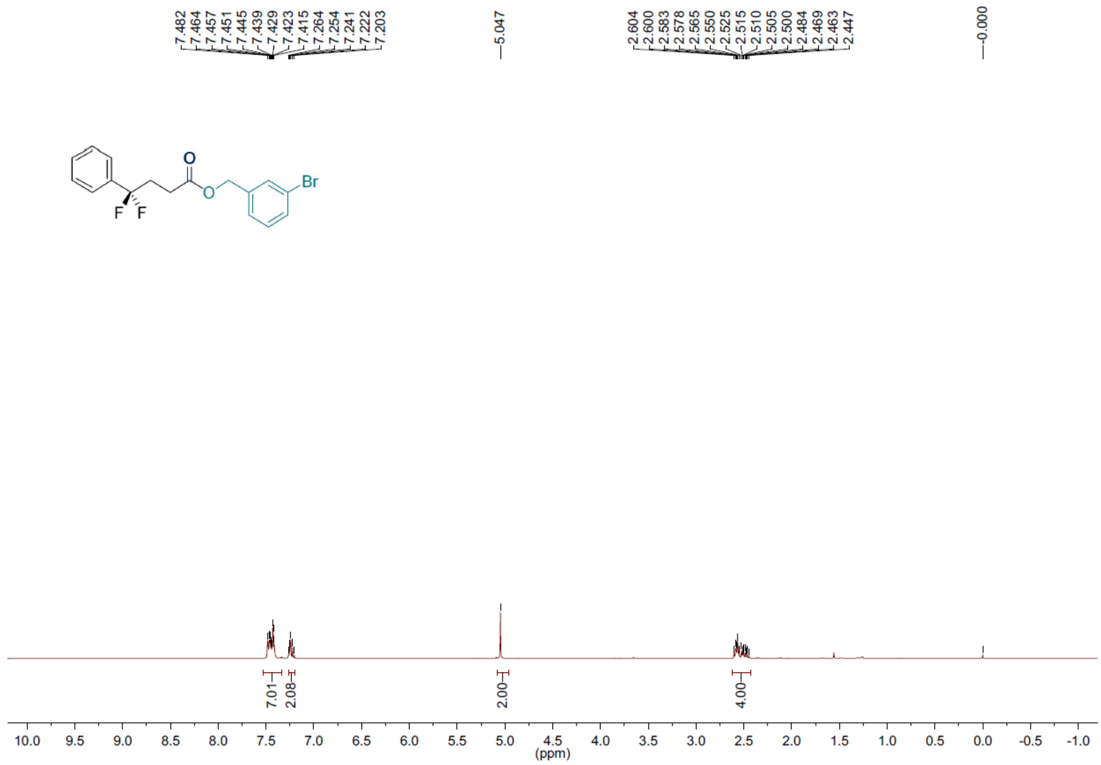

^{1}H NMR spectrum of **3f** in $CDCl_3$ (400 MHz)

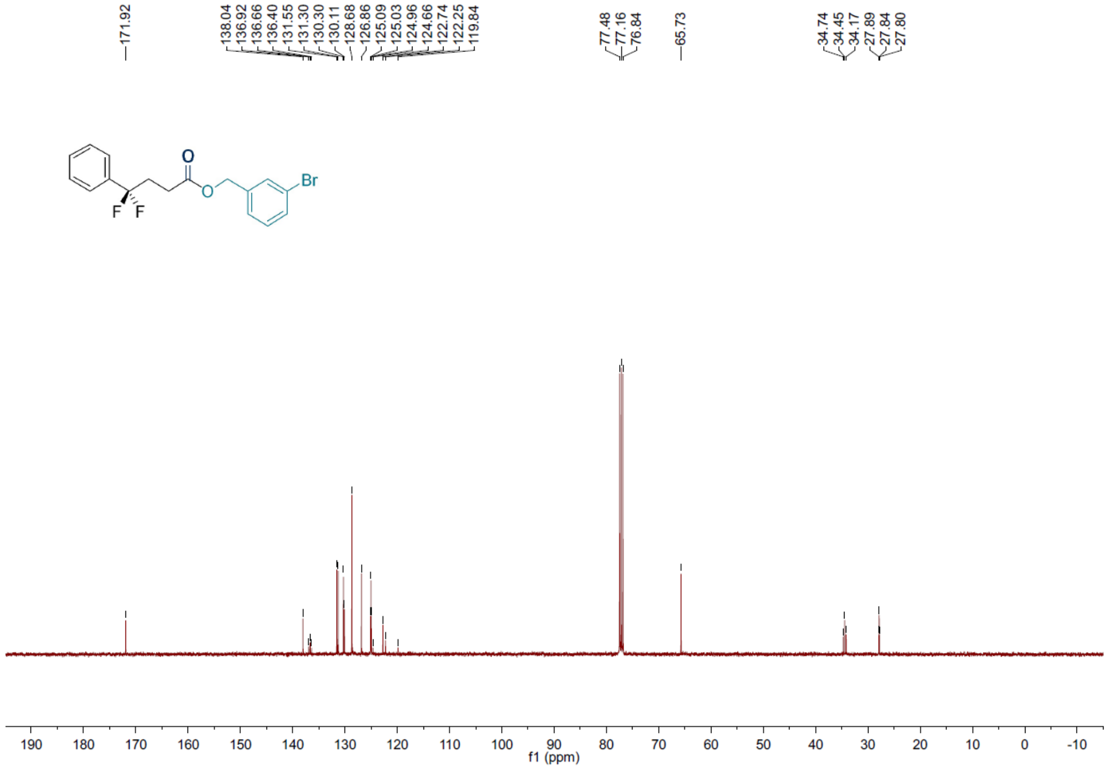

^{13}C NMR spectrum of **3f** in $CDCl_3$ (101 MHz)

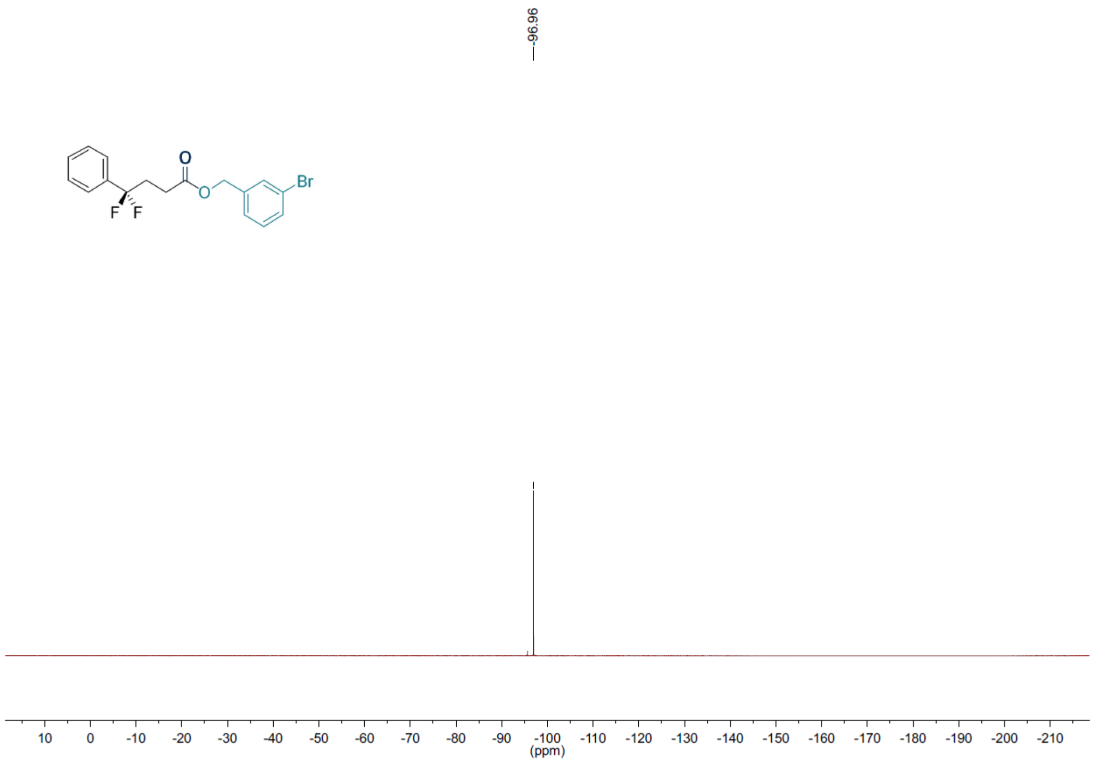

^{19}F NMR spectrum of **3f** in $CDCl_3$ (376 MHz)

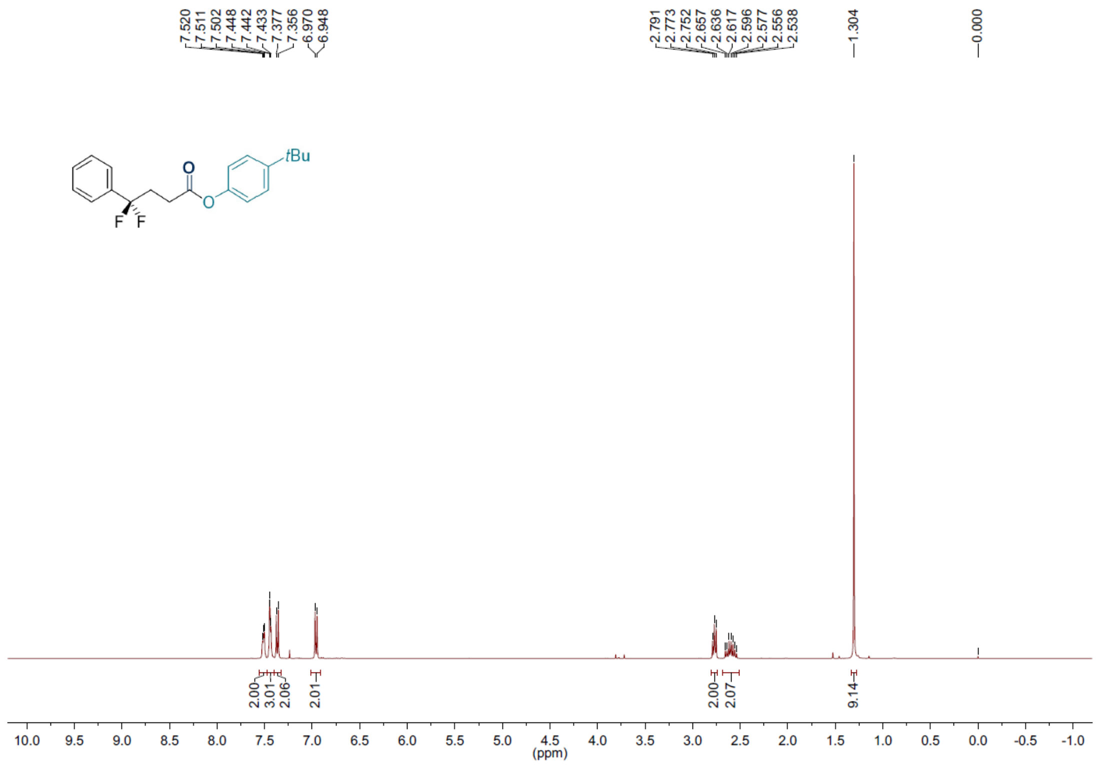

^{1}H NMR spectrum of **3g** in $CDCl_3$ (400 MHz)

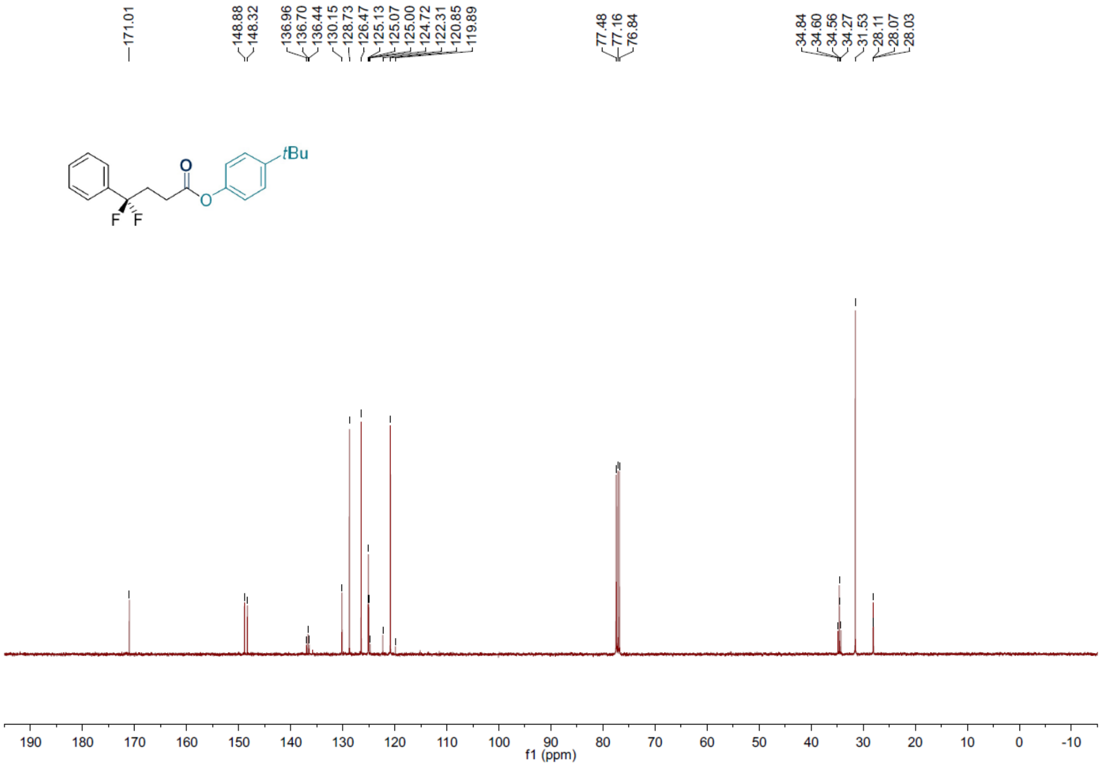

^{13}C NMR spectrum of **3g** in $CDCl_3$ (101 MHz)

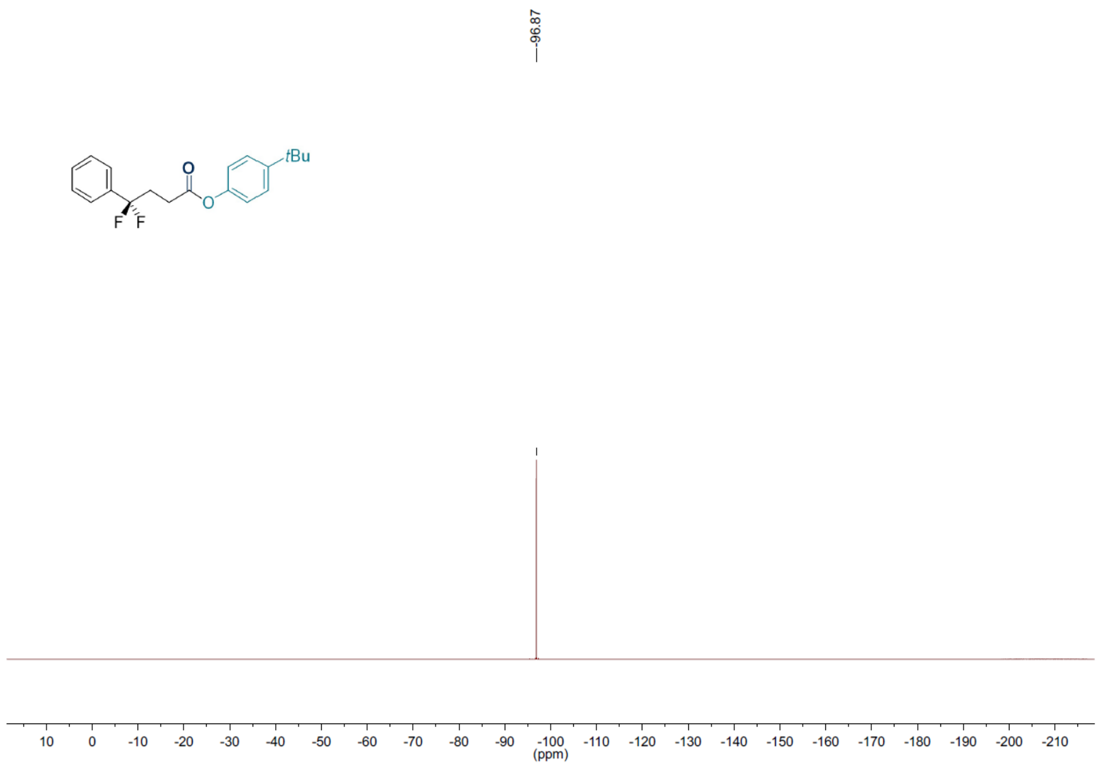

^{19}F NMR spectrum of **3g** in $CDCl_3$ (376 MHz)

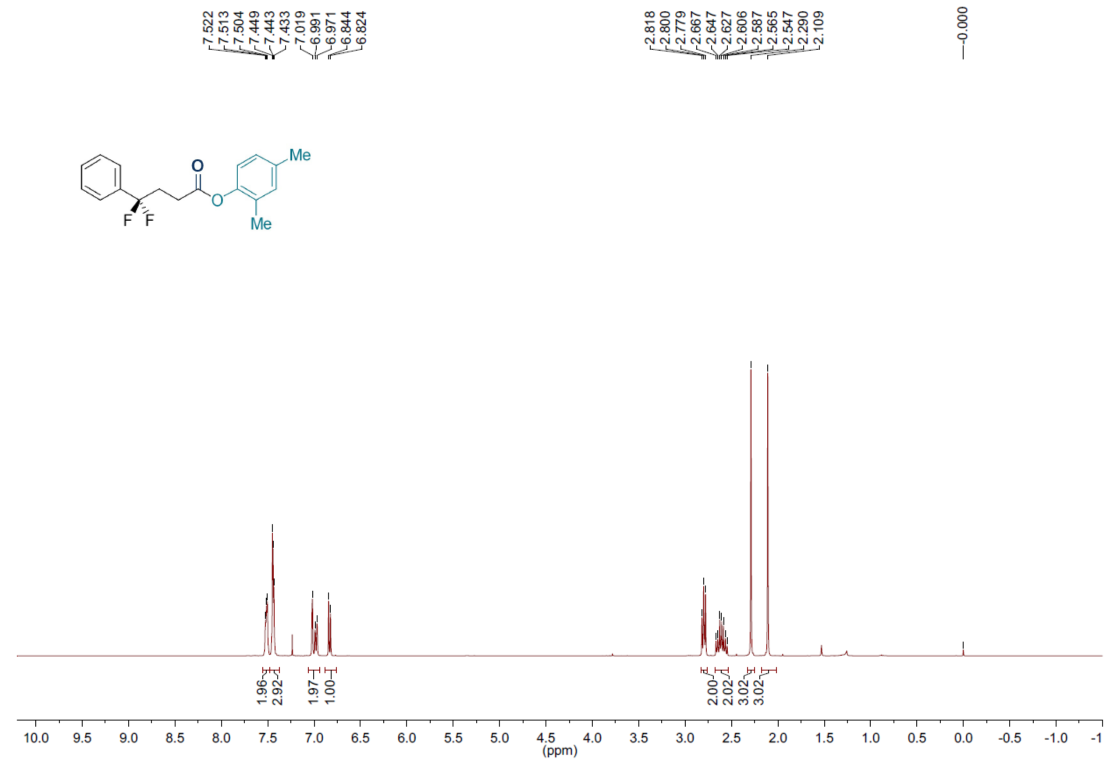

^{1}H NMR spectrum of **3h** in $CDCl_3$ (400 MHz)

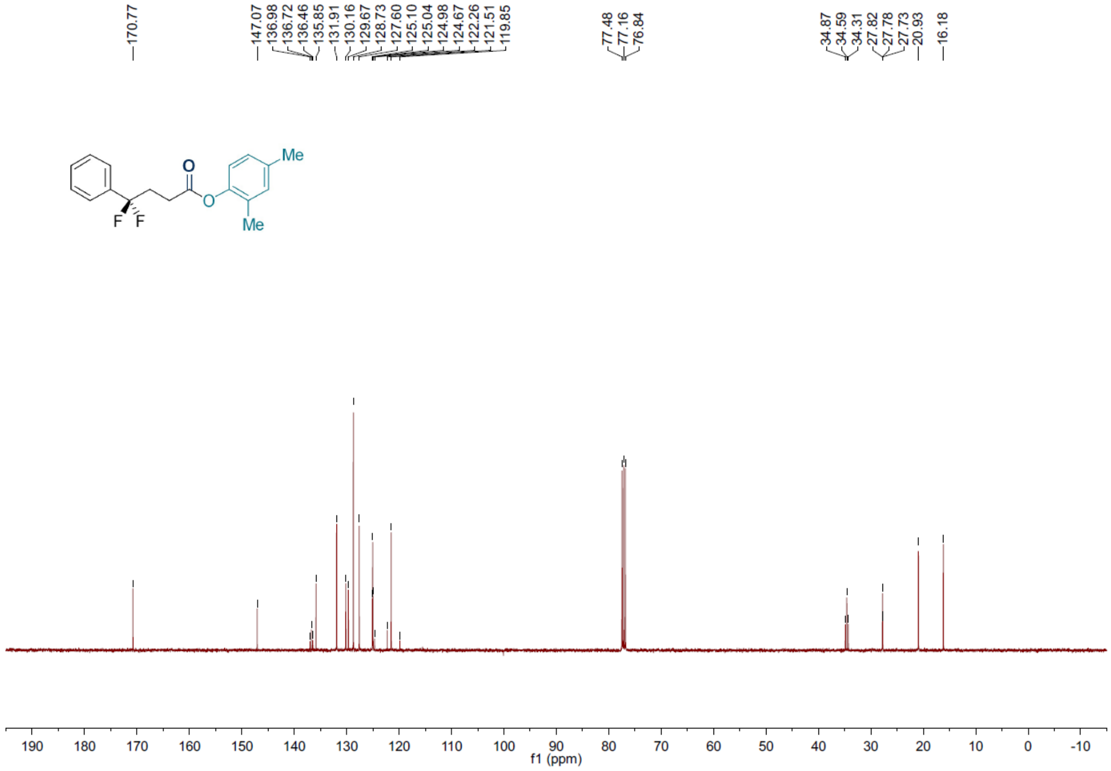

^{13}C NMR spectrum of **3h** in $CDCl_3$ (101 MHz)

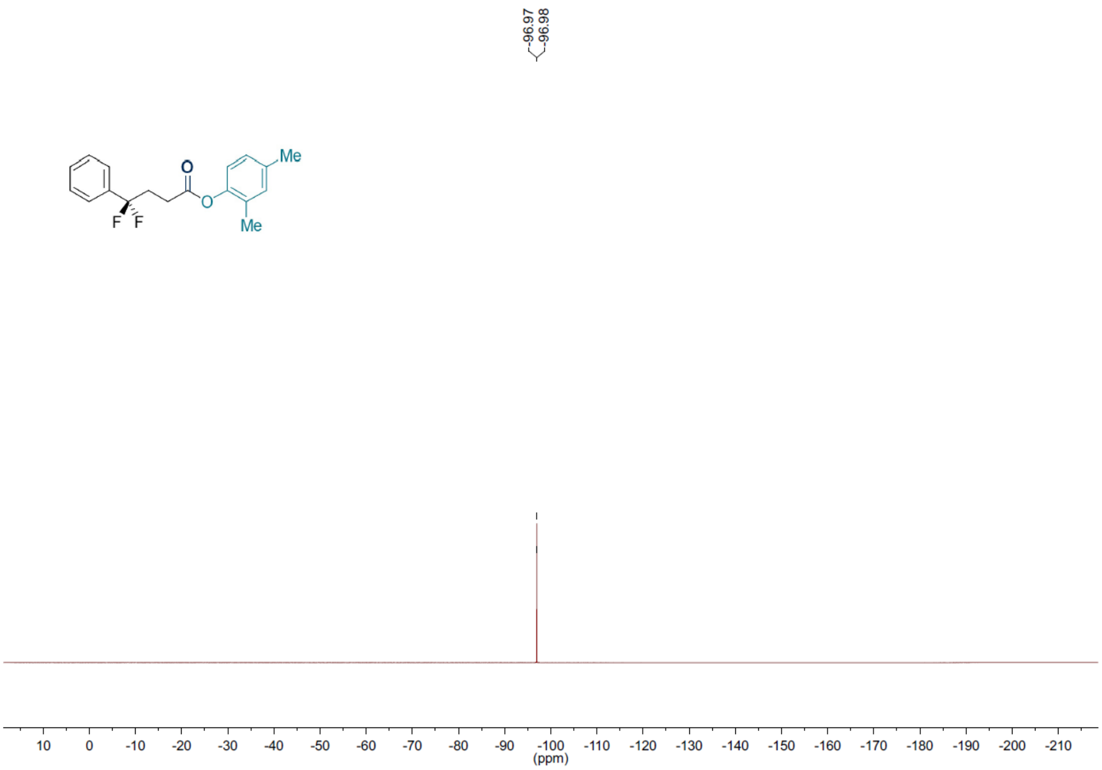

^{19}F NMR spectrum of **3h** in $CDCl_3$ (376 MHz)

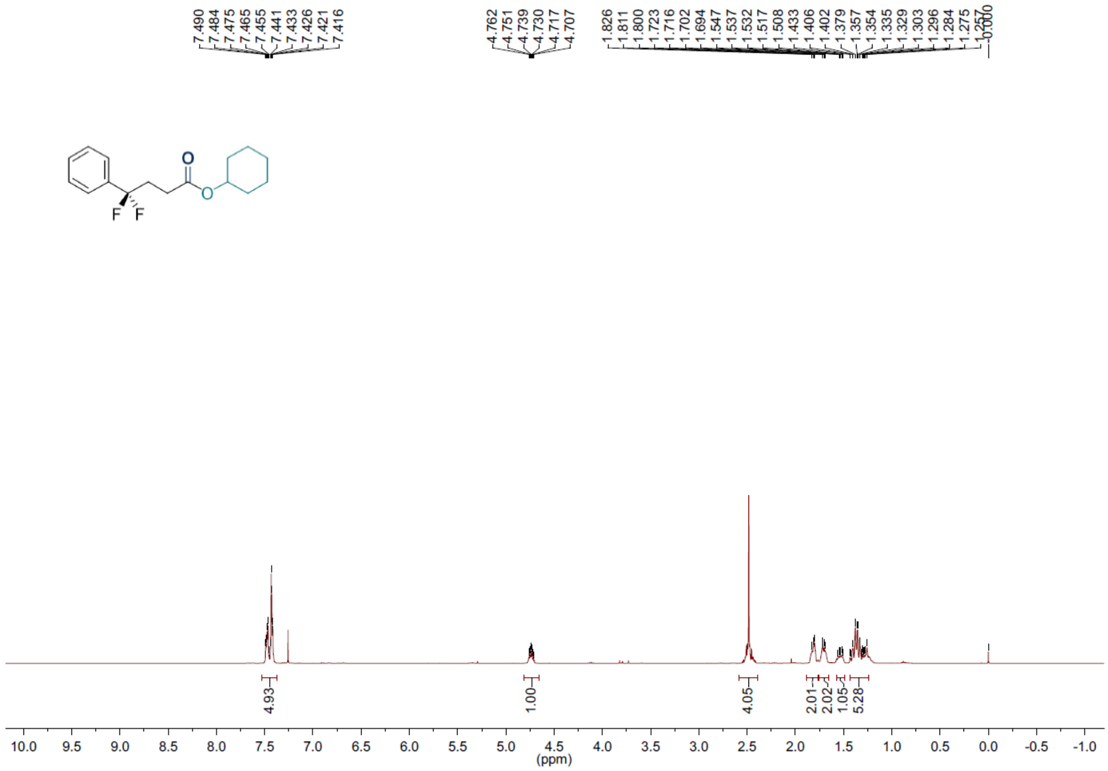

^{1}H NMR spectrum of **3i** in $CDCl_3$ (400 MHz)

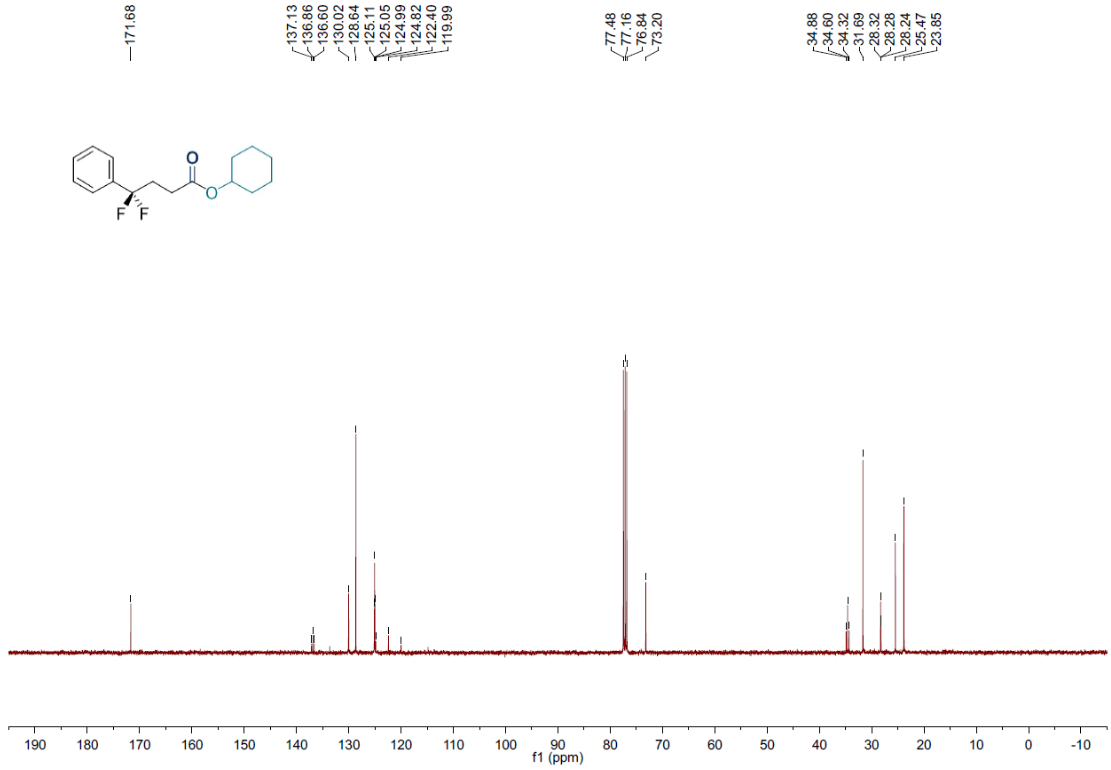

^{13}C NMR spectrum of **3i** in $CDCl_3$ (101 MHz)

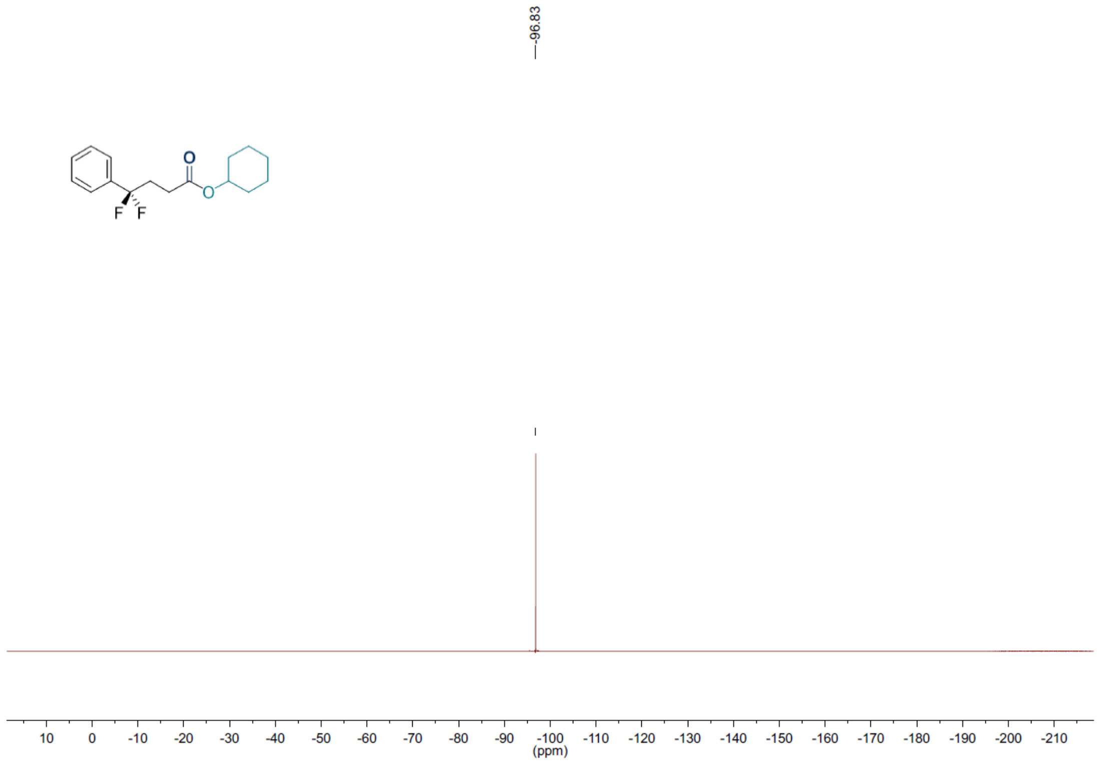

^{19}F NMR spectrum of **3i** in $CDCl_3$ (376 MHz)

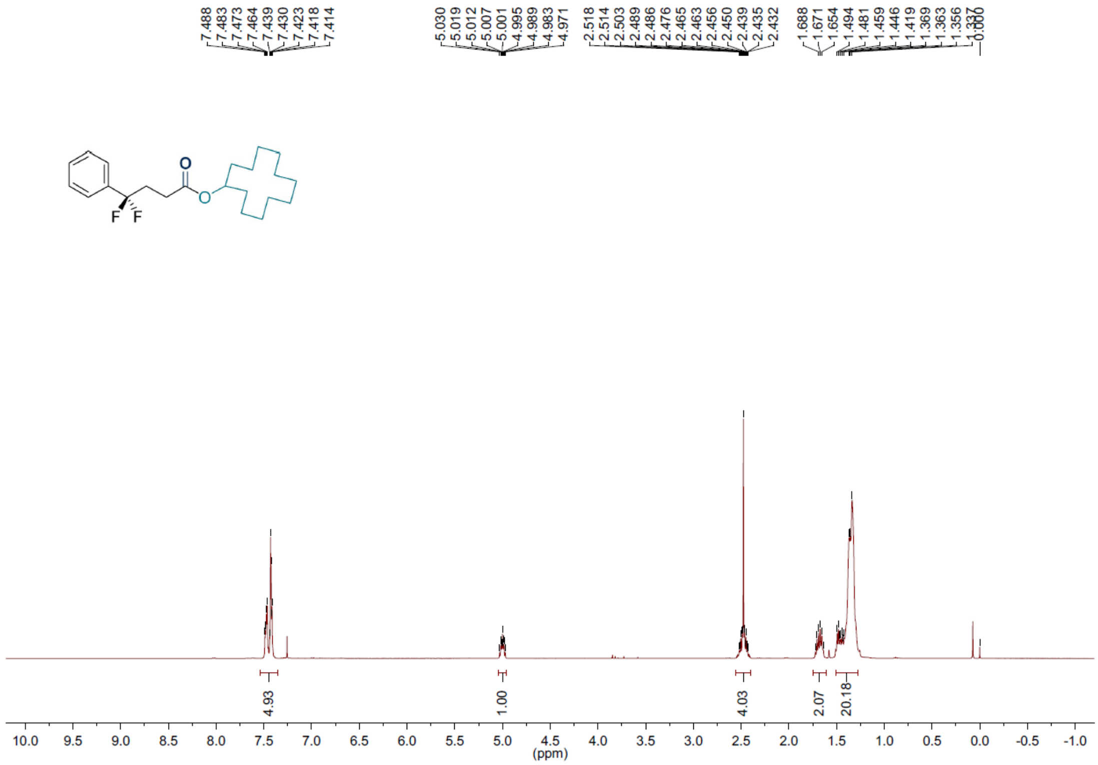

^{1}H NMR spectrum of **3j** in $CDCl_3$ (400 MHz)

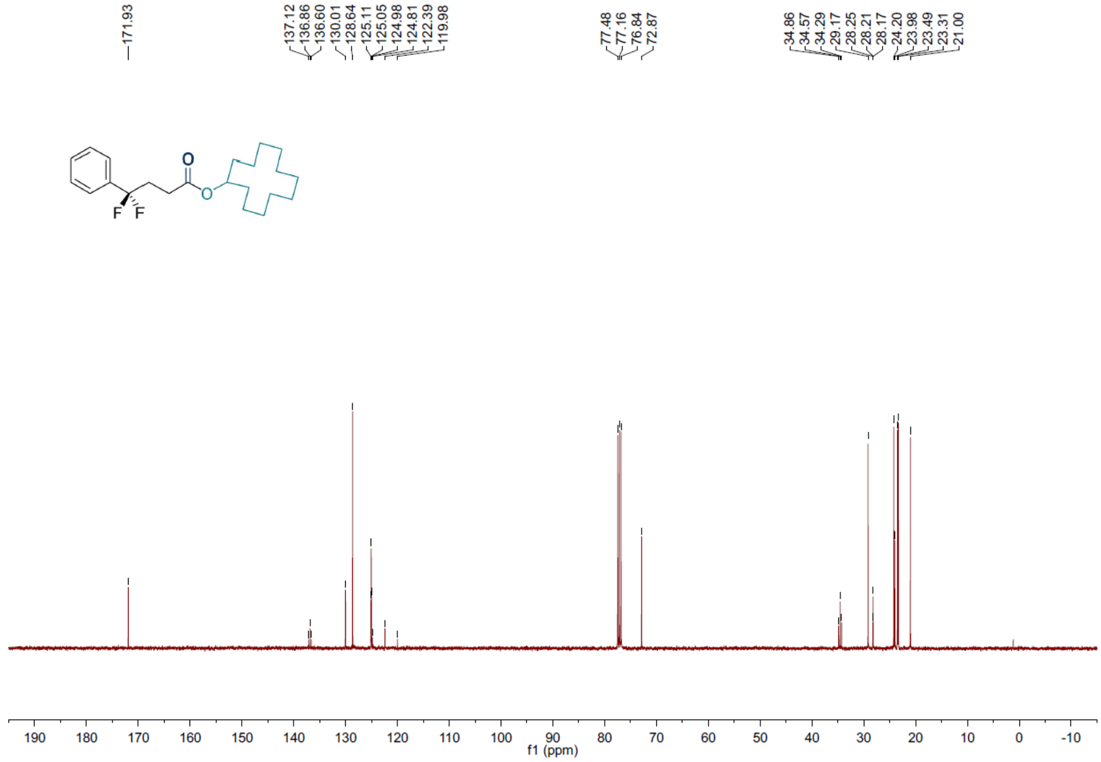

^{13}C NMR spectrum of **3j** in $CDCl_3$ (101 MHz)

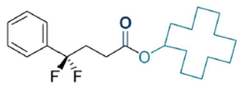

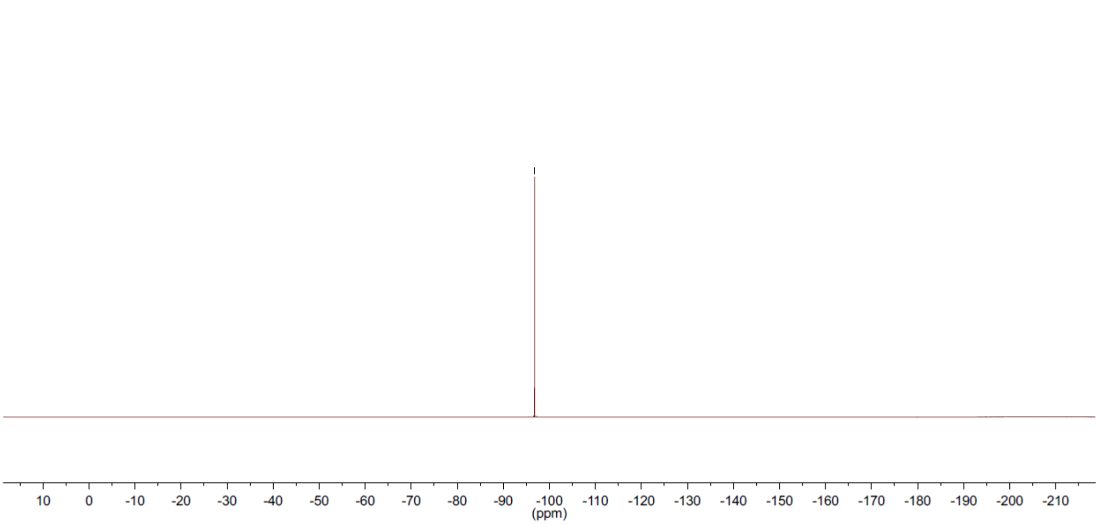

^{19}F NMR spectrum of **3j** in $CDCl_3$ (376 MHz)

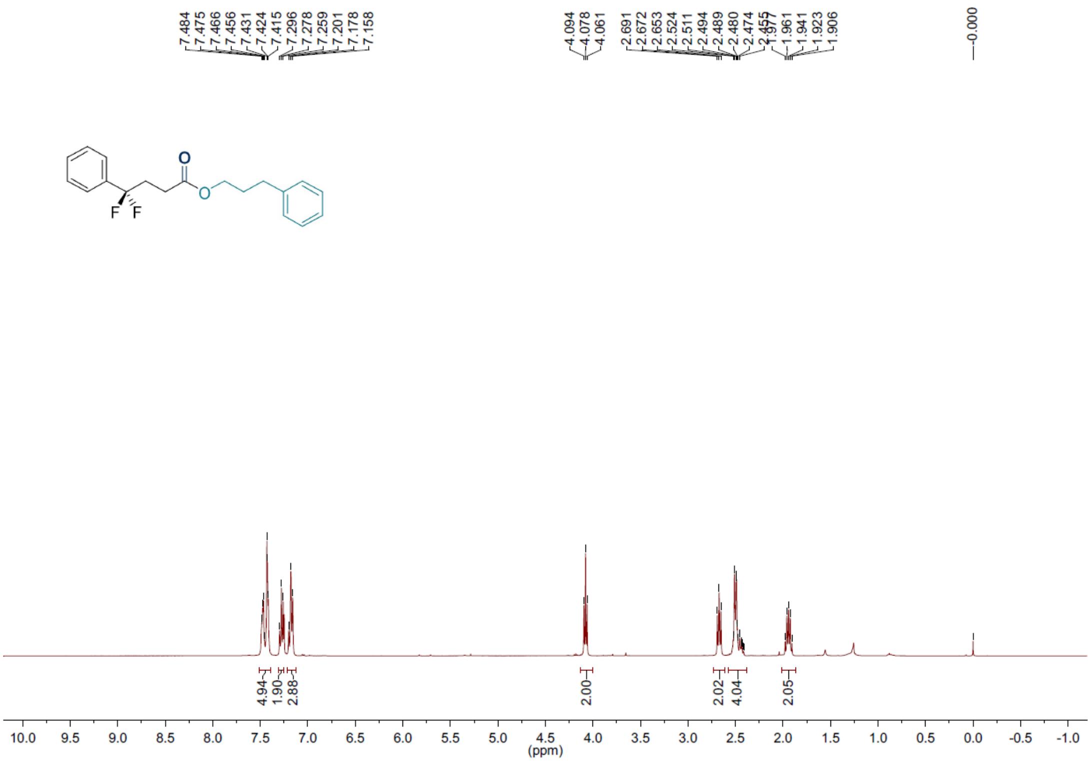

^{1}H NMR spectrum of **3k** in $CDCl_3$ (400 MHz)

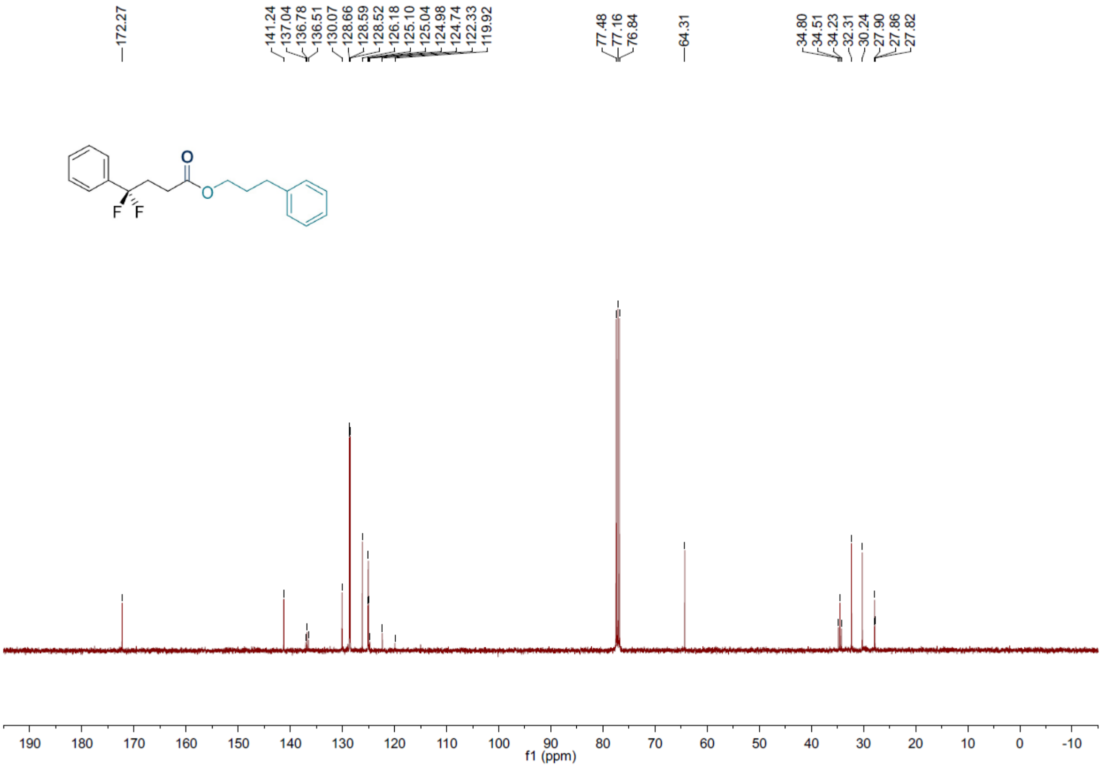

^{13}C NMR spectrum of **3k** in $CDCl_3$ (101 MHz)

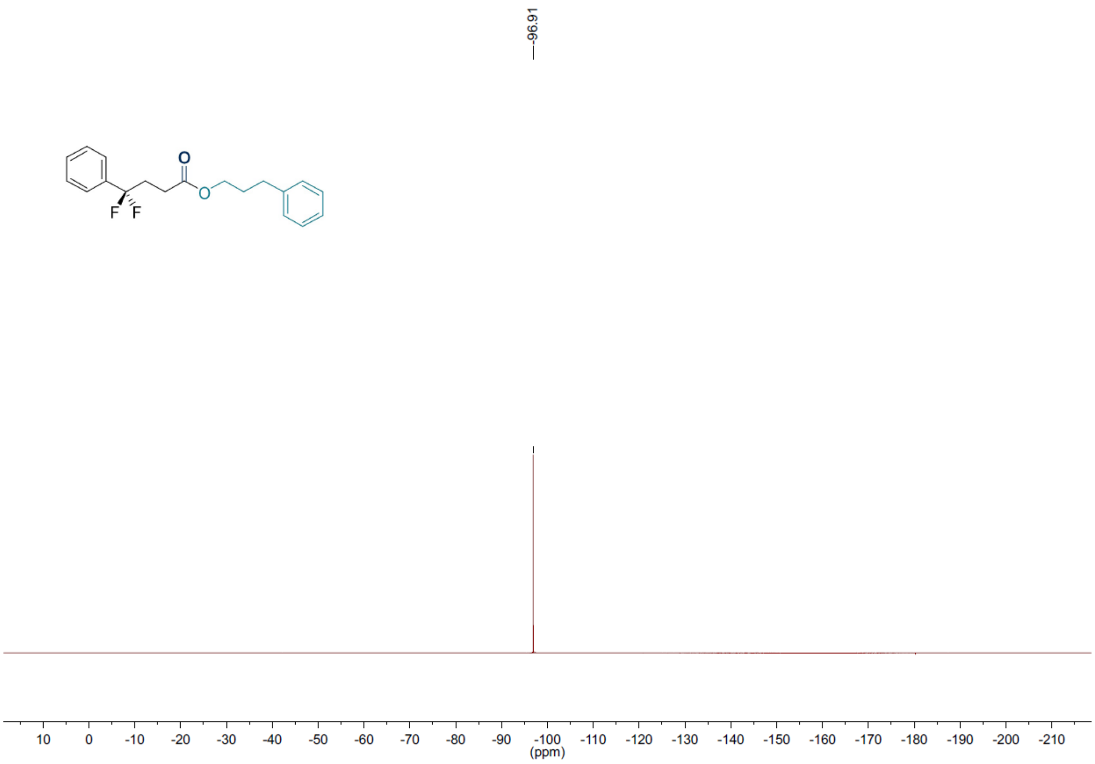

^{19}F NMR spectrum of **3k** in $CDCl_3$ (376 MHz)

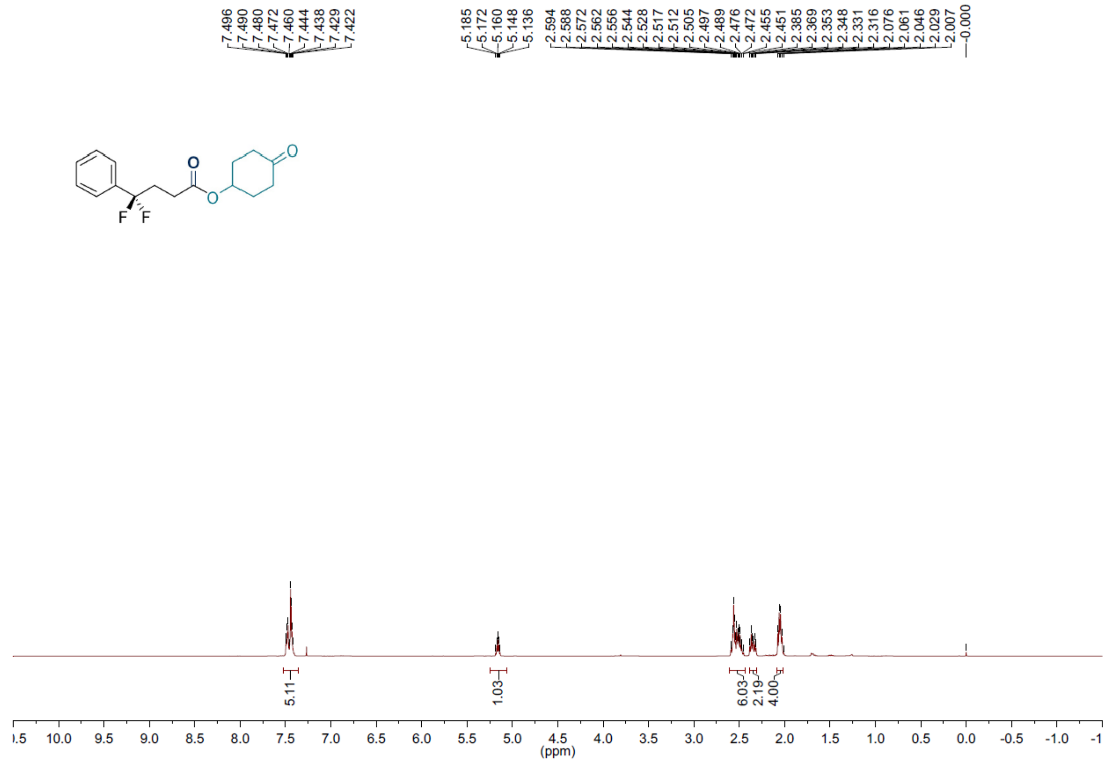

^{1}H NMR spectrum of **3l** in $CDCl_3$ (400 MHz)

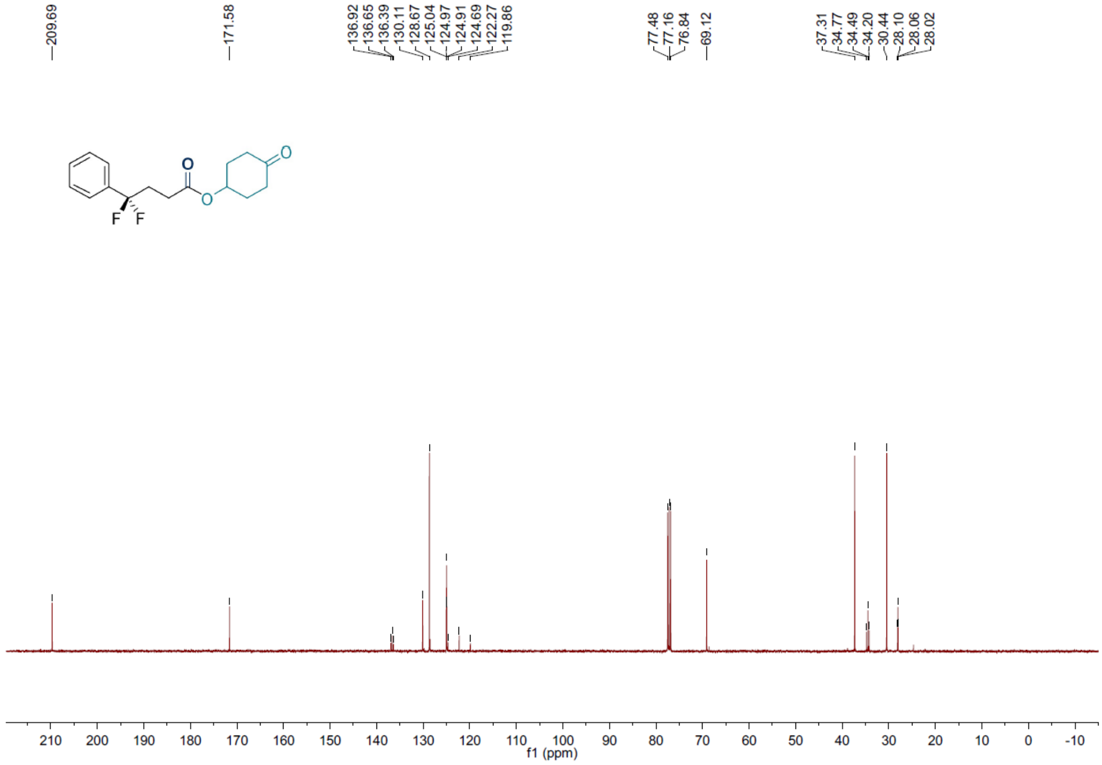

^{13}C NMR spectrum of **3l** in $CDCl_3$ (101 MHz)

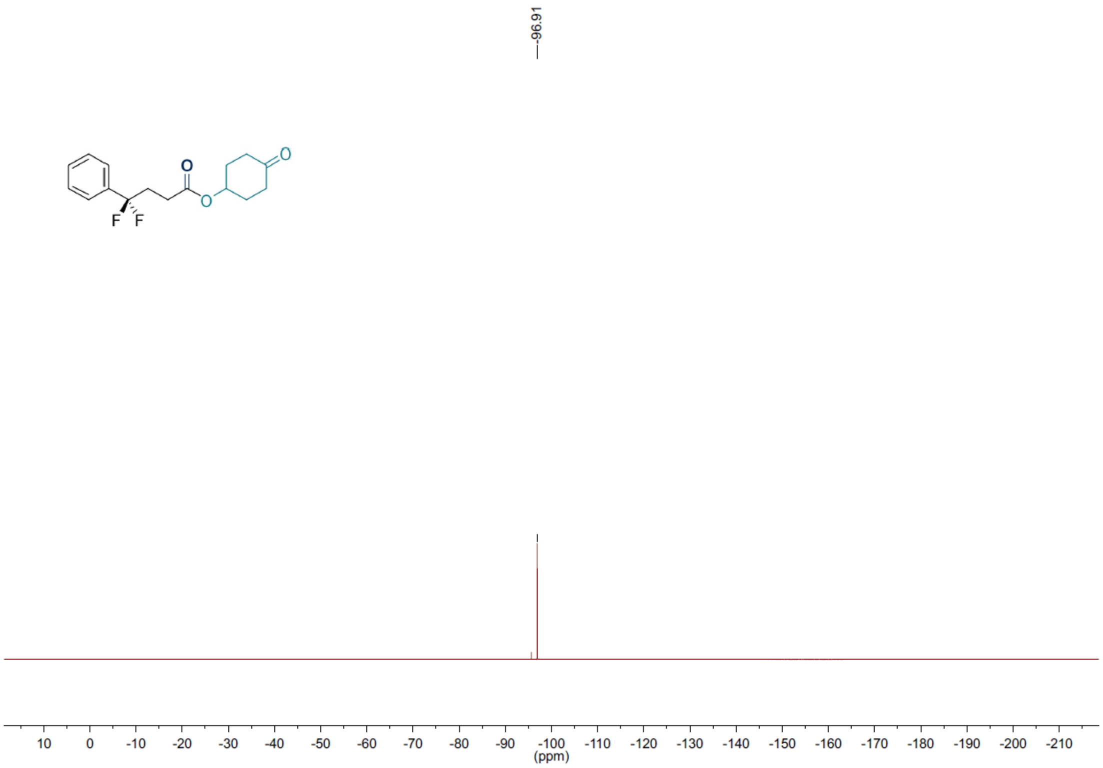

^{19}F NMR spectrum of **3l** in $CDCl_3$ (376 MHz)

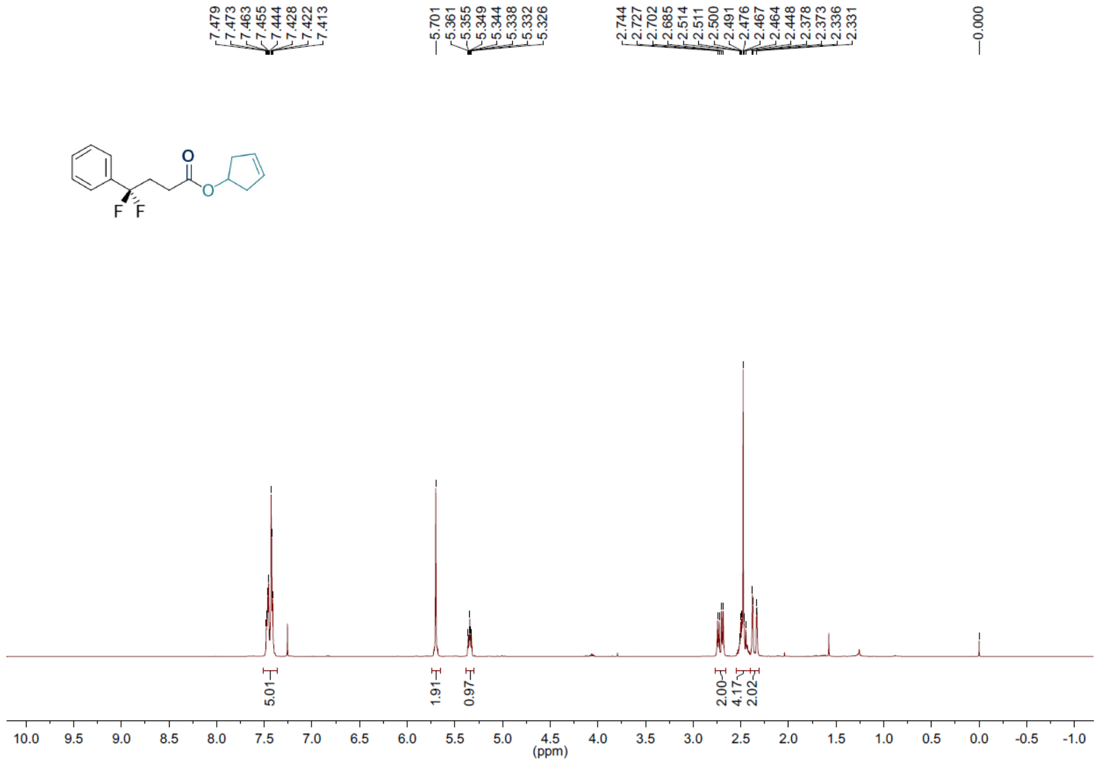

^{1}H NMR spectrum of **3m** in $CDCl_3$ (400 MHz)

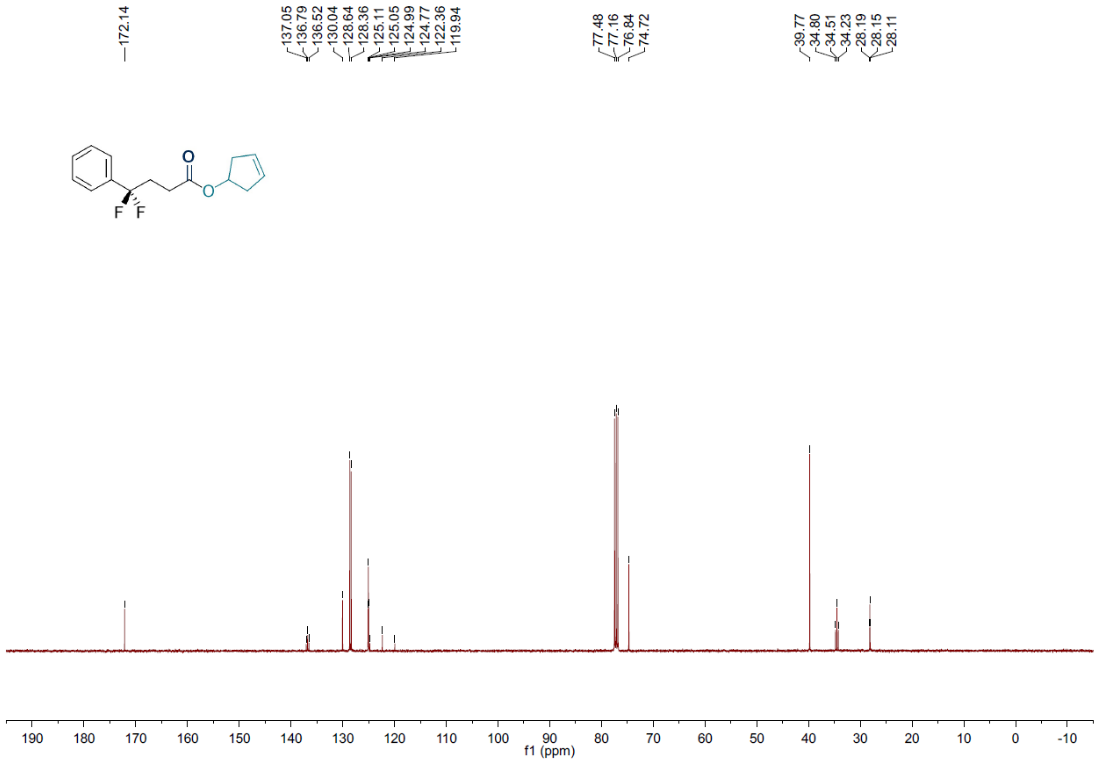

^{13}C NMR spectrum of **3m** in $CDCl_3$ (101 MHz)

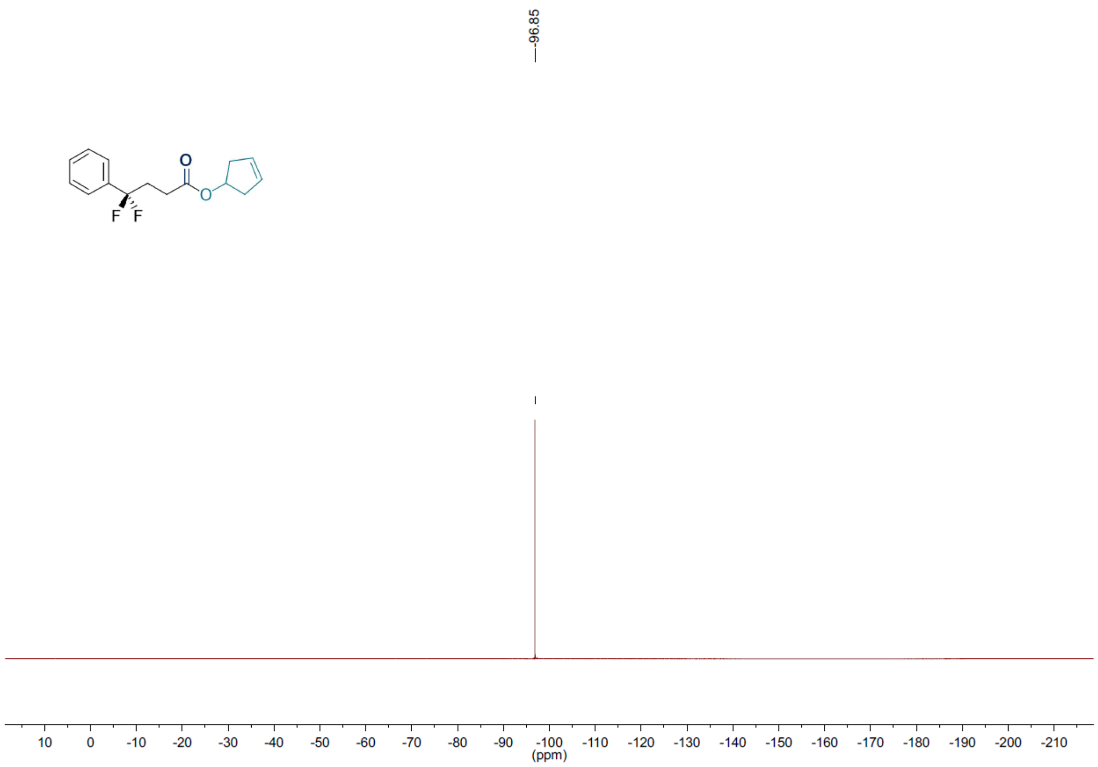

^{19}F NMR spectrum of **3m** in $CDCl_3$ (376 MHz)

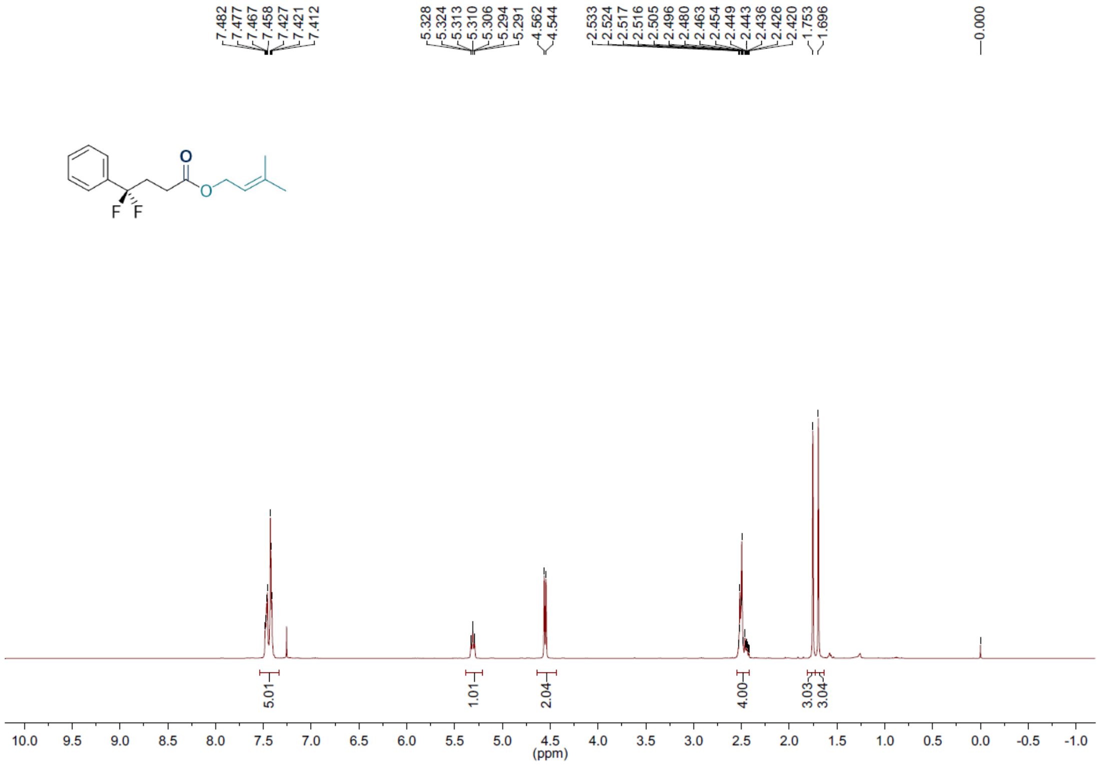

^{1}H NMR spectrum of **3n** in $CDCl_3$ (400 MHz)

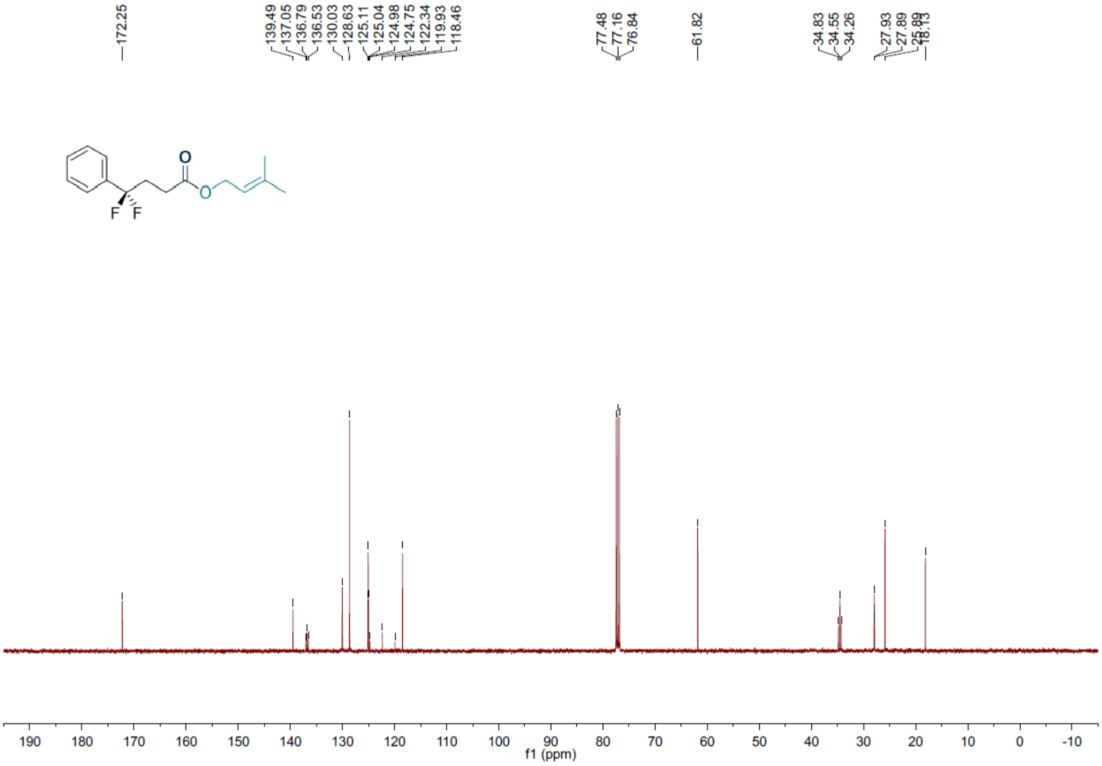

^{13}C NMR spectrum of **3n** in $CDCl_3$ (101 MHz)

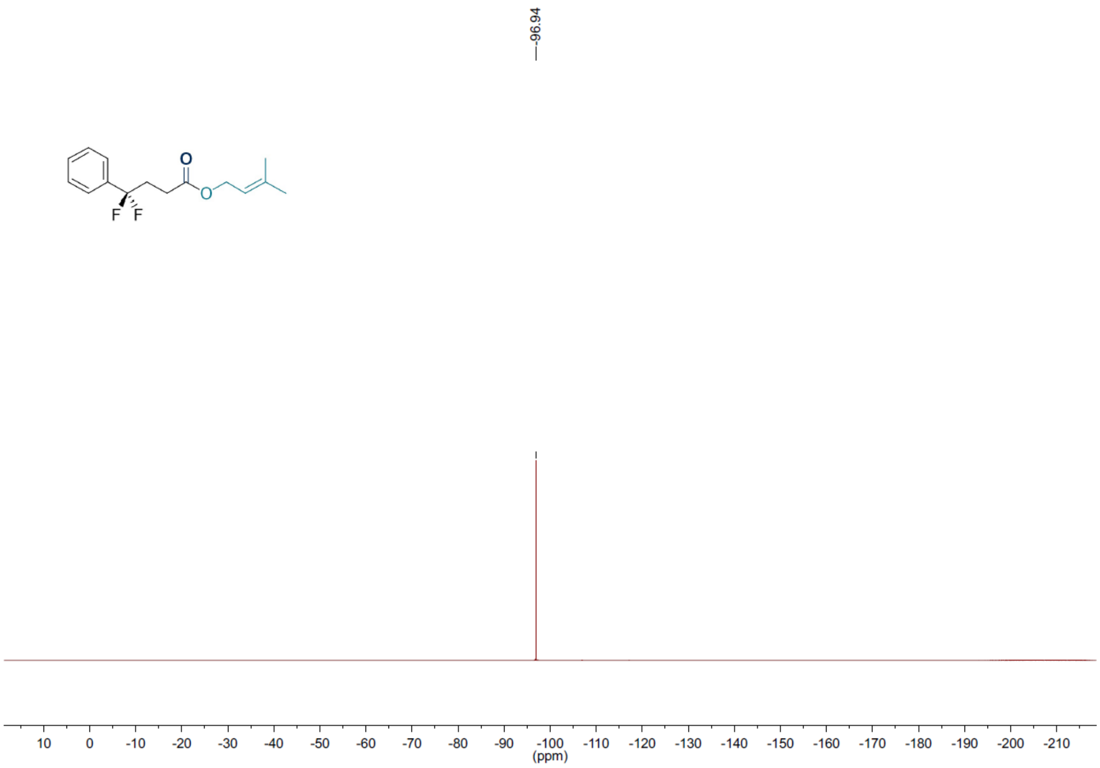

^{19}F NMR spectrum of **3n** in $CDCl_3$ (376 MHz)

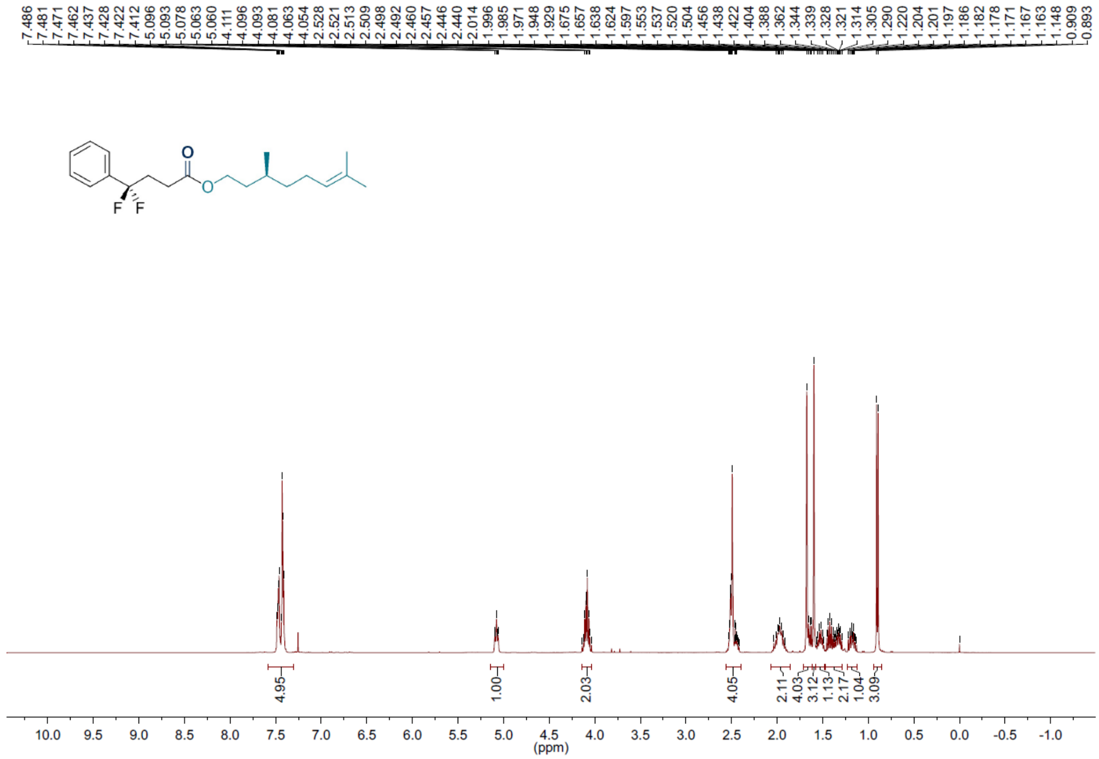

^{1}H NMR spectrum of **3o** in $CDCl_3$ (400 MHz)

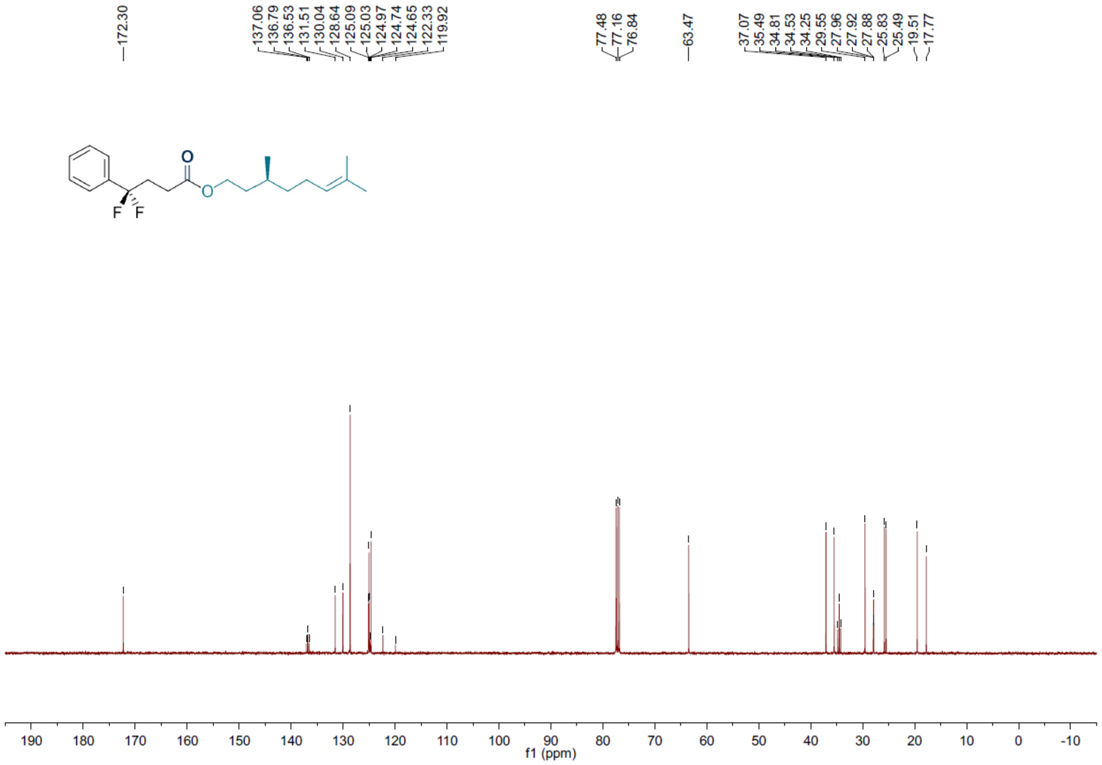

^{13}C NMR spectrum of **3o** in $CDCl_3$ (101 MHz)

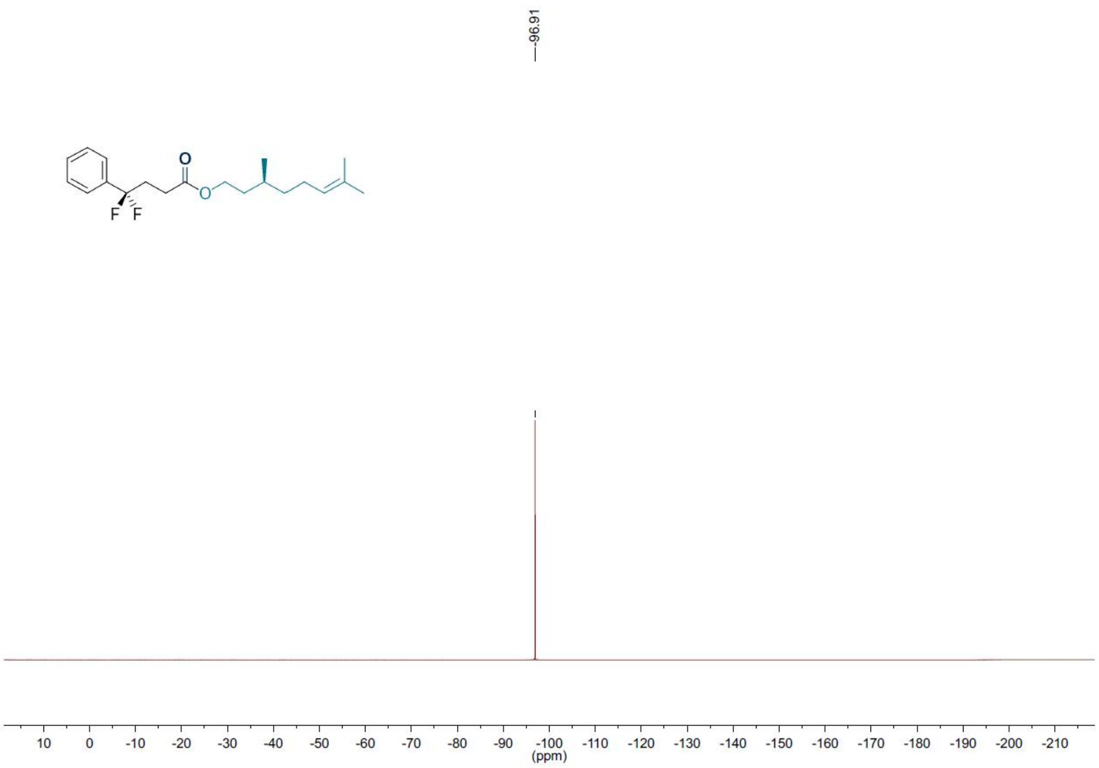

^{19}F NMR spectrum of **3o** in $CDCl_3$ (376 MHz)

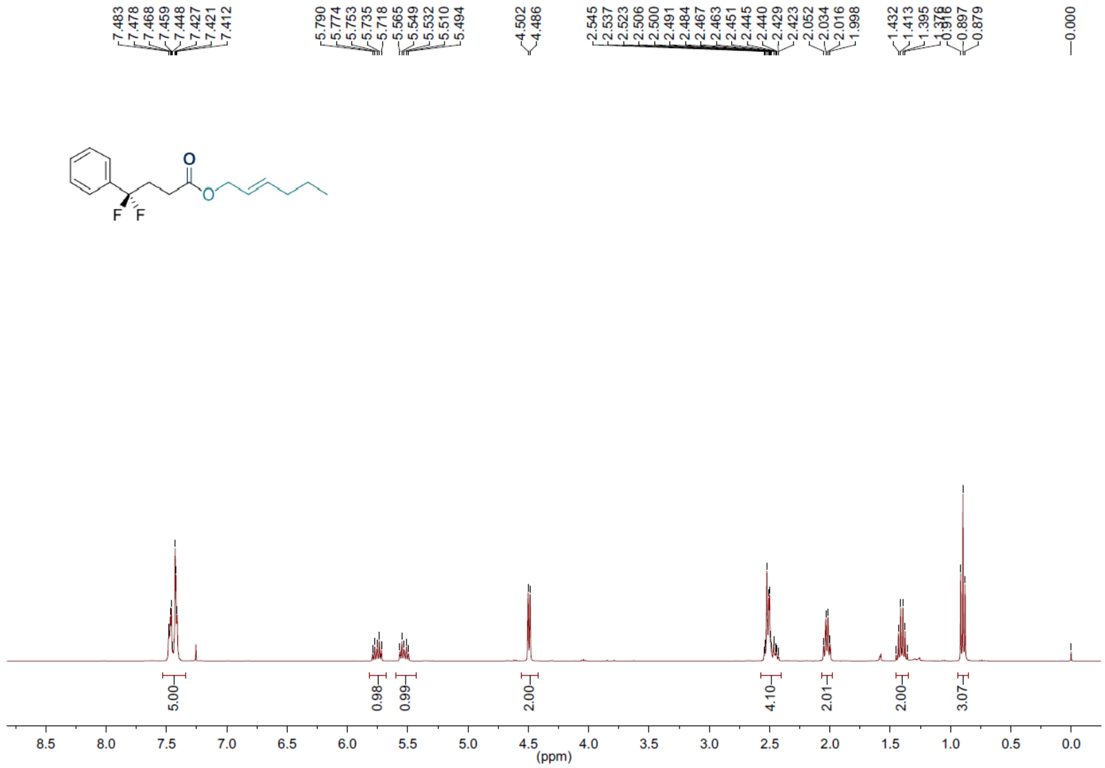

^{1}H NMR spectrum of **3p** in $CDCl_3$ (400 MHz)

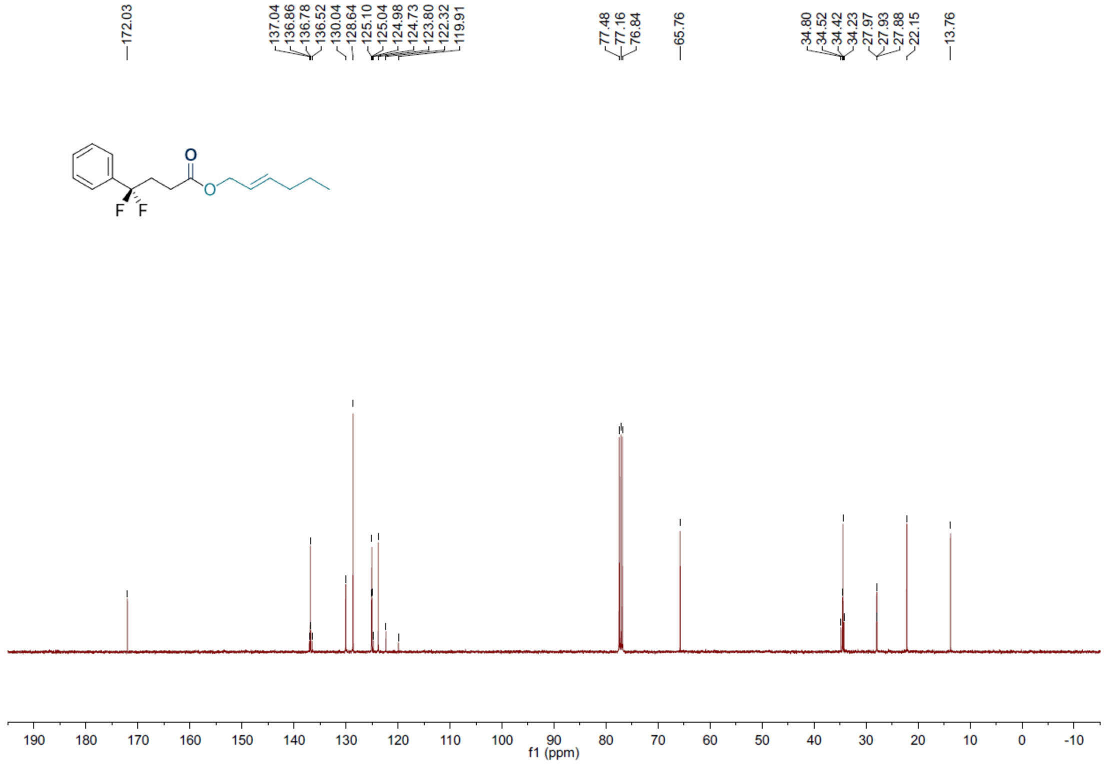

^{13}C NMR spectrum of **3p** in $CDCl_3$ (101 MHz)

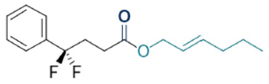

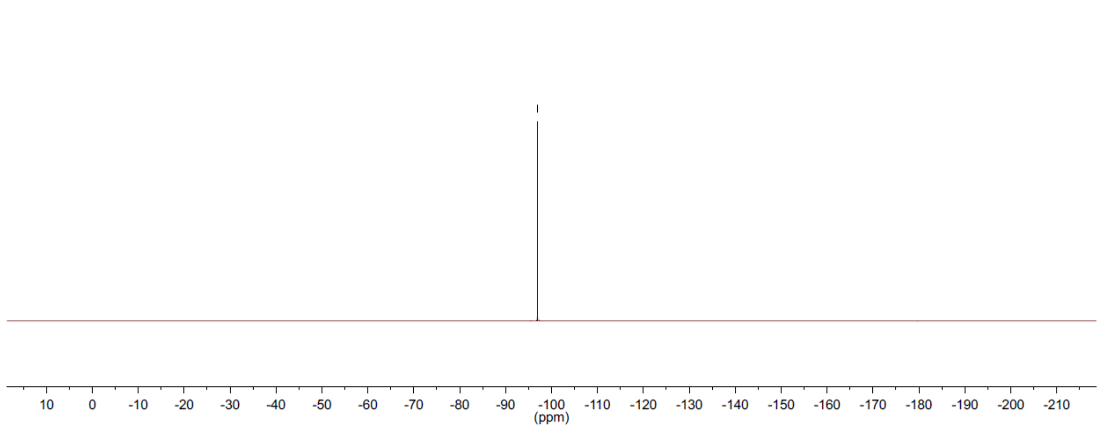

^{19}F NMR spectrum of **3p** in $CDCl_3$ (376 MHz)

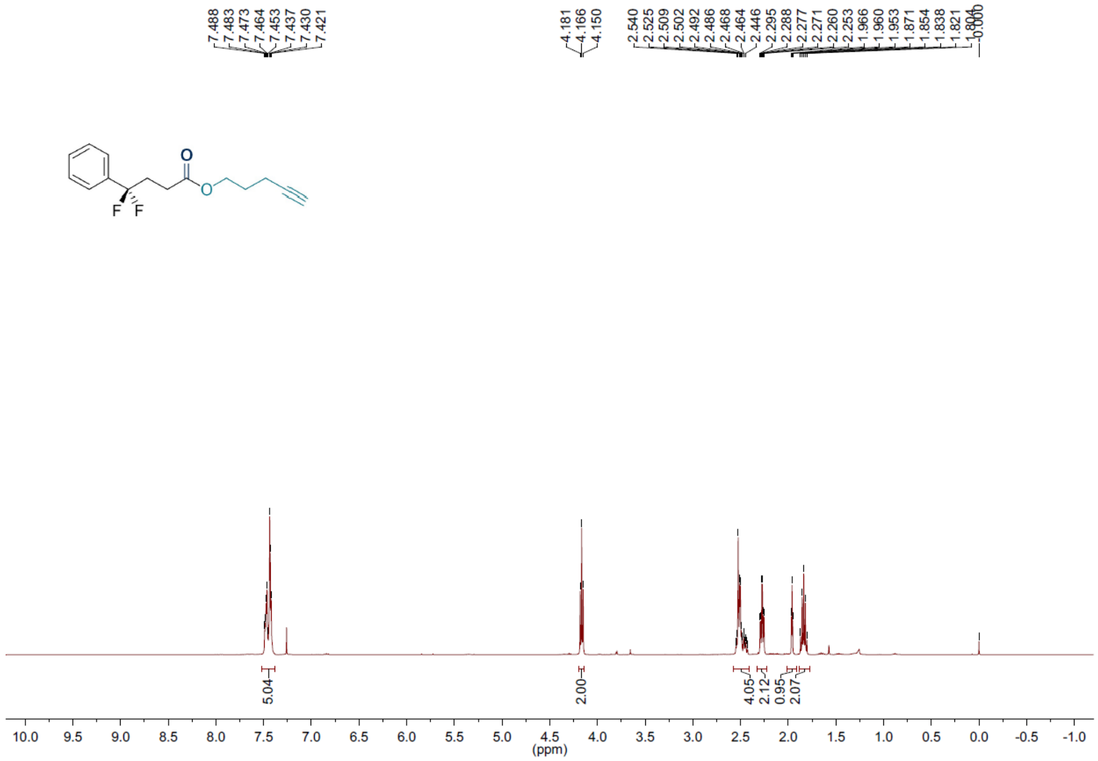

^{1}H NMR spectrum of **3q** in $CDCl_3$ (400 MHz)

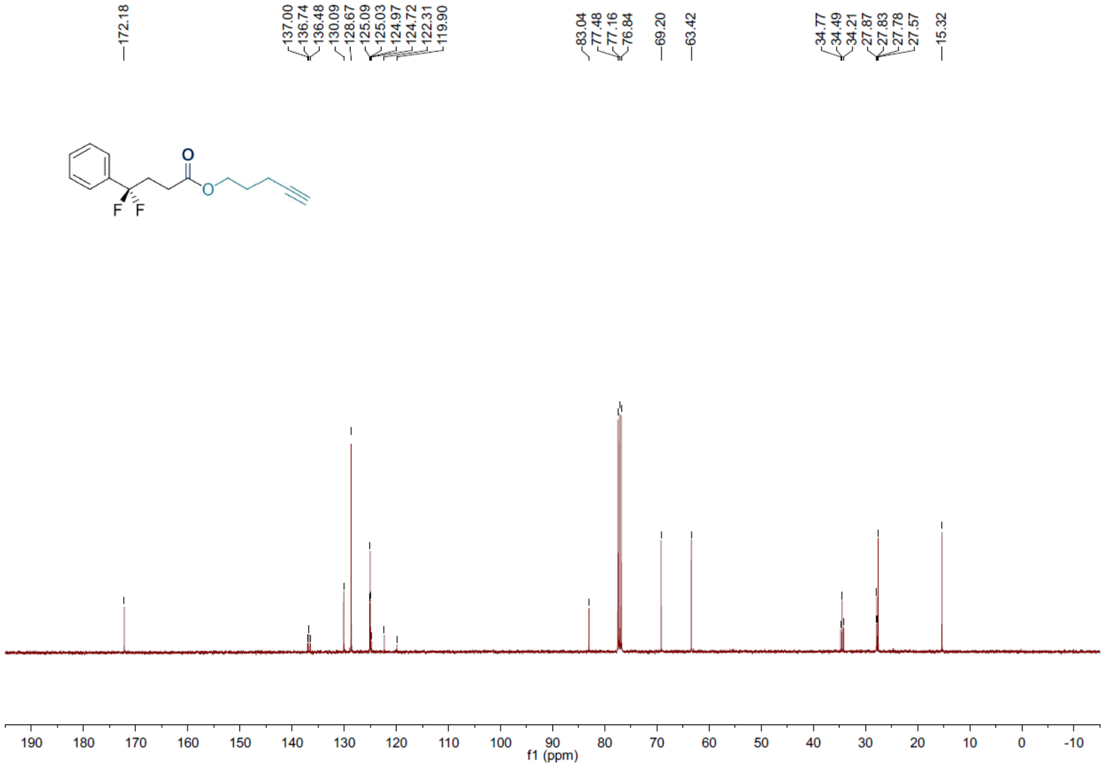

^{13}C NMR spectrum of **3q** in $CDCl_3$ (101 MHz)

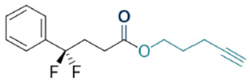

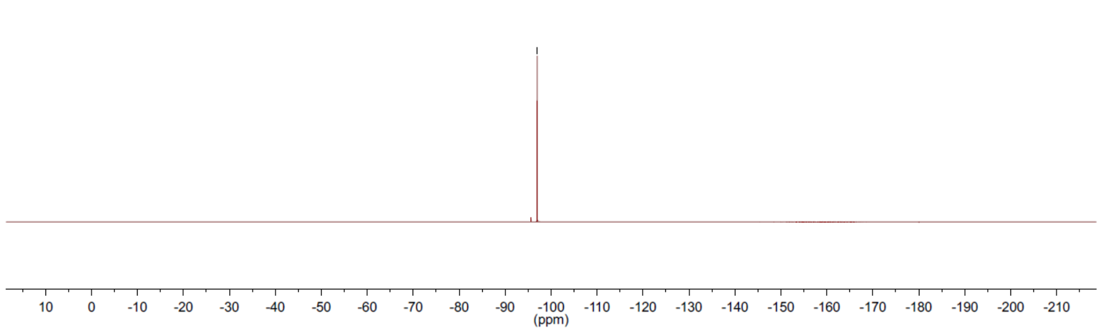

^{19}F NMR spectrum of **3q** in $CDCl_3$ (376 MHz)

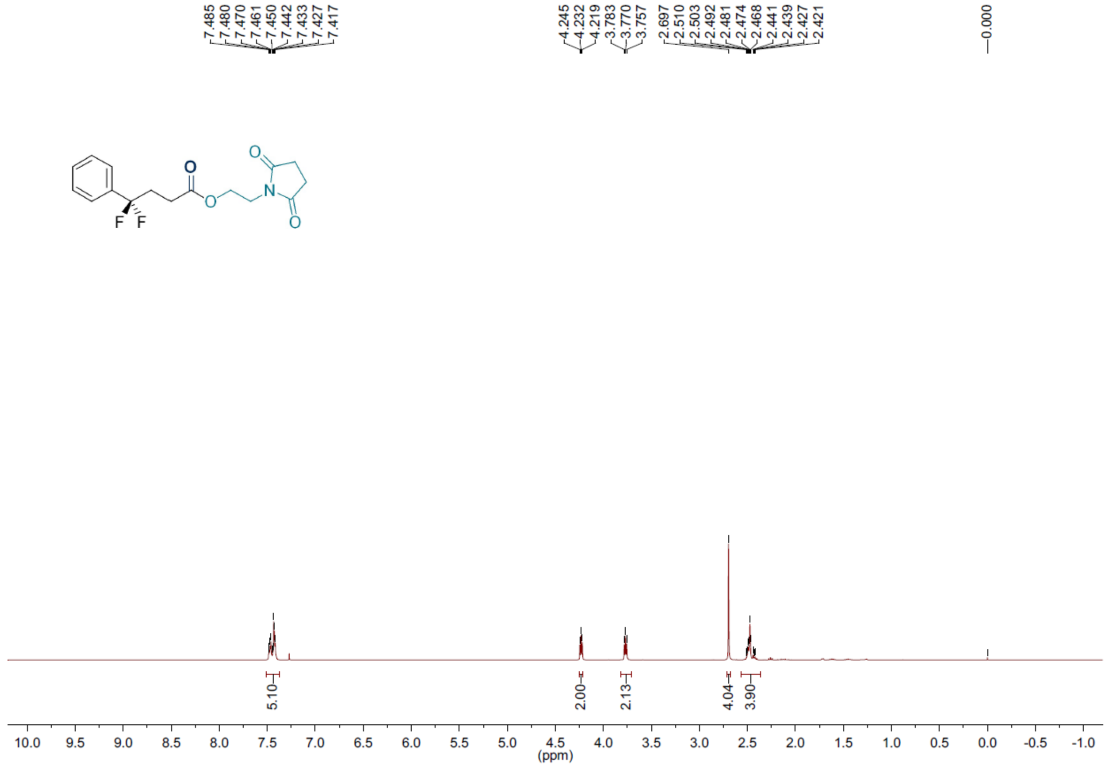

^{1}H NMR spectrum of **3r** in $CDCl_3$ (400 MHz)

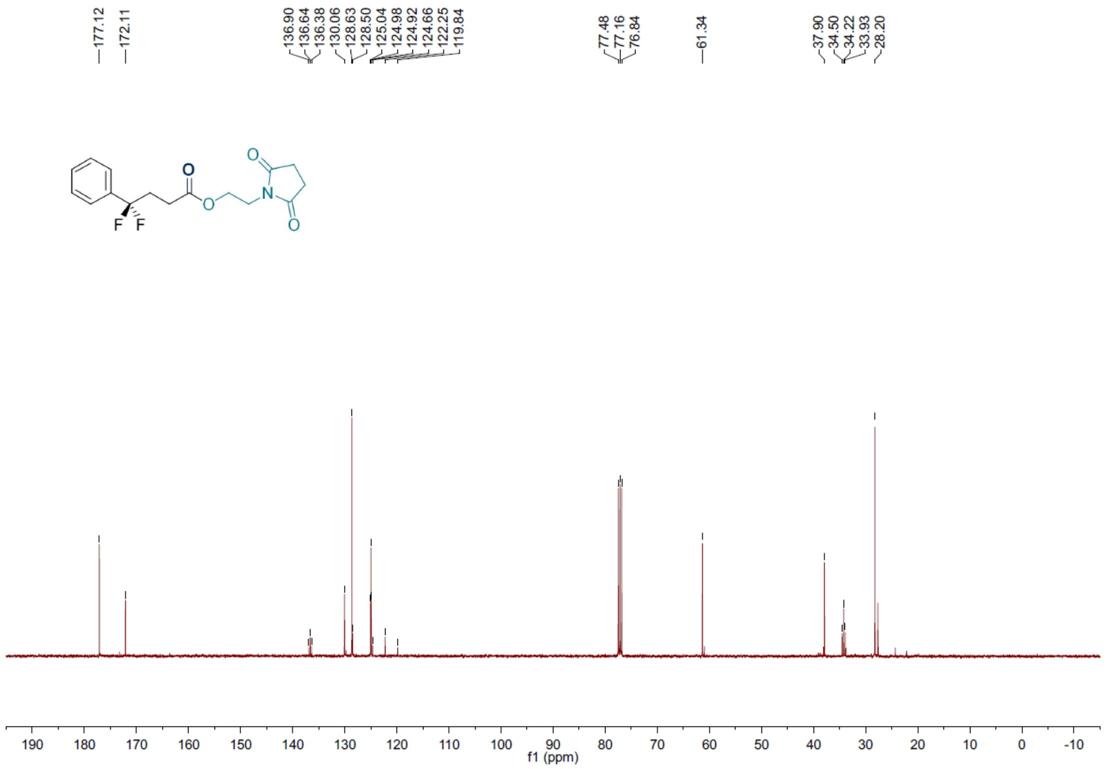

^{13}C NMR spectrum of **3r** in $CDCl_3$ (101 MHz)

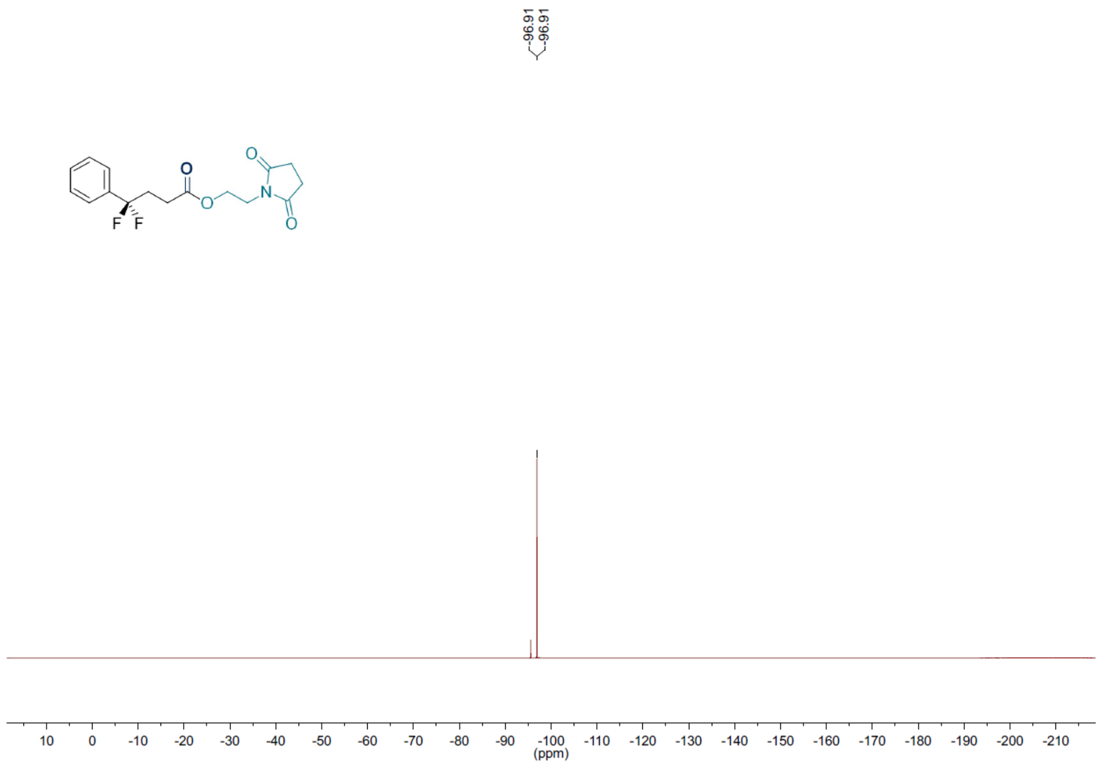

^{19}F NMR spectrum of **3r** in $CDCl_3$ (376 MHz)

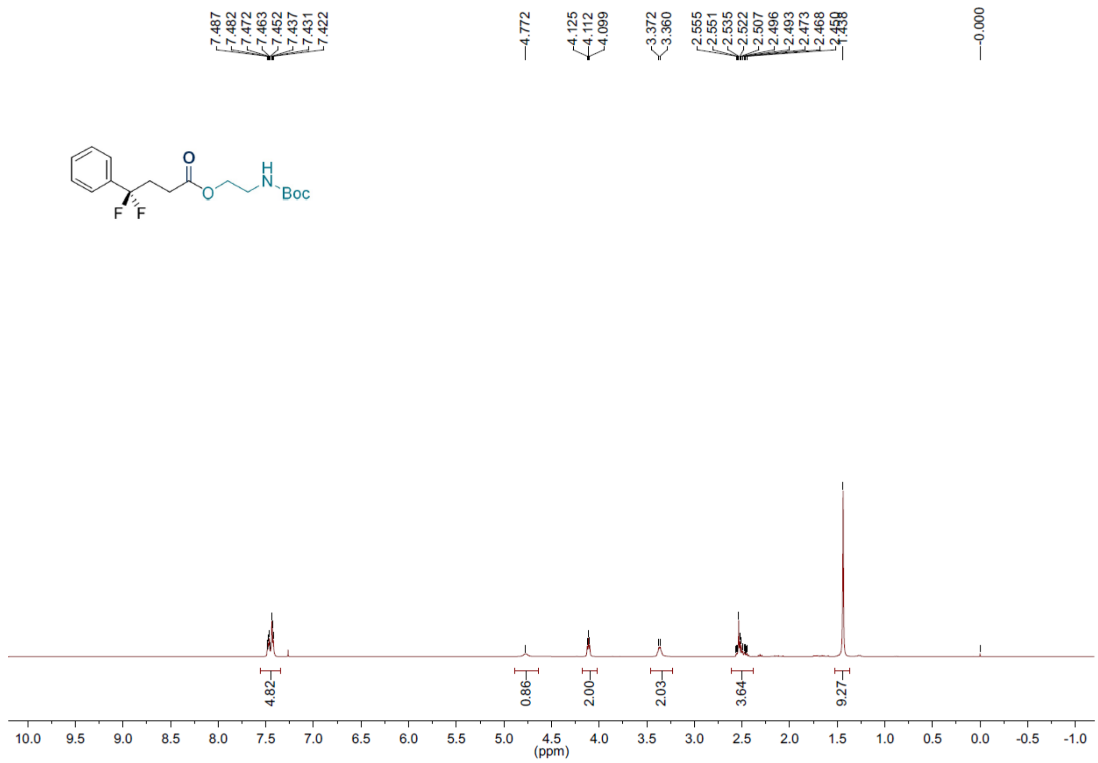

^{1}H NMR spectrum of **3s** in $CDCl_3$ (400 MHz)

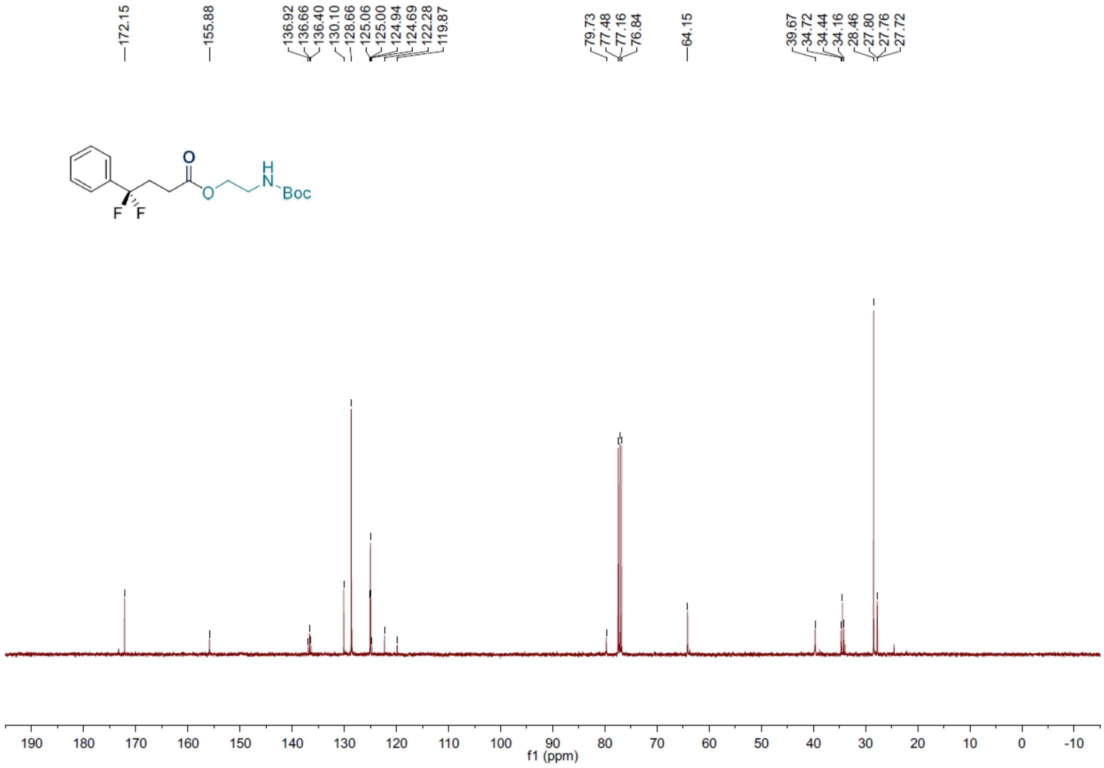

^{13}C NMR spectrum of **3s** in $CDCl_3$ (101 MHz)

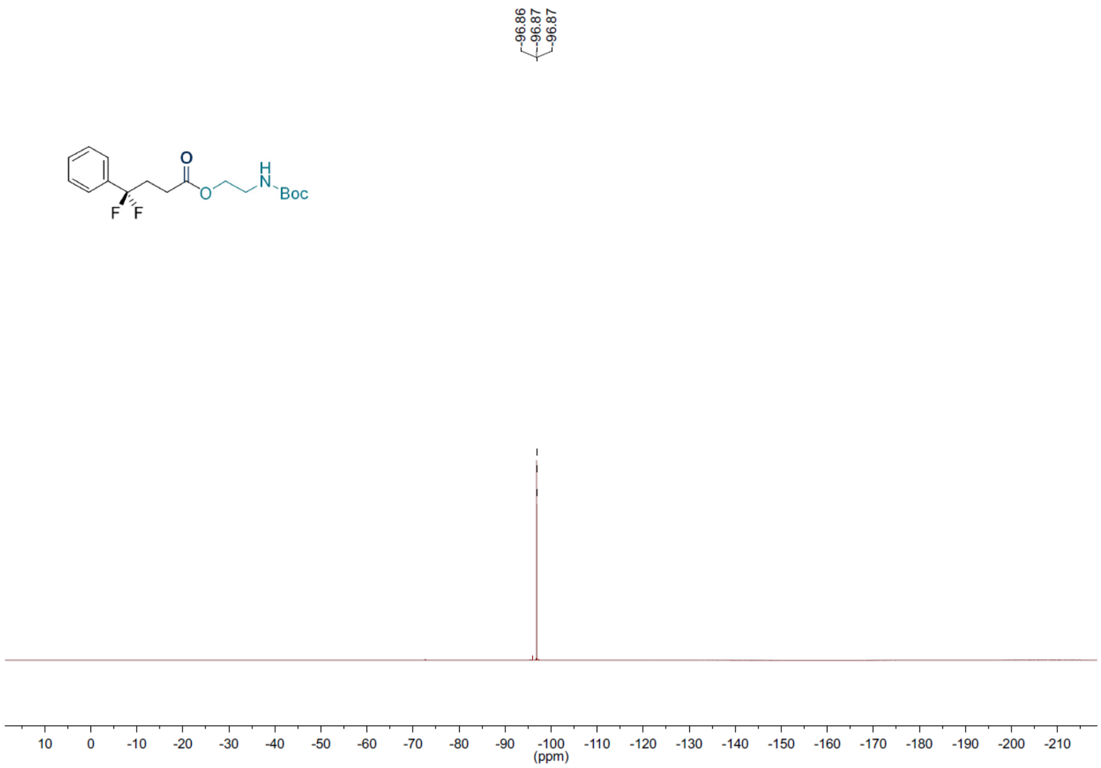

^{19}F NMR spectrum of **3s** in $CDCl_3$ (376 MHz)

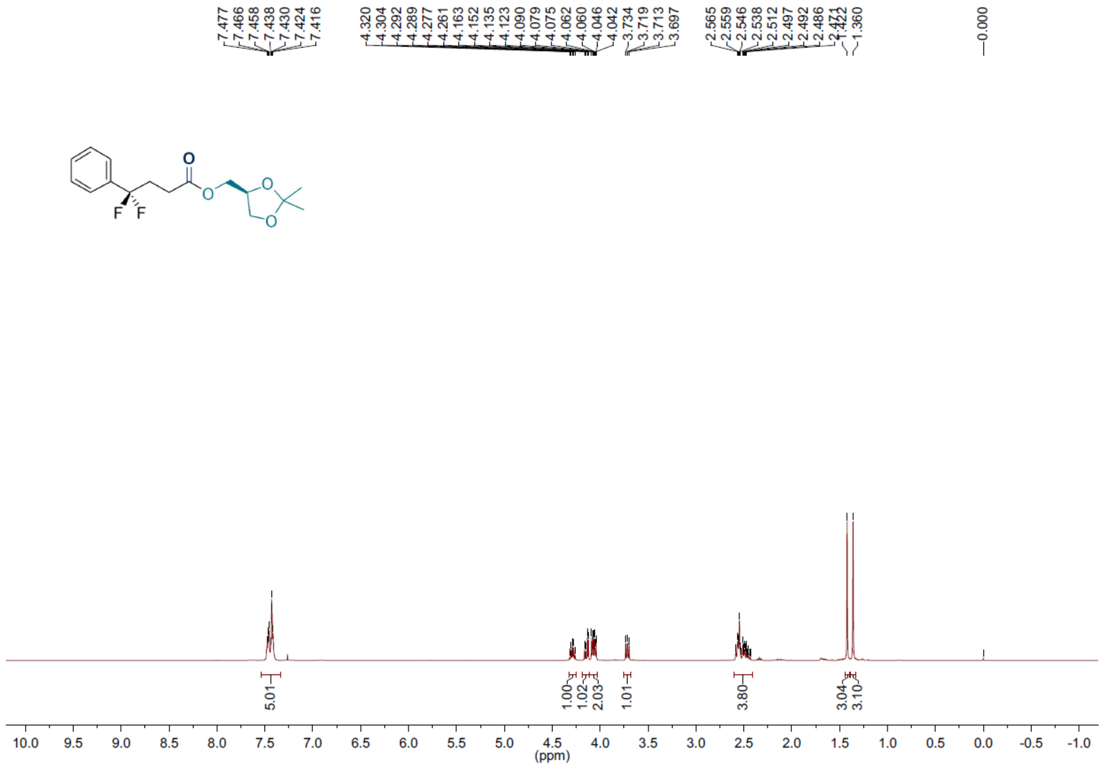

^{1}H NMR spectrum of **3t** in $CDCl_3$ (400 MHz)

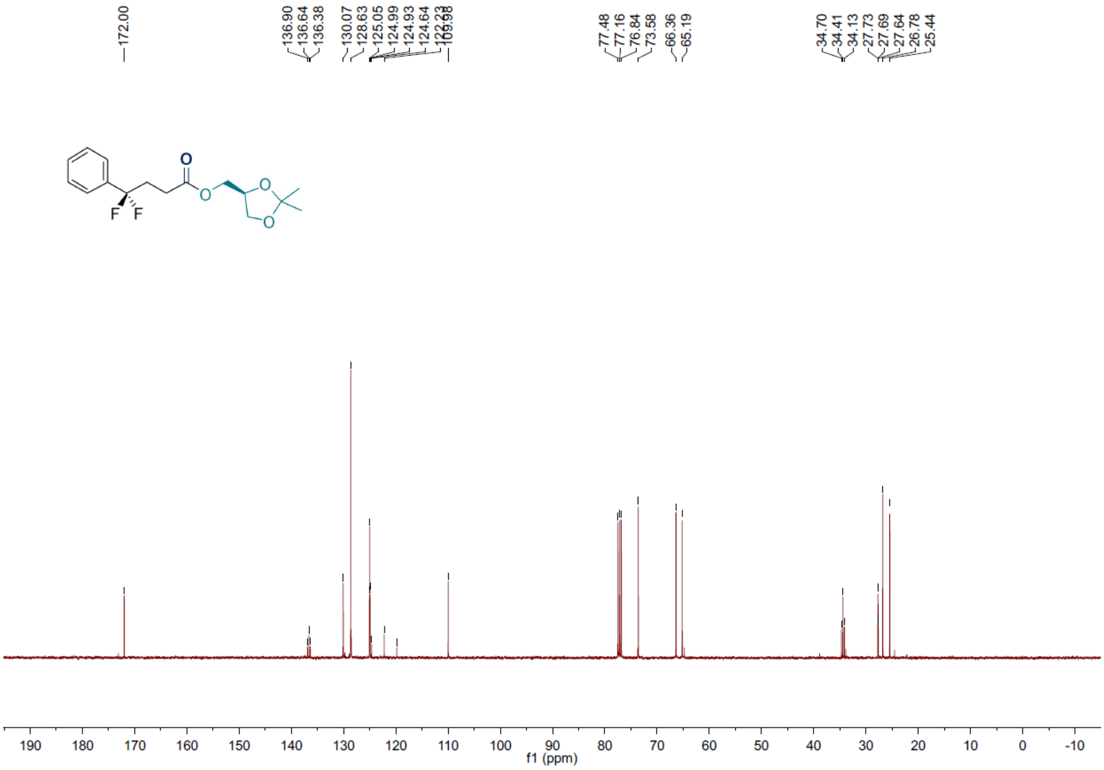

^{13}C NMR spectrum of **3t** in $CDCl_3$ (101 MHz)

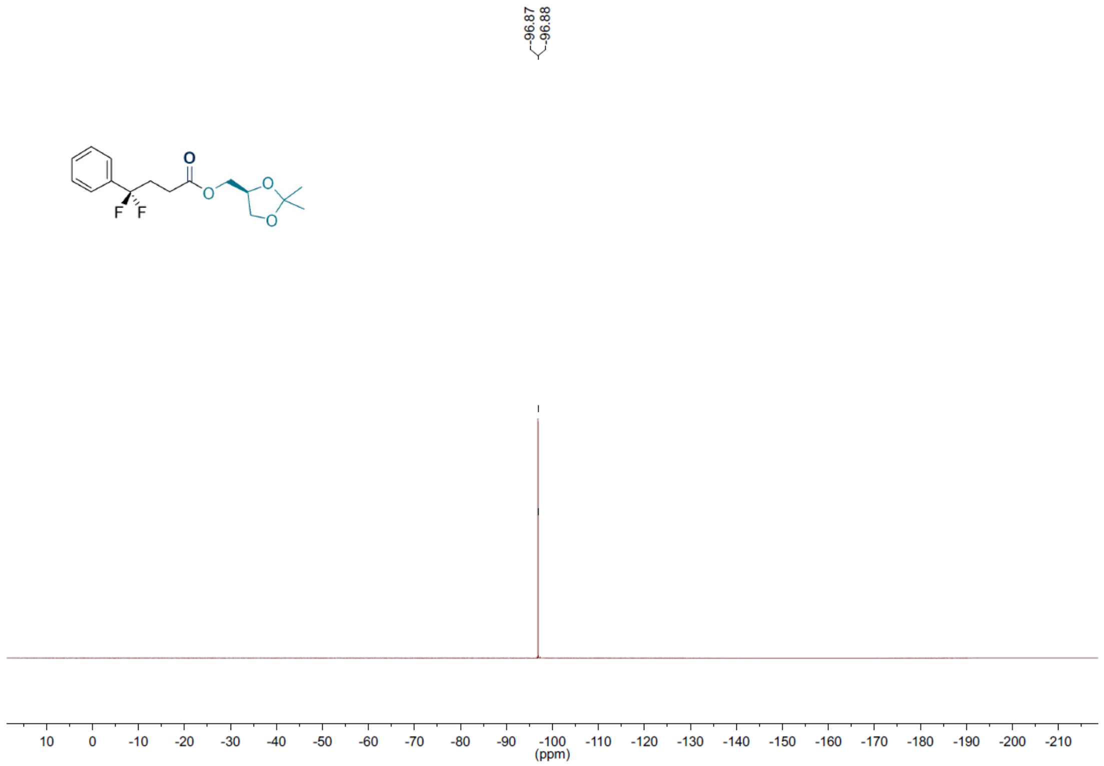

^{19}F NMR spectrum of **3t** in $CDCl_3$ (376 MHz)

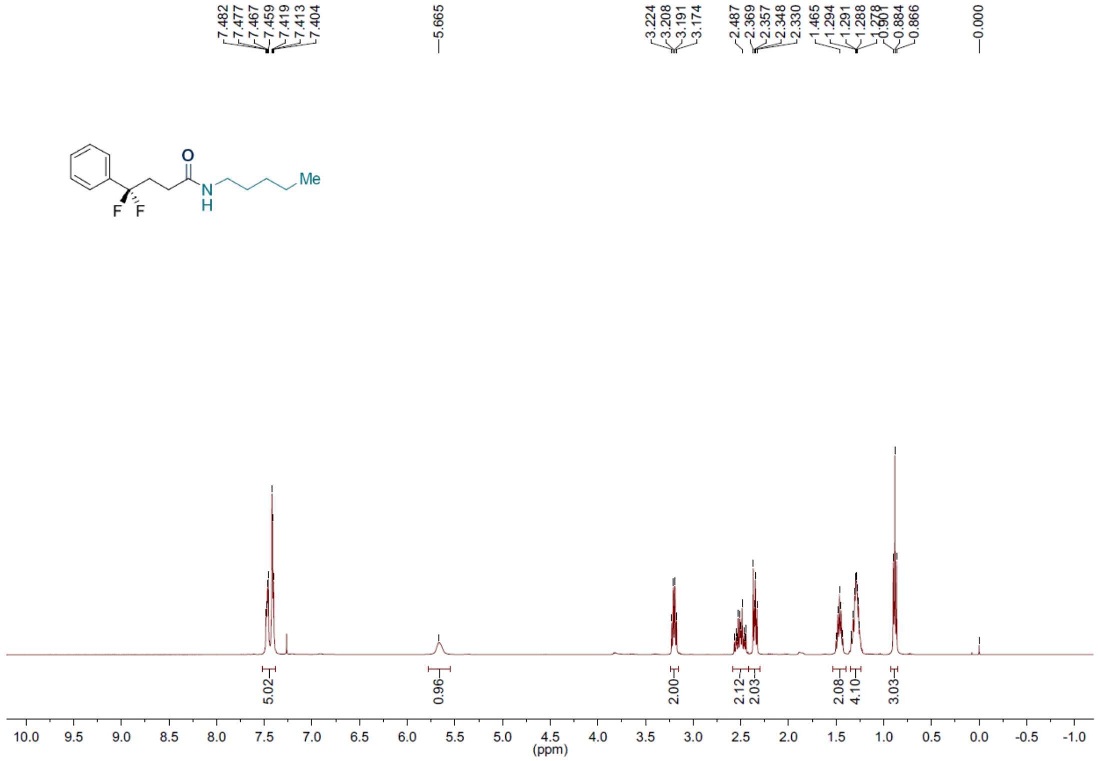

^{1}H NMR spectrum of **4a** in $CDCl_3$ (400 MHz)

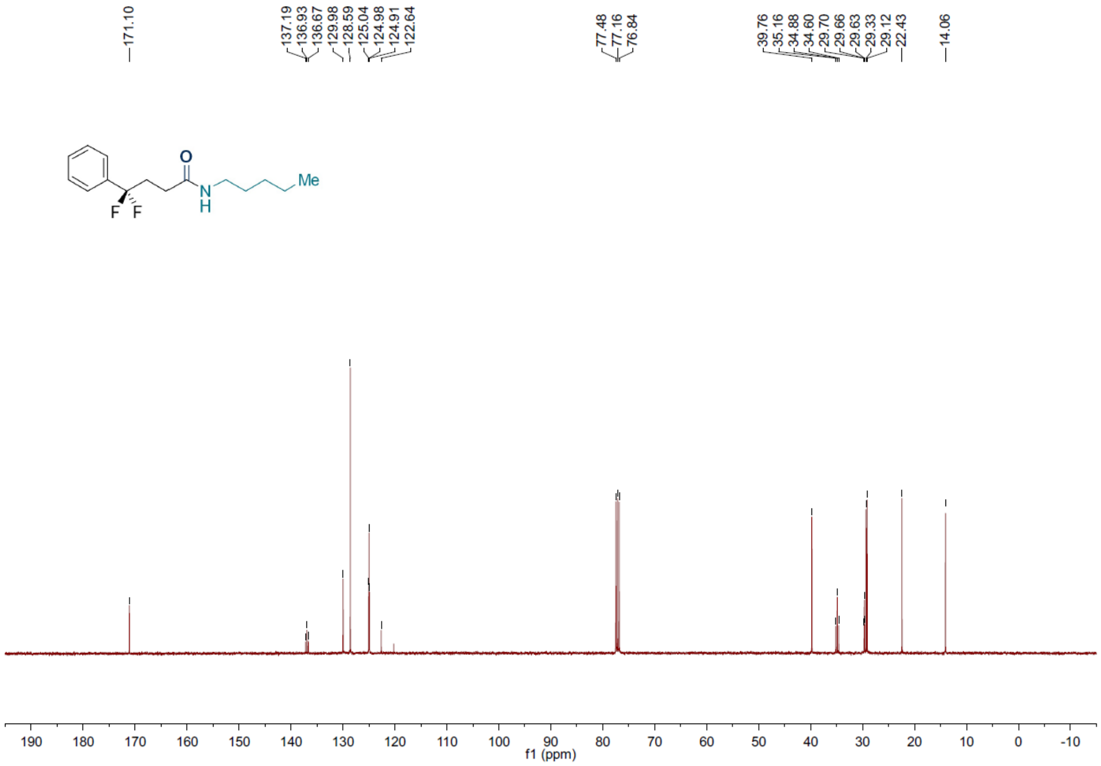

^{13}C NMR spectrum of 4**a** in $CDCl_3$ (101 MHz)

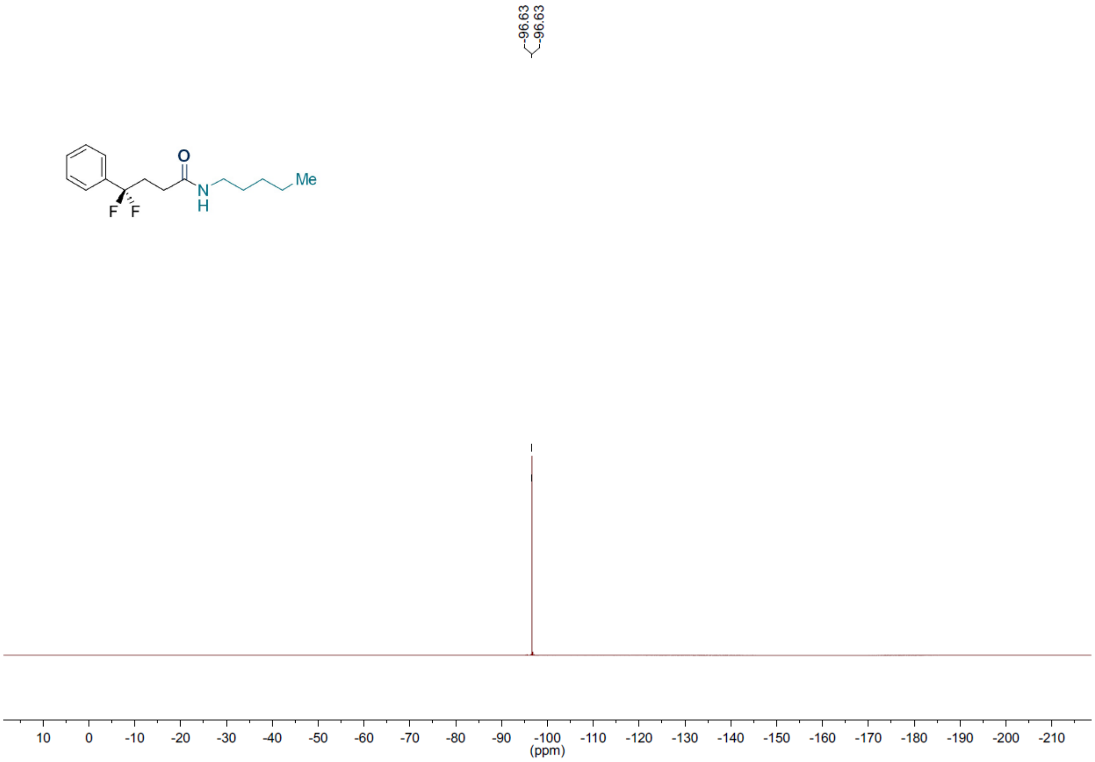

^{19}F NMR spectrum of **4a** in $CDCl_3$ (376 MHz)

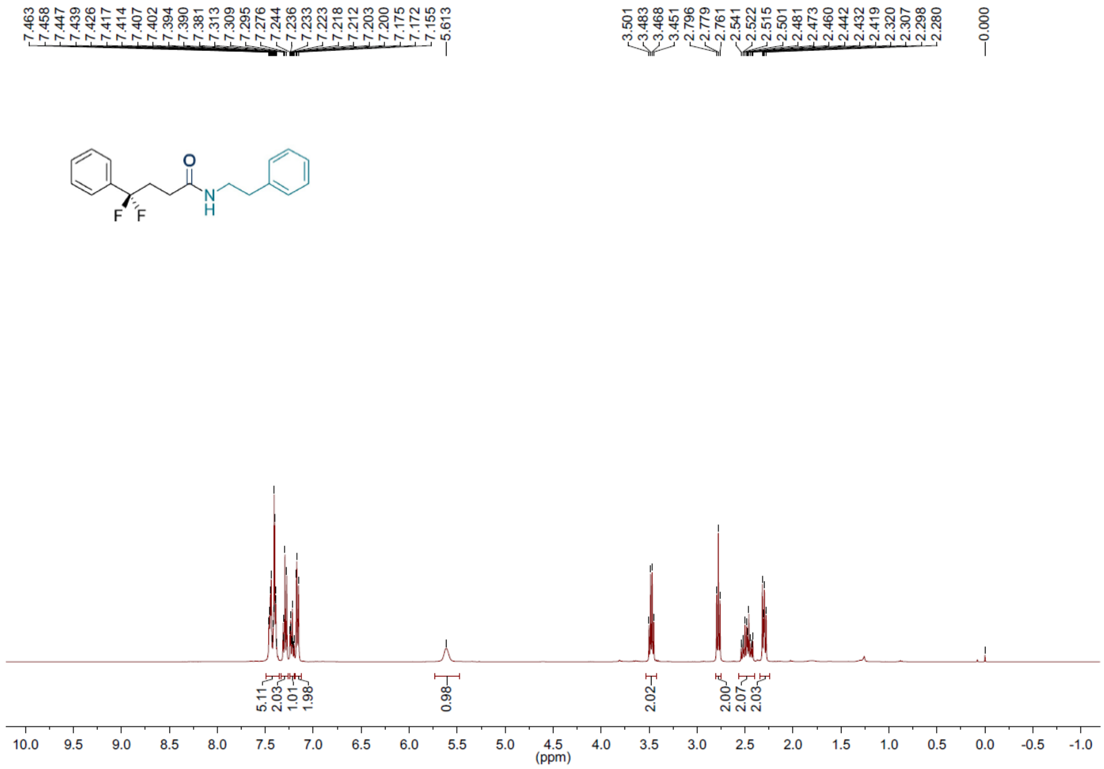

^{1}H NMR spectrum of **4b** in $CDCl_3$ (400 MHz)

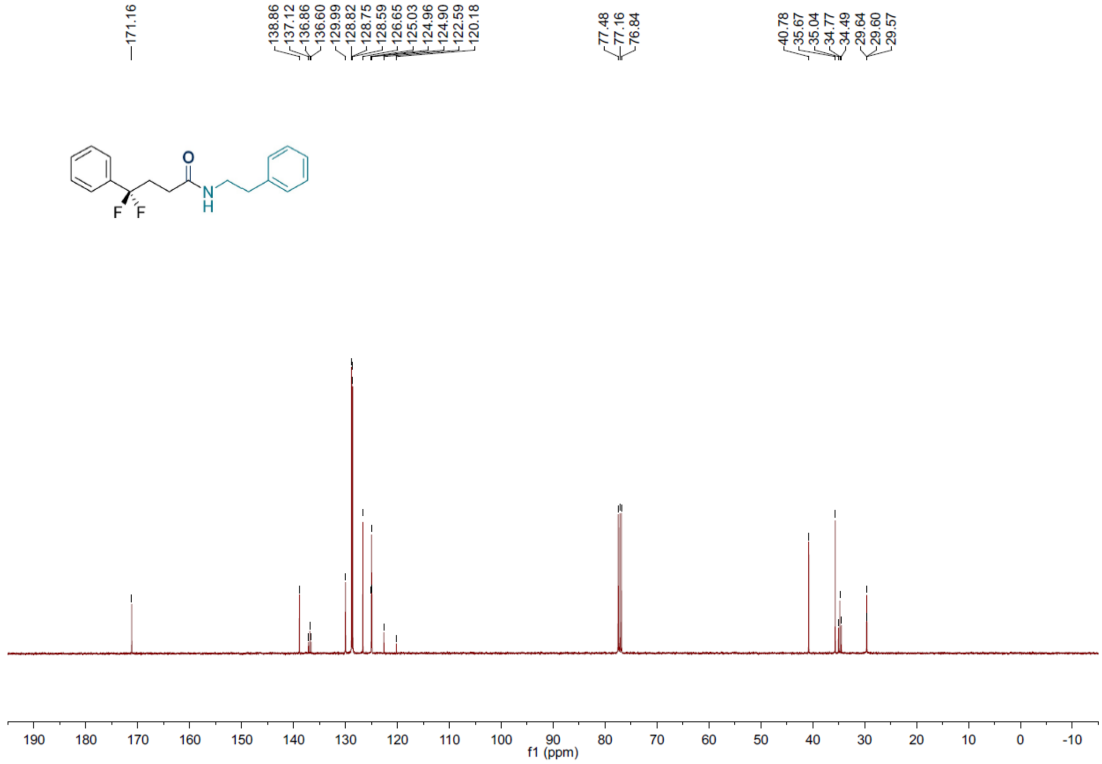

^{13}C NMR spectrum of **4b** in $CDCl_3$ (101 MHz)

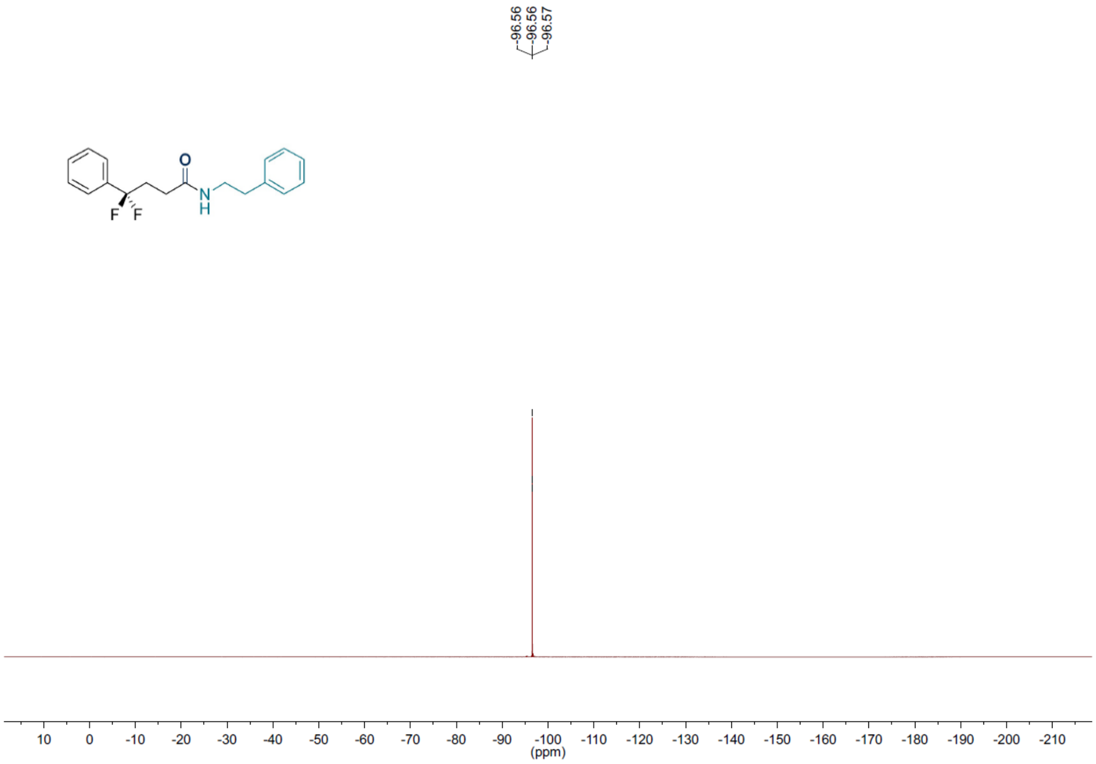

^{19}F NMR spectrum of **4b** in $CDCl_3$ (376 MHz)

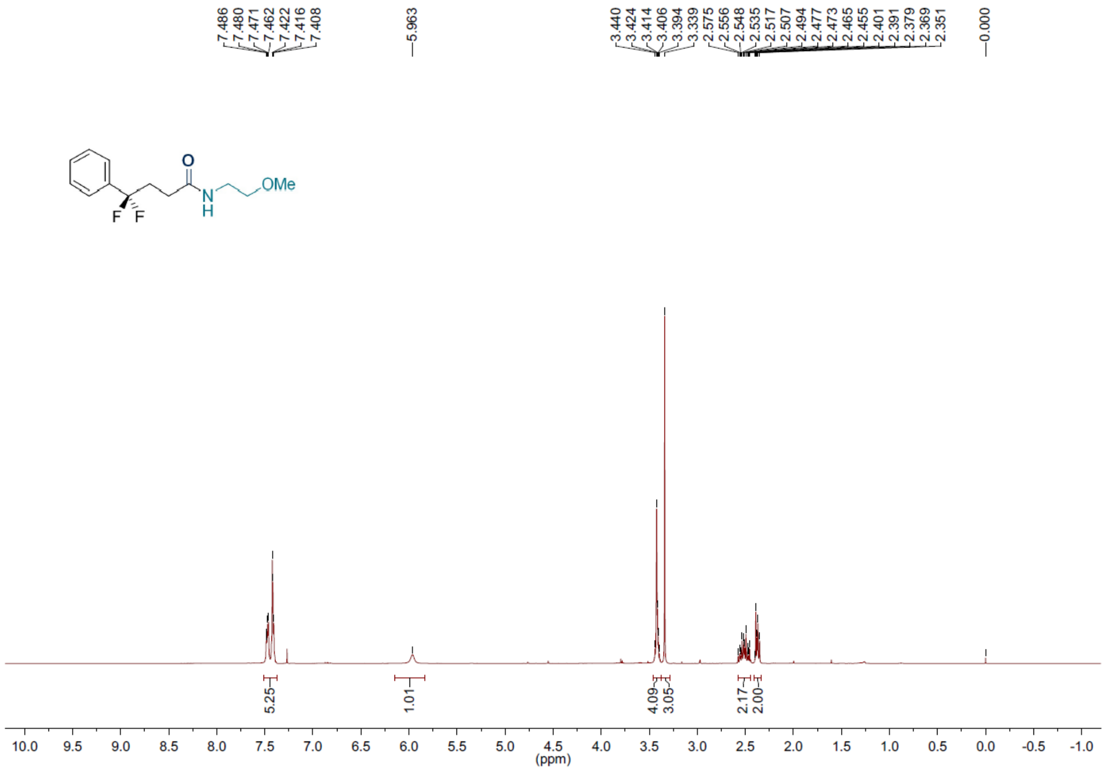

^{1}H NMR spectrum of **4c** in $CDCl_3$ (400 MHz)

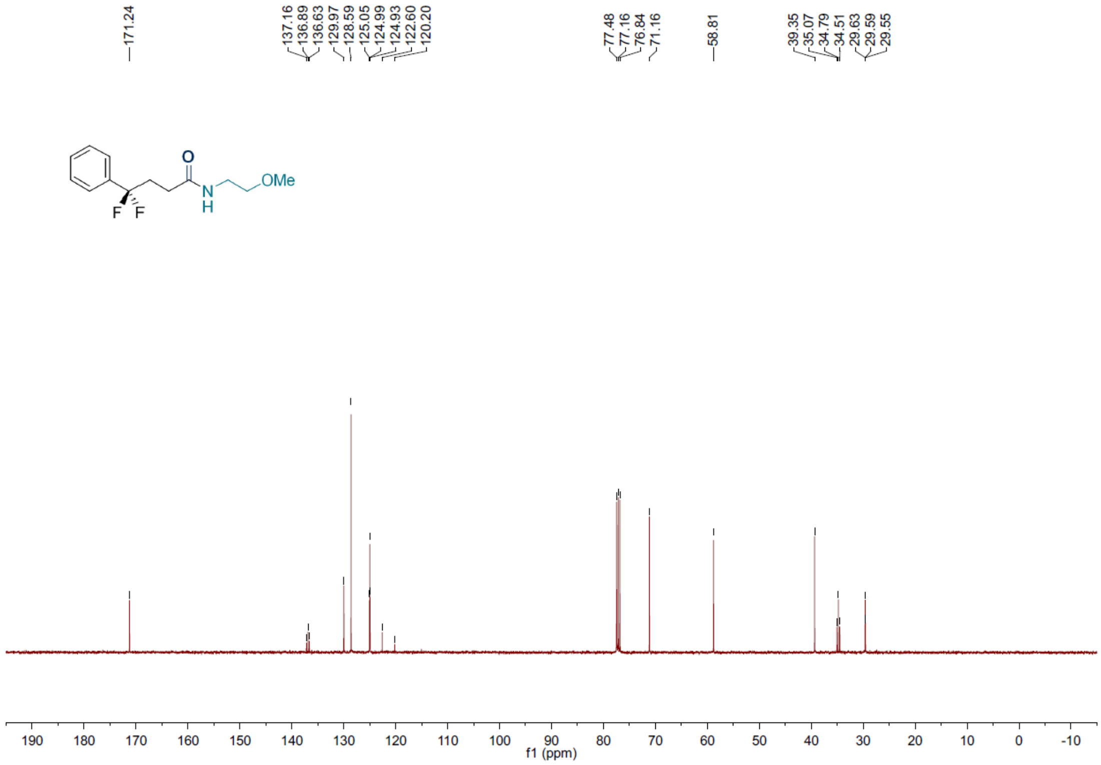

^{13}C NMR spectrum of **4c** in $CDCl_3$ (101 MHz)

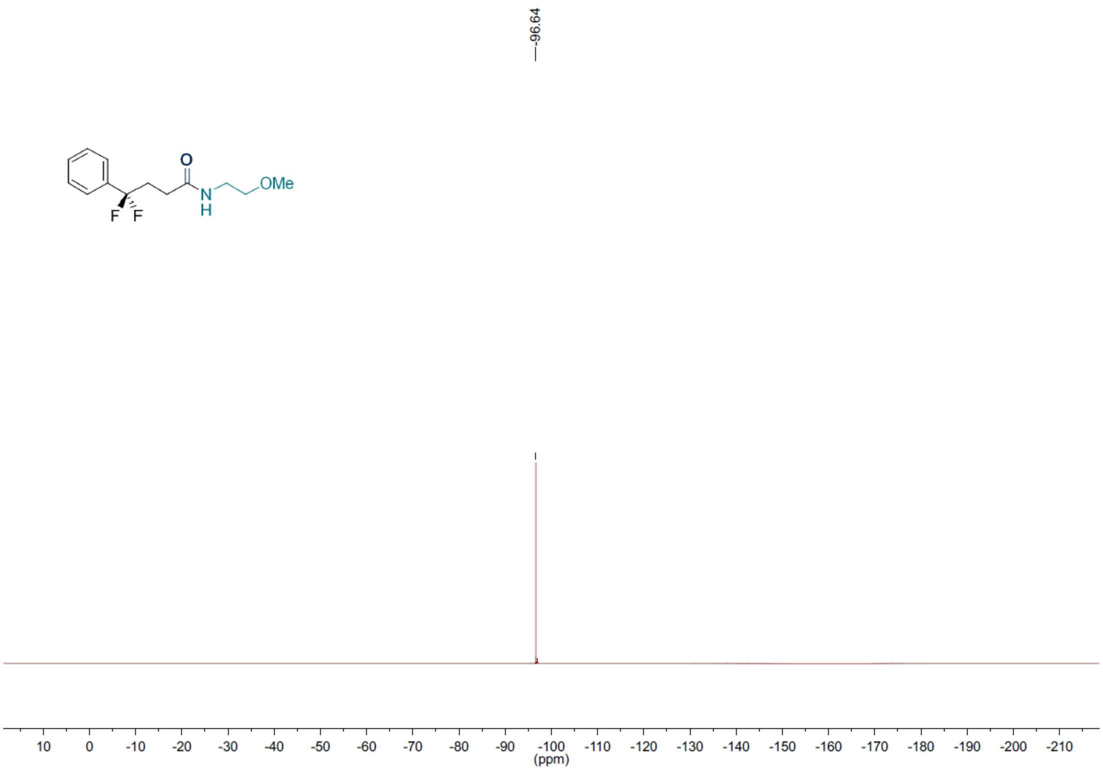

^{19}F NMR spectrum of **4c** in $CDCl_3$ (376 MHz)

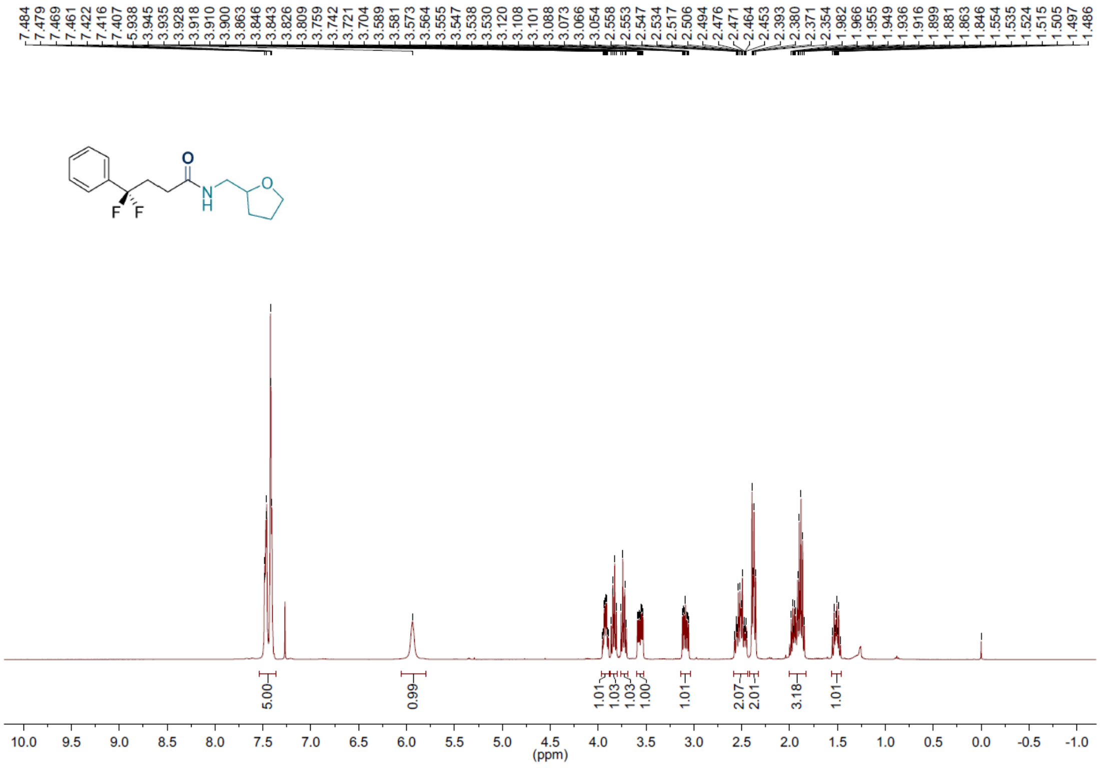

^{1}H NMR spectrum of **4d** in $CDCl_3$ (400 MHz)

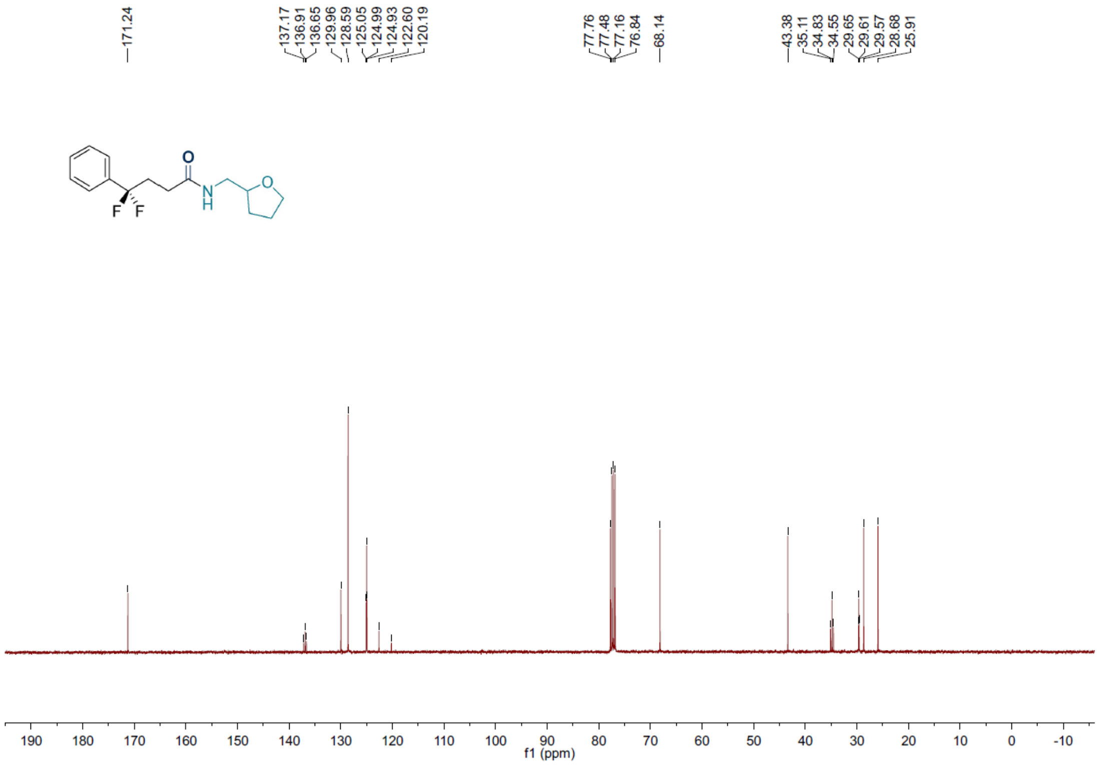

^{13}C NMR spectrum of **4d** in $CDCl_3$ (101 MHz)

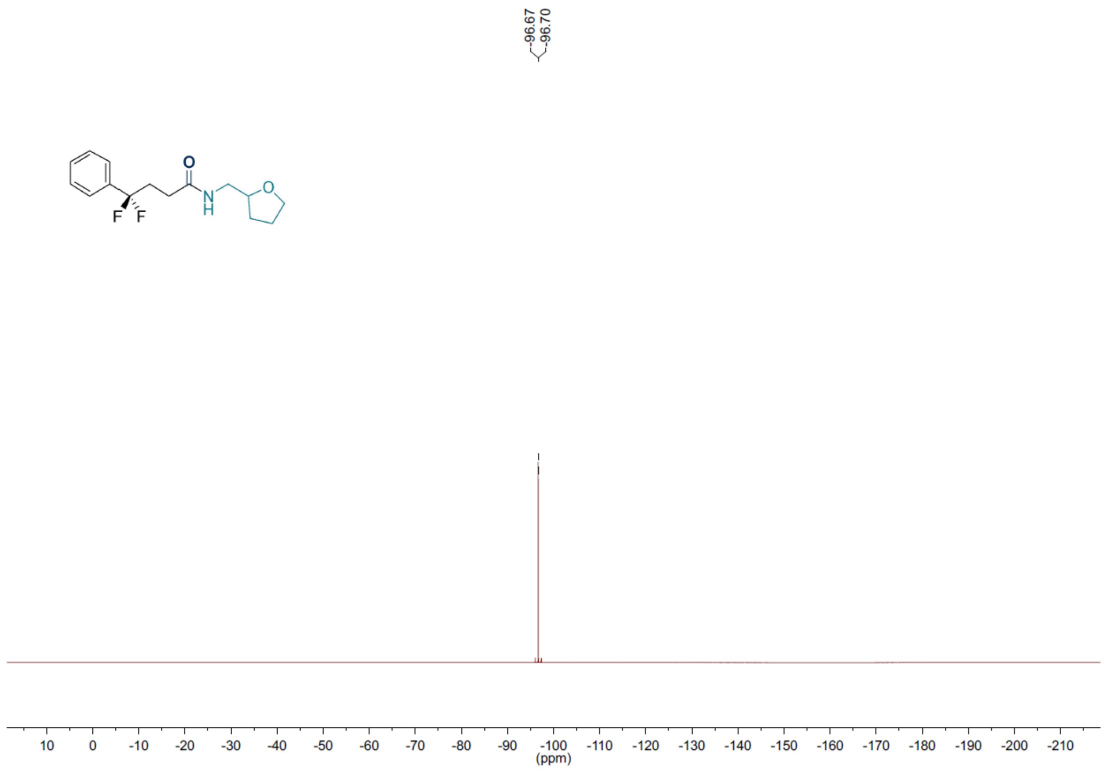

^{19}F NMR spectrum of **4d** in $CDCl_3$ (376 MHz)

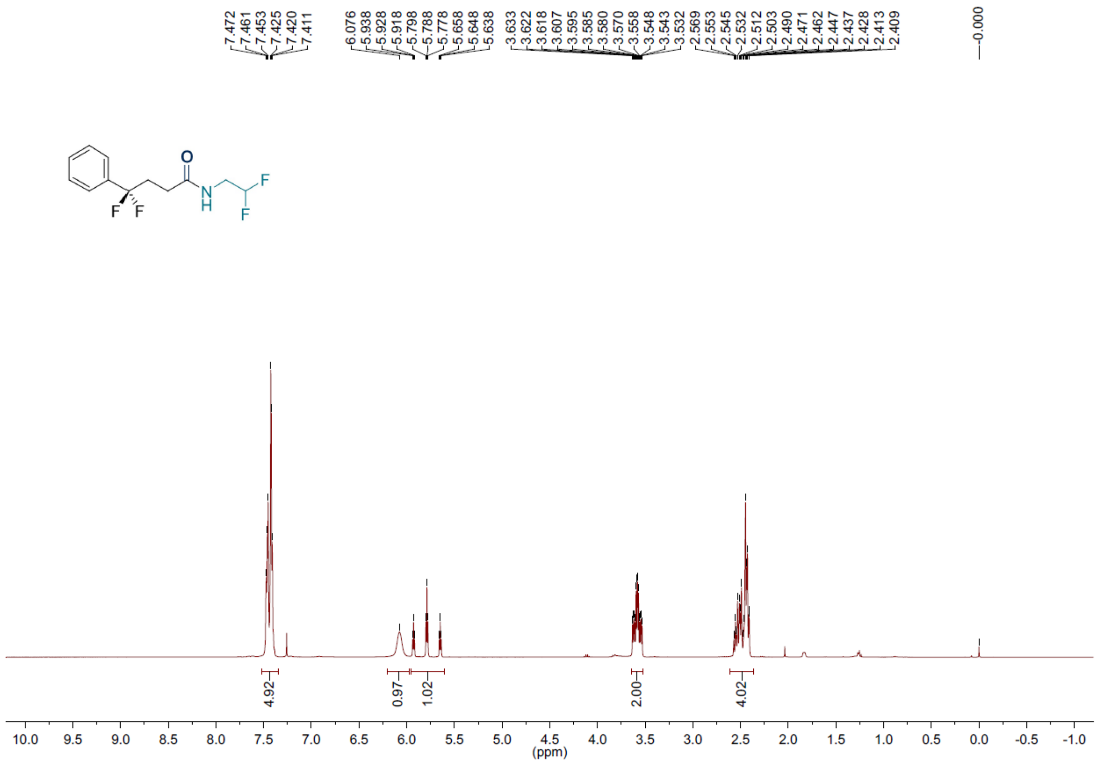

^{1}H NMR spectrum of **4e** in $CDCl_3$ (400 MHz)

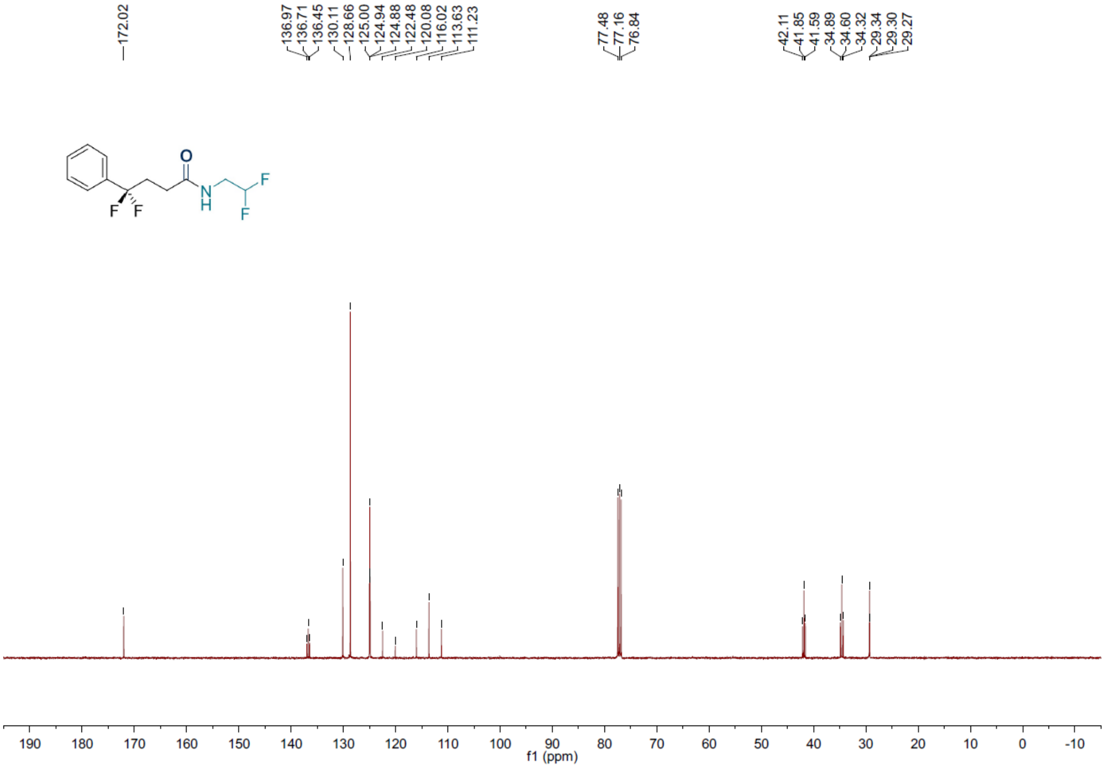

^{13}C NMR spectrum of **4e** in $CDCl_3$ (101 MHz)

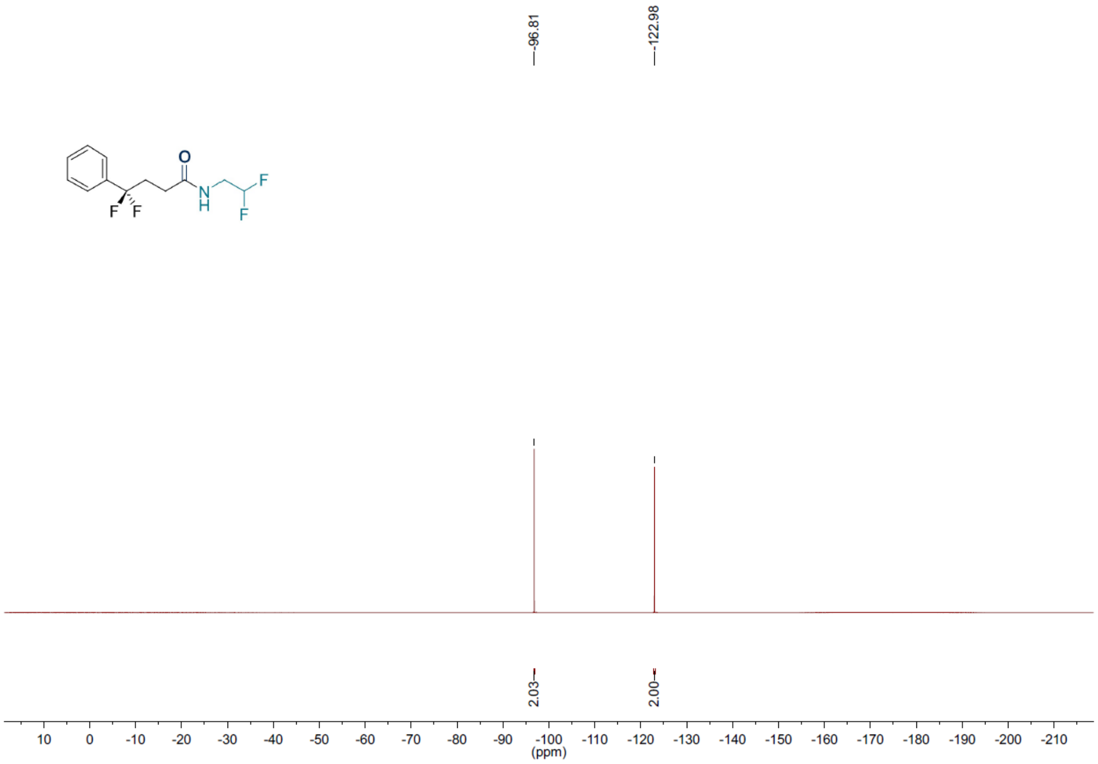

^{19}F NMR spectrum of **4e** in $CDCl_3$ (376 MHz)

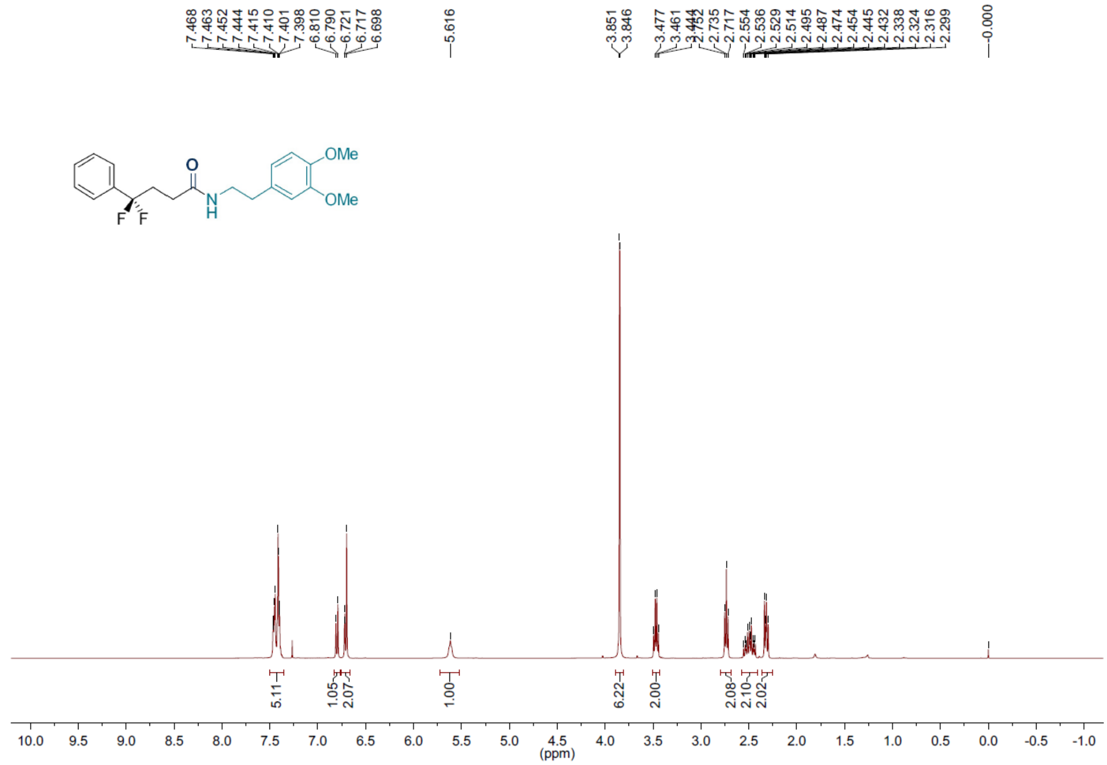

^{1}H NMR spectrum of **4f** in $CDCl_3$ (400 MHz)

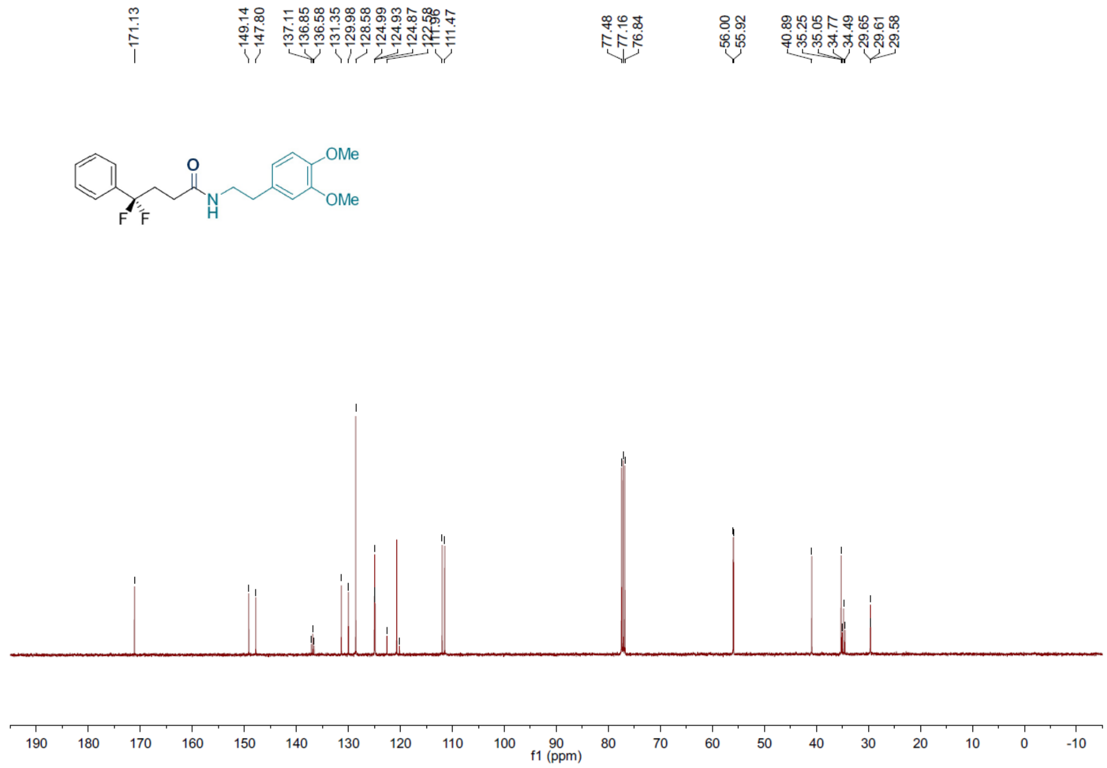

^{13}C NMR spectrum of **4f** in $CDCl_3$ (101 MHz)

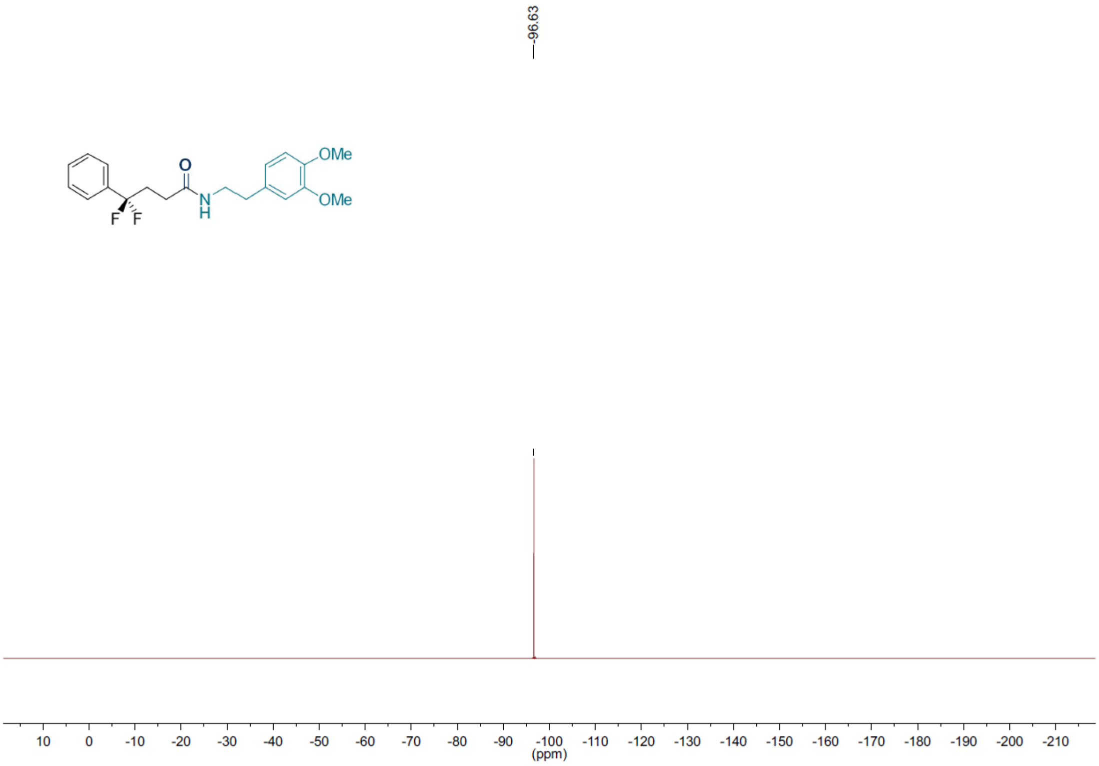

^{19}F NMR spectrum of **4f** in $CDCl_3$ (376 MHz)

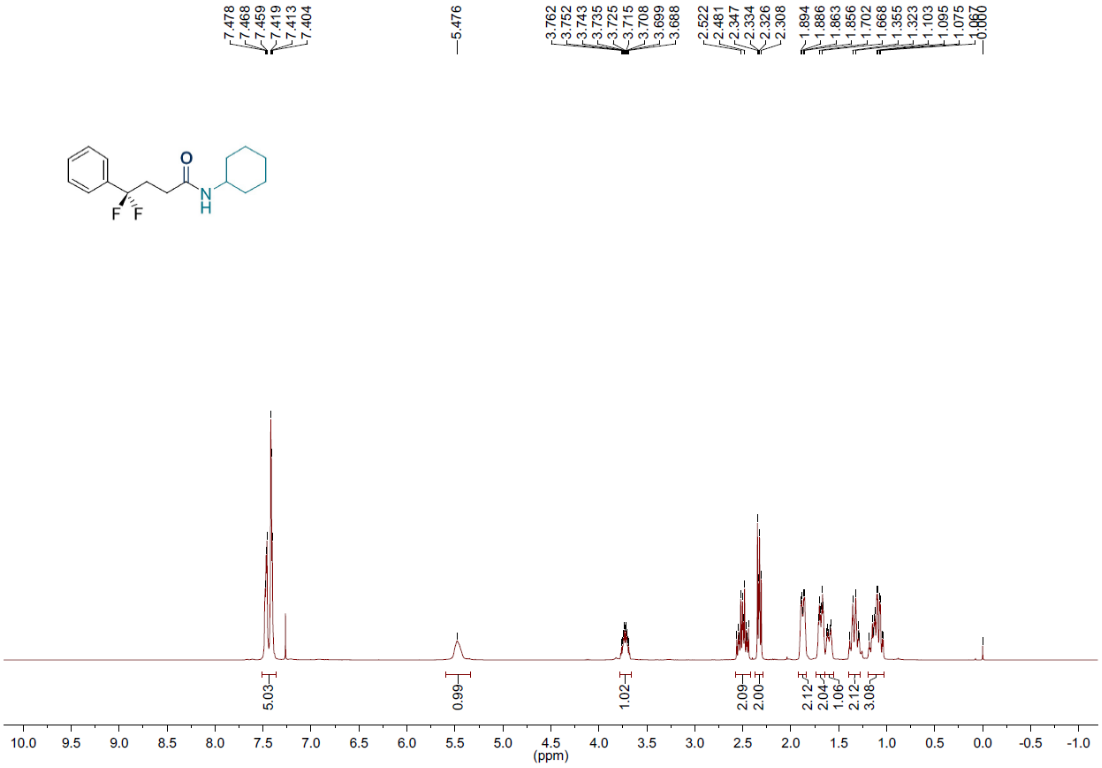

^{1}H NMR spectrum of **4g** in $CDCl_3$ (400 MHz)

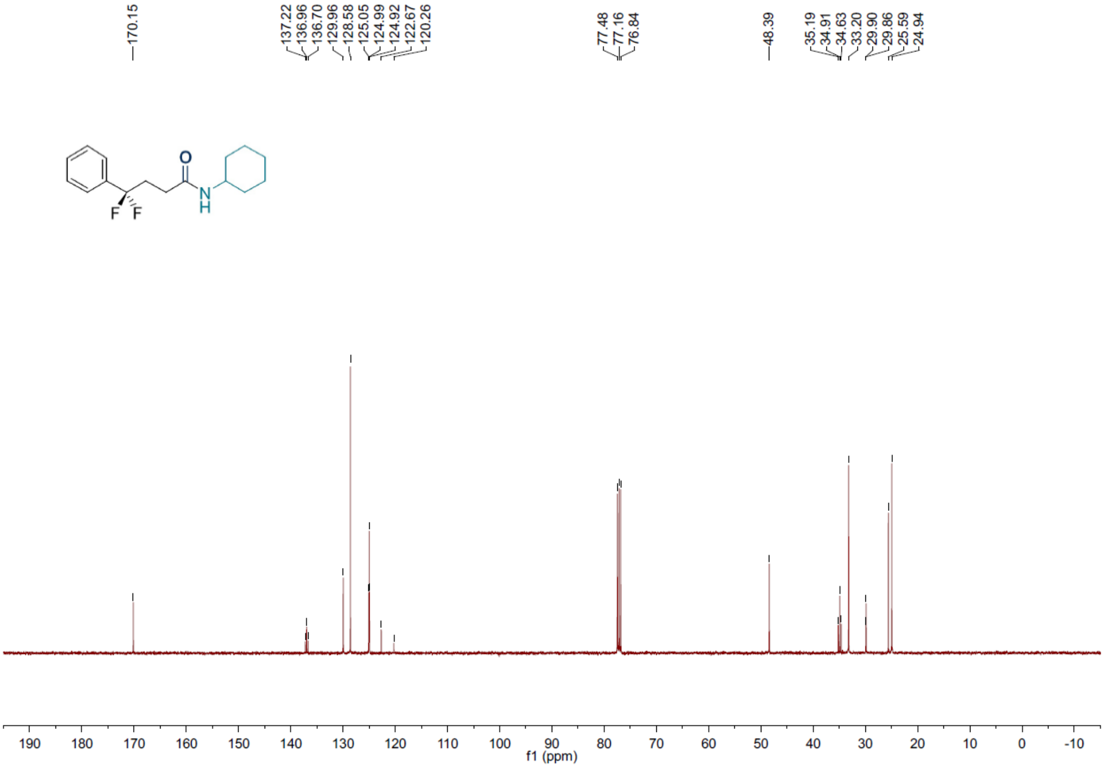

^{13}C NMR spectrum of **4g** in $CDCl_3$ (101 MHz)

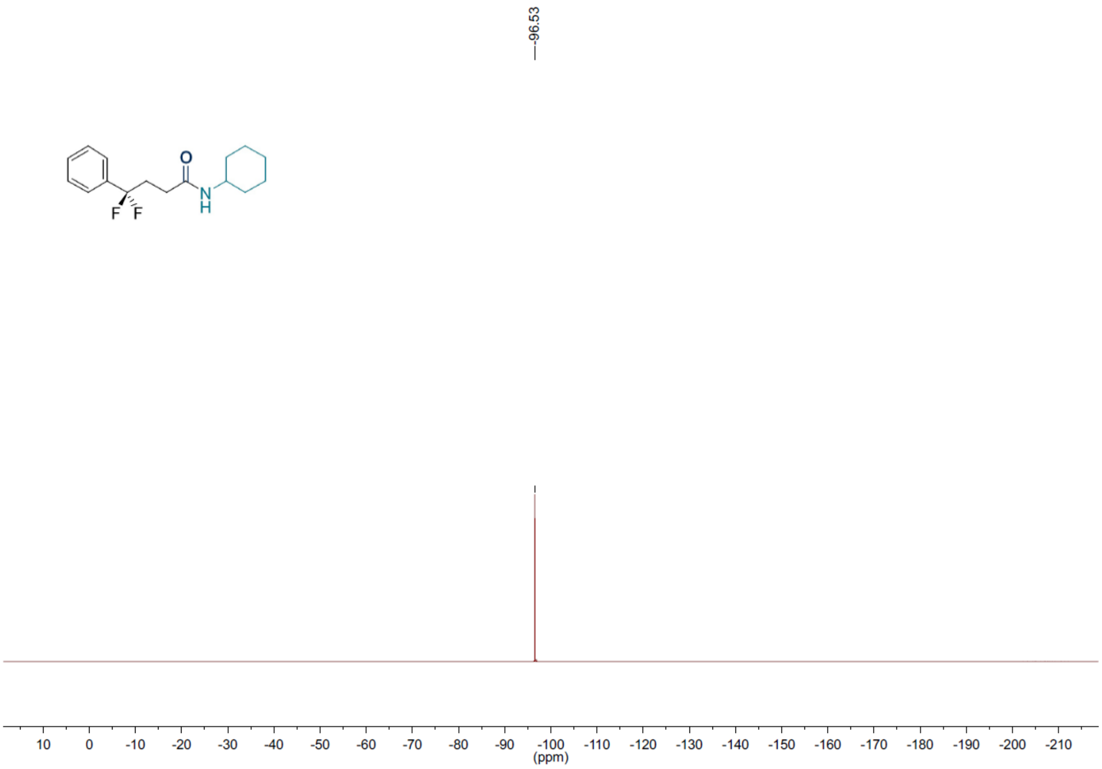

^{19}F NMR spectrum of **4g** in $CDCl_3$ (376 MHz)

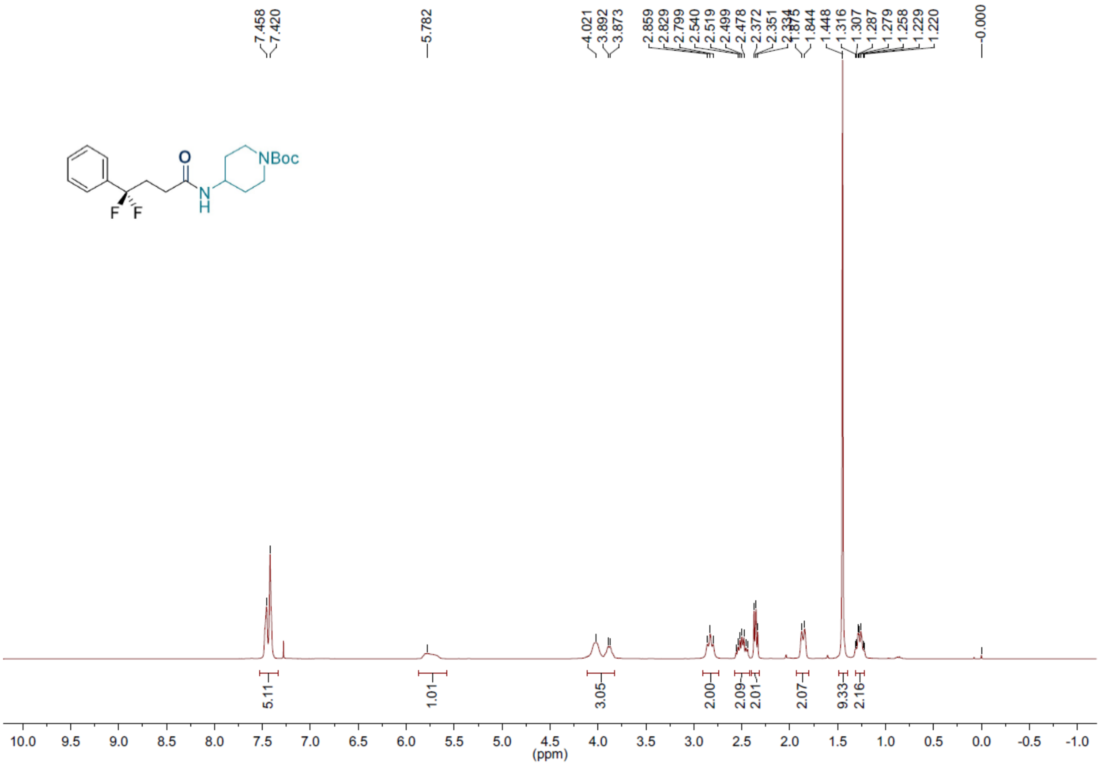

^{1}H NMR spectrum of **4h** in $CDCl_3$ (400 MHz)

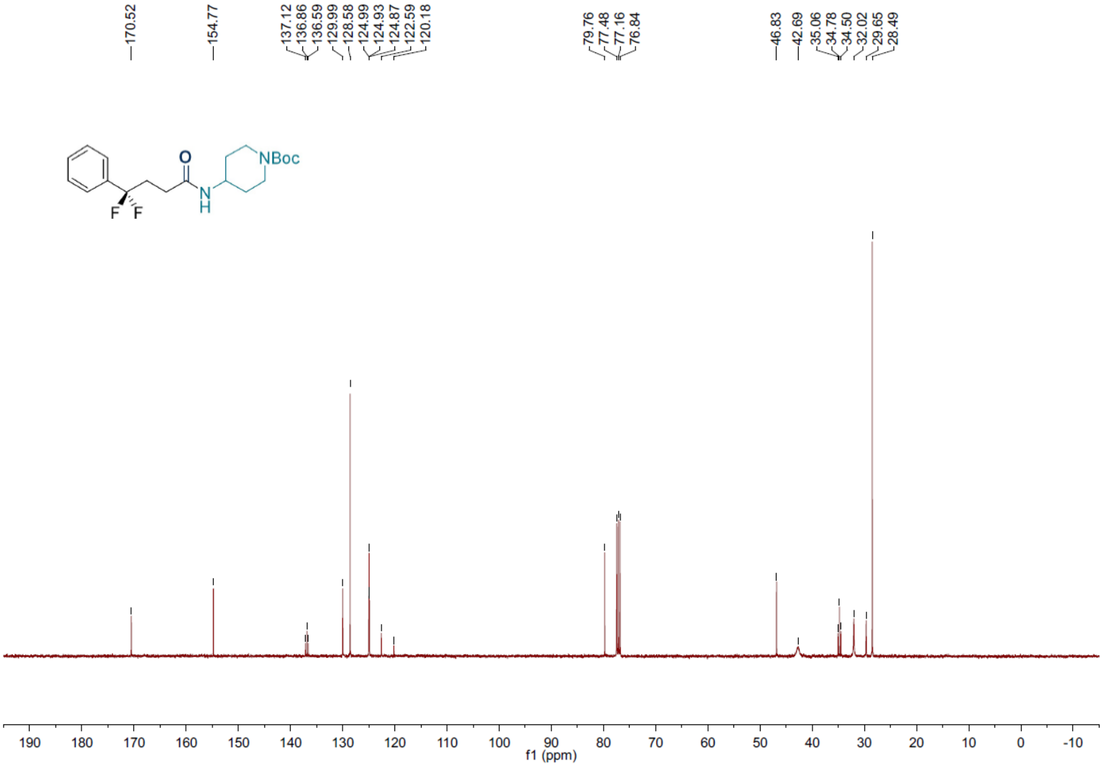

^{13}C NMR spectrum of **4h** in $CDCl_3$ (101 MHz)

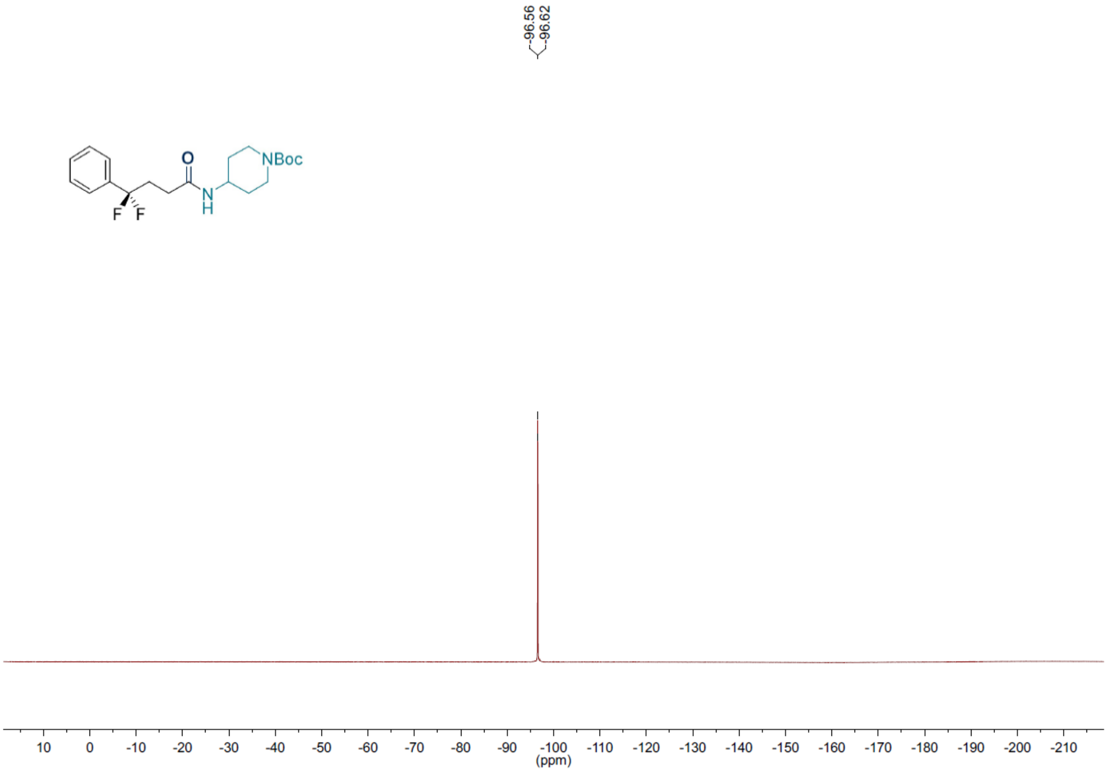

^{19}F NMR spectrum of **4h** in $CDCl_3$ (376 MHz)

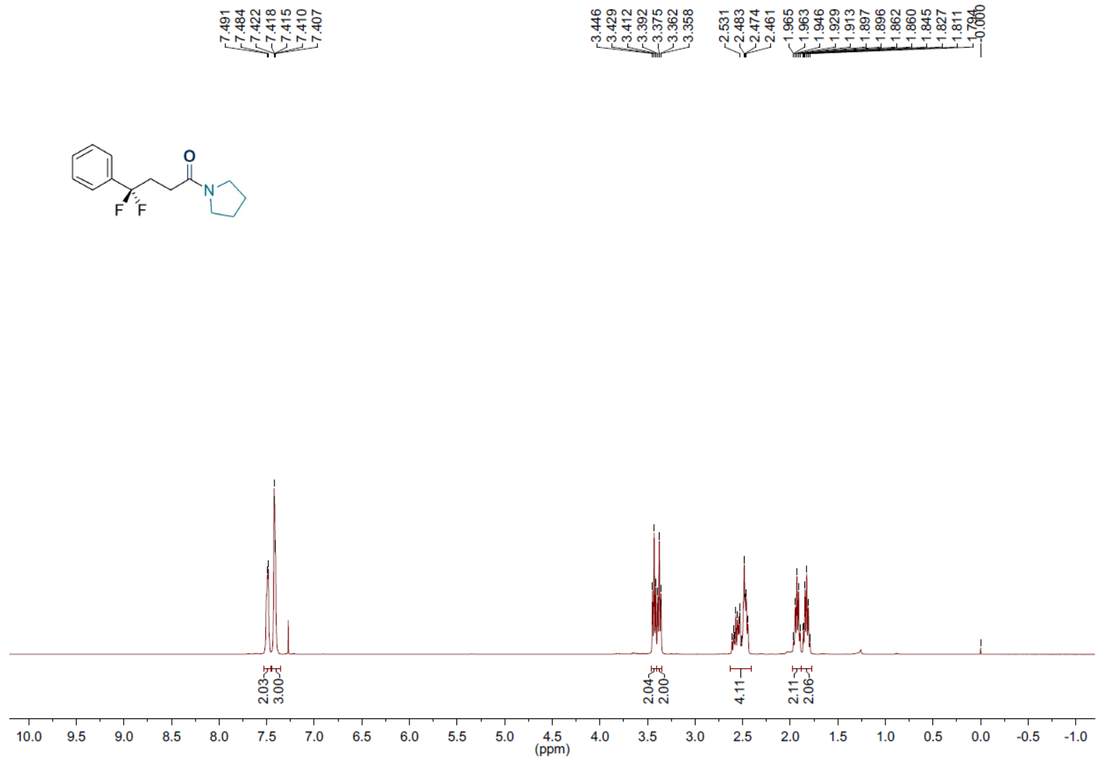

^{1}H NMR spectrum of **4i** in $CDCl_3$ (400 MHz)

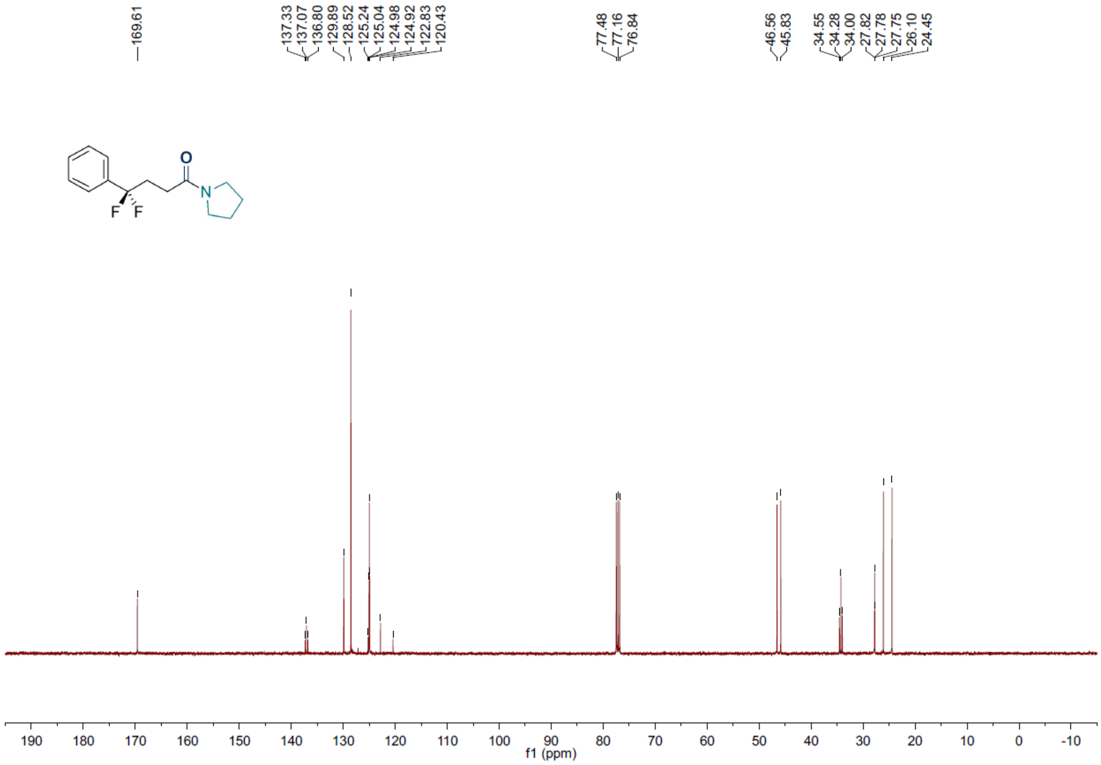

^{13}C NMR spectrum of **4i** in $CDCl_3$ (101 MHz)

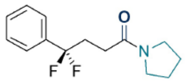

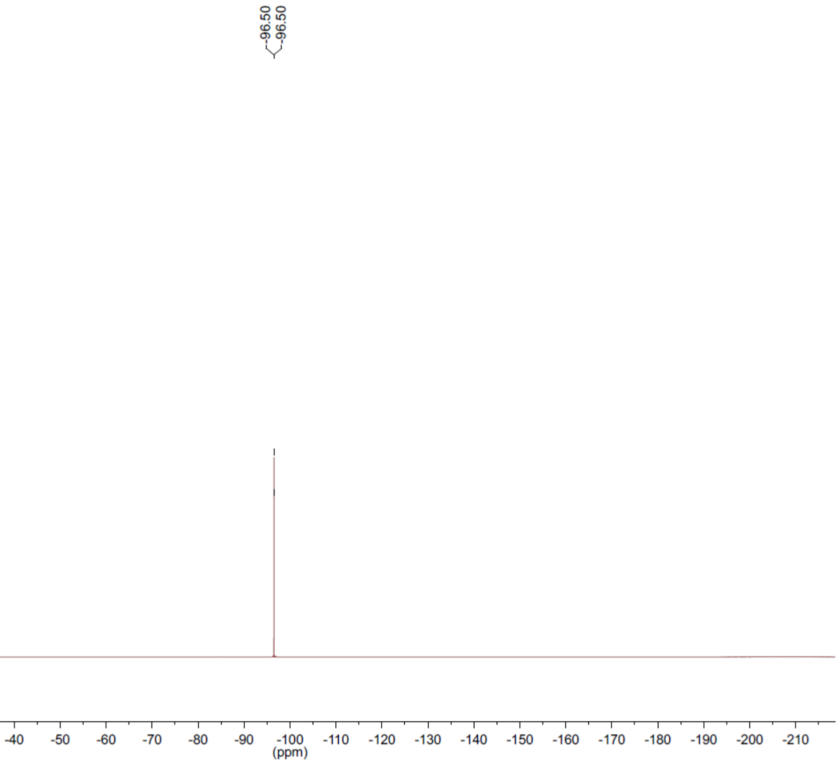

^{19}F NMR spectrum of **4i** in $CDCl_3$ (376 MHz)

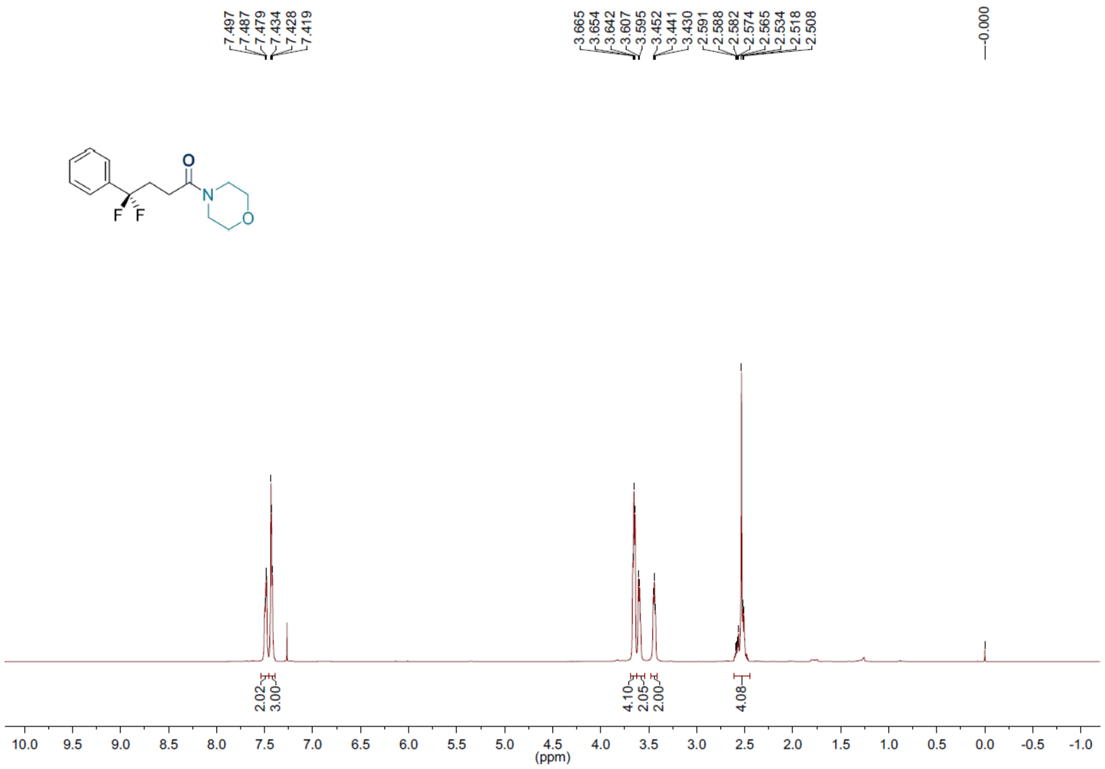

^{1}H NMR spectrum of **4j** in $CDCl_3$ (400 MHz)

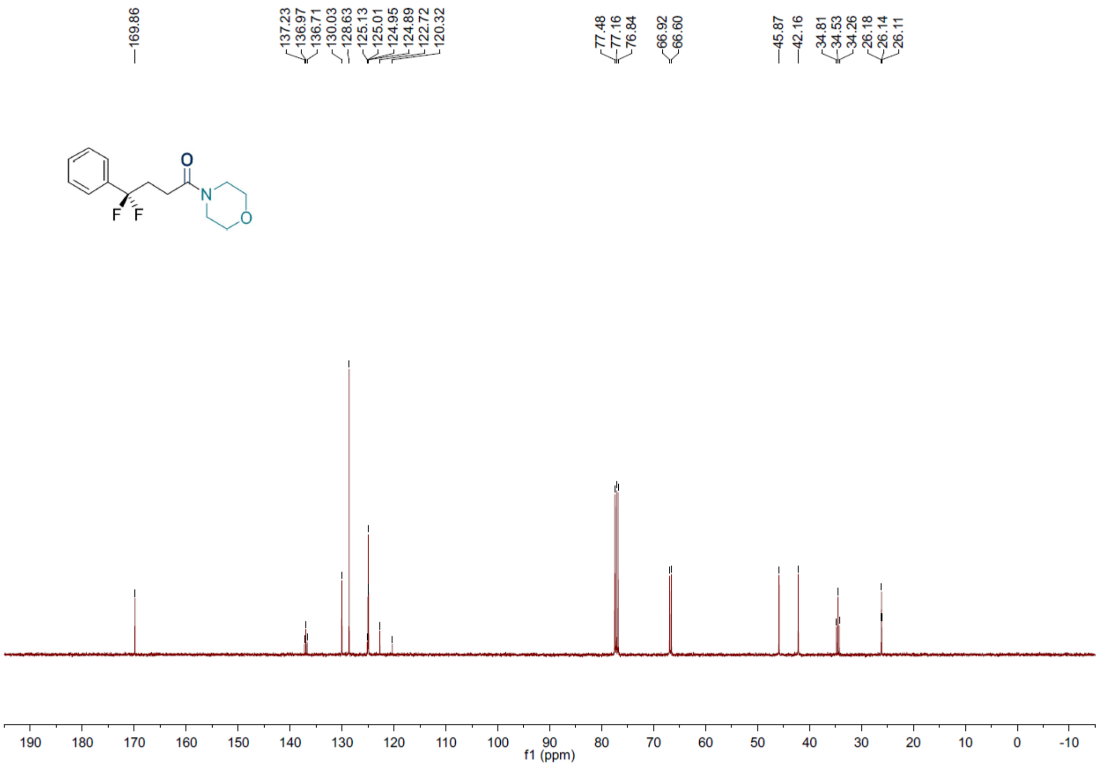

^{13}C NMR spectrum of **4j** in $CDCl_3$ (101 MHz)

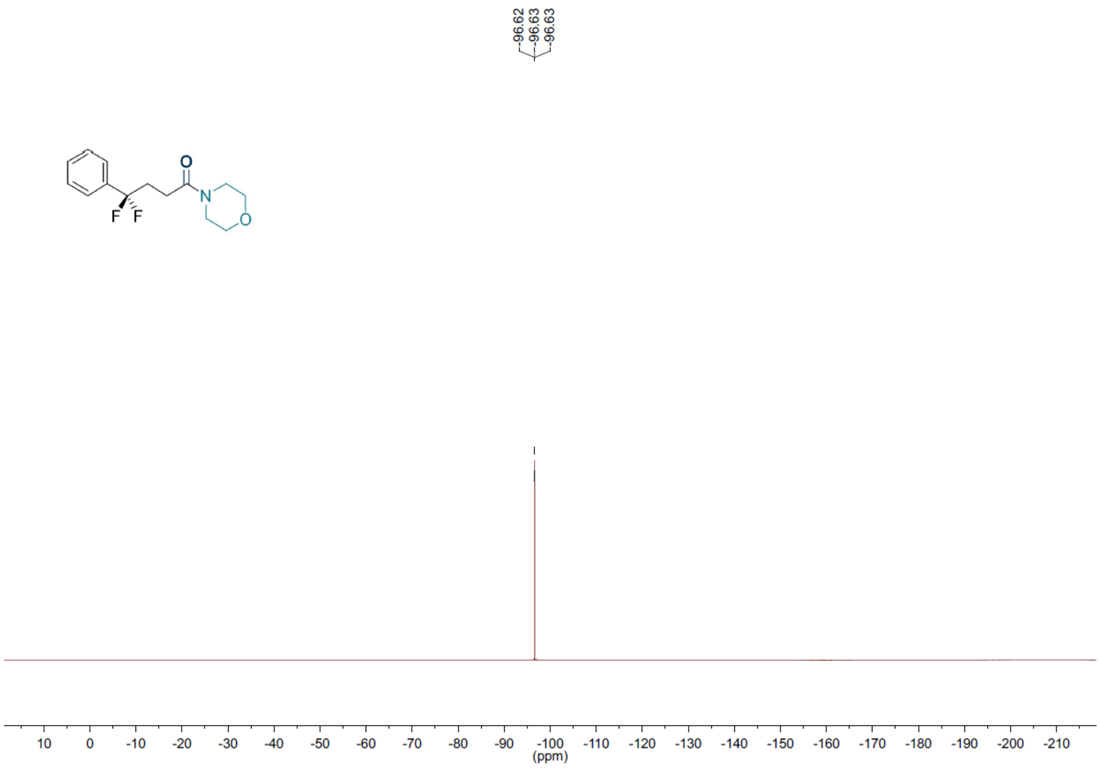

^{19}F NMR spectrum of **4j** in $CDCl_3$ (376 MHz)

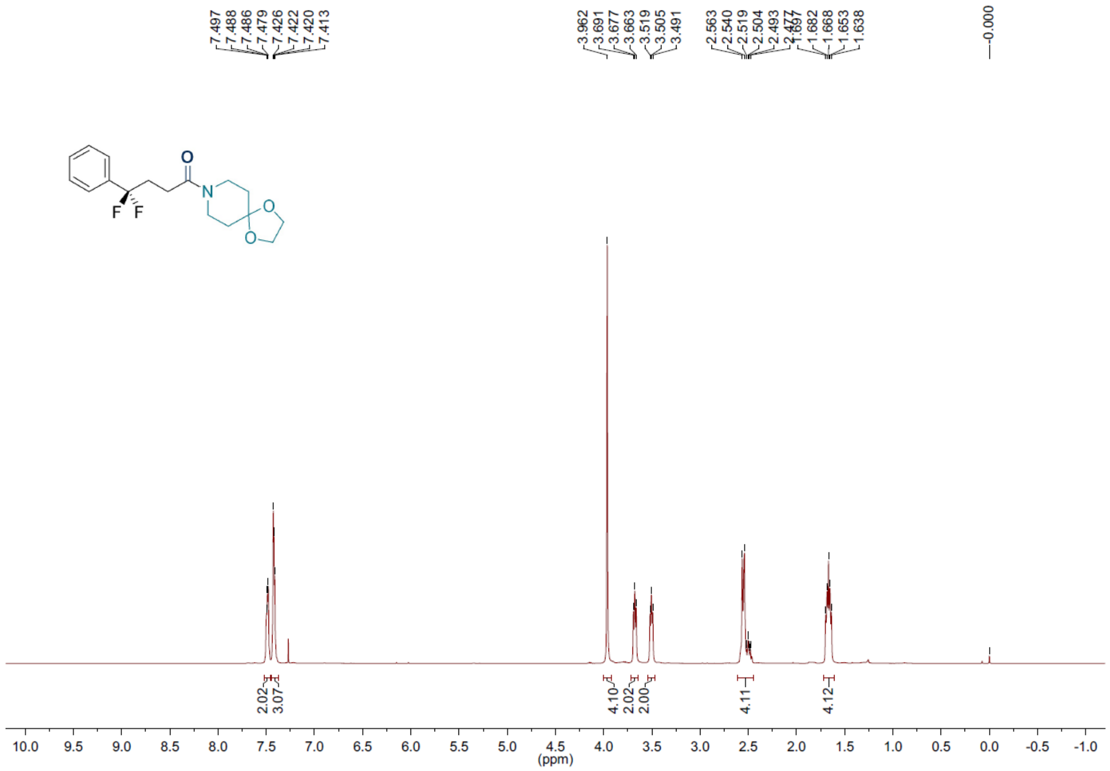

^{1}H NMR spectrum of **4k** in $CDCl_3$ (400 MHz)

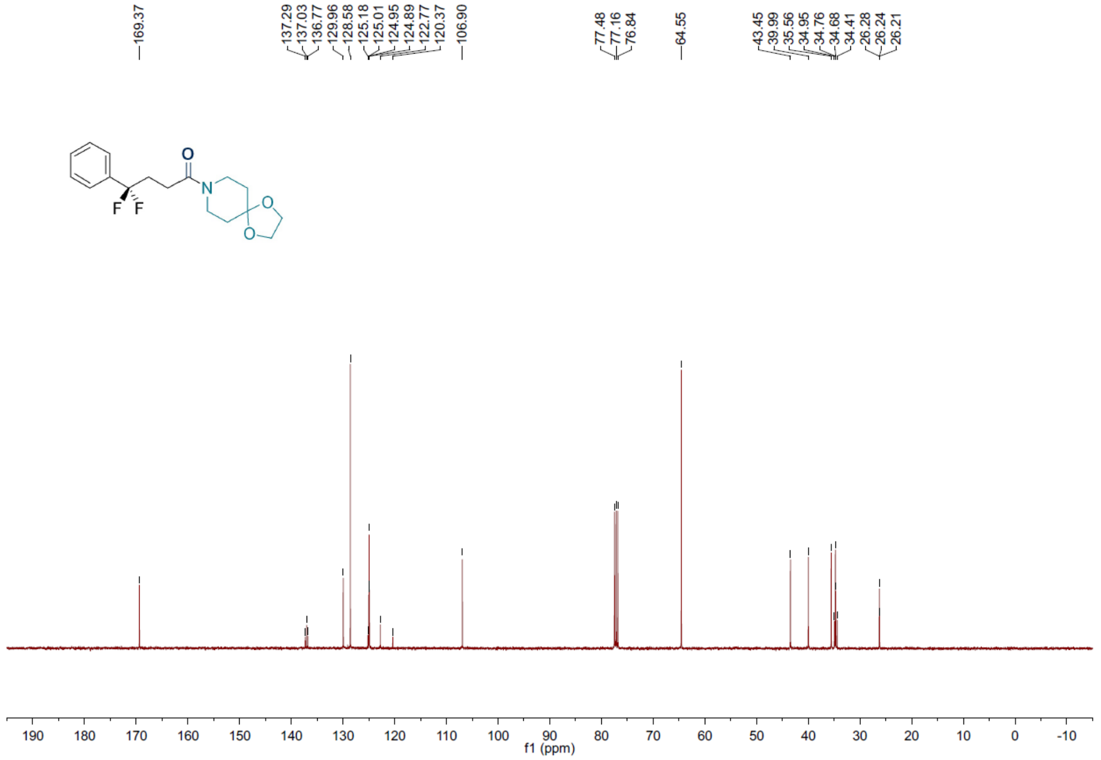

^{13}C NMR spectrum of **4k** in $CDCl_3$ (101 MHz)

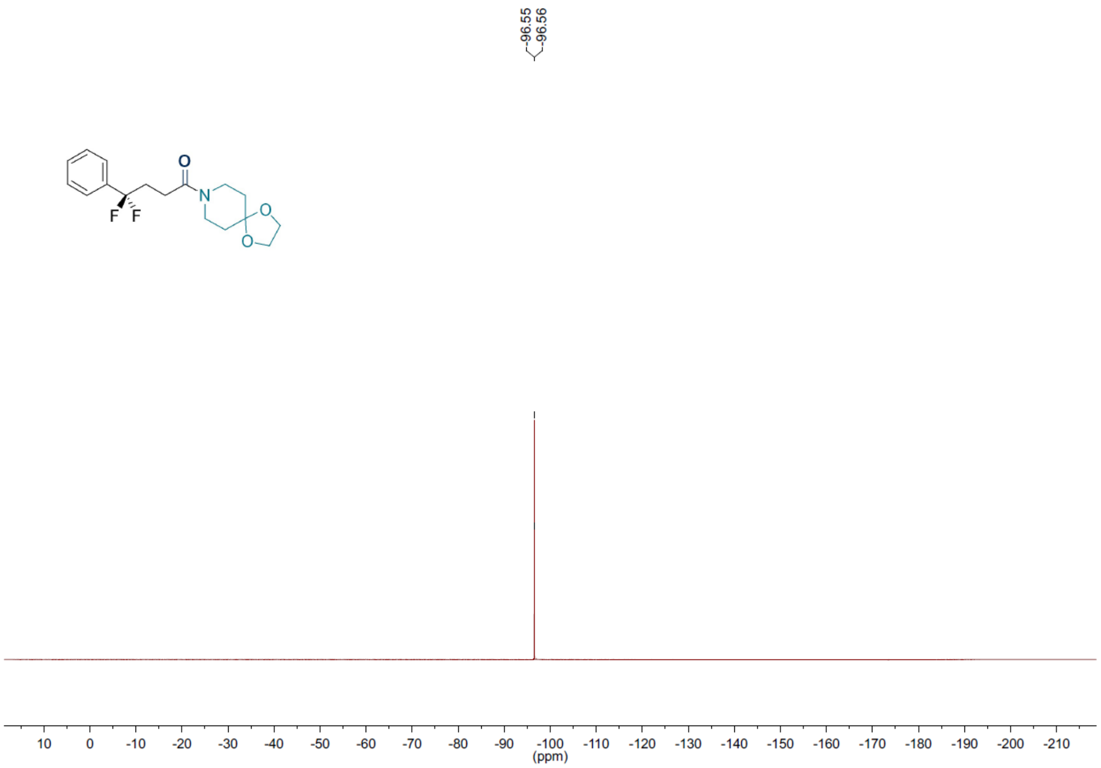

^{19}F NMR spectrum of **4k** in $CDCl_3$ (376 MHz)

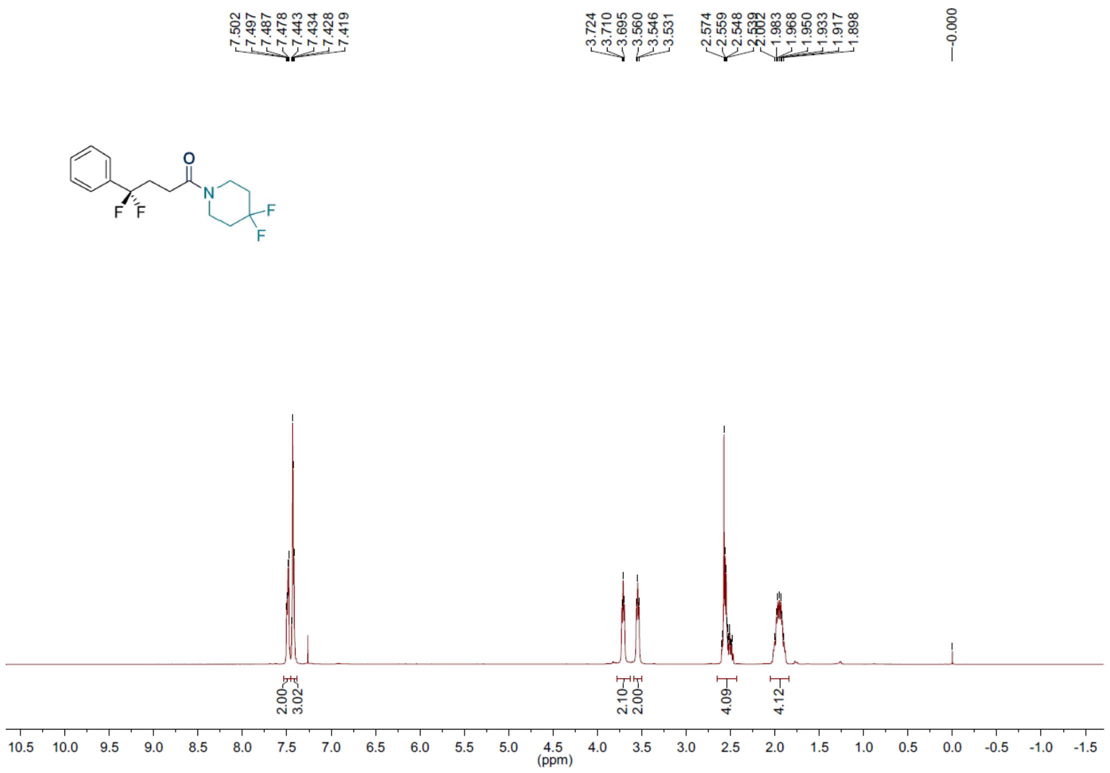

^{1}H NMR spectrum of **4l** in $CDCl_3$ (400 MHz)

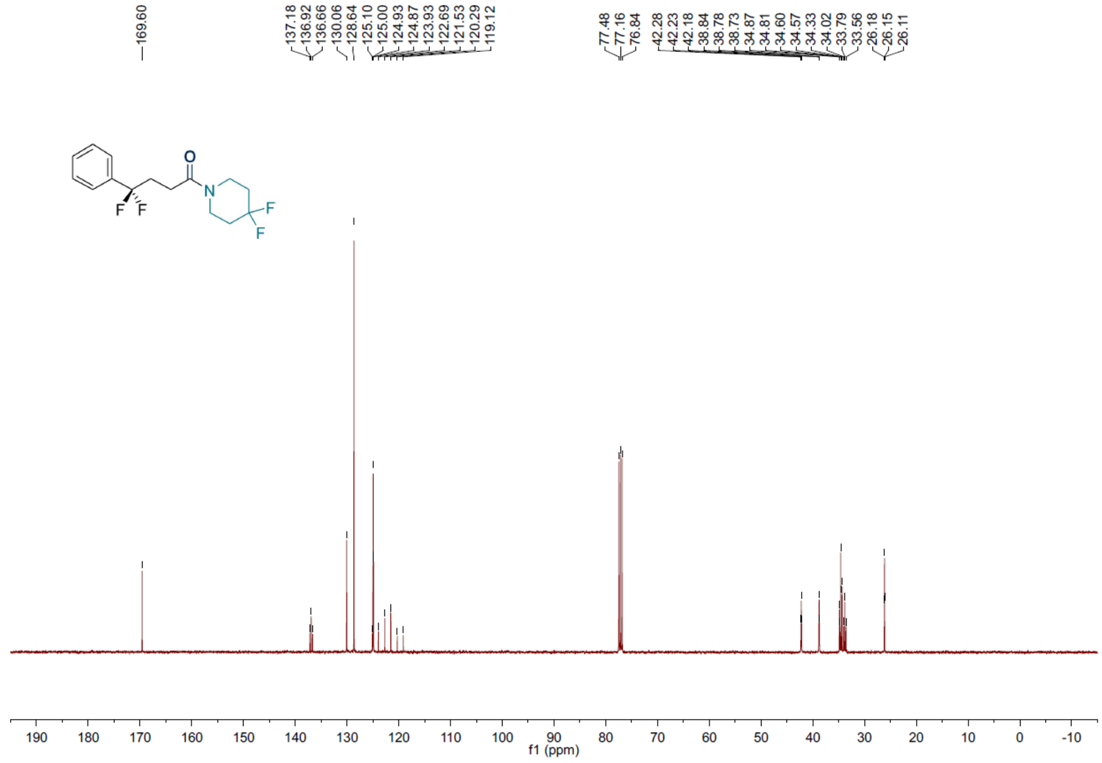

^{13}C NMR spectrum of **4l** in $CDCl_3$ (101 MHz)

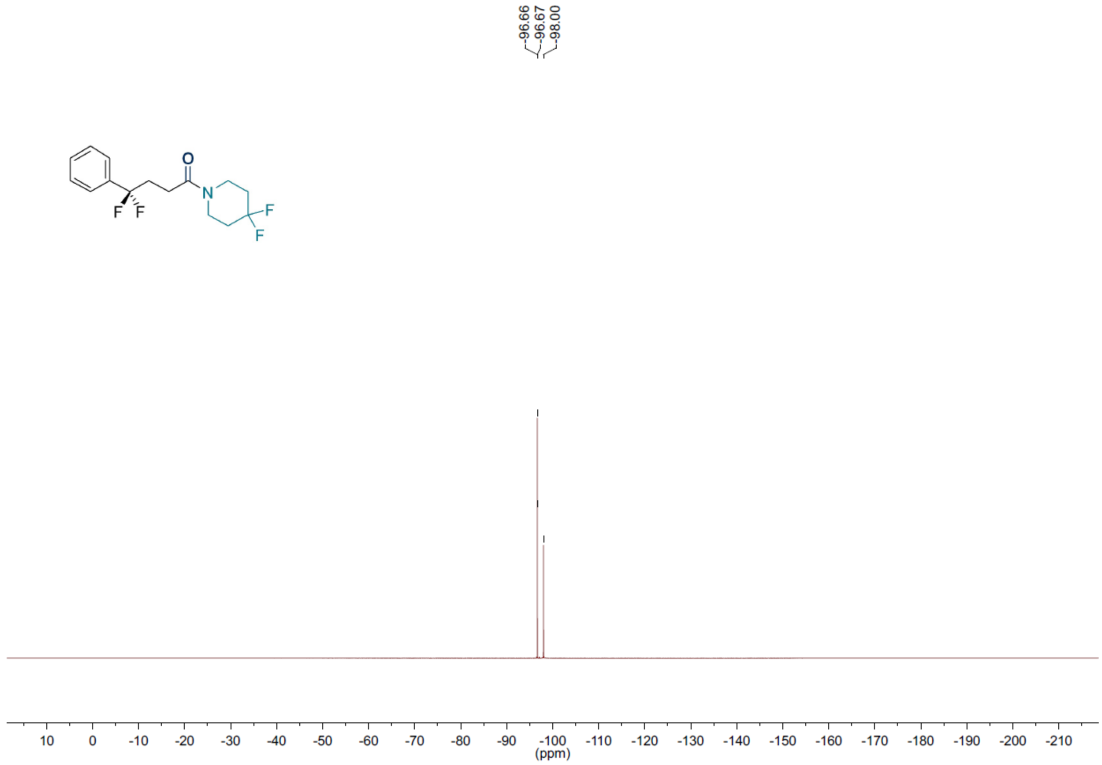

^{19}F NMR spectrum of **4l** in $CDCl_3$ (376 MHz)

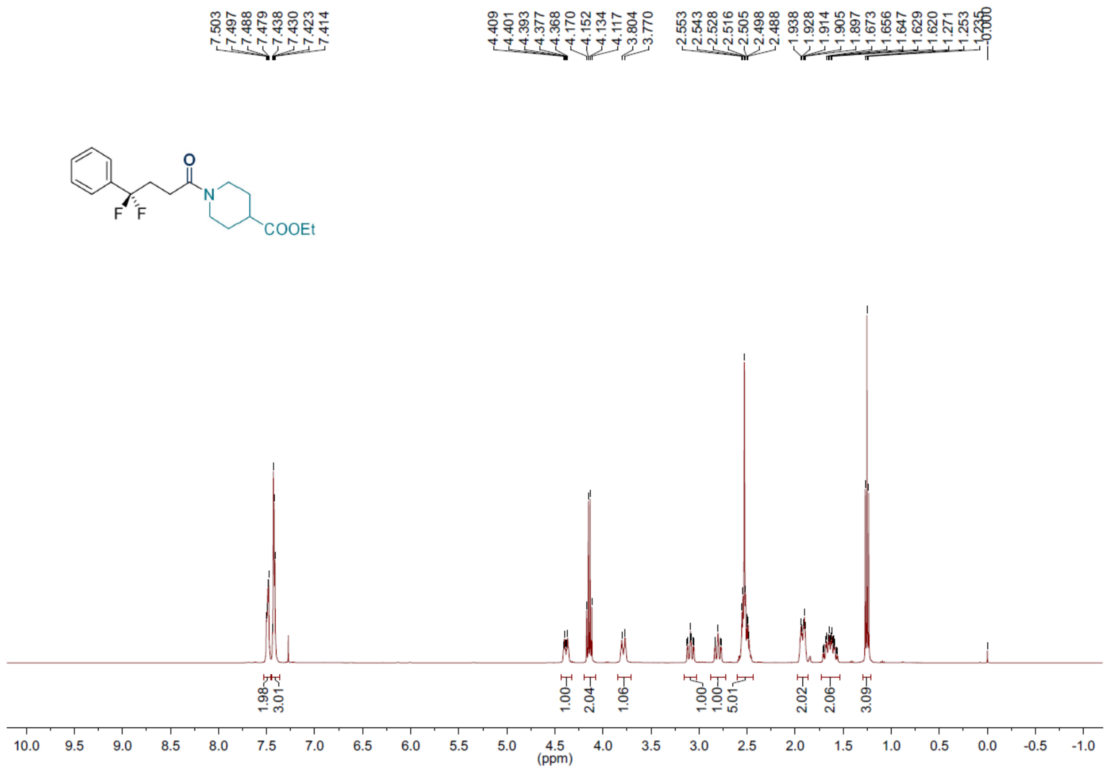

^{1}H NMR spectrum of **4m** in $CDCl_3$ (400 MHz)

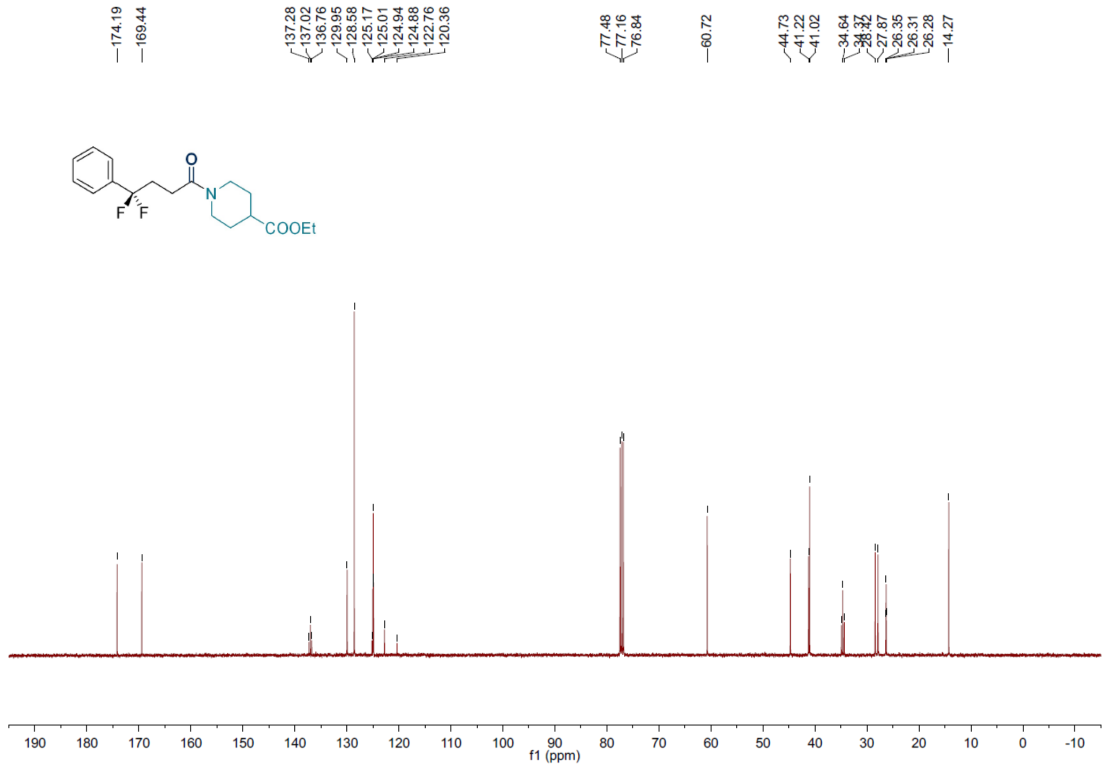

^{13}C NMR spectrum of **4m** in $CDCl_3$ (101 MHz)

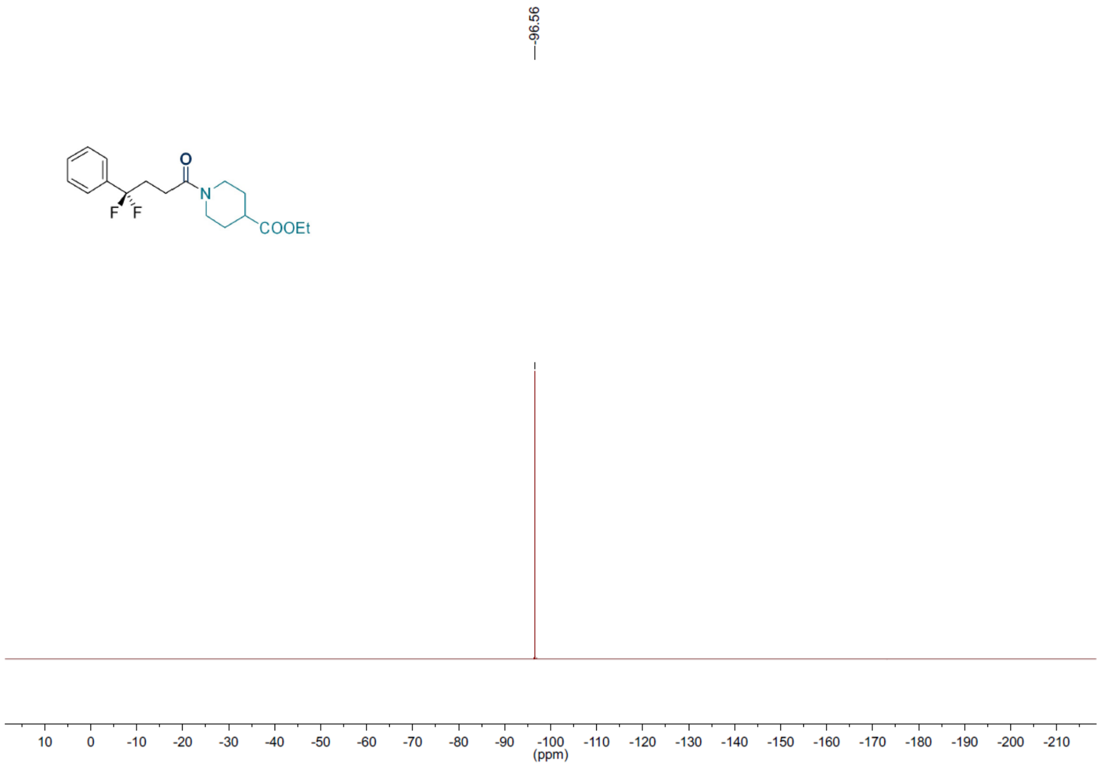

^{19}F NMR spectrum of **4m** in $CDCl_3$ (376 MHz)

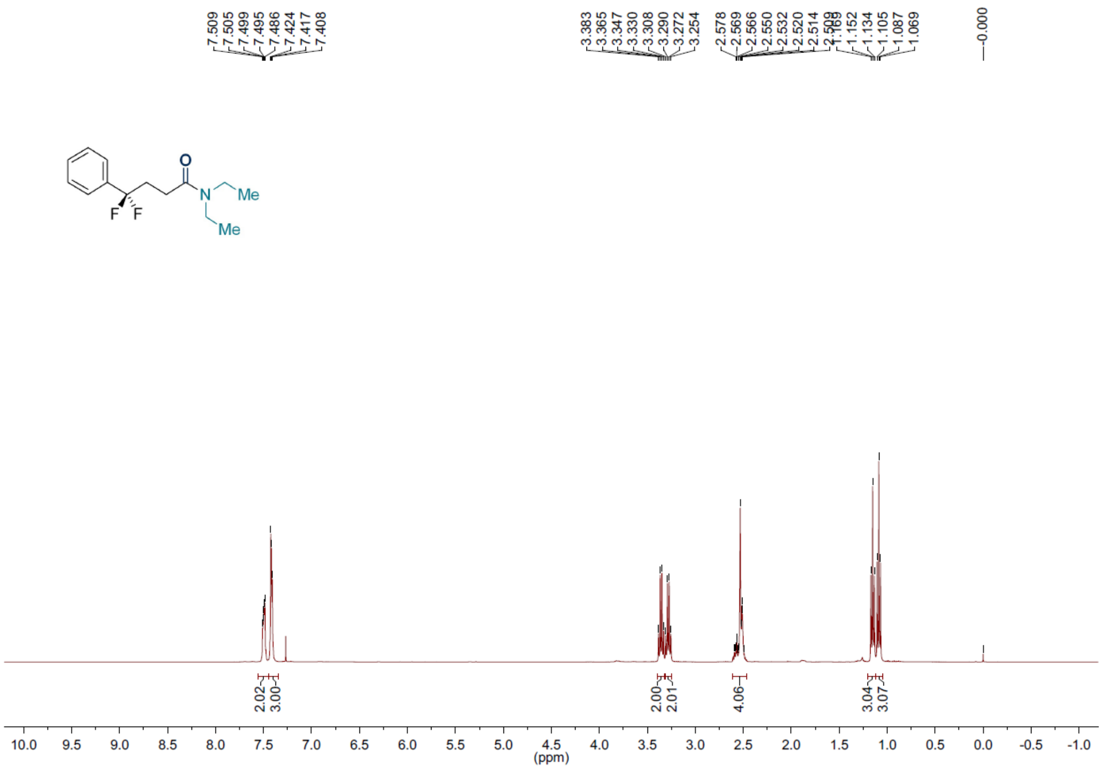

^{1}H NMR spectrum of **4n** in $CDCl_3$ (400 MHz)

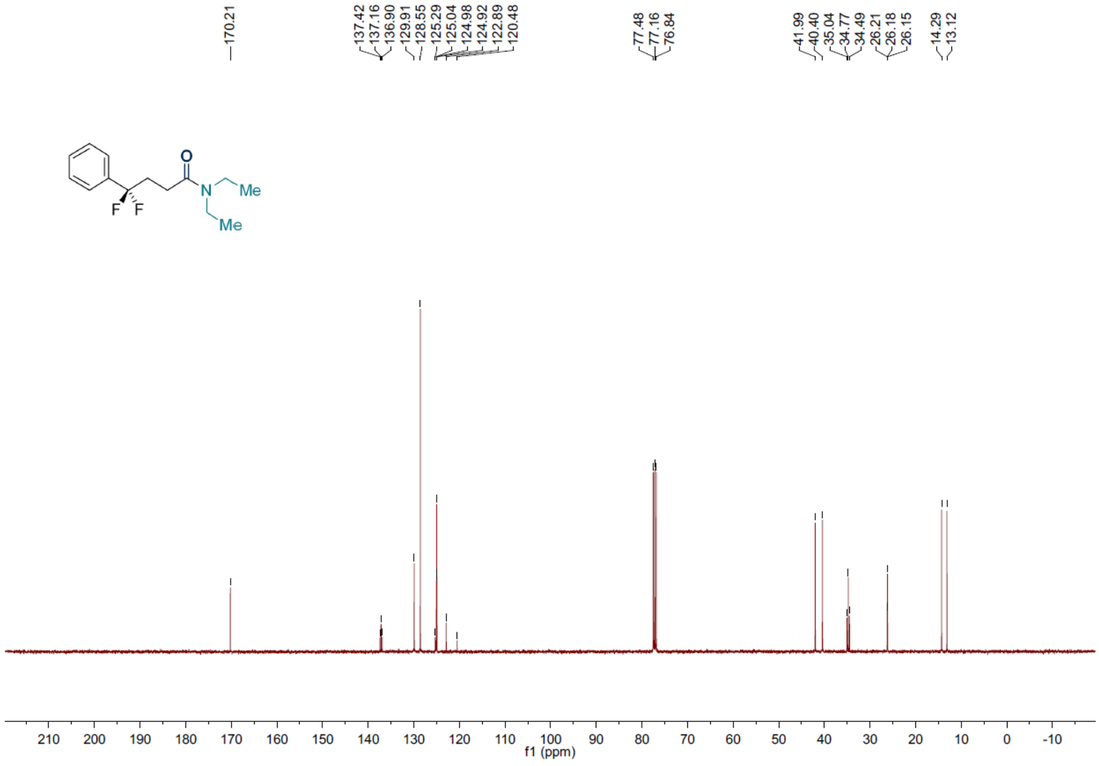

^{13}C NMR spectrum of **4n** in $CDCl_3$ (101 MHz)

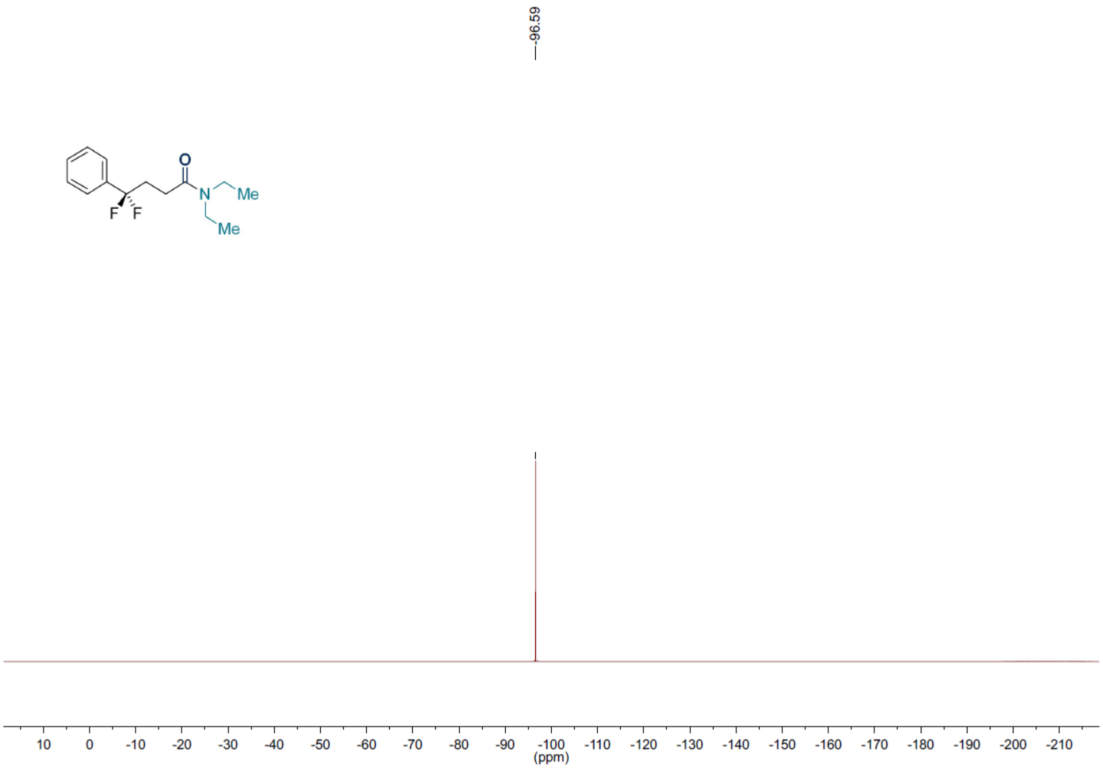

^{19}F NMR spectrum of **4n** in $CDCl_3$ (376 MHz)

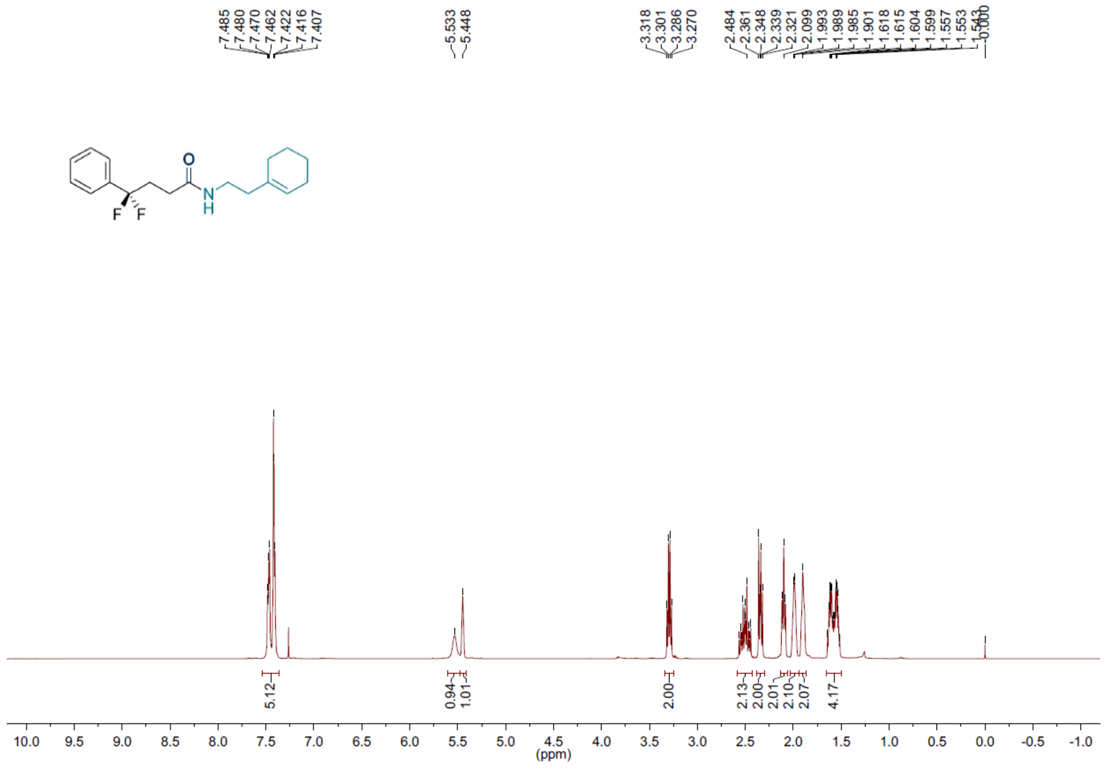

^{1}H NMR spectrum of **4o** in $CDCl_3$ (400 MHz)

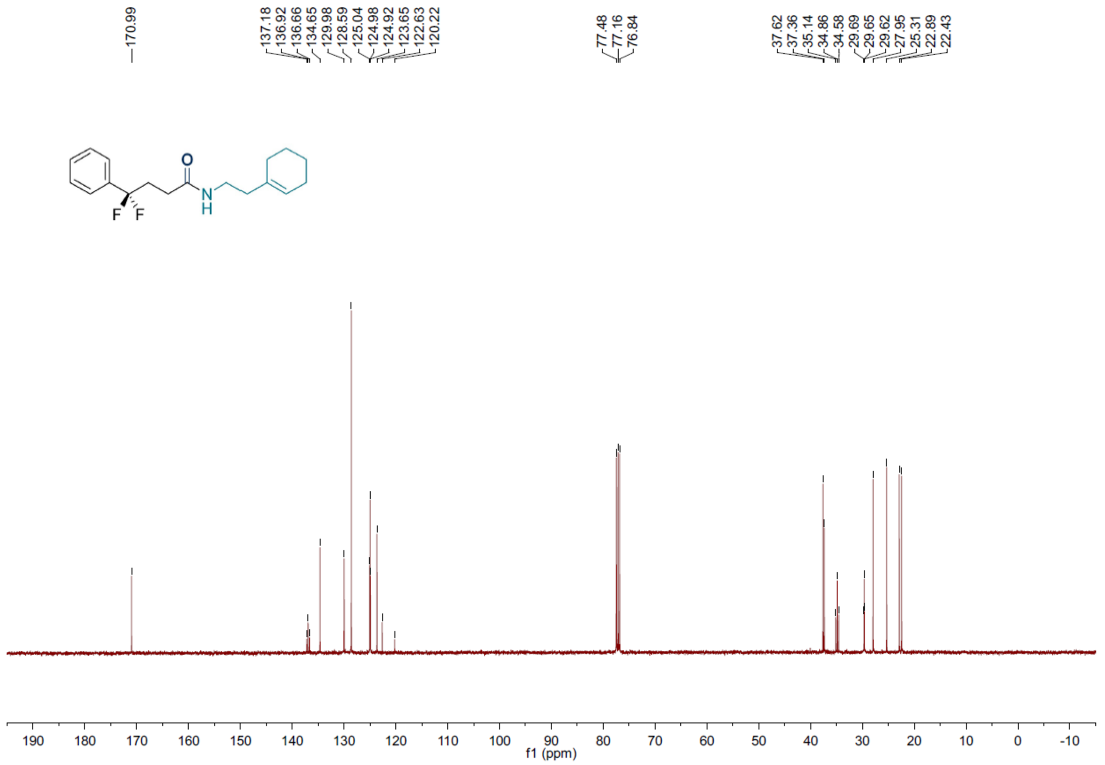

^{13}C NMR spectrum of **4o** in $CDCl_3$ (101 MHz)

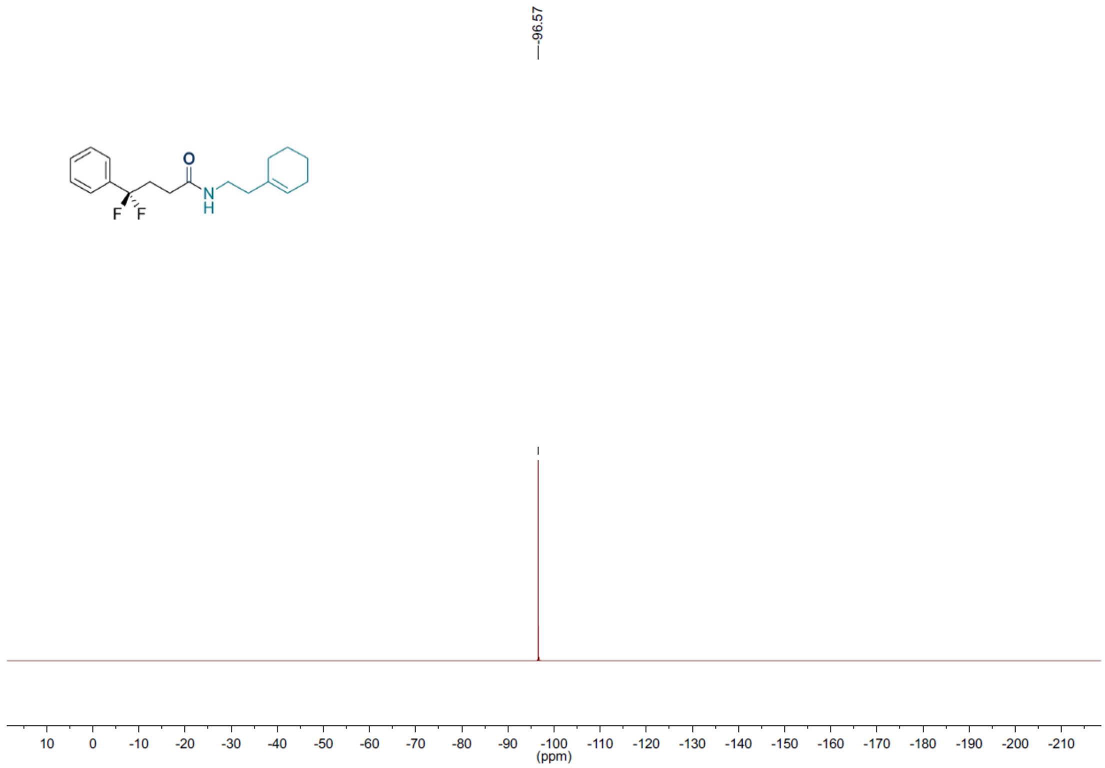

^{19}F NMR spectrum of **4o** in $CDCl_3$ (376 MHz)

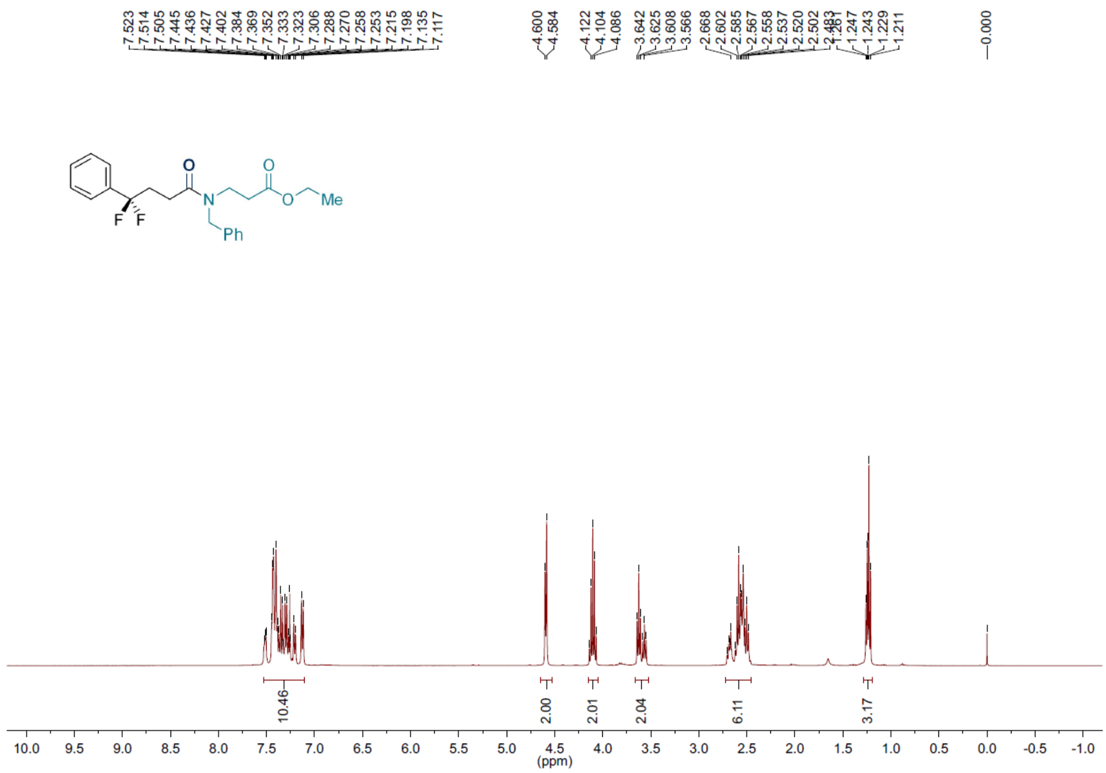

^{1}H NMR spectrum of **4p** in $CDCl_3$ (400 MHz)

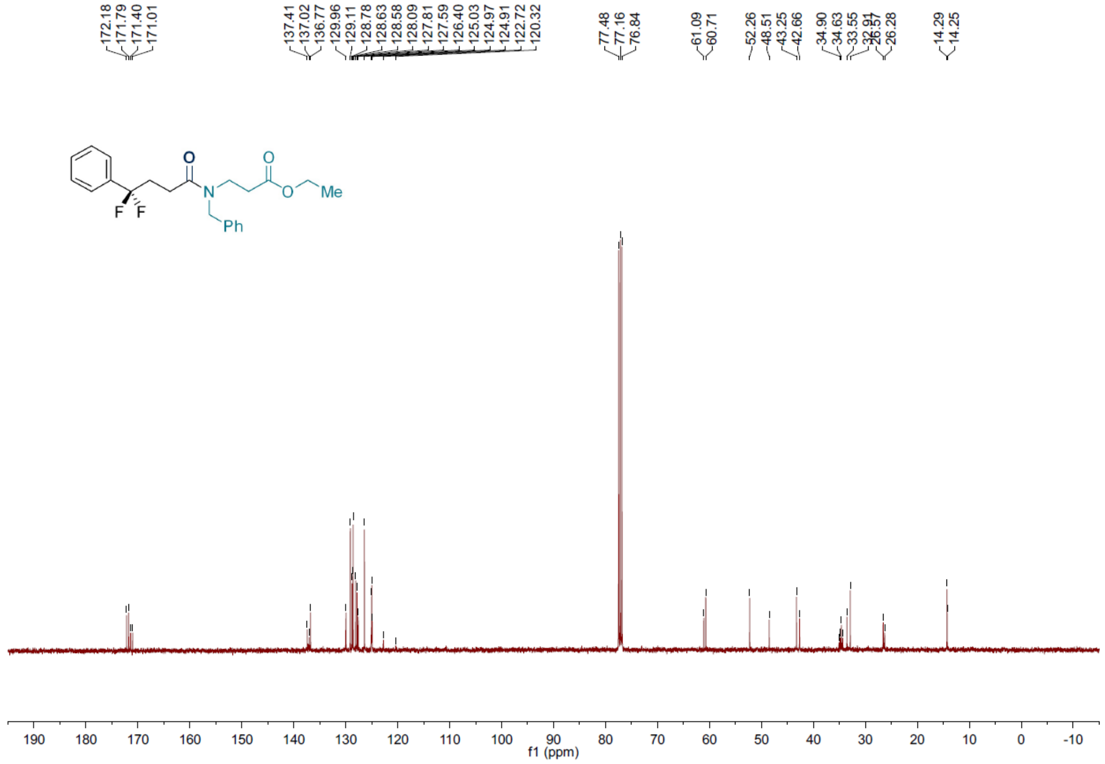

^{13}C NMR spectrum of **4p** in $CDCl_3$ (101 MHz)

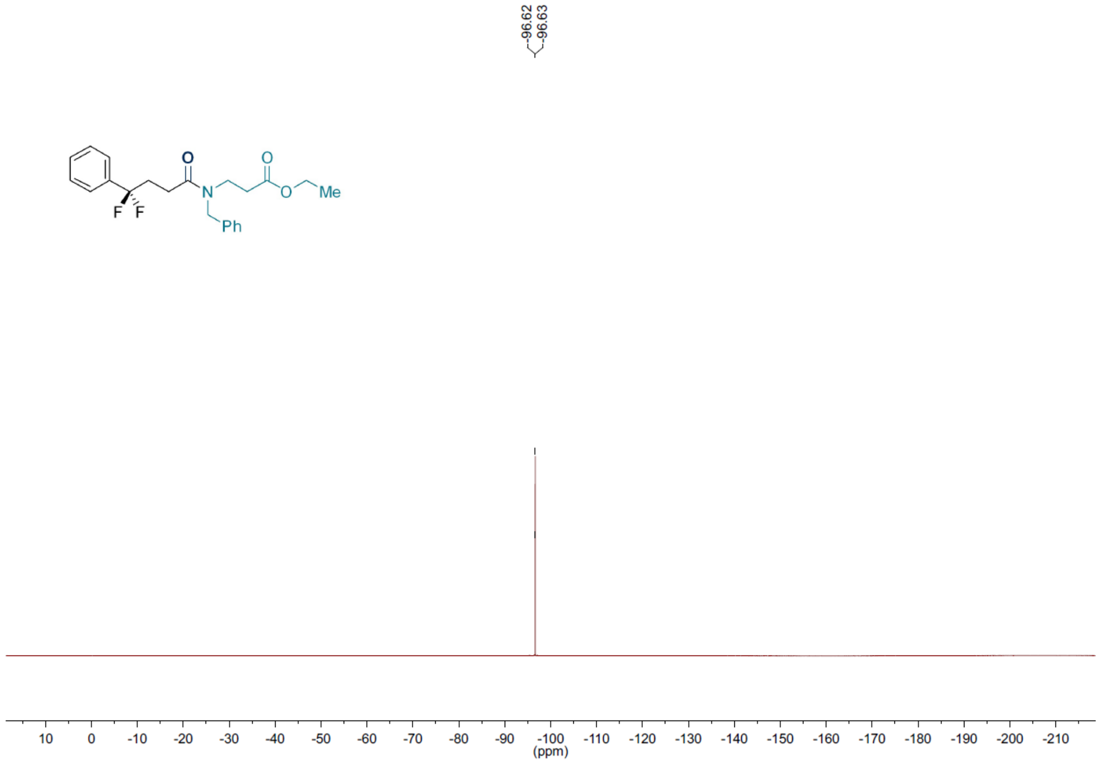

^{19}F NMR spectrum of **4p** in $CDCl_3$ (376 MHz)

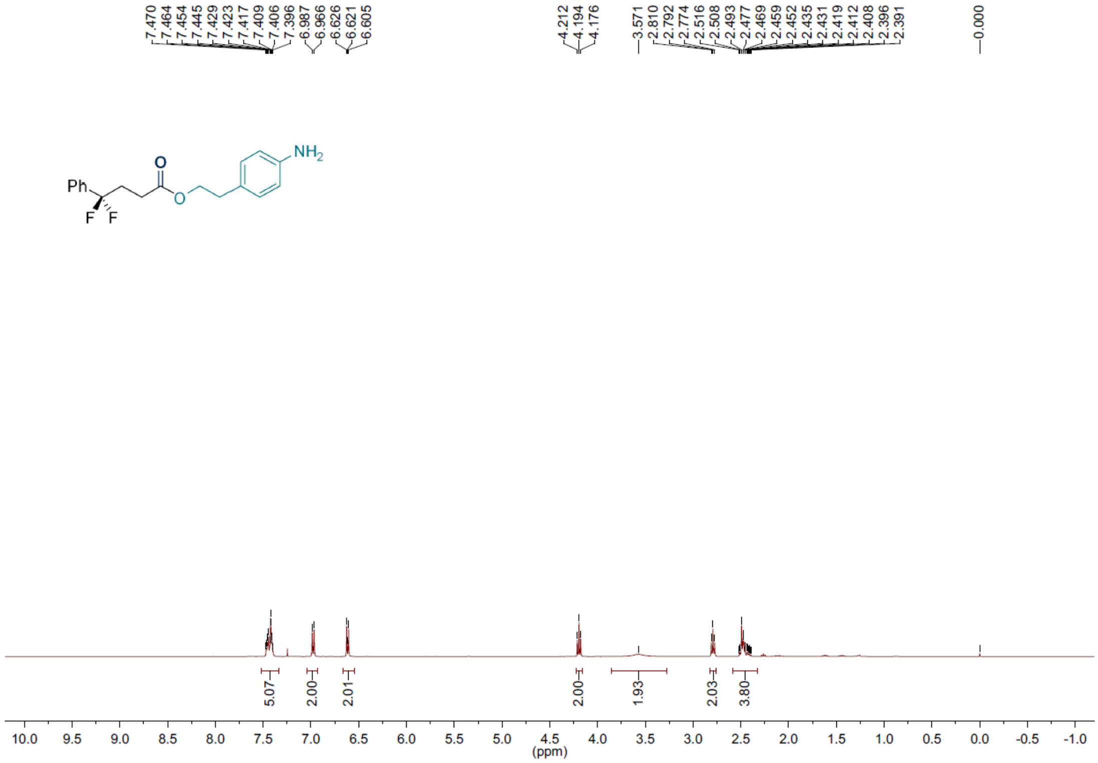

^{1}H NMR spectrum of **4q** in $CDCl_3$ (400 MHz)

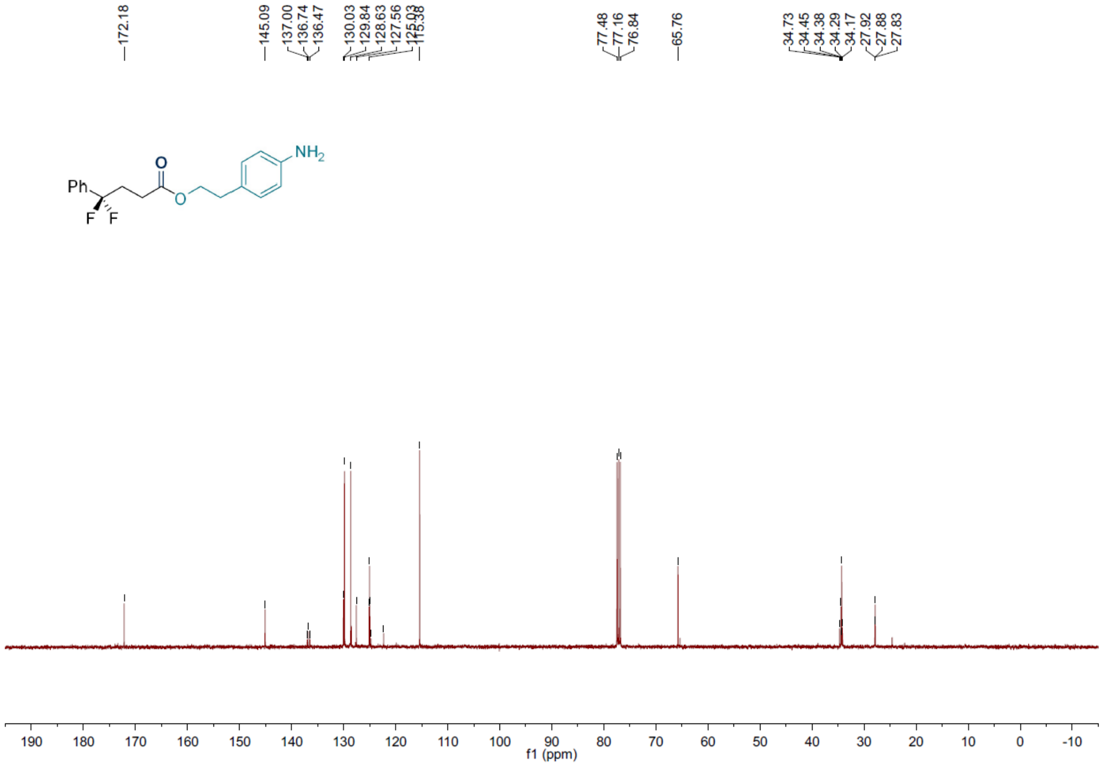

^{13}C NMR spectrum of **4q** in $CDCl_3$ (101 MHz)

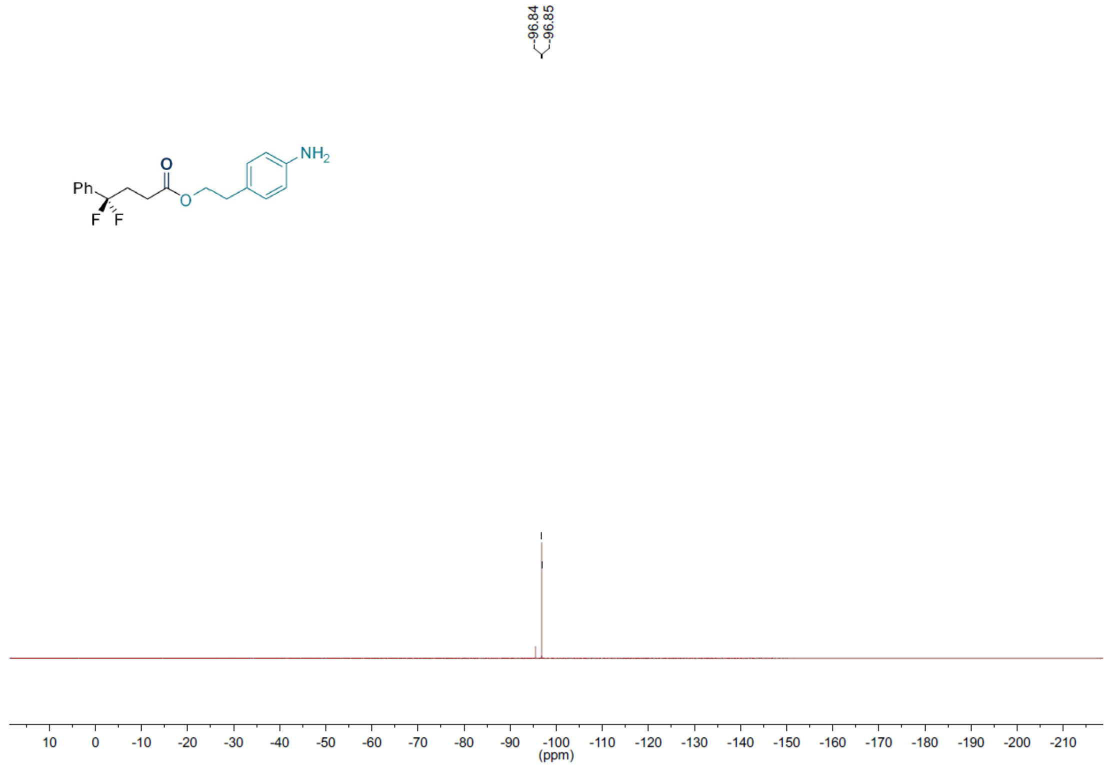

^{19}F NMR spectrum of **4q** in $CDCl_3$ (376 MHz)

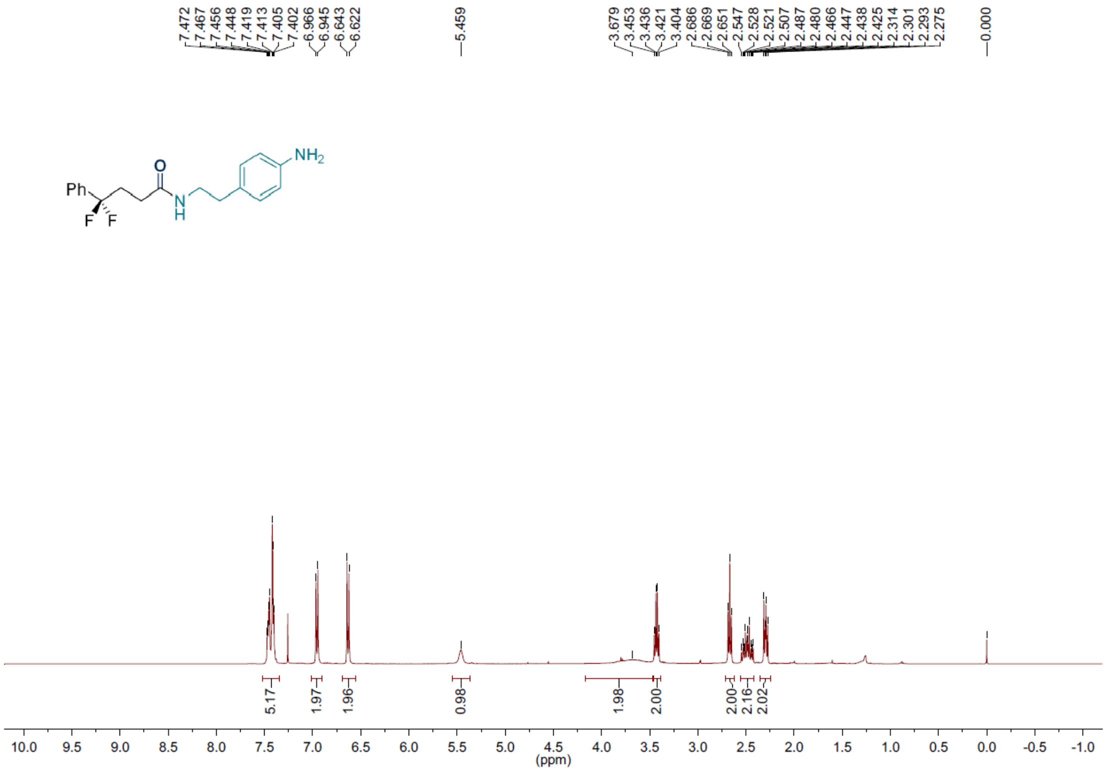

^{1}H NMR spectrum of **4r** in $CDCl_3$ (400 MHz)

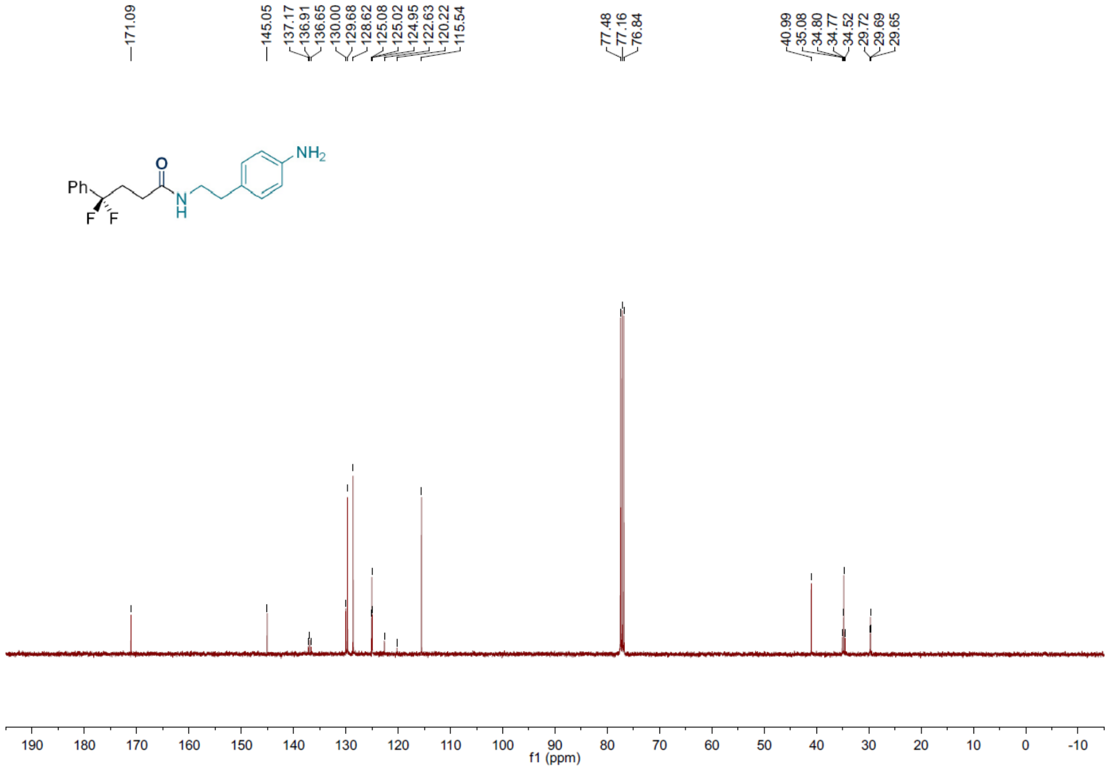

^{13}C NMR spectrum of **4r** in $CDCl_3$ (101 MHz)

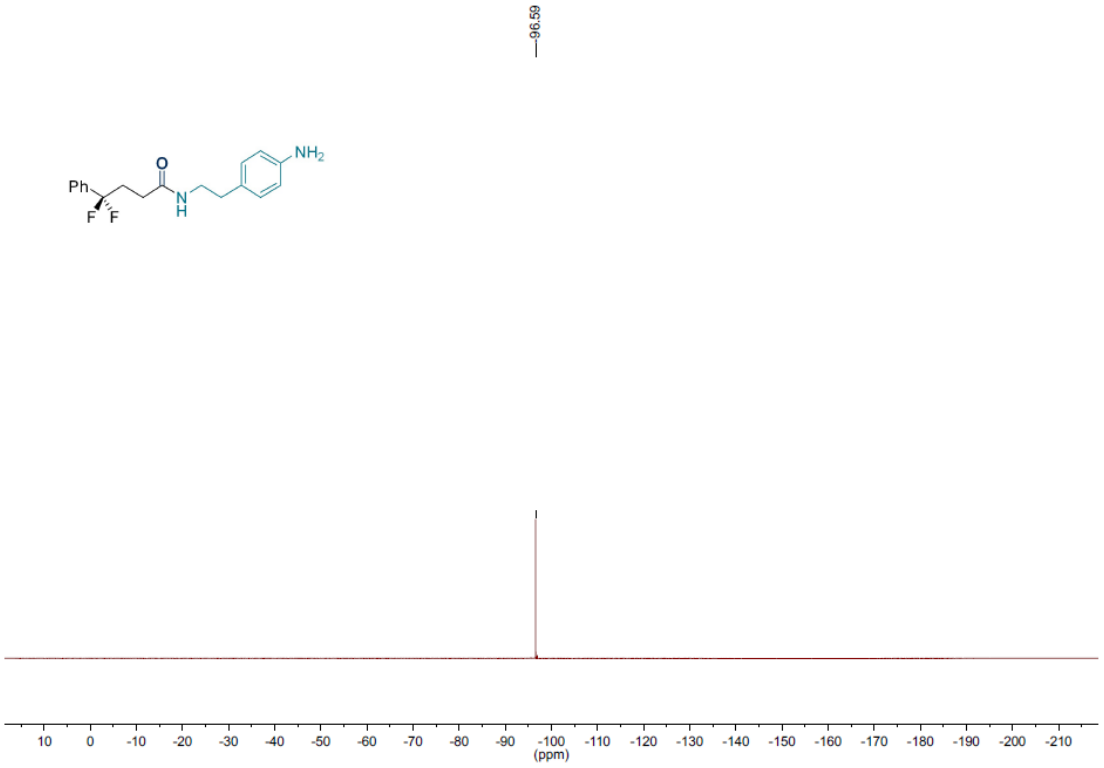

^{19}F NMR spectrum of **4r** in $CDCl_3$ (376 MHz)

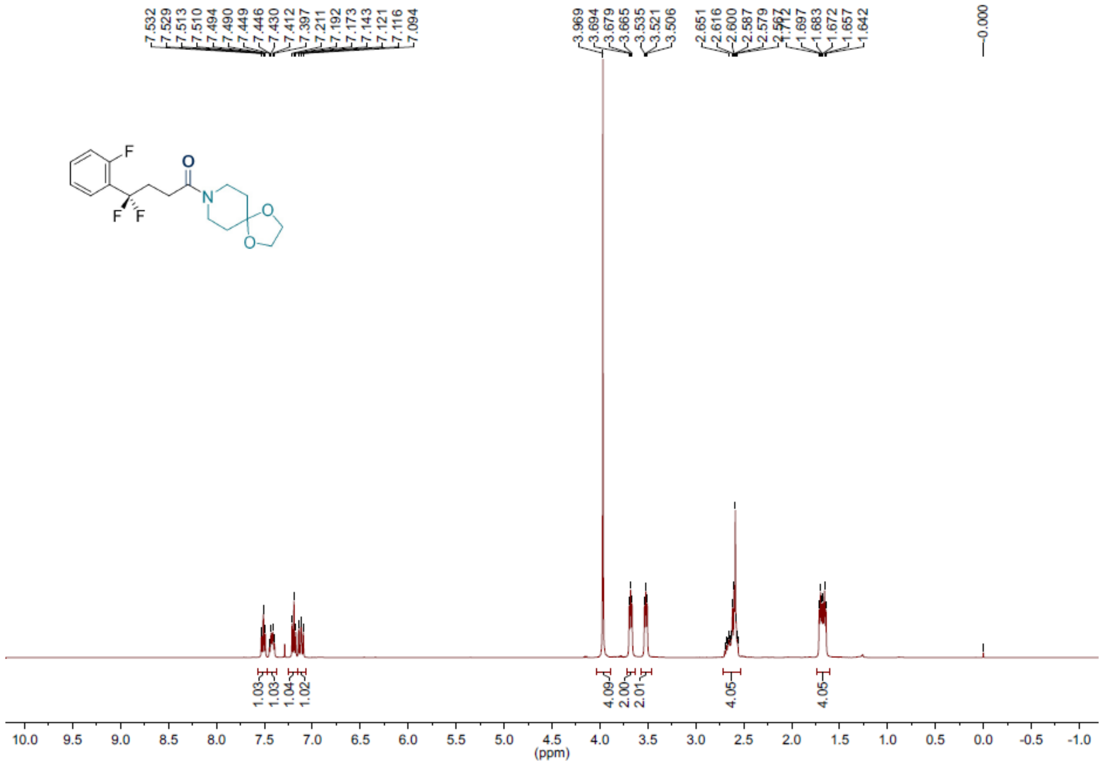

^{1}H NMR spectrum of **5a** in $CDCl_3$ (400 MHz)

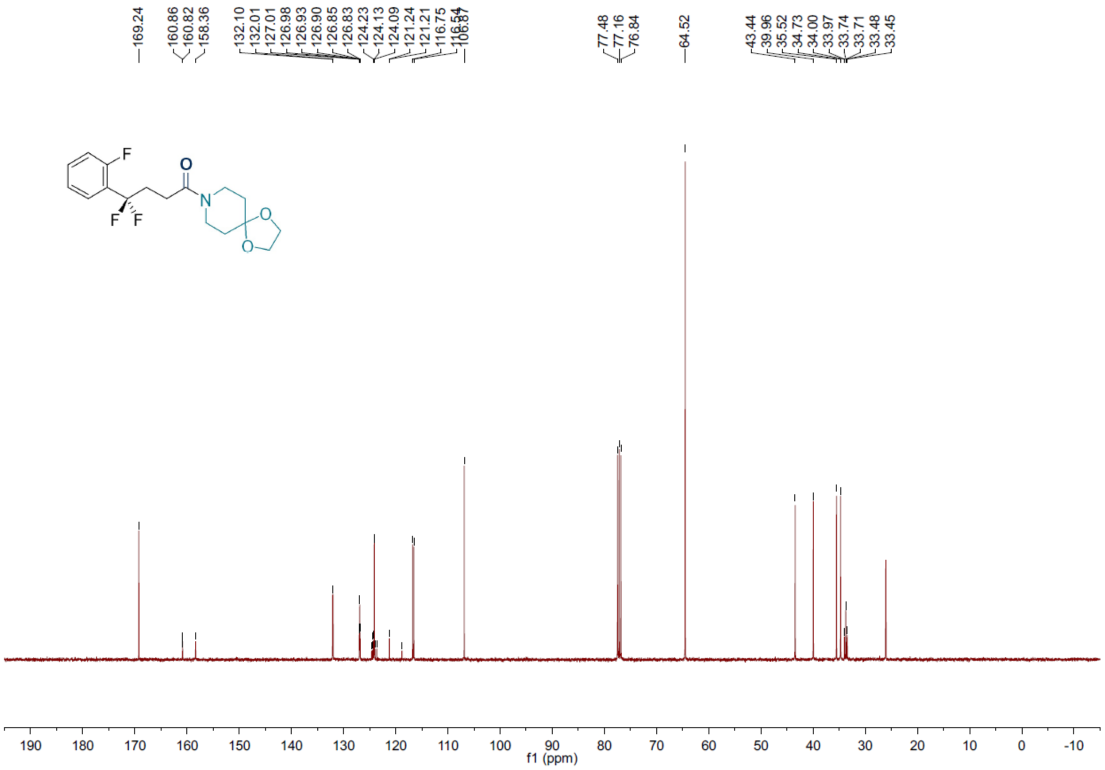

^{13}C NMR spectrum of **5a** in $CDCl_3$ (101 MHz)

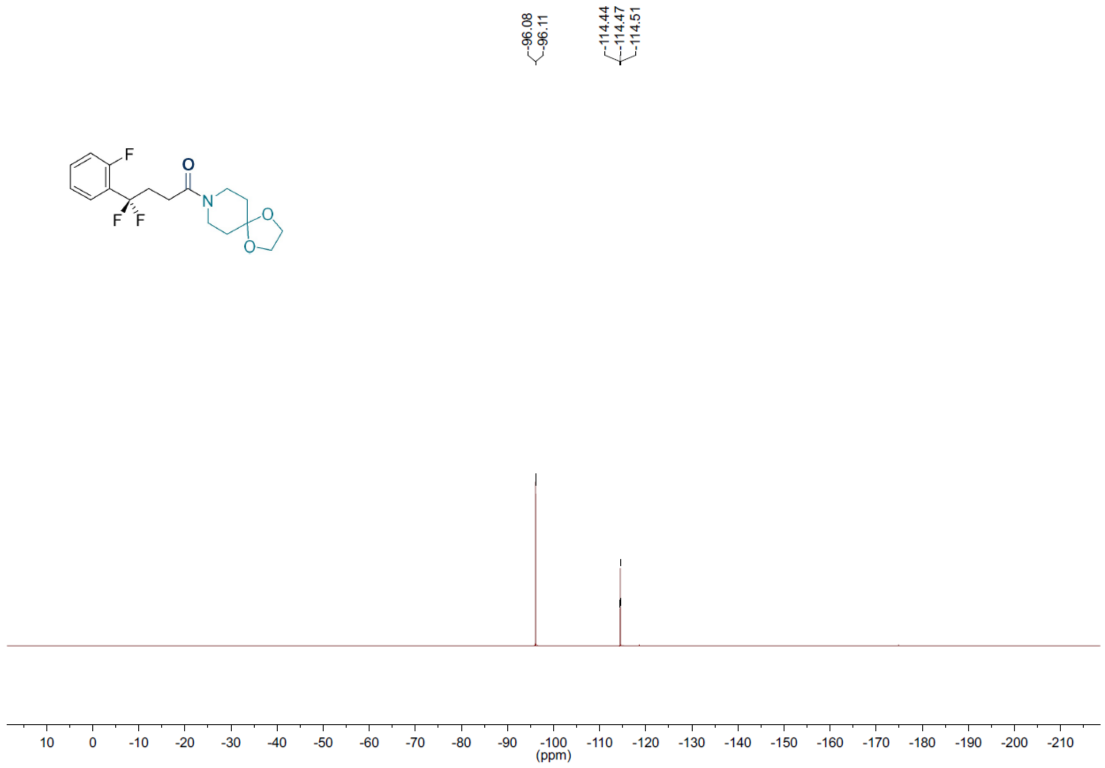

^{19}F NMR spectrum of **5a** in $CDCl_3$ (376 MHz)

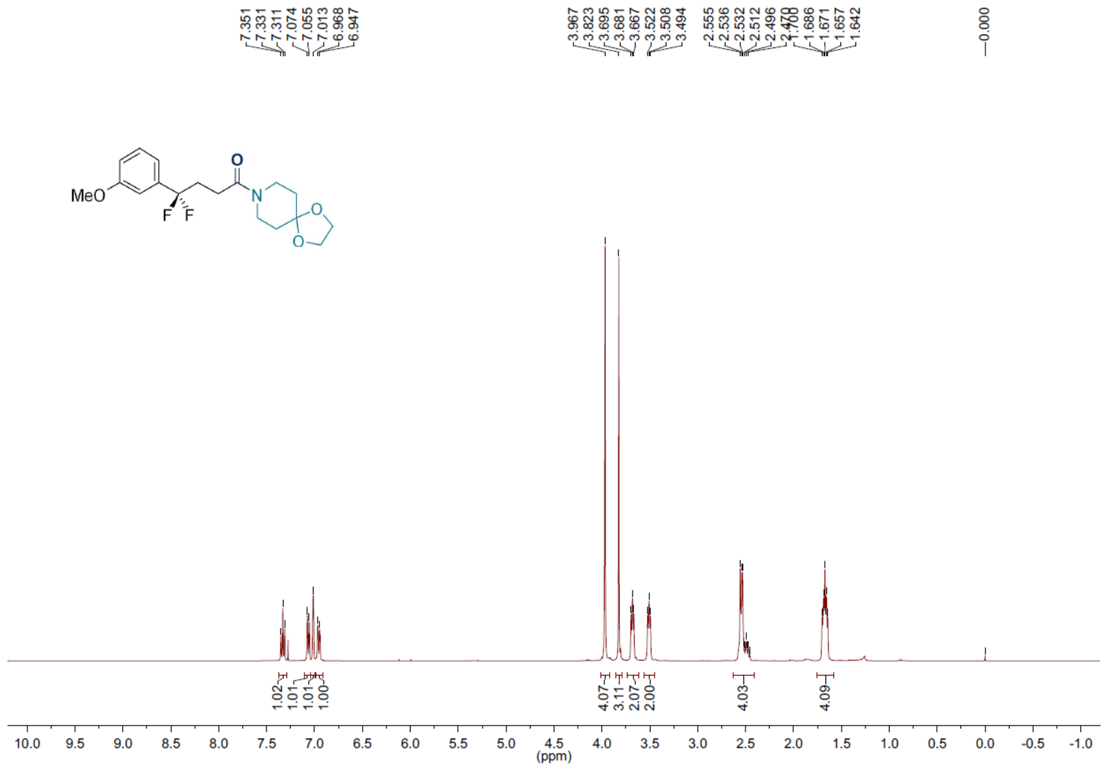

^{1}H NMR spectrum of **5b** in $CDCl_3$ (400 MHz)

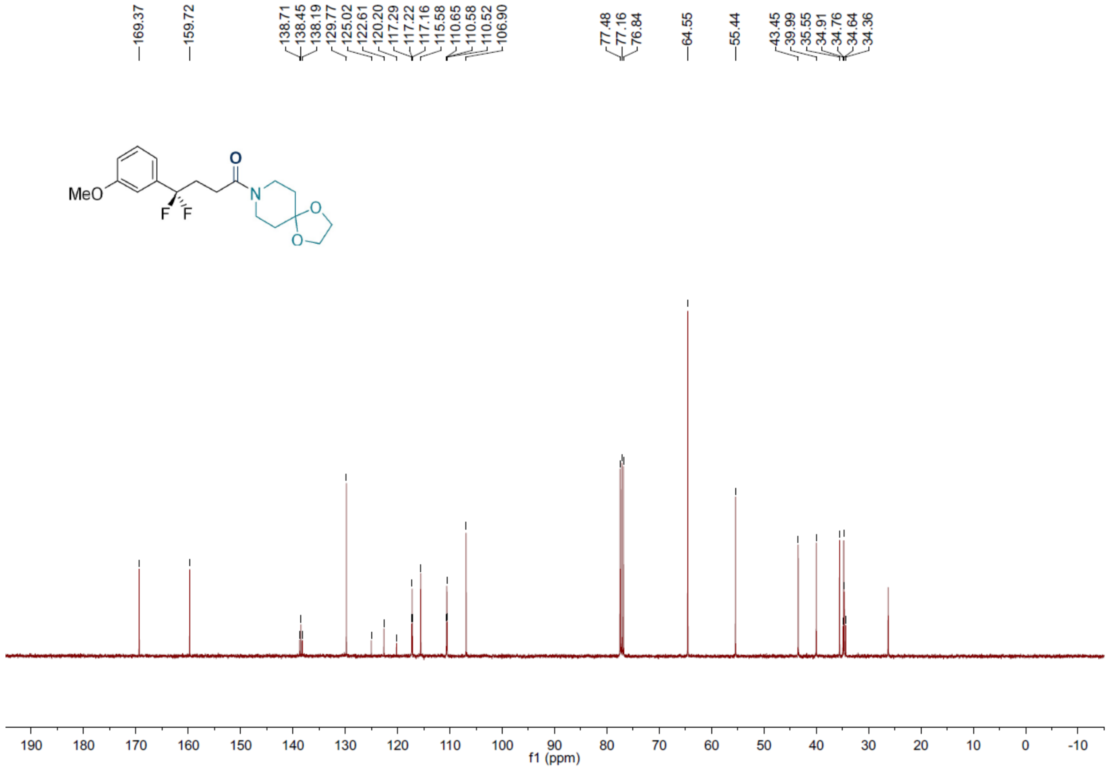

^{13}C NMR spectrum of **5b** in $CDCl_3$ (101 MHz)

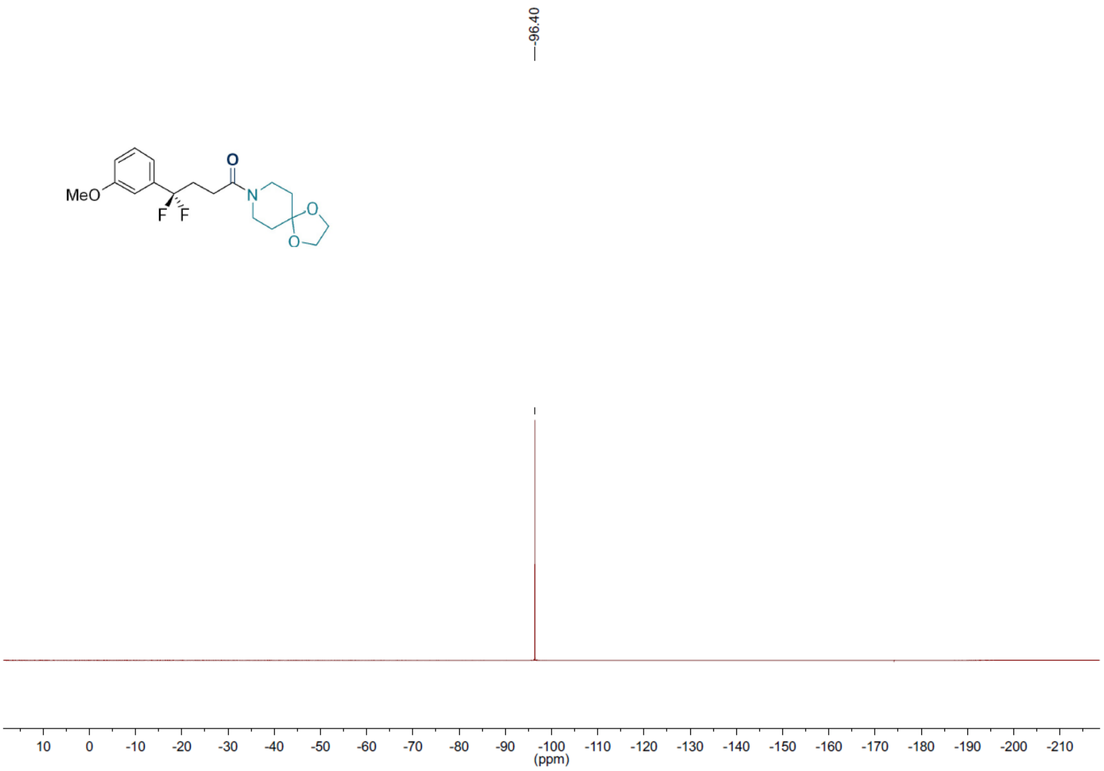

^{19}F NMR spectrum of **5b** in $CDCl_3$ (376 MHz)

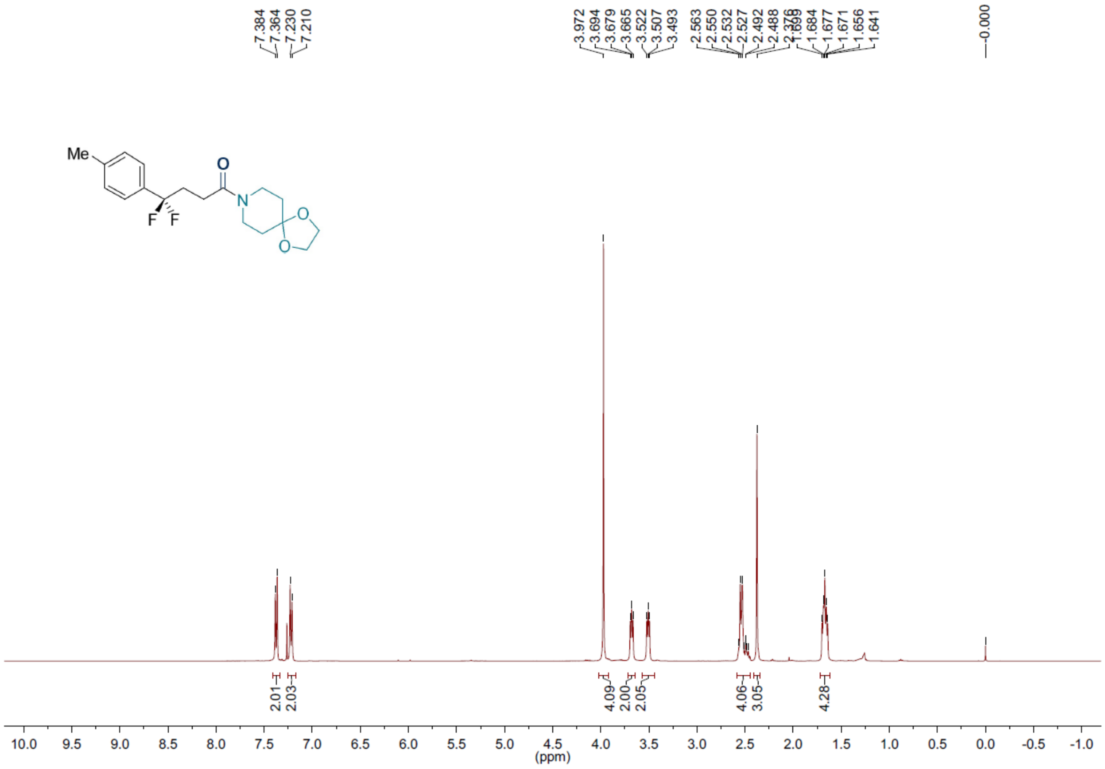

^{1}H NMR spectrum of **5c** in $CDCl_3$ (400 MHz)

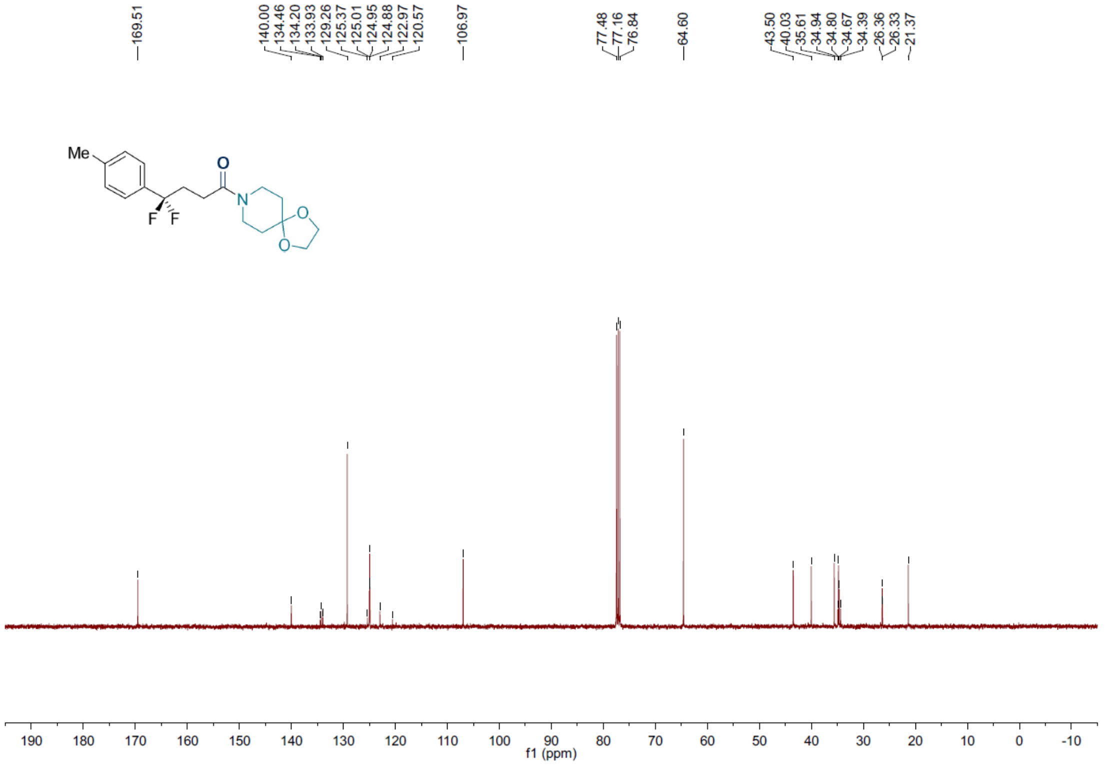

^{13}C NMR spectrum of **5c** in $CDCl_3$ (101 MHz)

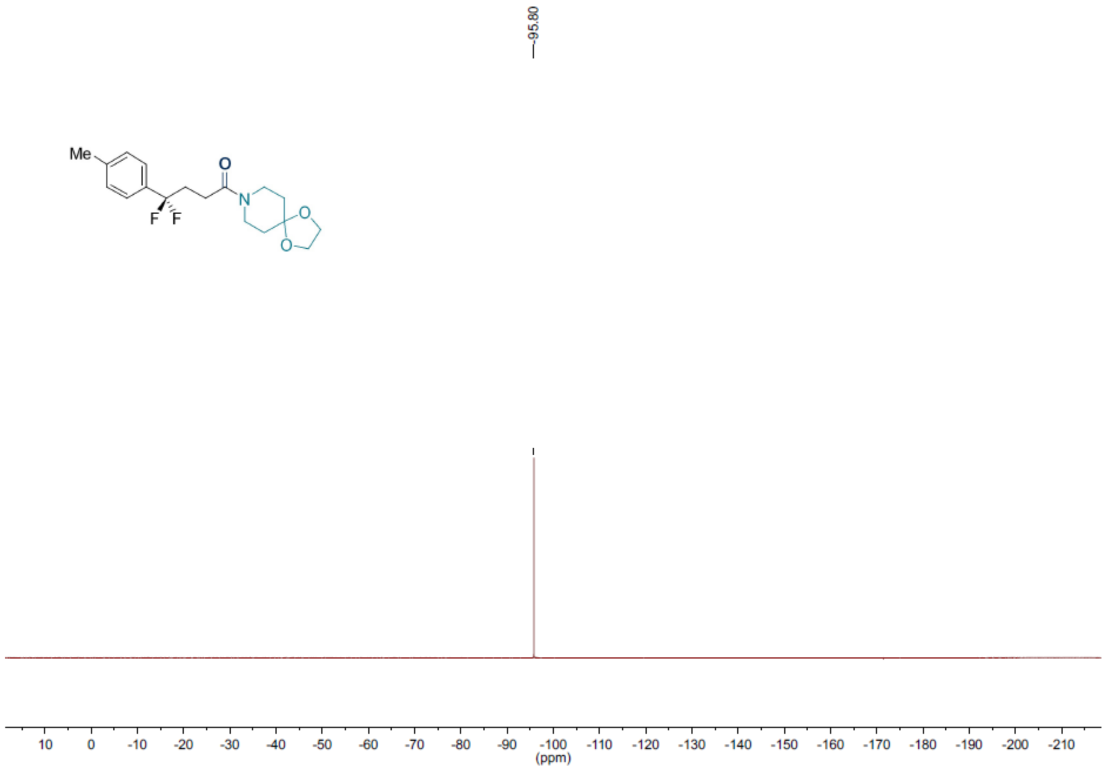

^{19}F NMR spectrum of **5c** in $CDCl_3$ (376 MHz)

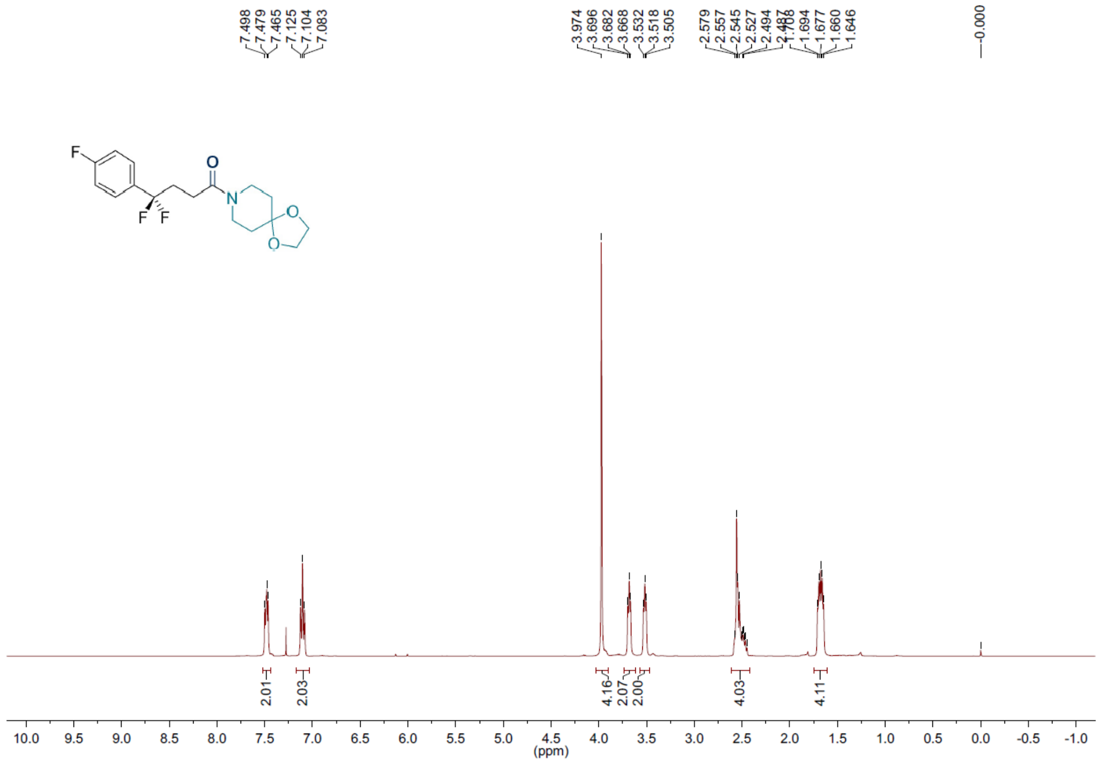

^{1}H NMR spectrum of **5d** in $CDCl_3$ (400 MHz)

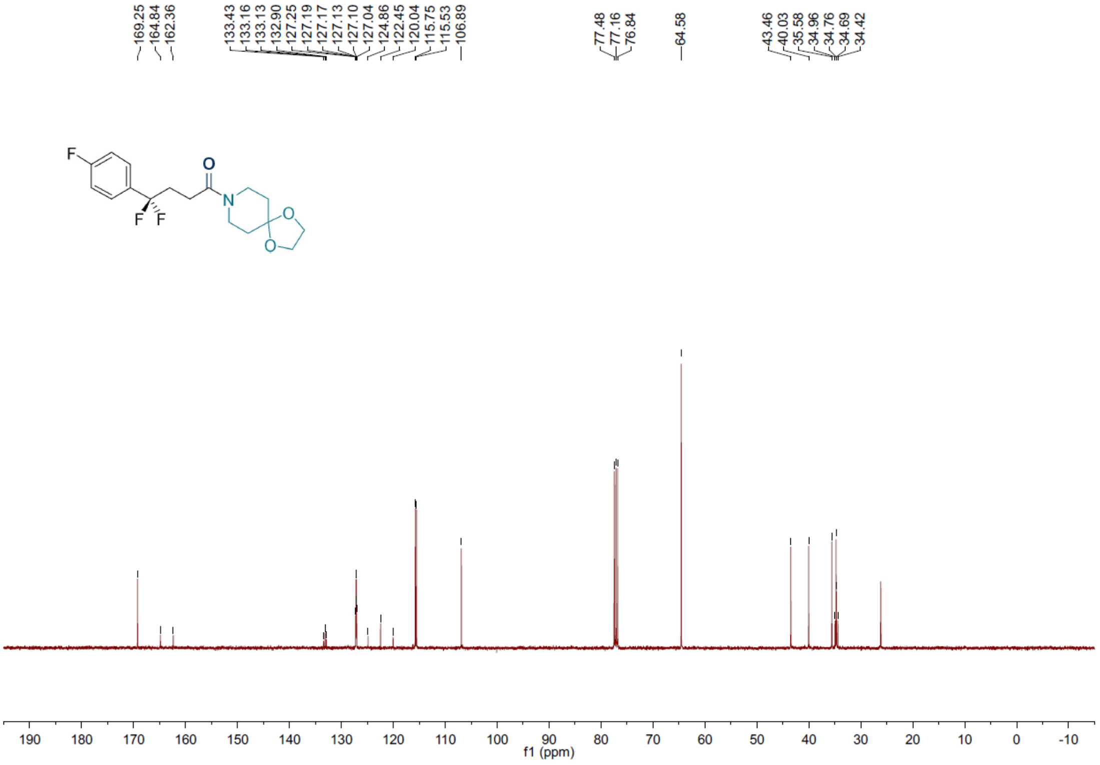

^{13}C NMR spectrum of **5d** in $CDCl_3$ (101 MHz)

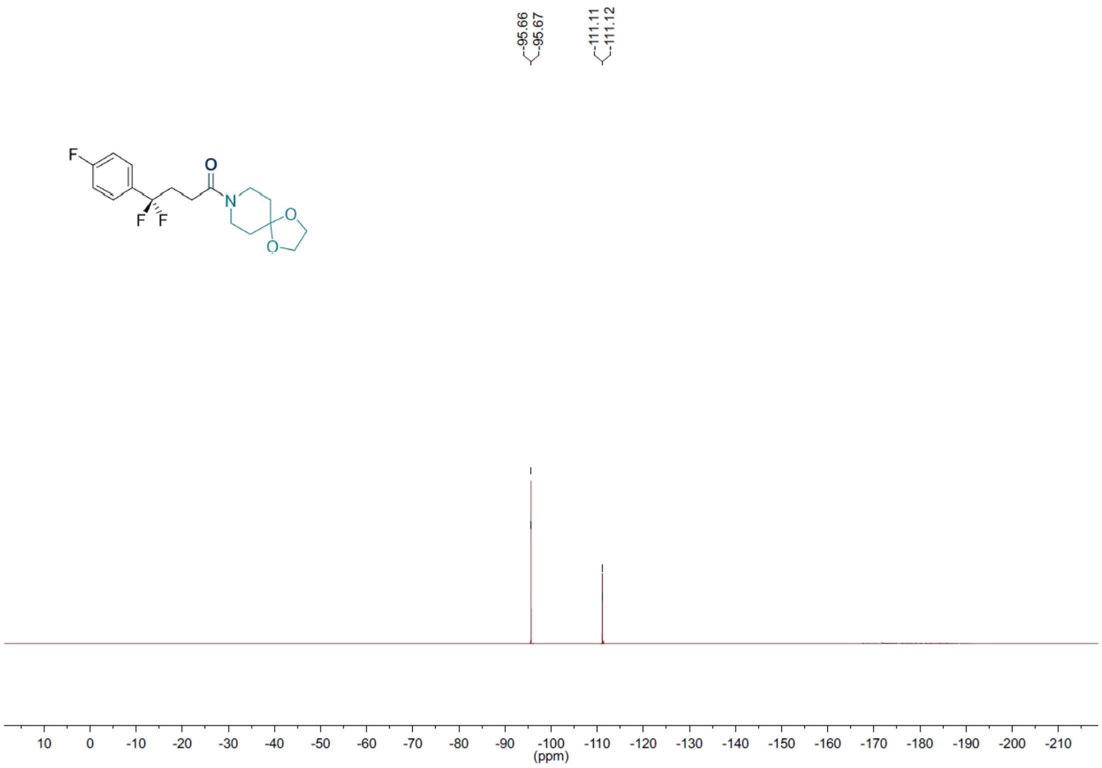

^{19}F NMR spectrum of **5d** in $CDCl_3$ (376 MHz)

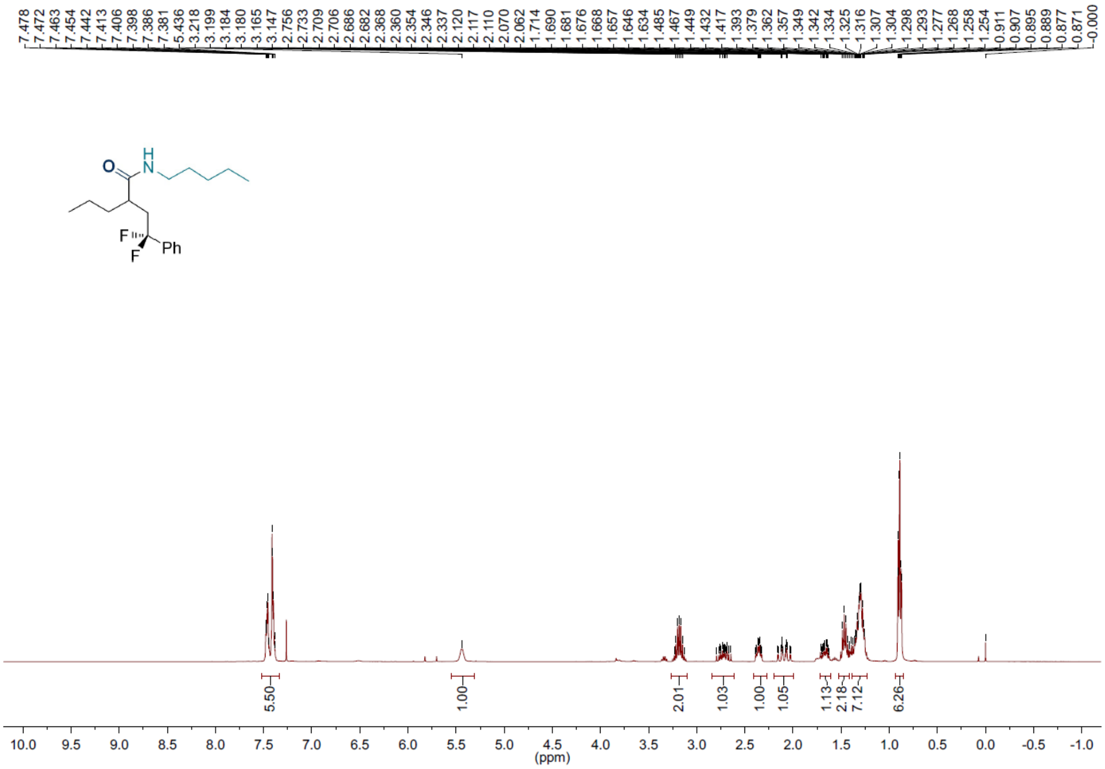

^{1}H NMR spectrum of **6a** in $CDCl_3$ (400 MHz)

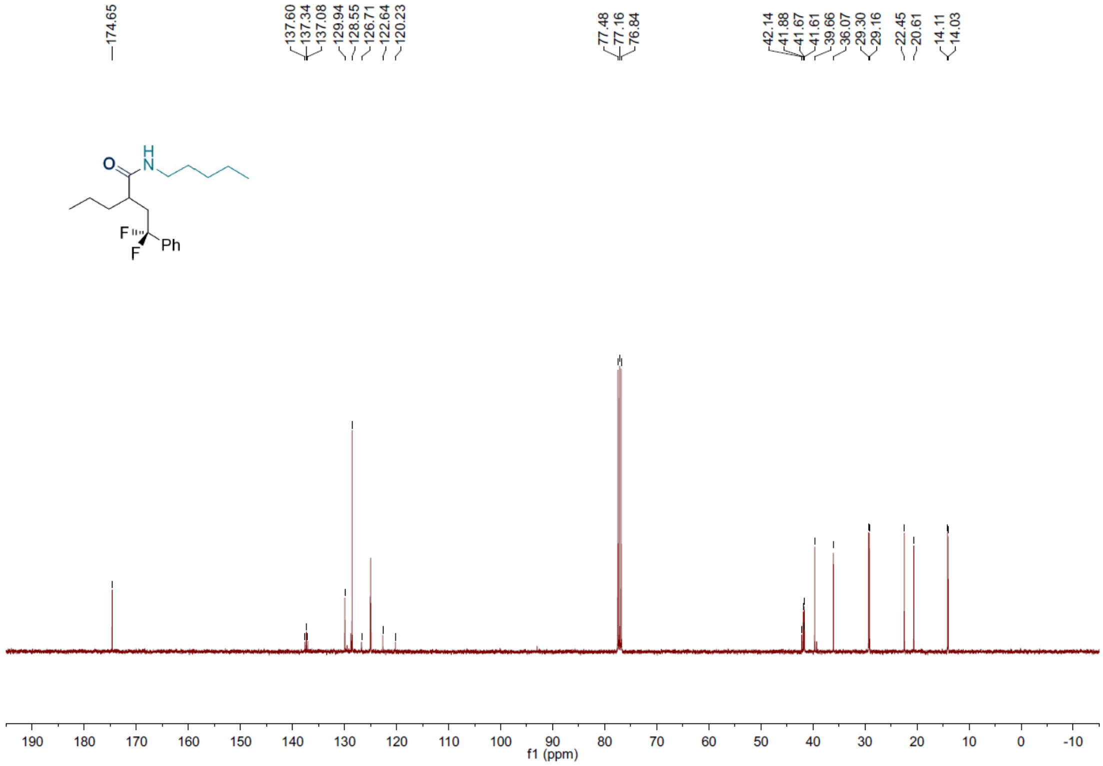

^{13}C NMR spectrum of **6a** in $CDCl_3$ (101 MHz)

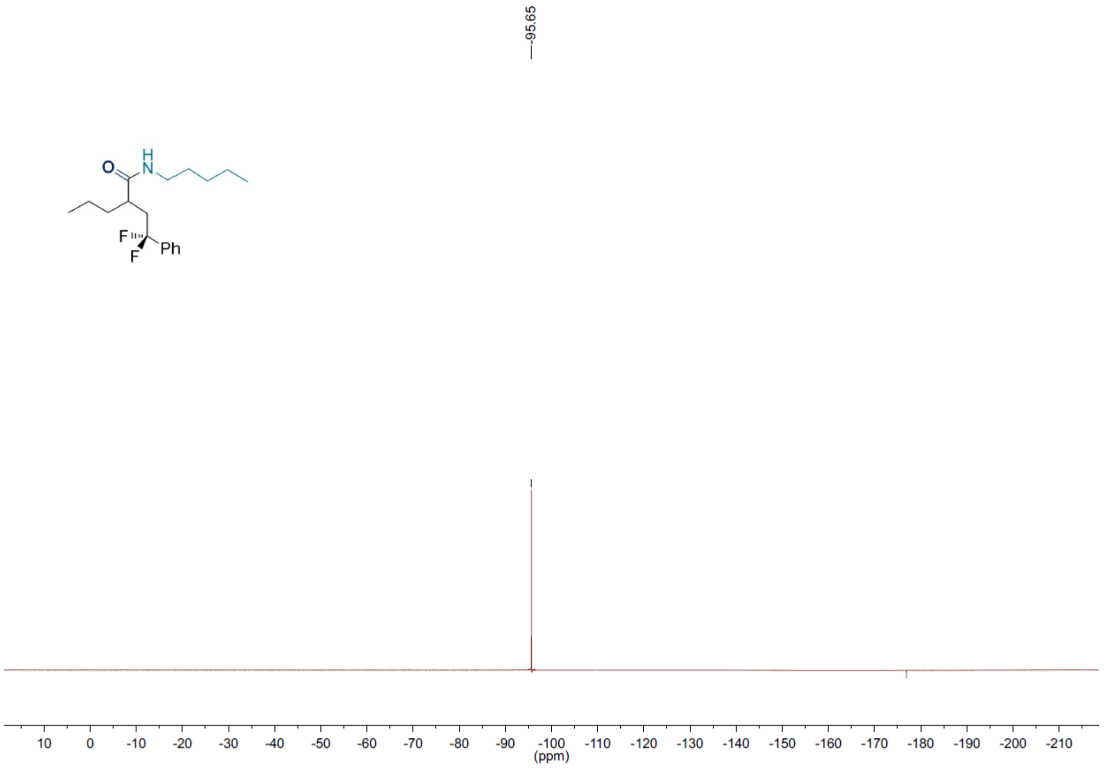

^{19}F NMR spectrum of **6a** in $CDCl_3$ (376 MHz)

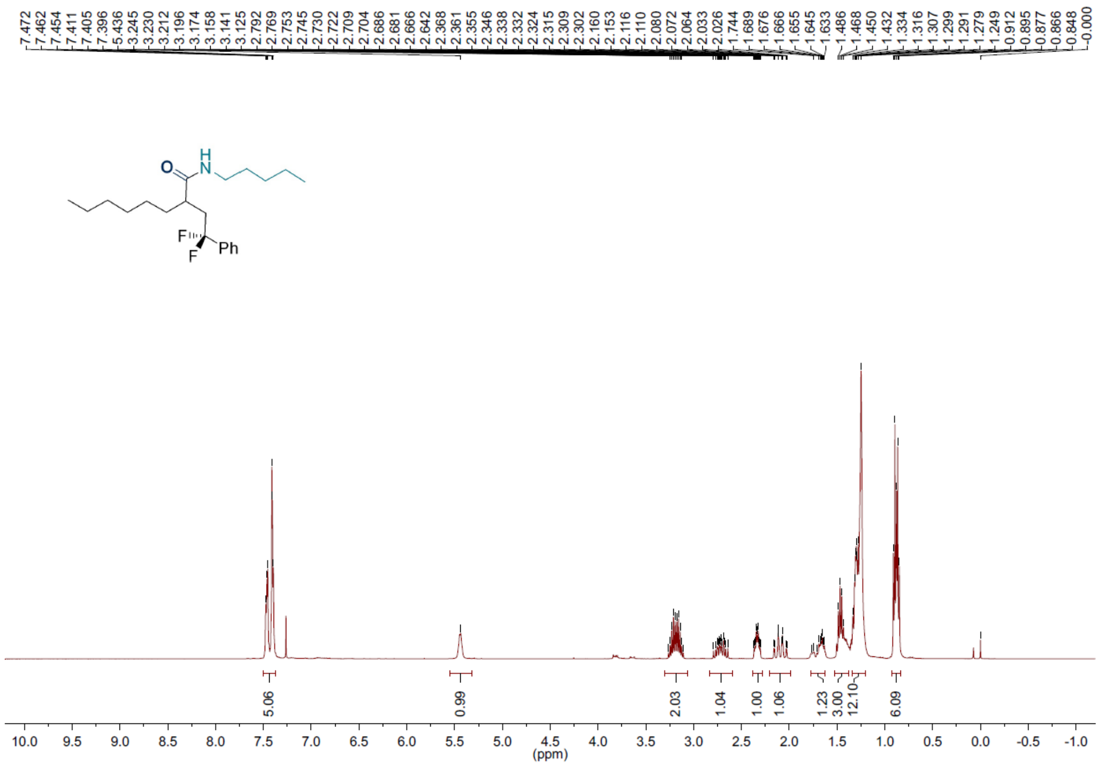

^{1}H NMR spectrum of 6**b** in $CDCl_3$ (400 MHz)

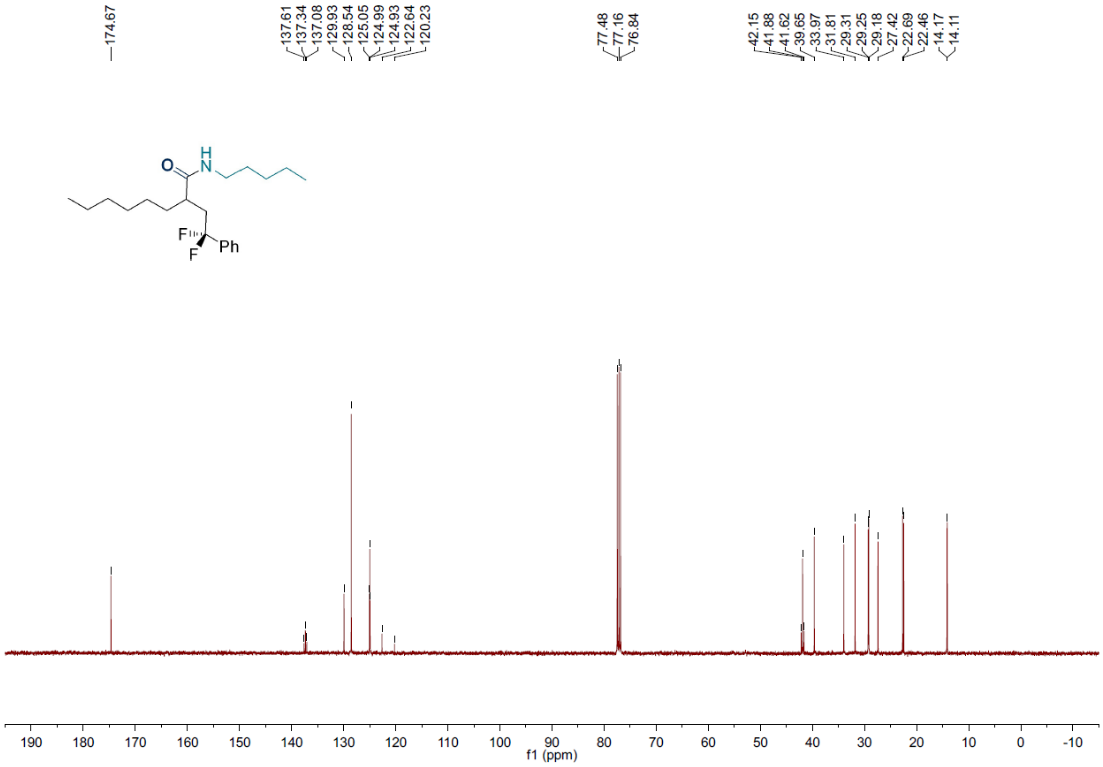

^{13}C NMR spectrum of **6b** in $CDCl_3$ (101 MHz)

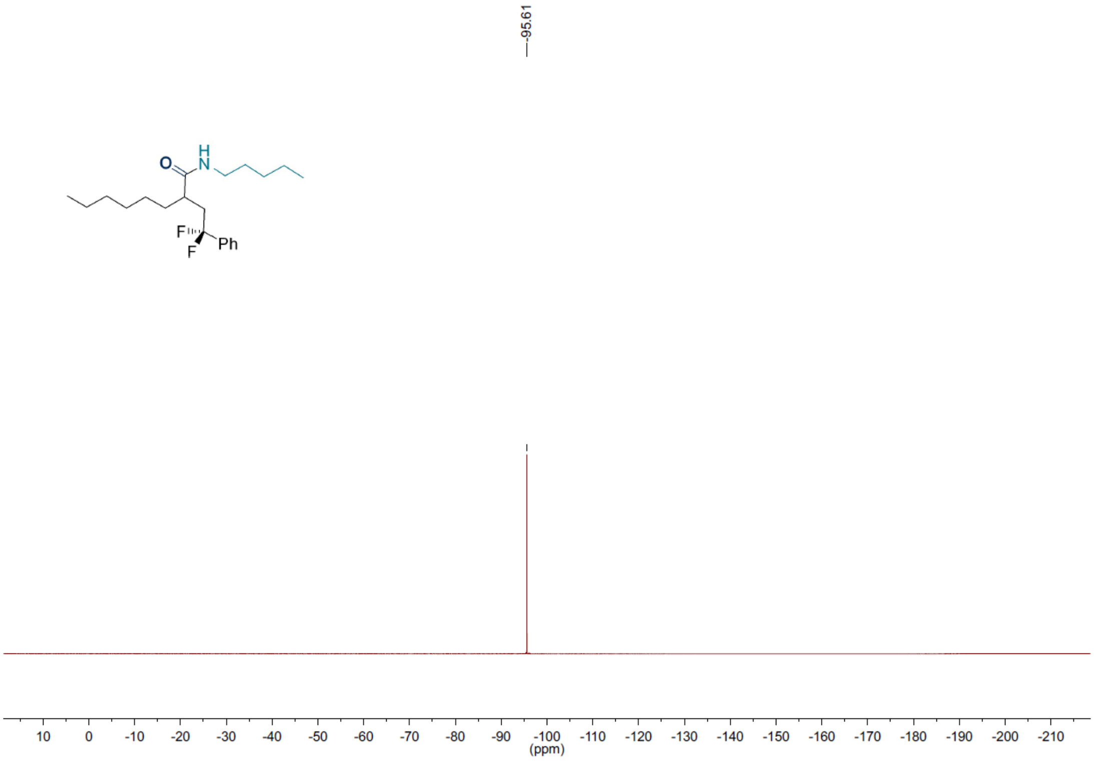

^{19}F NMR spectrum of **6b** in $CDCl_3$ (376 MHz)

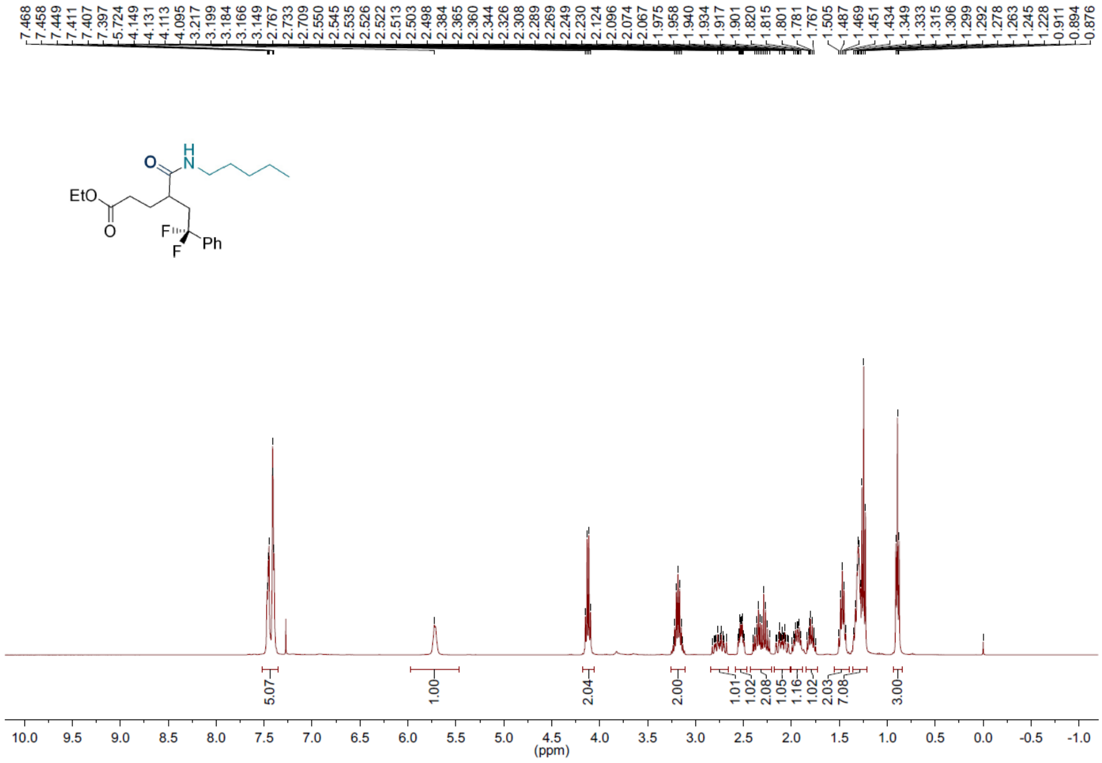

^{1}H NMR spectrum of **6c** in $CDCl_3$ (400 MHz)

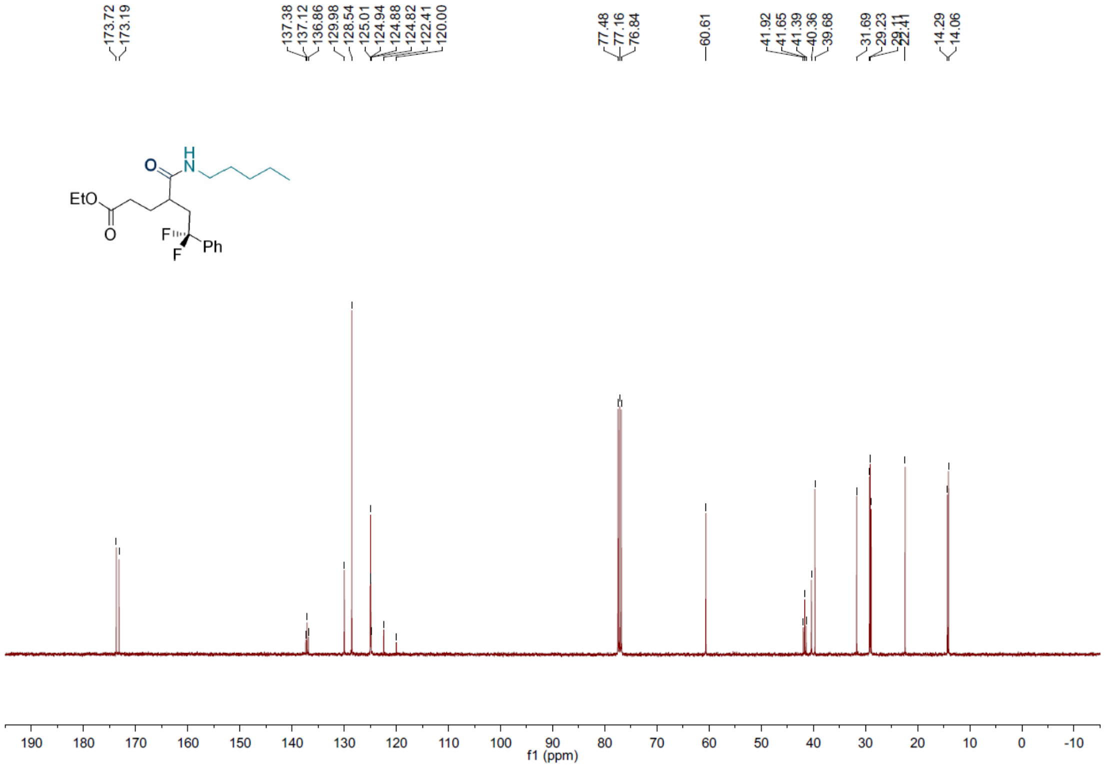

^{13}C NMR spectrum of **6c** in $CDCl_3$ (101 MHz)

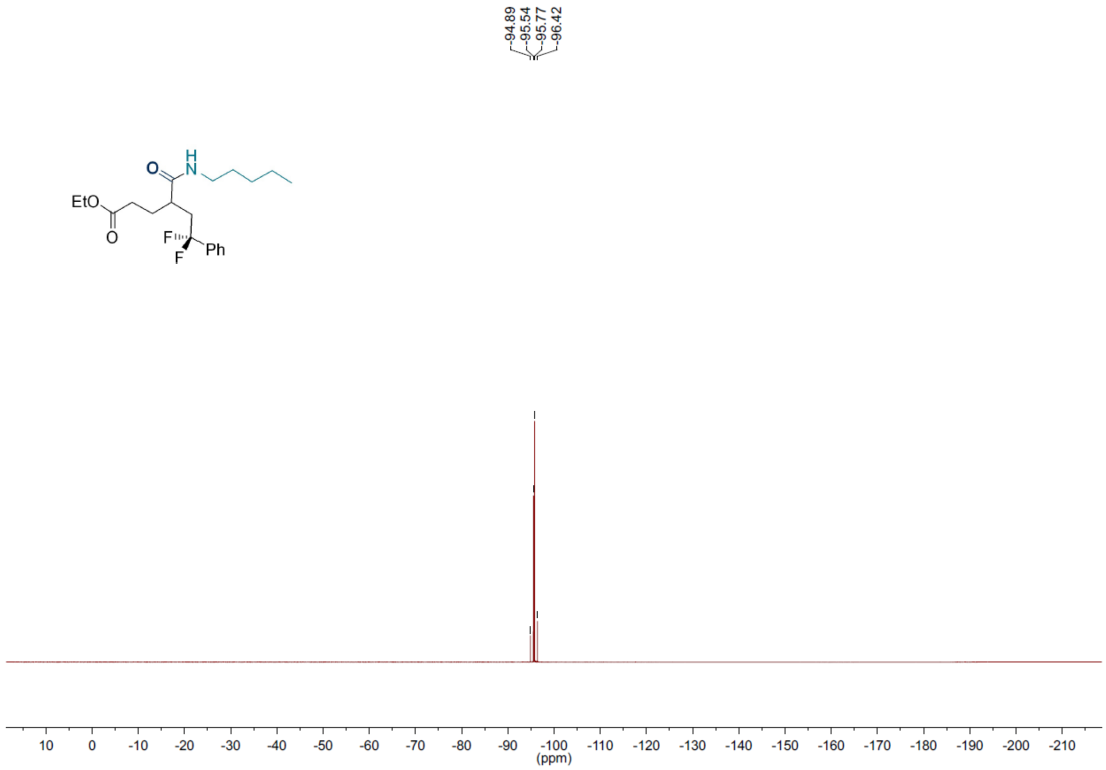

^{19}F NMR spectrum of **6c** in $CDCl_3$ (376 MHz)

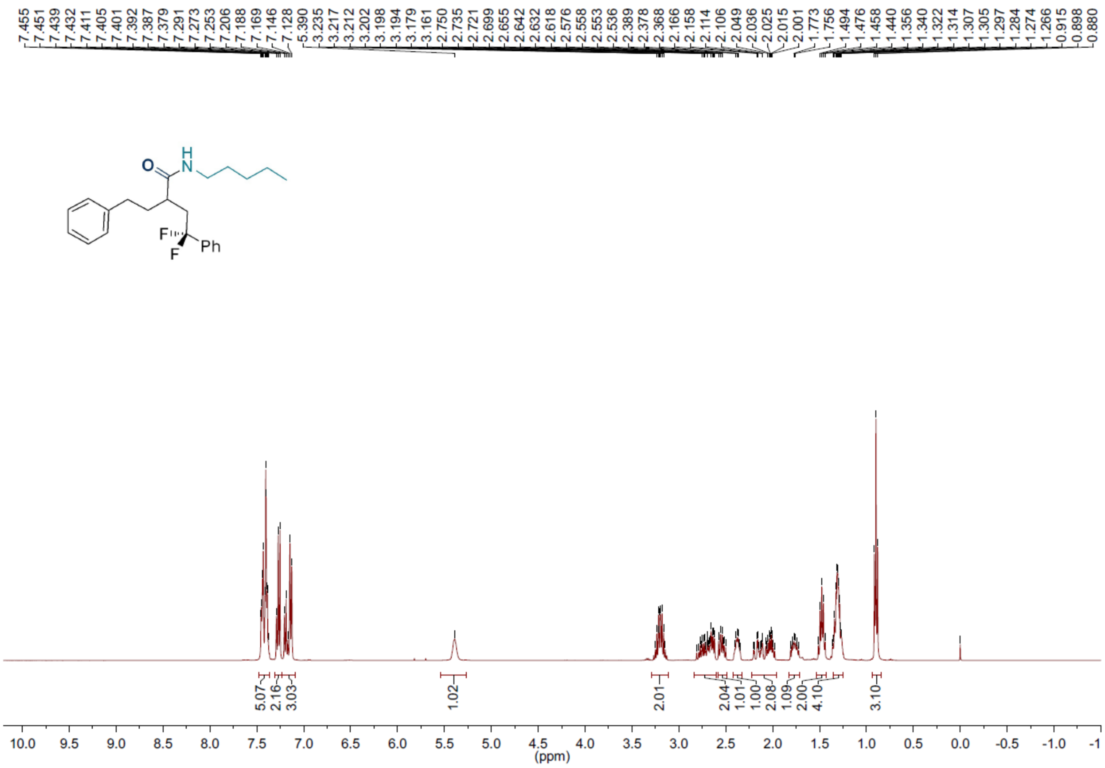

^{1}H NMR spectrum of **6d** in $CDCl_3$ (400 MHz)

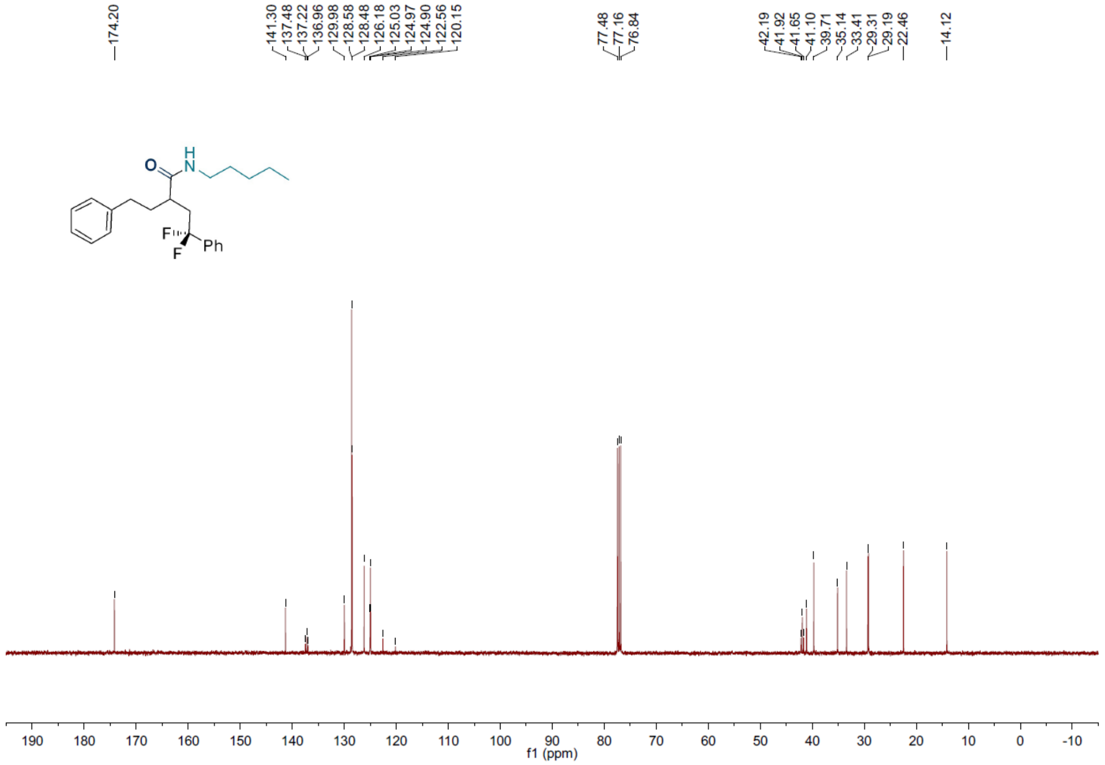

^{13}C NMR spectrum of **6d** in $CDCl_3$ (101 MHz)

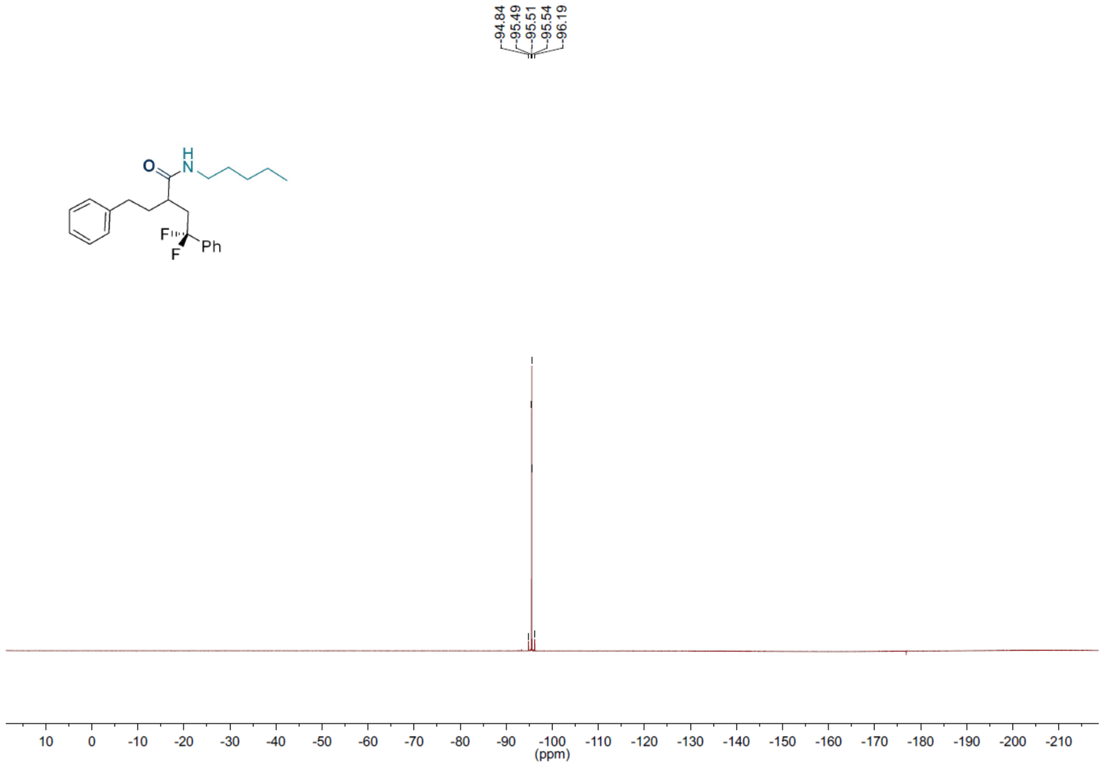

^{19}F NMR spectrum of **6d** in $CDCl_3$ (376 MHz)

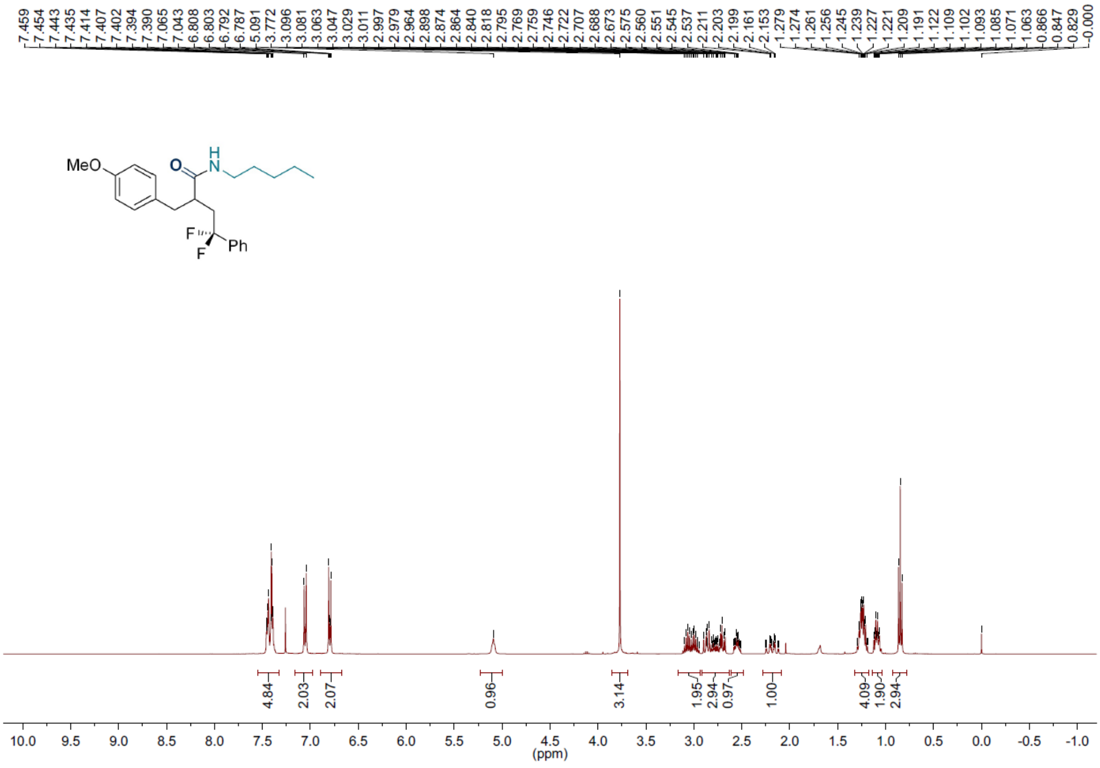

^{1}H NMR spectrum of **6e** in $CDCl_3$ (400 MHz)

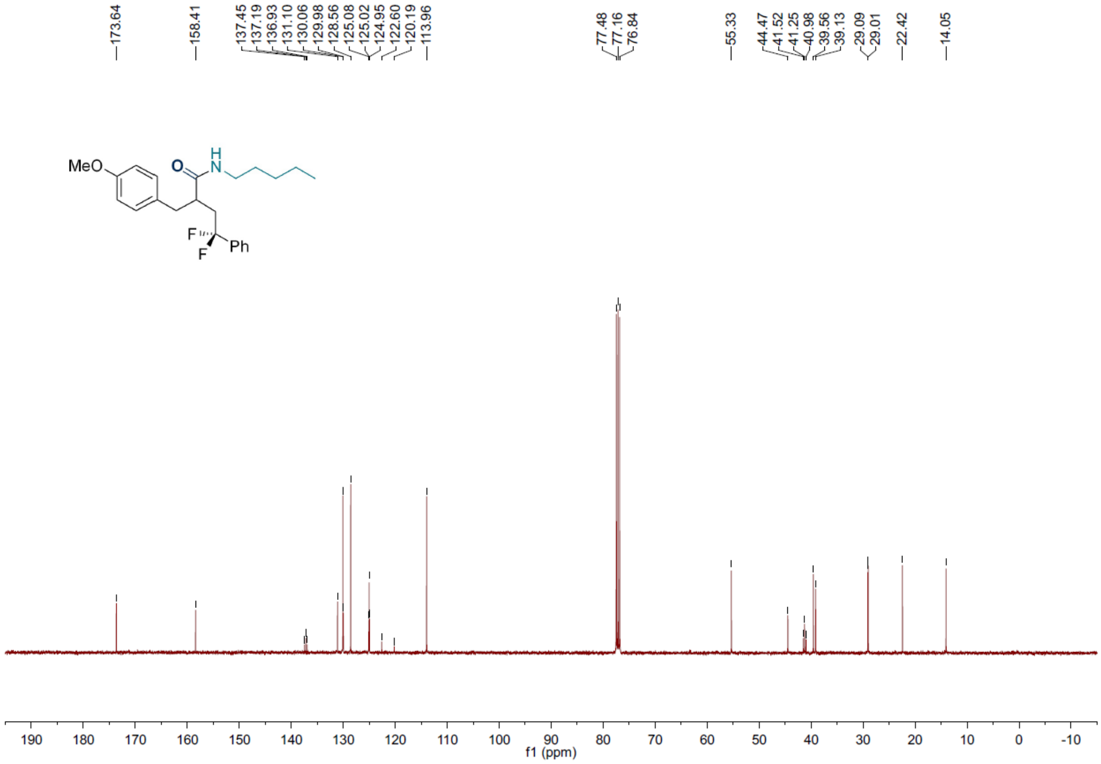

^{13}C NMR spectrum of **6e** in $CDCl_3$ (101 MHz)

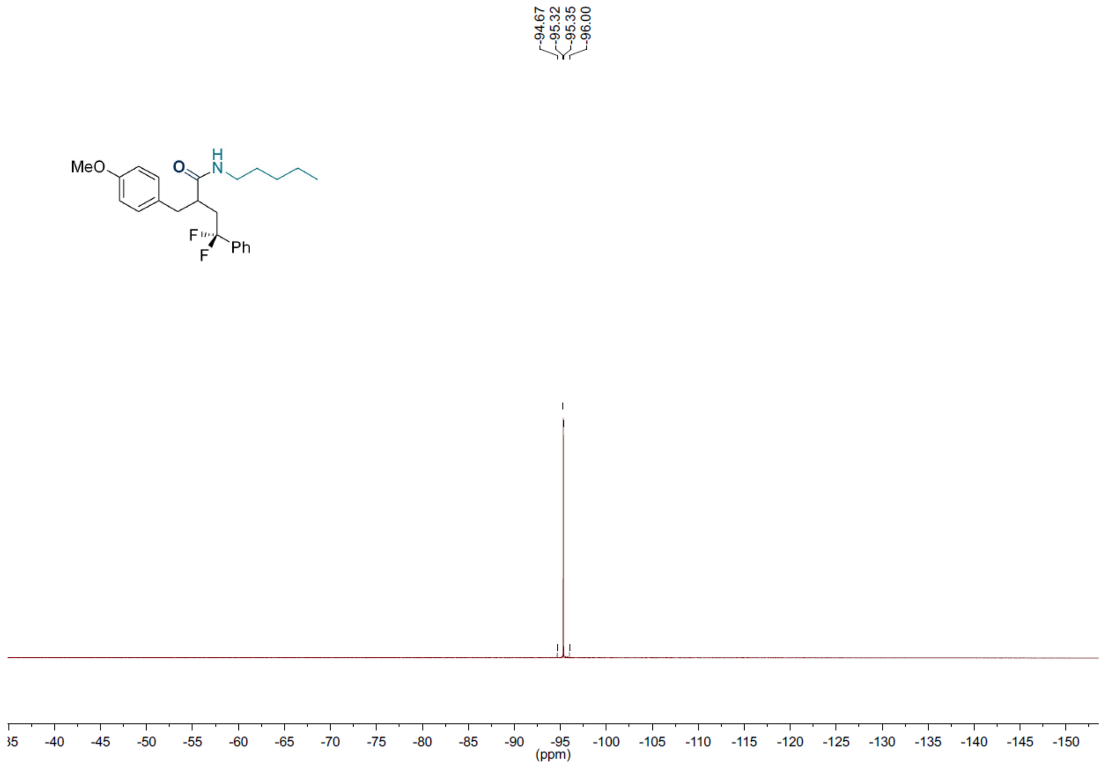

^{19}F NMR spectrum of **6e** in $CDCl_3$ (376 MHz)

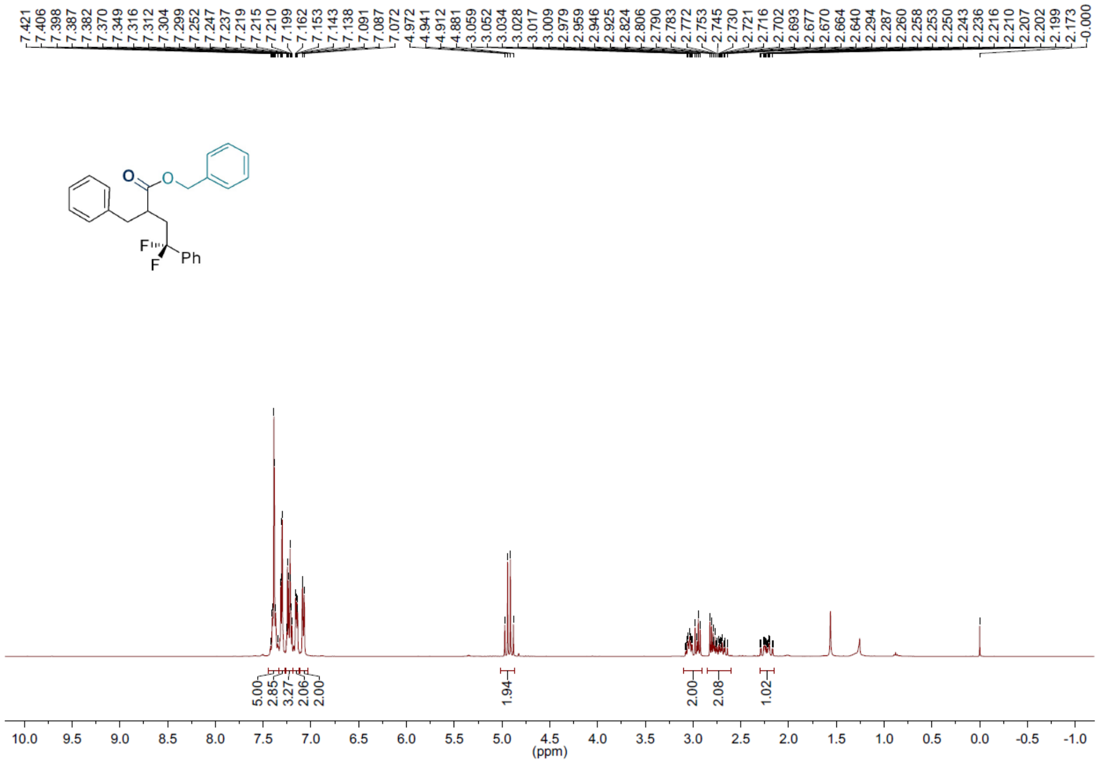

^{1}H NMR spectrum of **6f** in $CDCl_3$ (400 MHz)

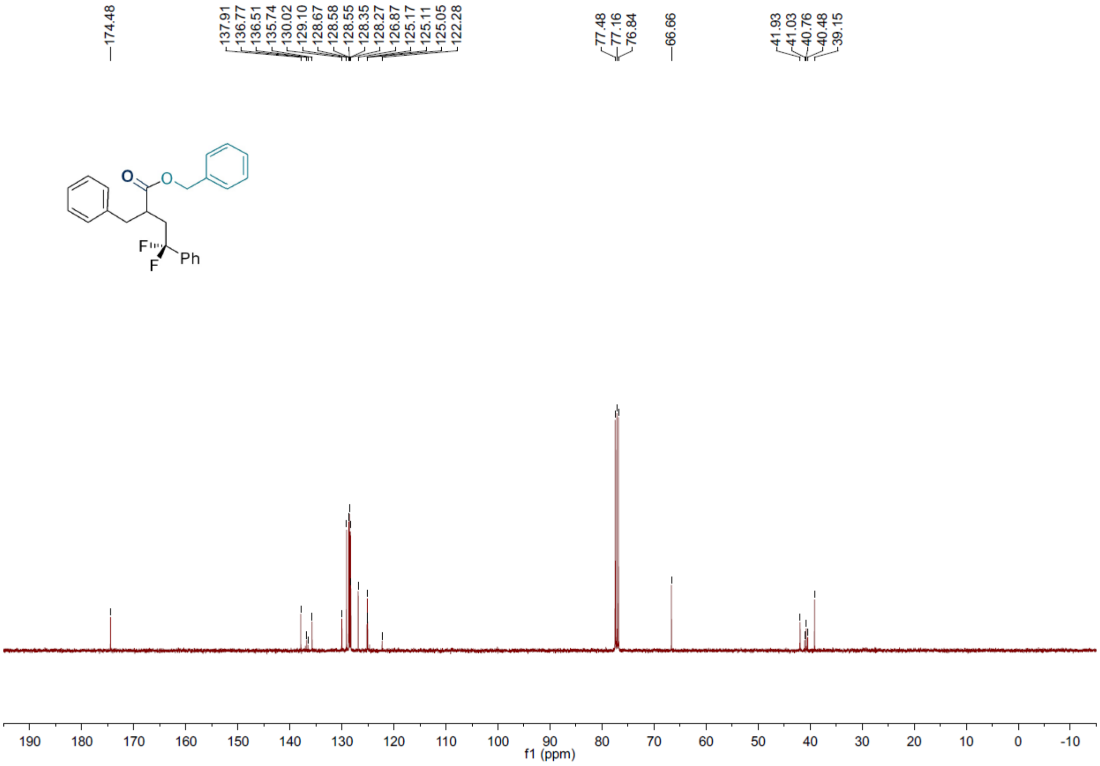

^{13}C NMR spectrum of **6f** in $CDCl_3$ (101 MHz)

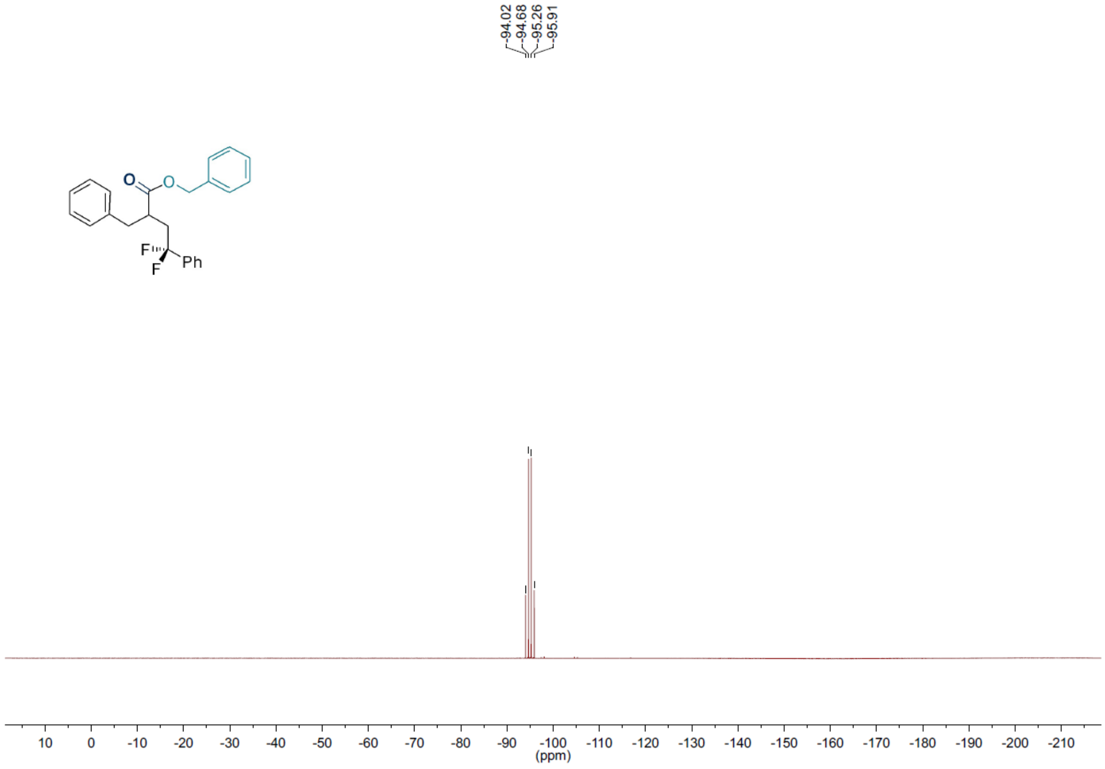

^{19}F NMR spectrum of **6f** in $CDCl_3$ (376 MHz)

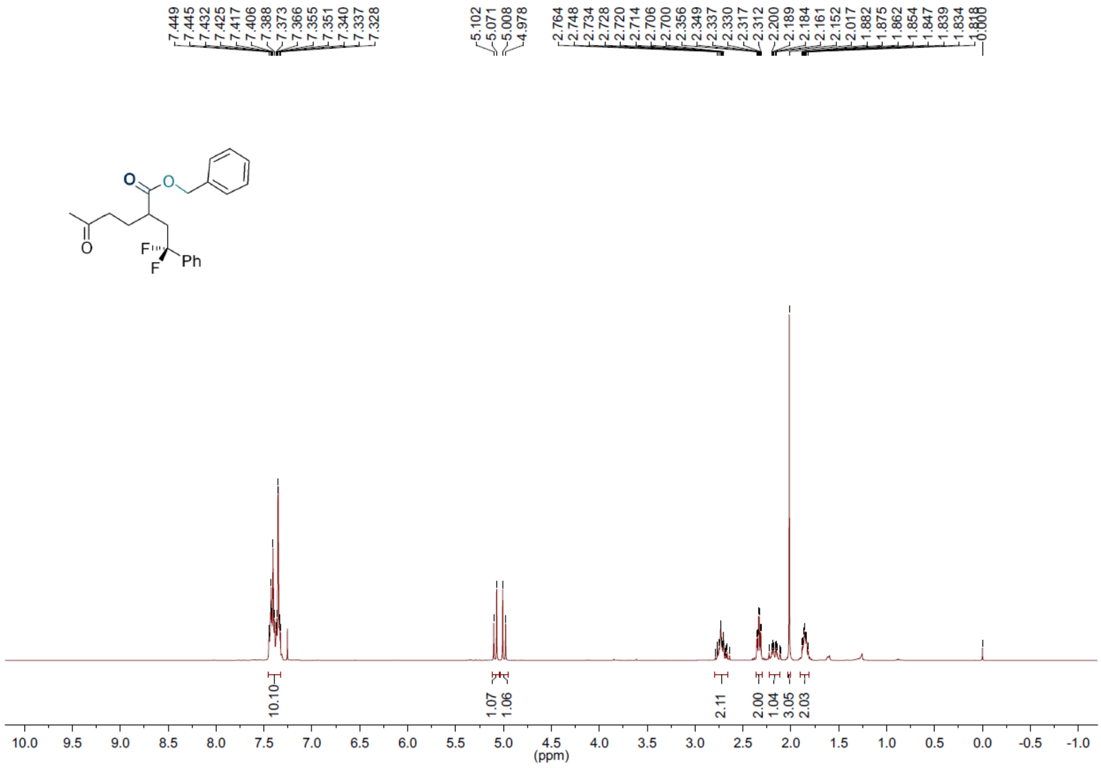

^{1}H NMR spectrum of **6g** in $CDCl_3$ (400 MHz)

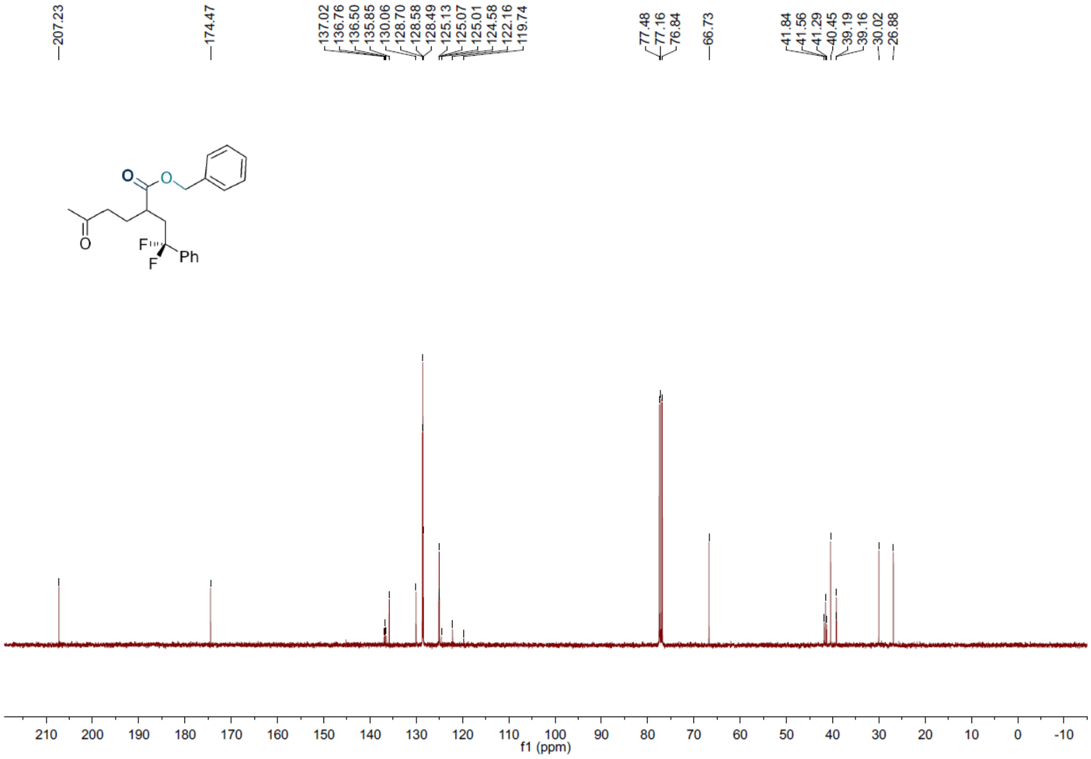

^{13}C NMR spectrum of **6g** in $CDCl_3$ (101 MHz)

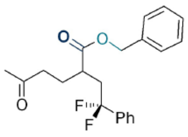

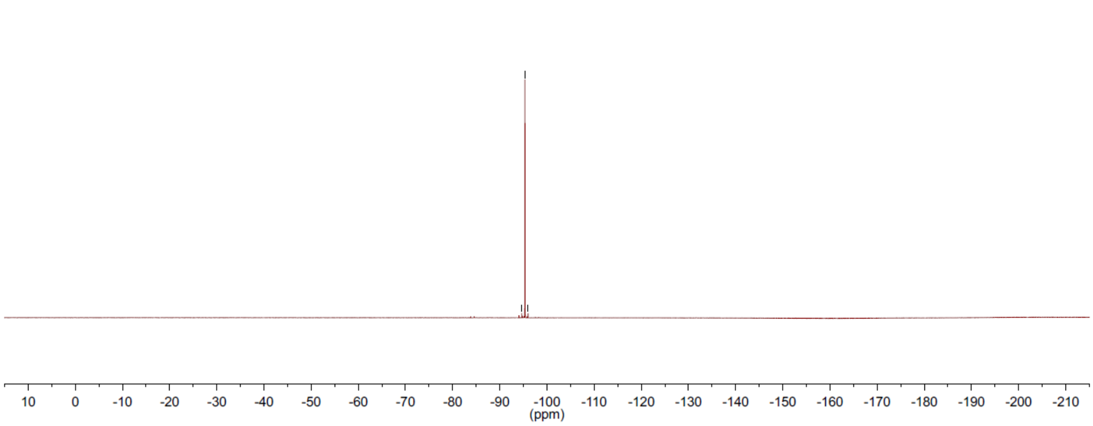

^{19}F NMR spectrum of **6g** in $CDCl_3$ (376 MHz)

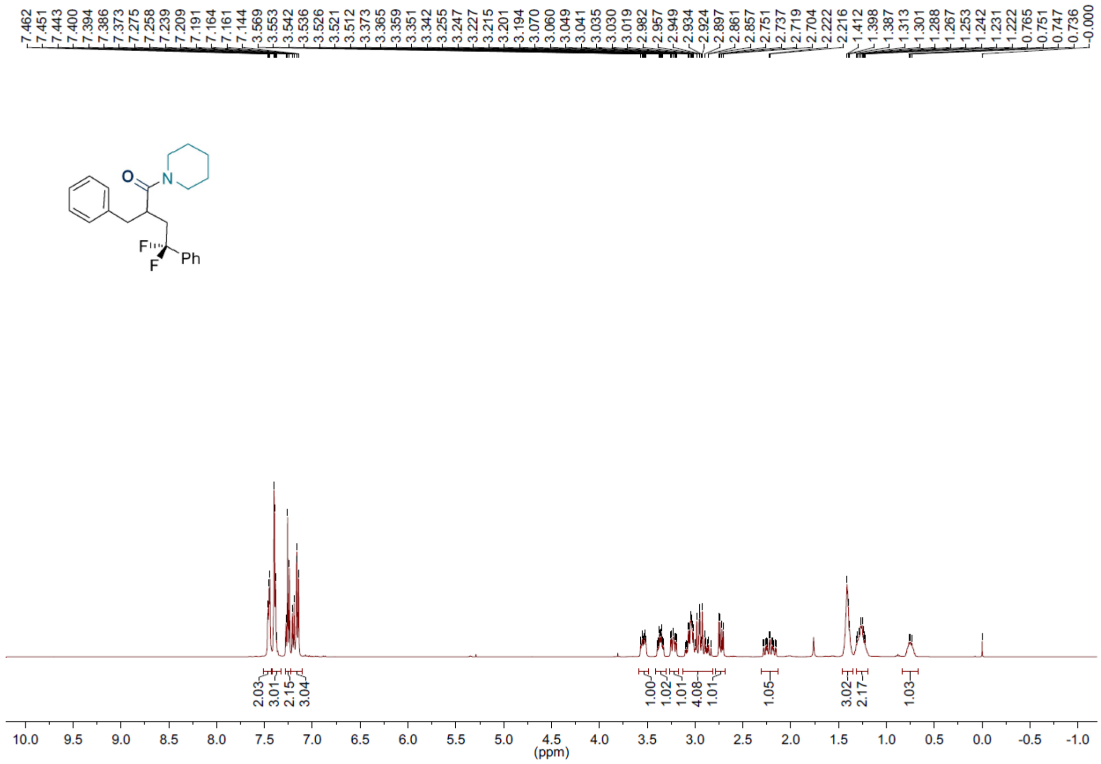

^{1}H NMR spectrum of **6h** in $CDCl_3$ (400 MHz)

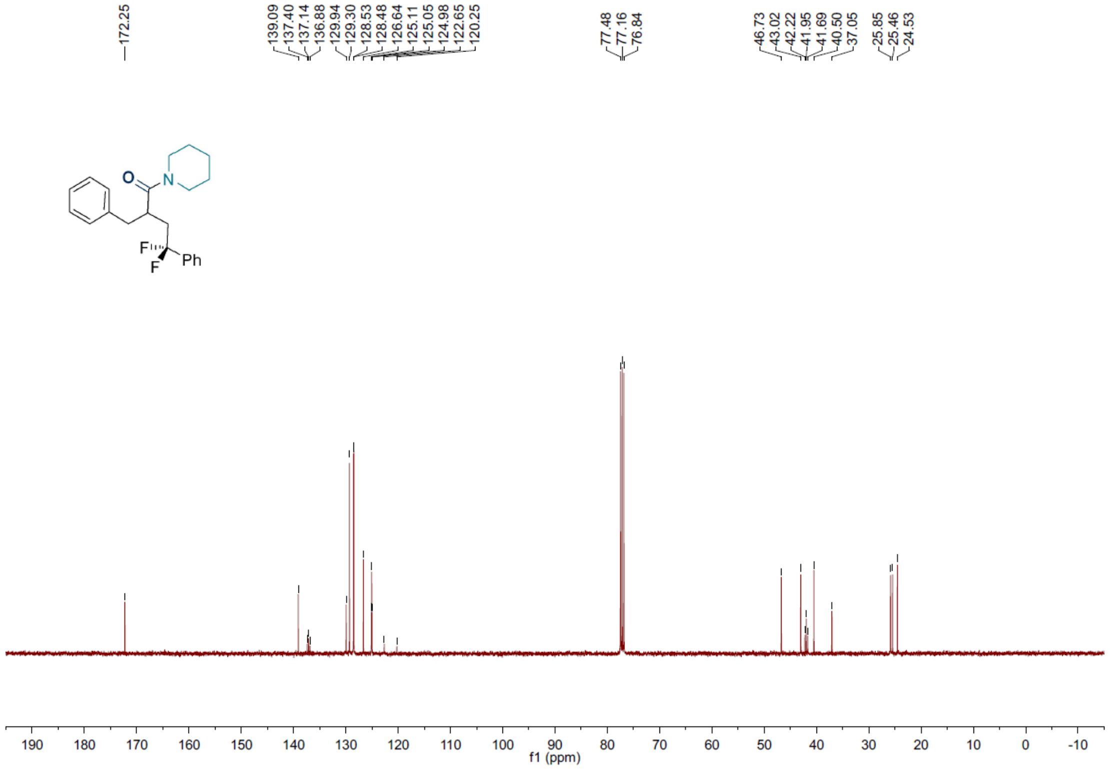

^{13}C NMR spectrum of **6h** in $CDCl_3$ (101 MHz)

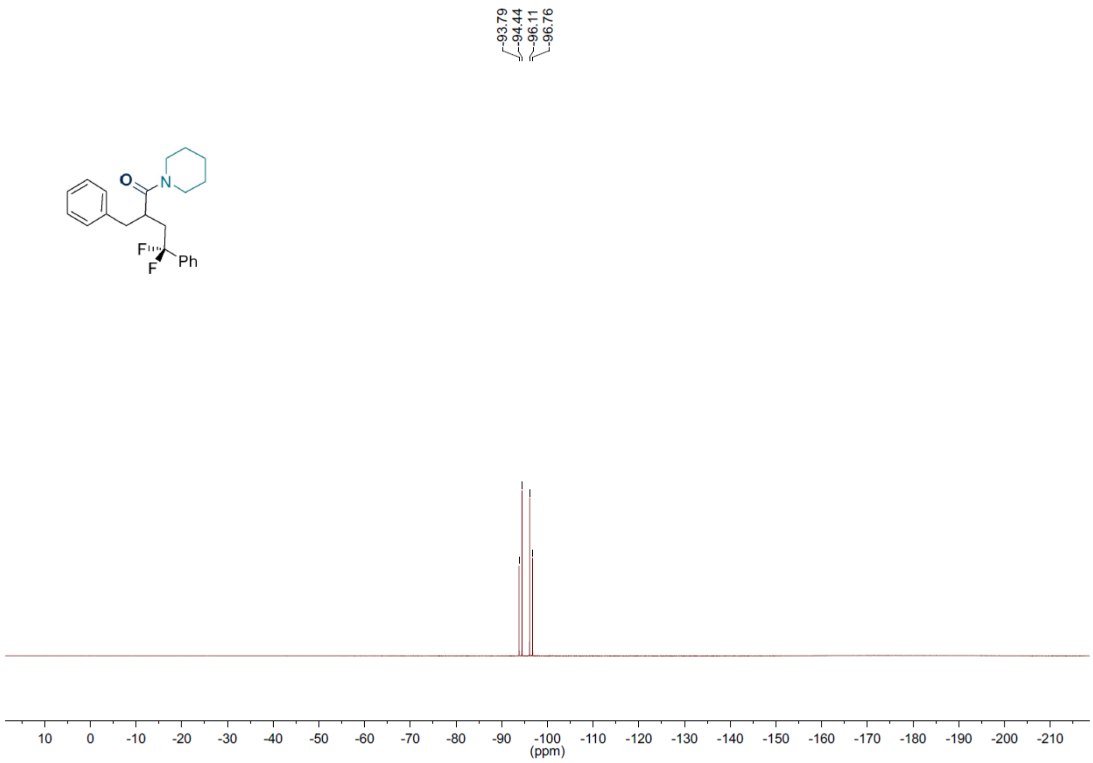

^{19}F NMR spectrum of **6h** in $CDCl_3$ (376 MHz)

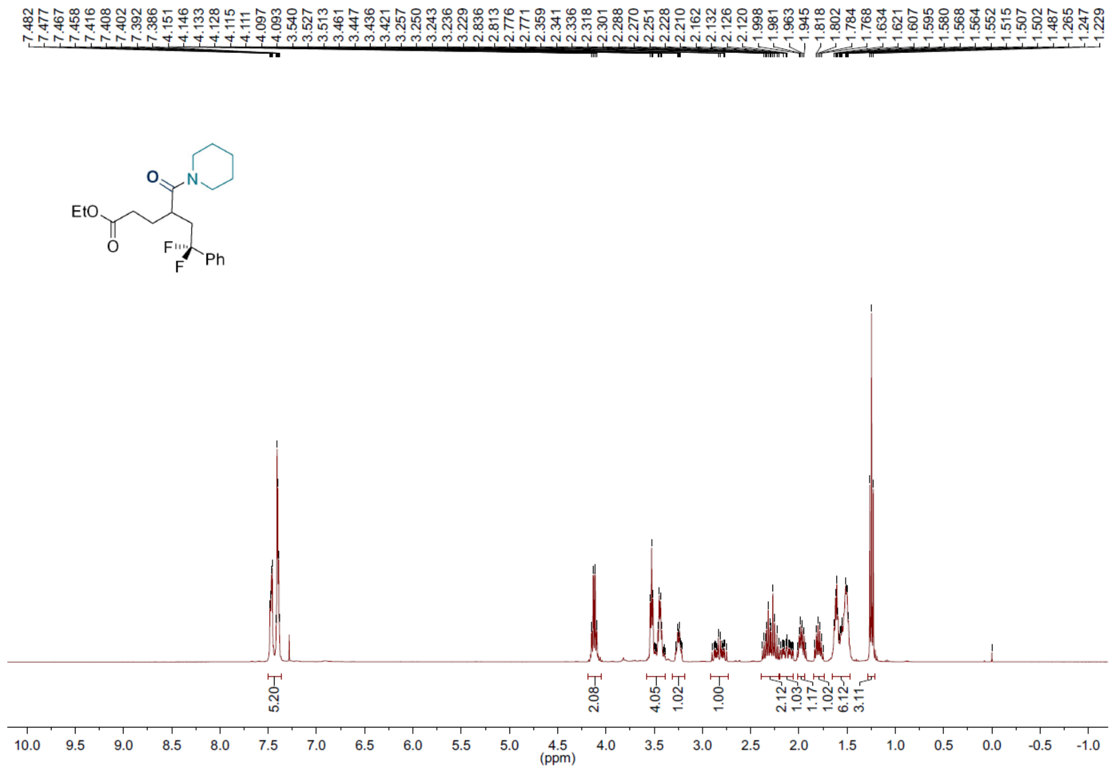

^{1}H NMR spectrum of **6i** in $CDCl_3$ (400 MHz)

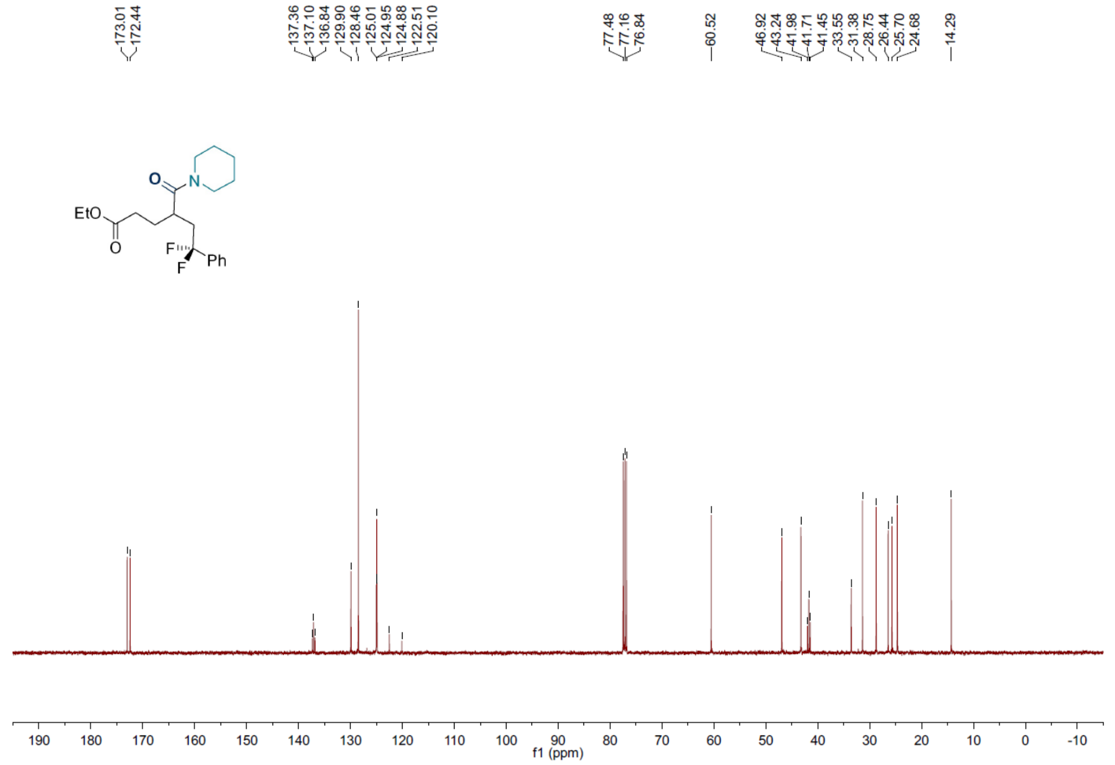

^{13}C NMR spectrum of **6i** in $CDCl_3$ (101 MHz)

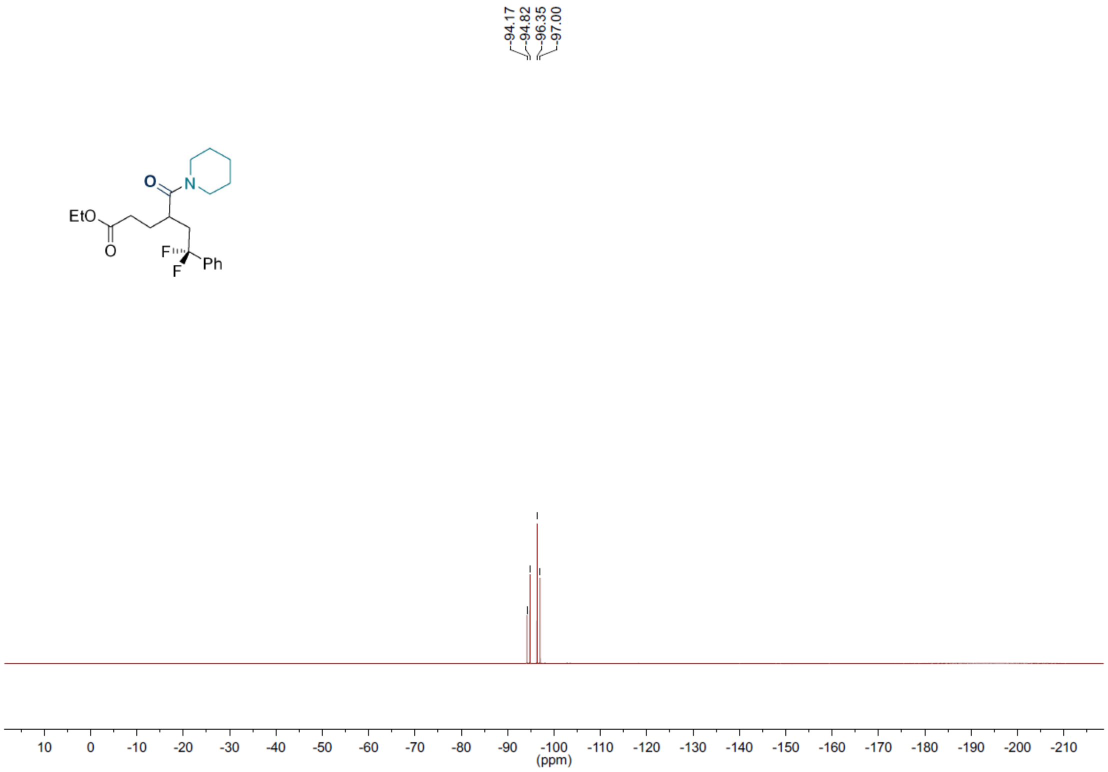

^{19}F NMR spectrum of **6i** in $CDCl_3$ (376 MHz)

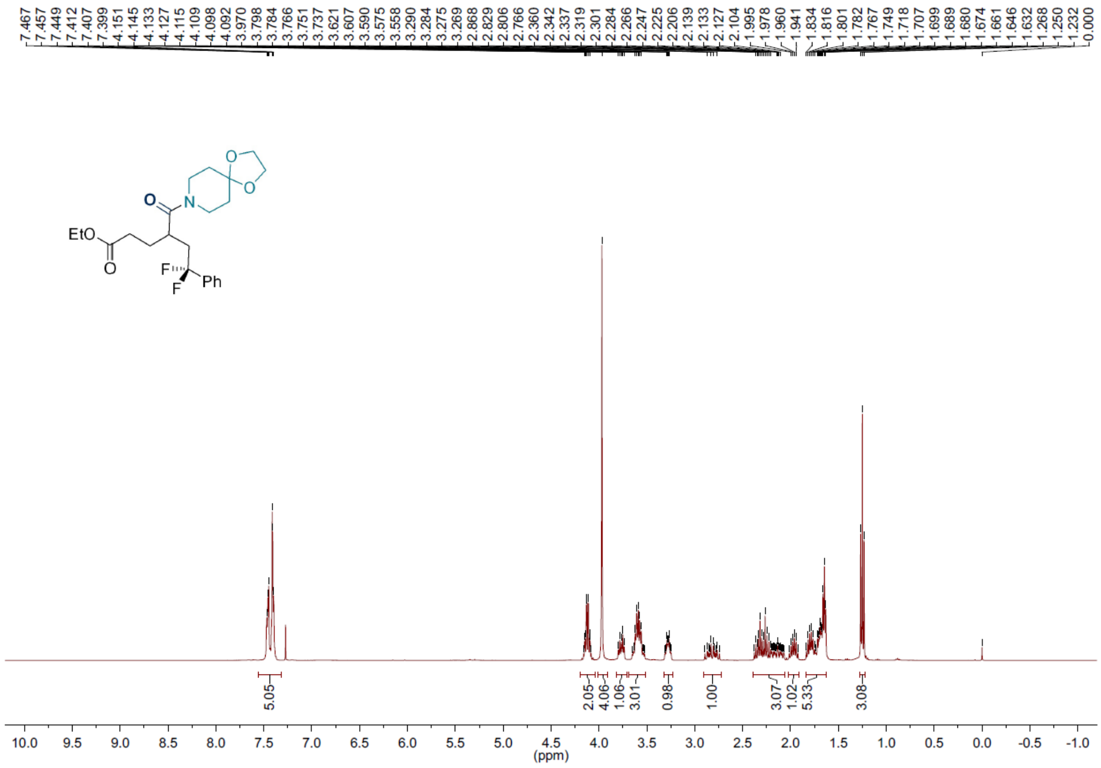

^{1}H NMR spectrum of **6j** in $CDCl_3$ (400 MHz)

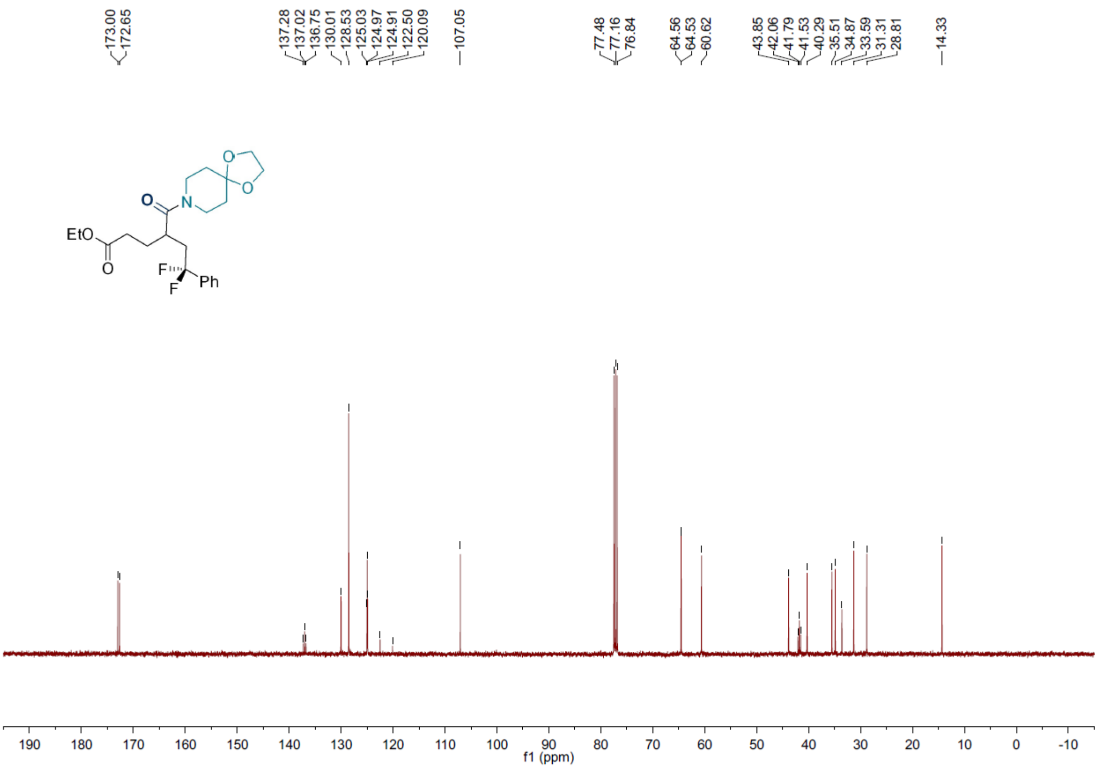

^{13}C NMR spectrum of **6j** in $CDCl_3$ (101 MHz)

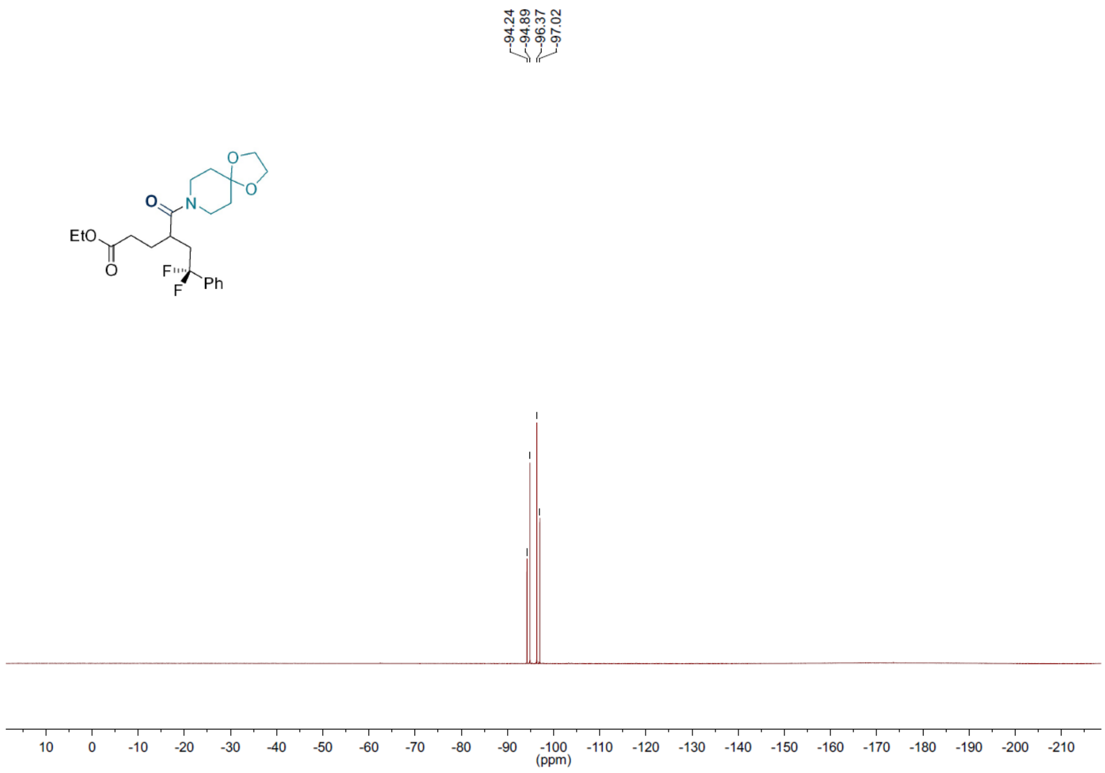

^{19}F NMR spectrum of **6j** in $CDCl_3$ (376 MHz)

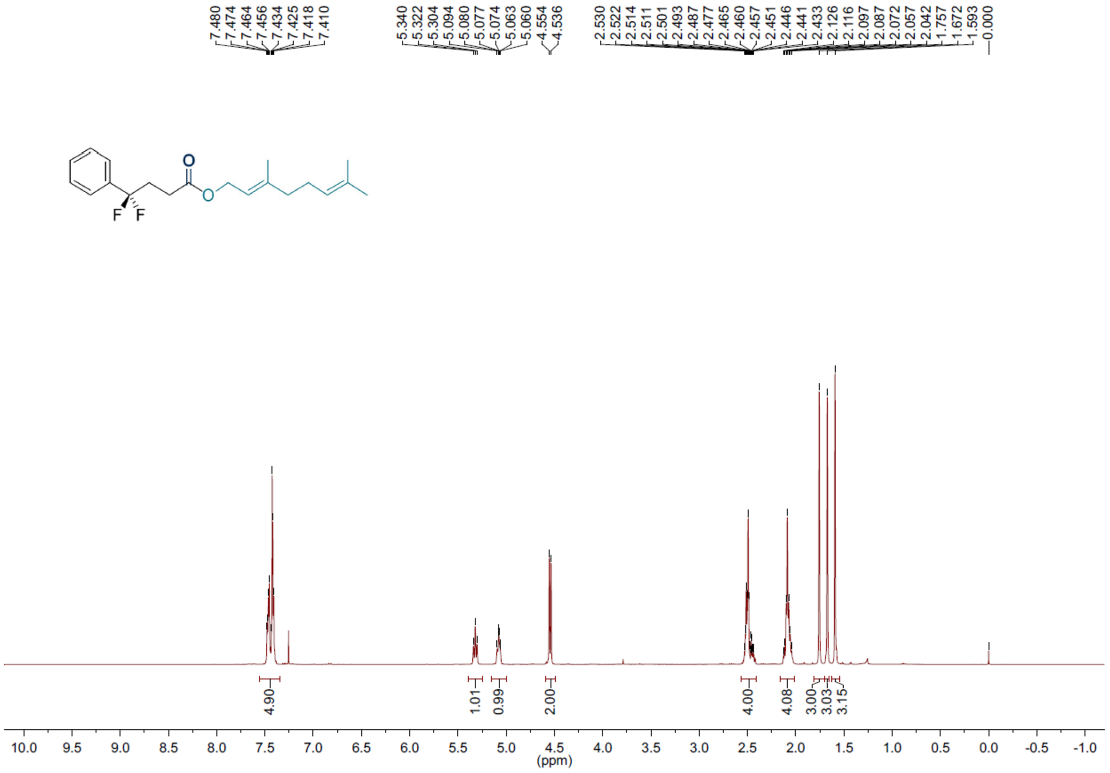

^{1}H NMR spectrum of **7a** in $CDCl_3$ (400 MHz)

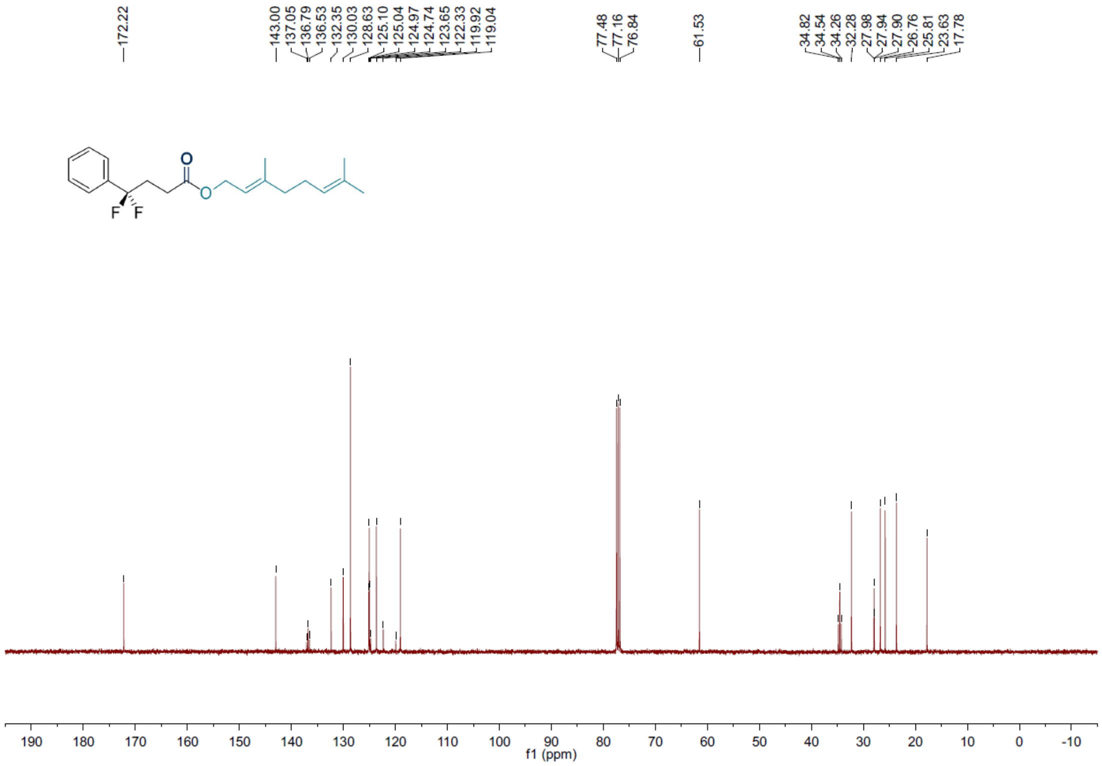

^{13}C NMR spectrum of **7a** in $CDCl_3$ (101 MHz)

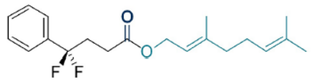

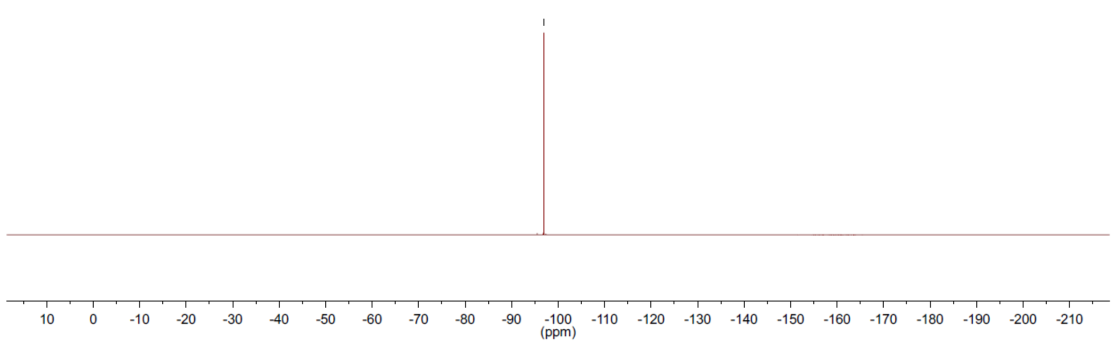

^{19}F NMR spectrum of **7a** in $CDCl_3$ (376 MHz)

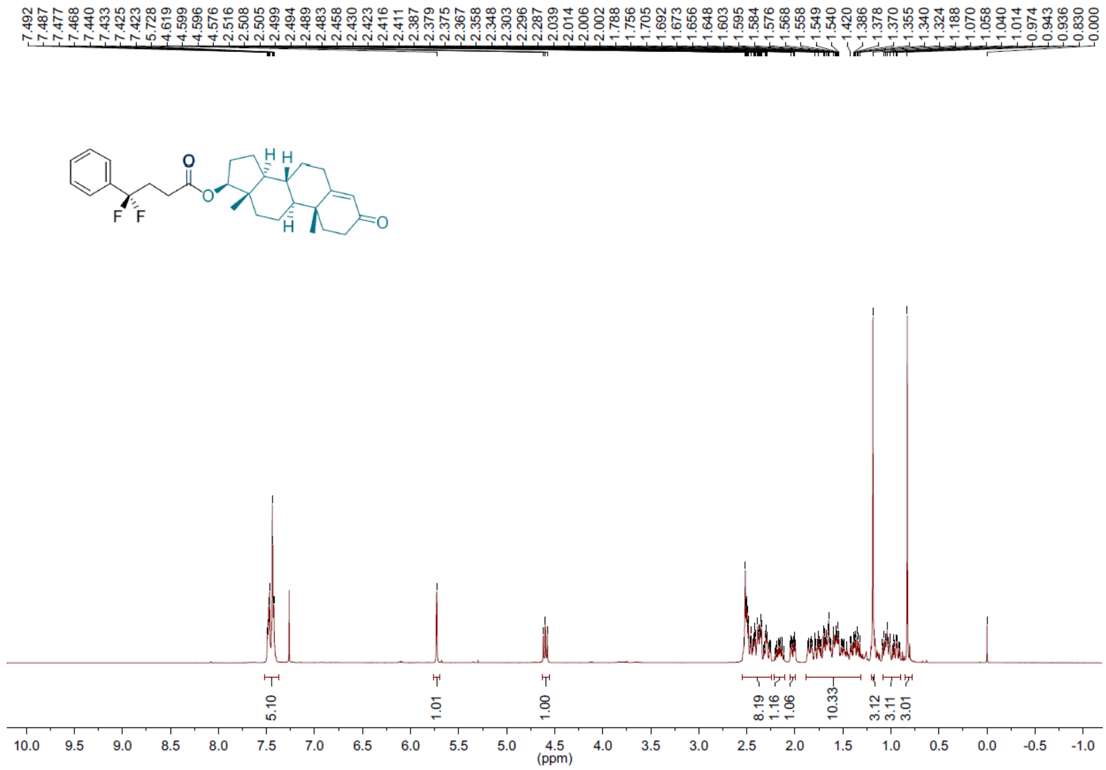

^{1}H NMR spectrum of 7**b** in $CDCl_3$ (400 MHz)

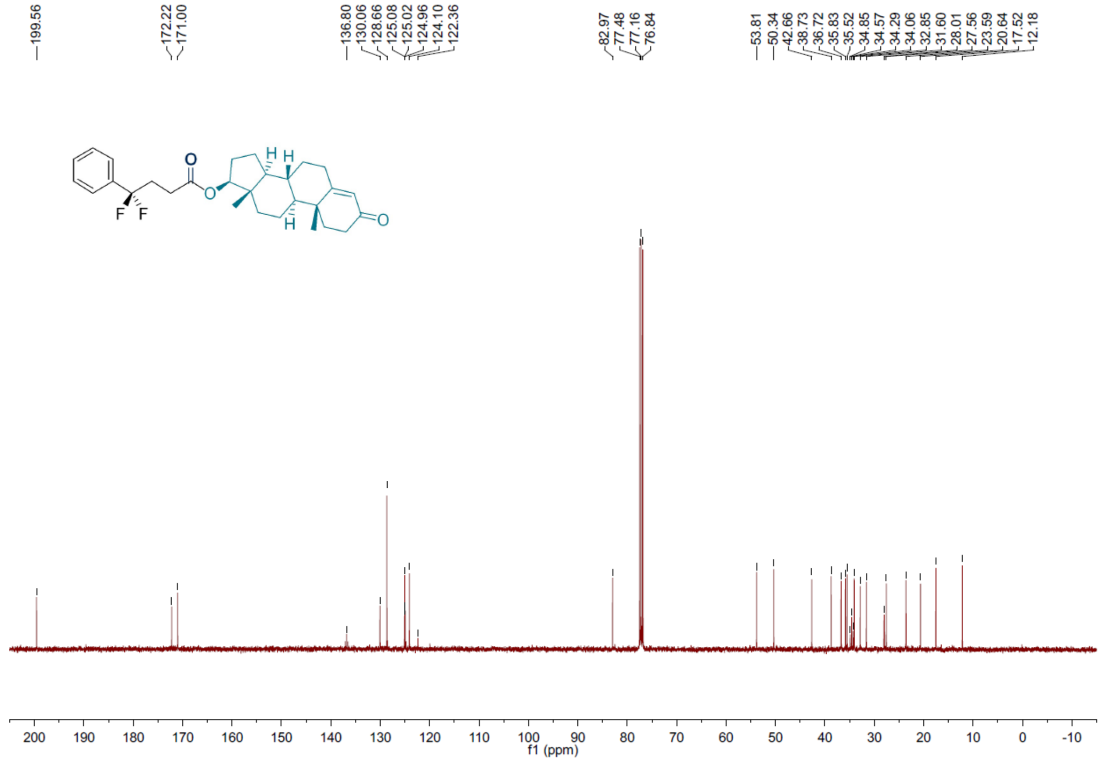

^{13}C NMR spectrum of **7b** in $CDCl_3$ (101 MHz)

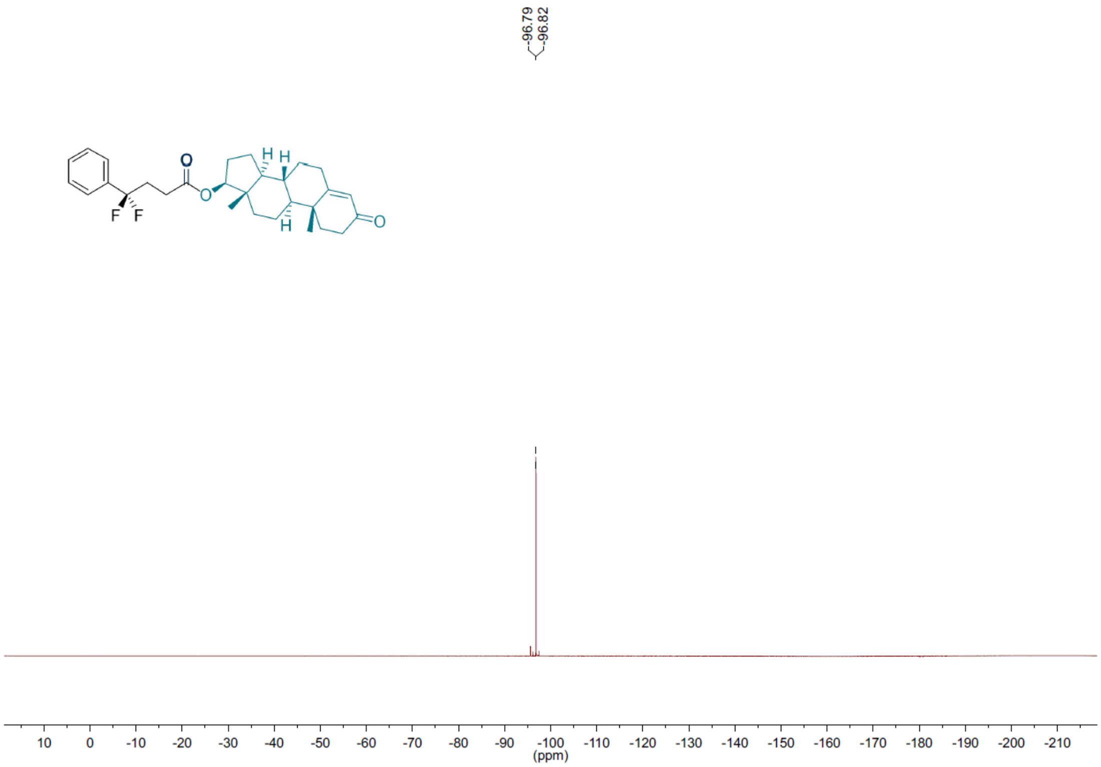

^{19}F NMR spectrum of **7b** in $CDCl_3$ (376 MHz)

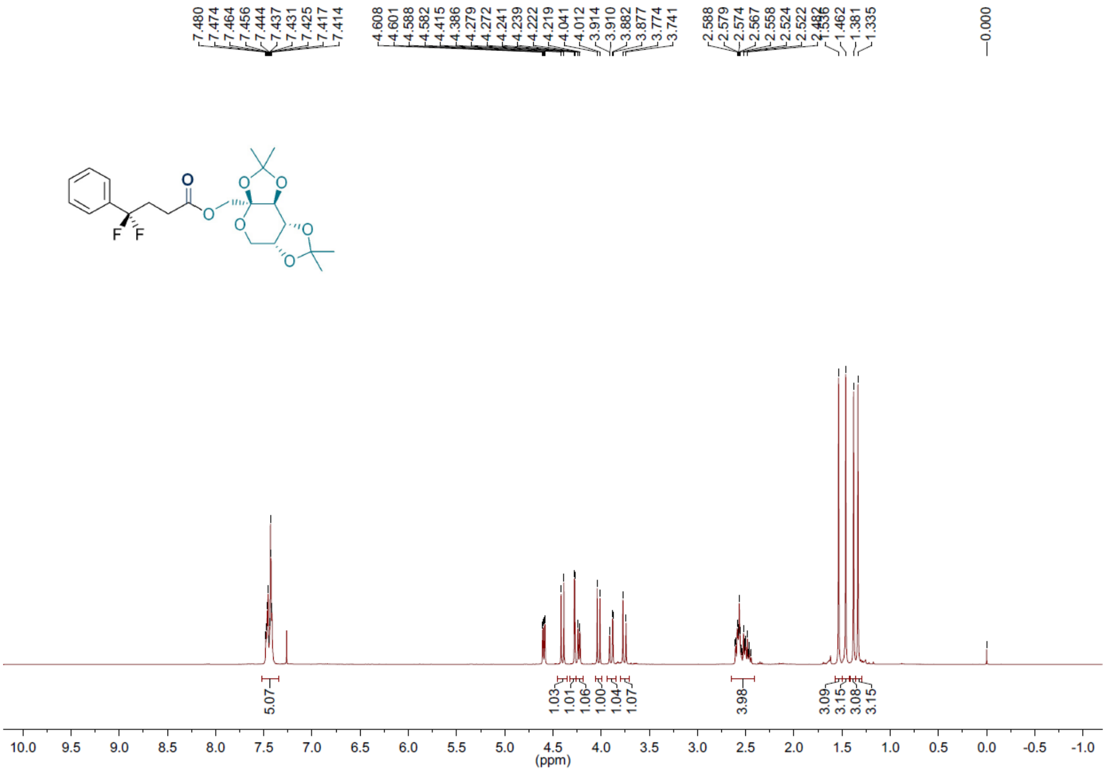

^{1}H NMR spectrum of **7c** in $CDCl_3$ (400 MHz)

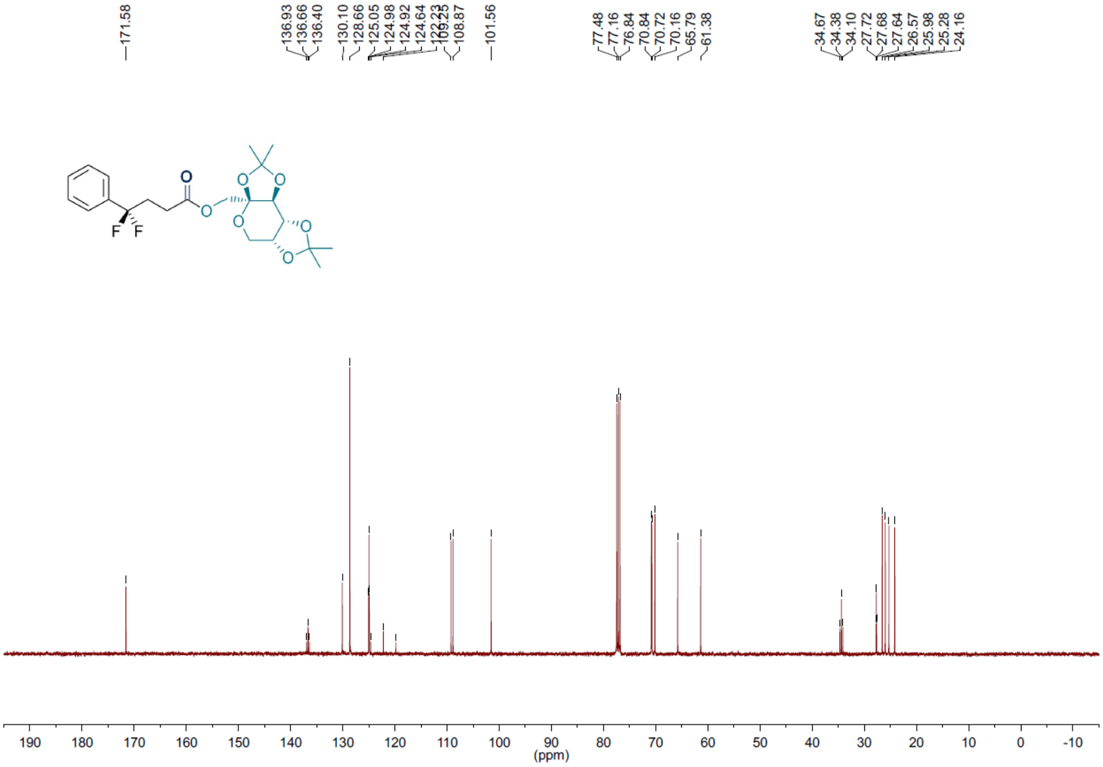

^{13}C NMR spectrum of **7c** in $CDCl_3$ (101 MHz)

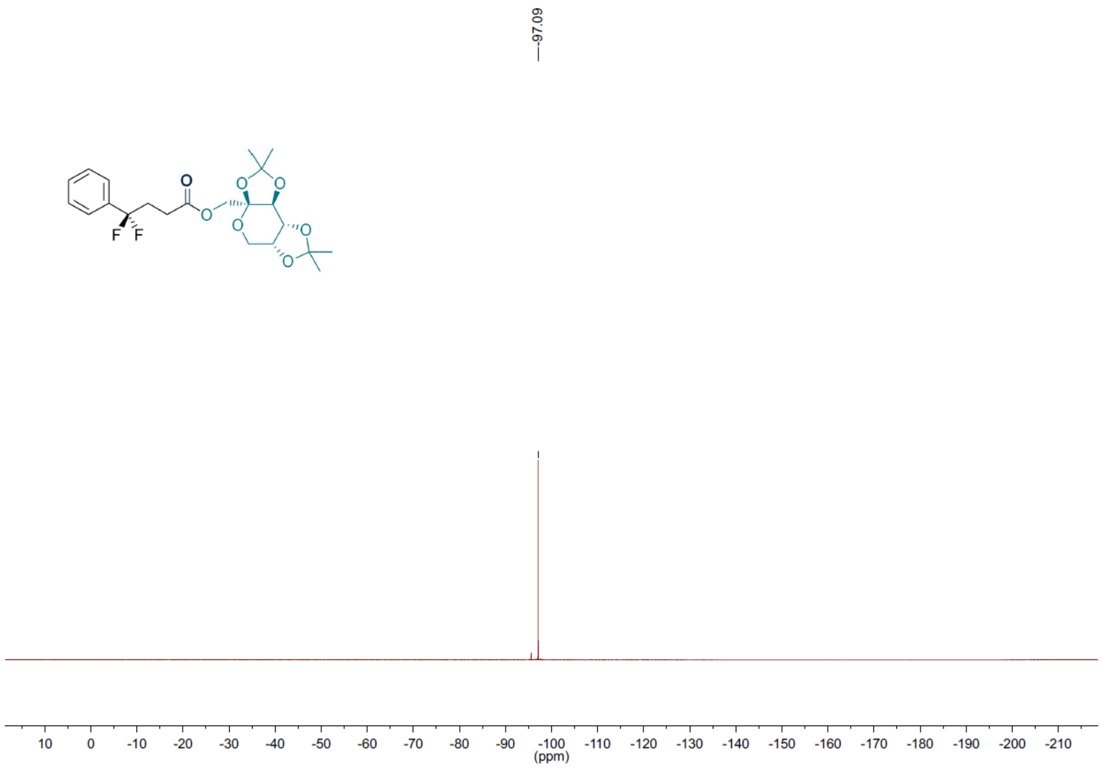

^{19}F NMR spectrum of **7c** in $CDCl_3$ (376 MHz)

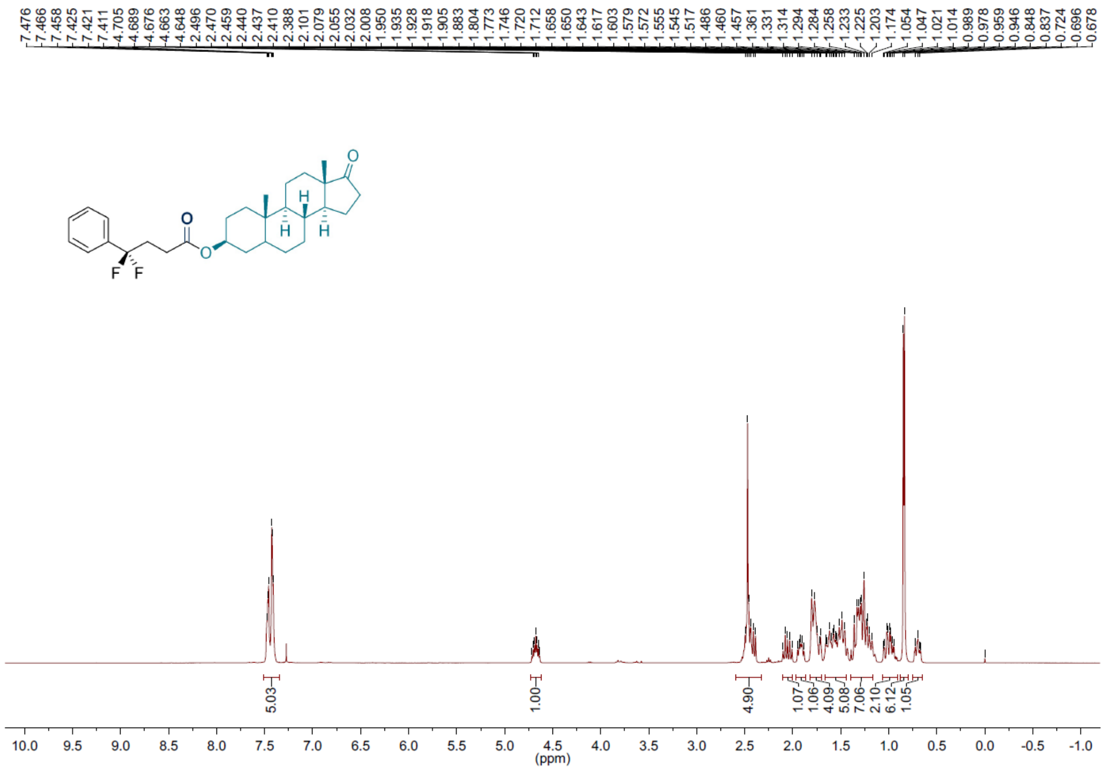

^{1}H NMR spectrum of **7d** in $CDCl_3$ (400 MHz)

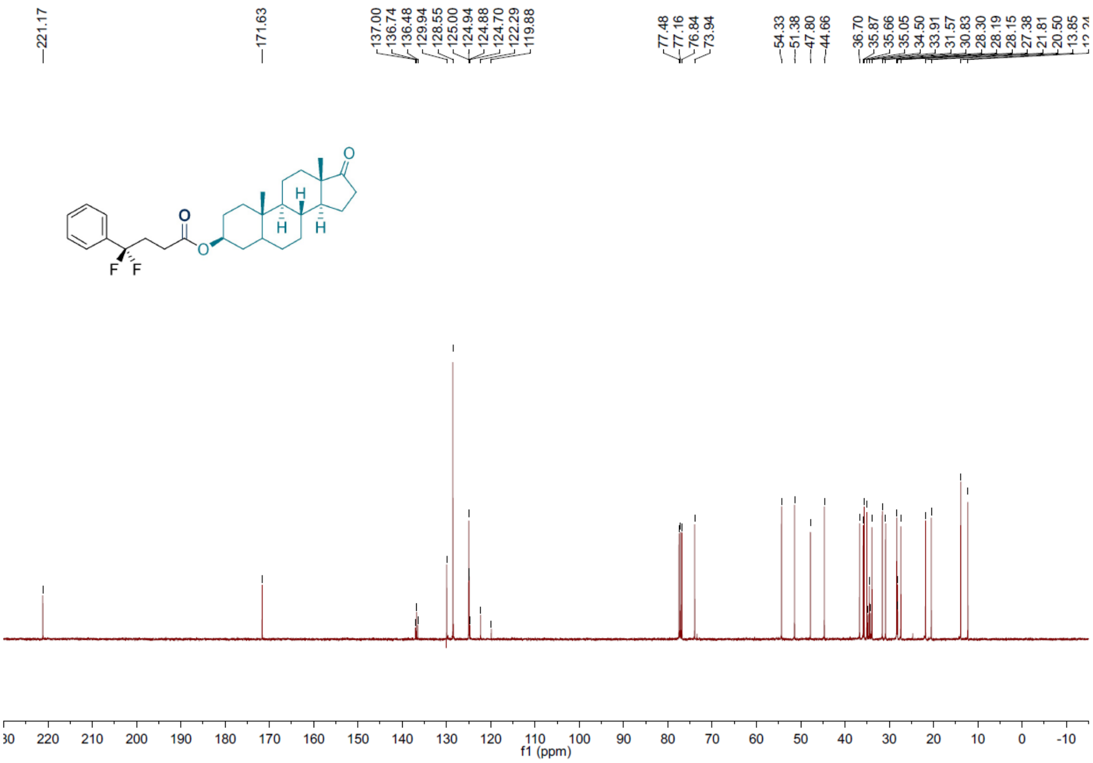

^{13}C NMR spectrum of **7d** in $CDCl_3$ (101 MHz)

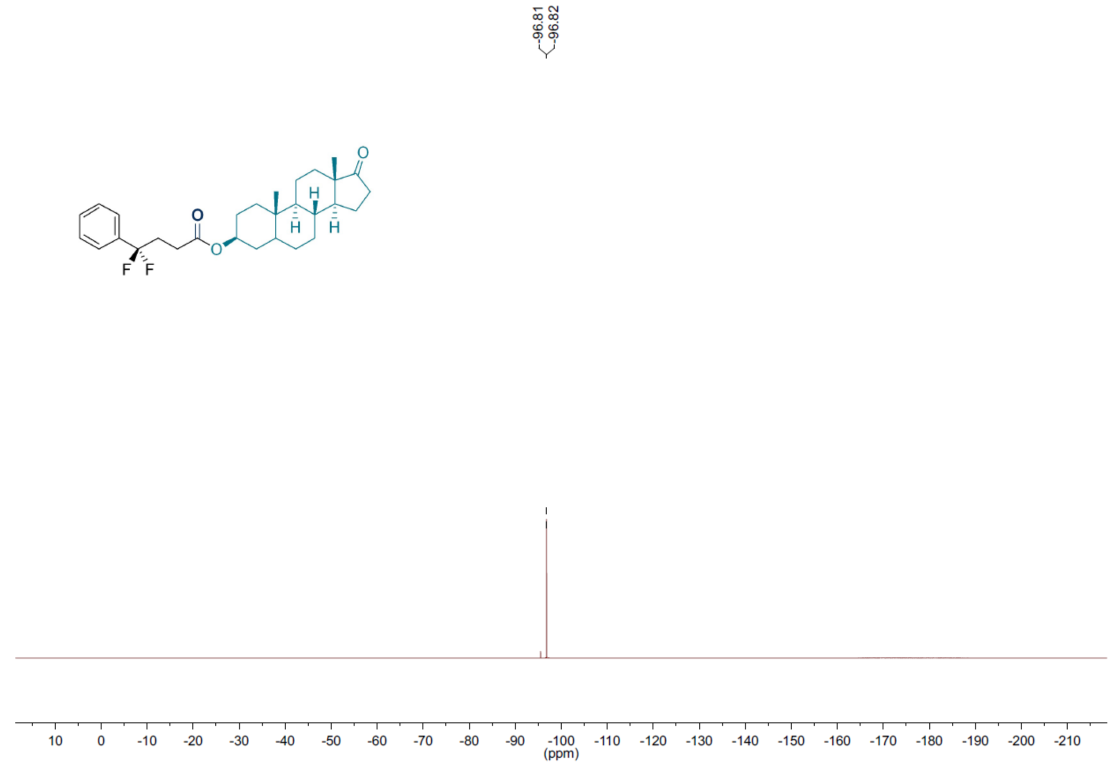

^{19}F NMR spectrum of **7d** in $CDCl_3$ (376 MHz)

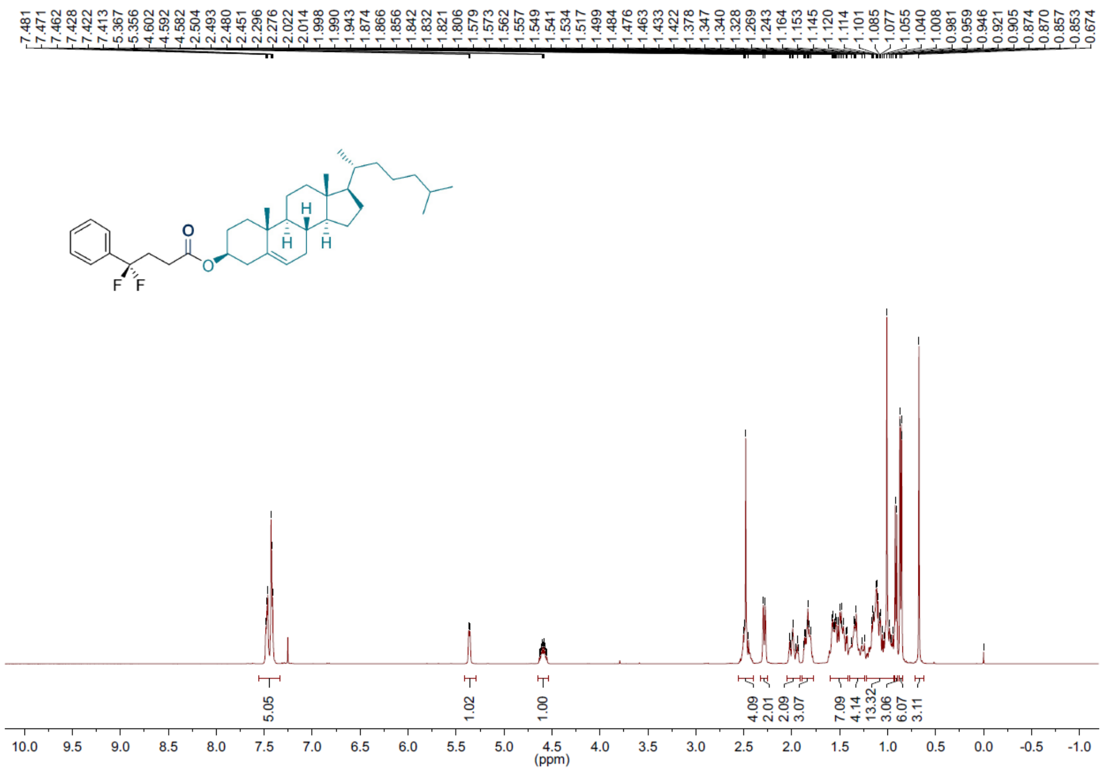

^{1}H NMR spectrum of **7e** in $CDCl_3$ (400 MHz)

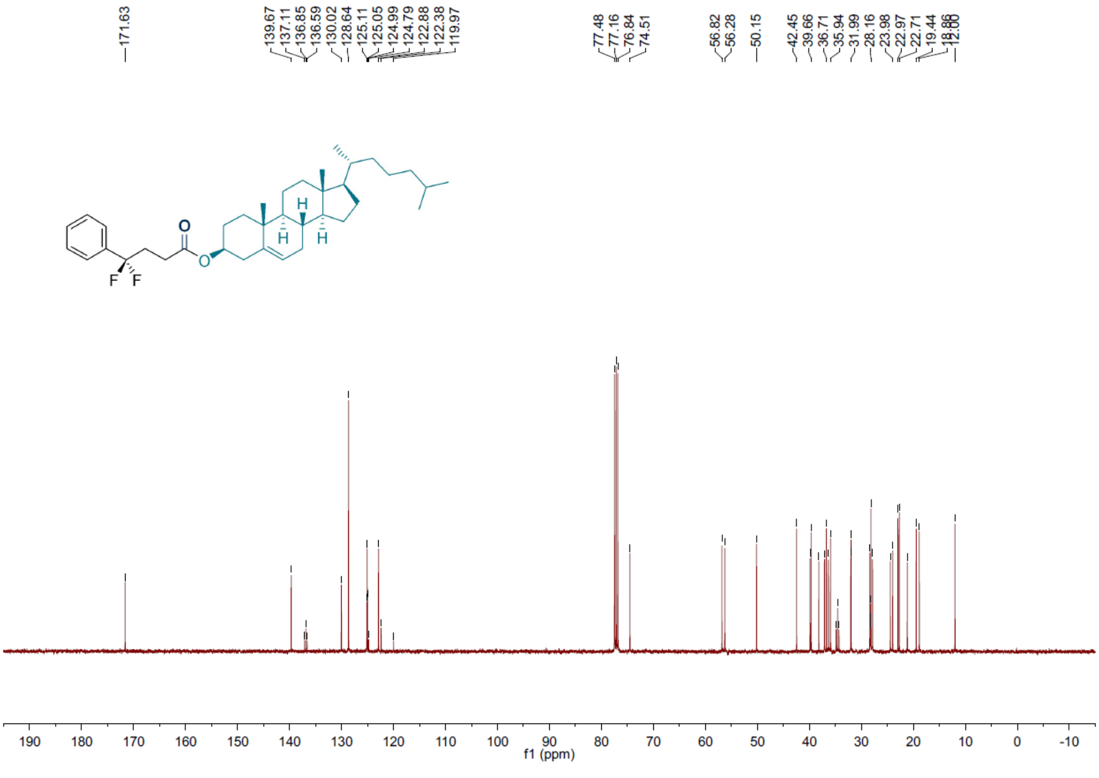

^{13}C NMR spectrum of **7e** in $CDCl_3$ (101 MHz)

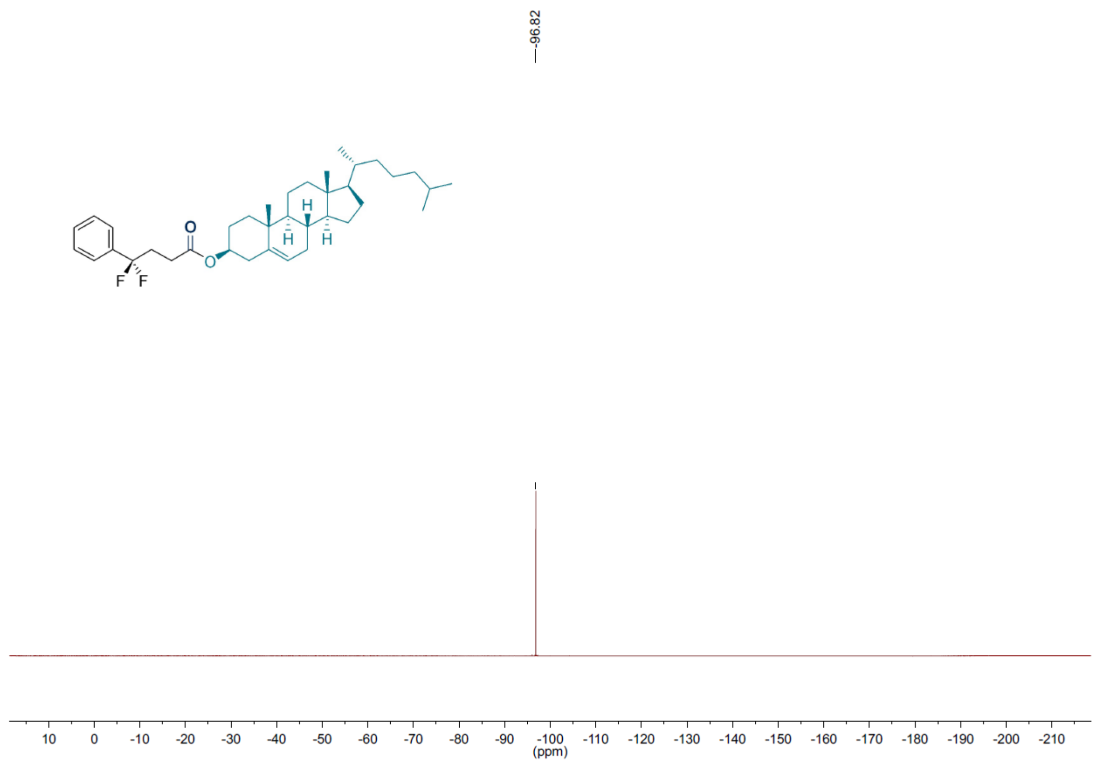

^{19}F NMR spectrum of **7e** in $CDCl_3$ (376 MHz)

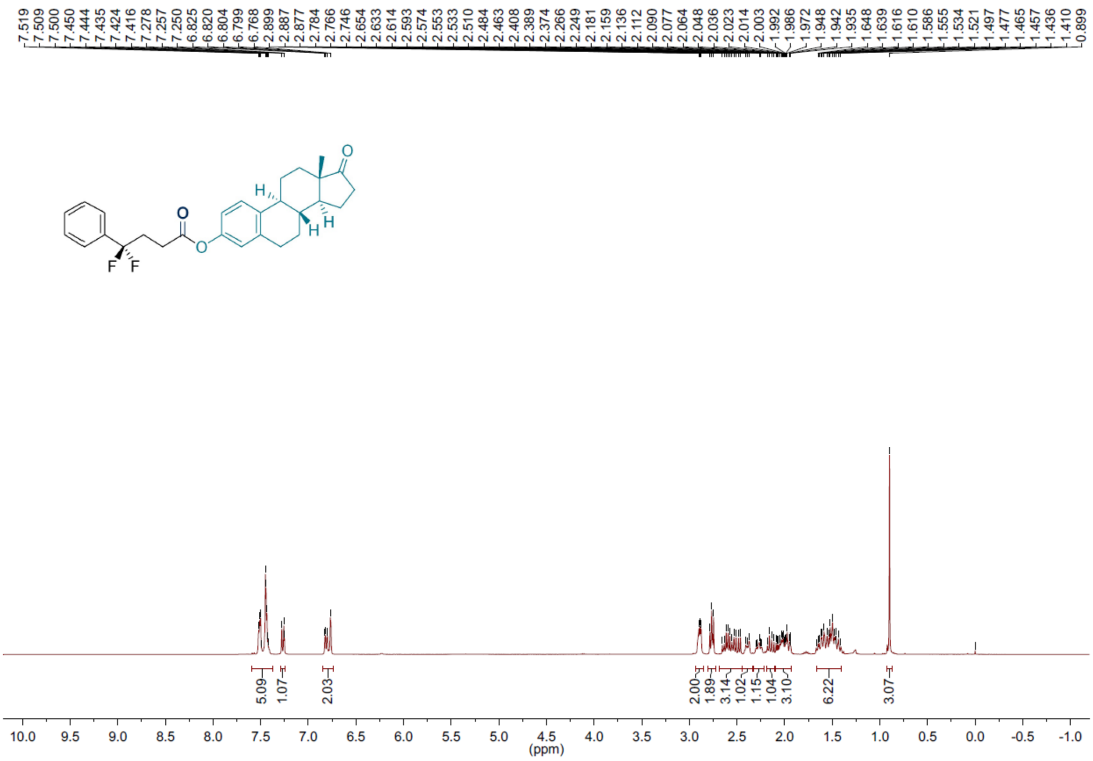

^{1}H NMR spectrum of **7f** in $CDCl_3$ (400 MHz)

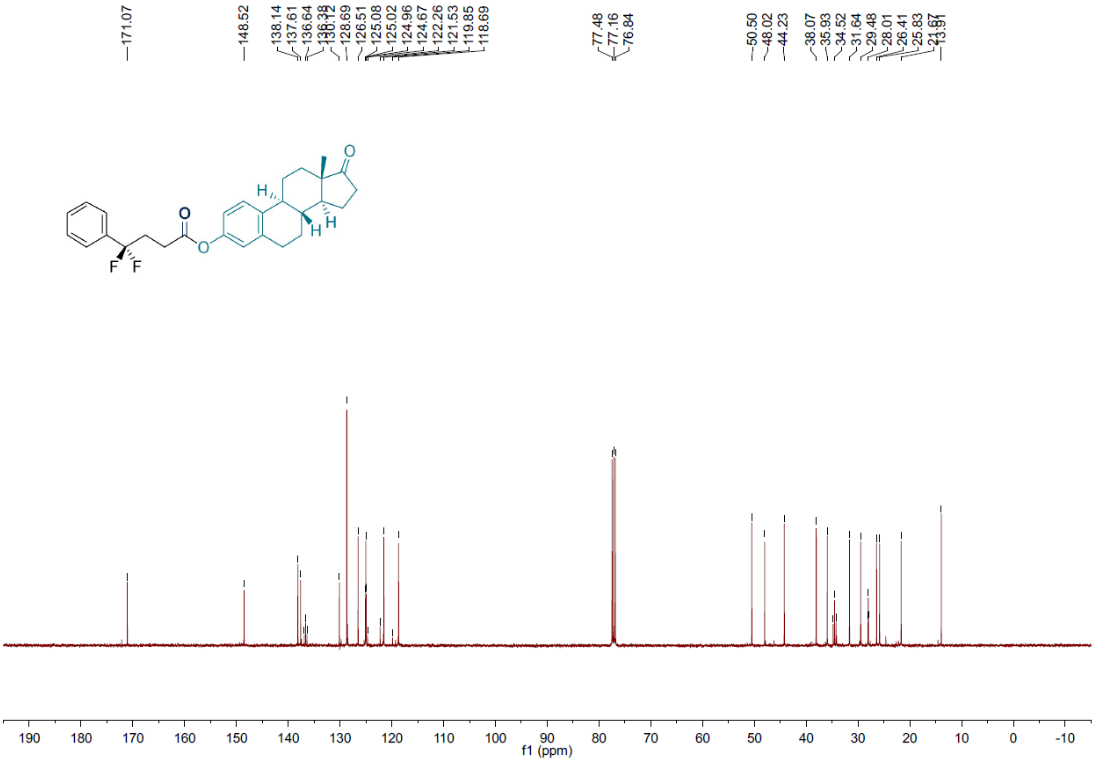

^{13}C NMR spectrum of **7f** in $CDCl_3$ (101 MHz)

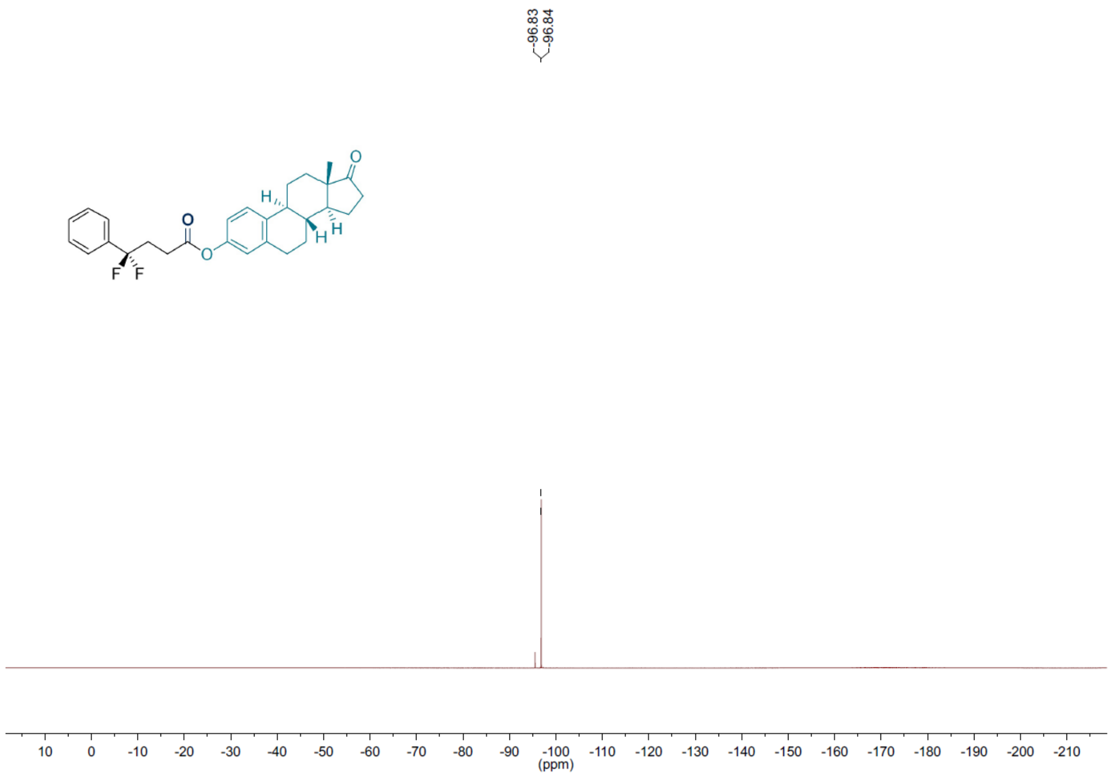

^{19}F NMR spectrum of **7f** in $CDCl_3$ (376 MHz)

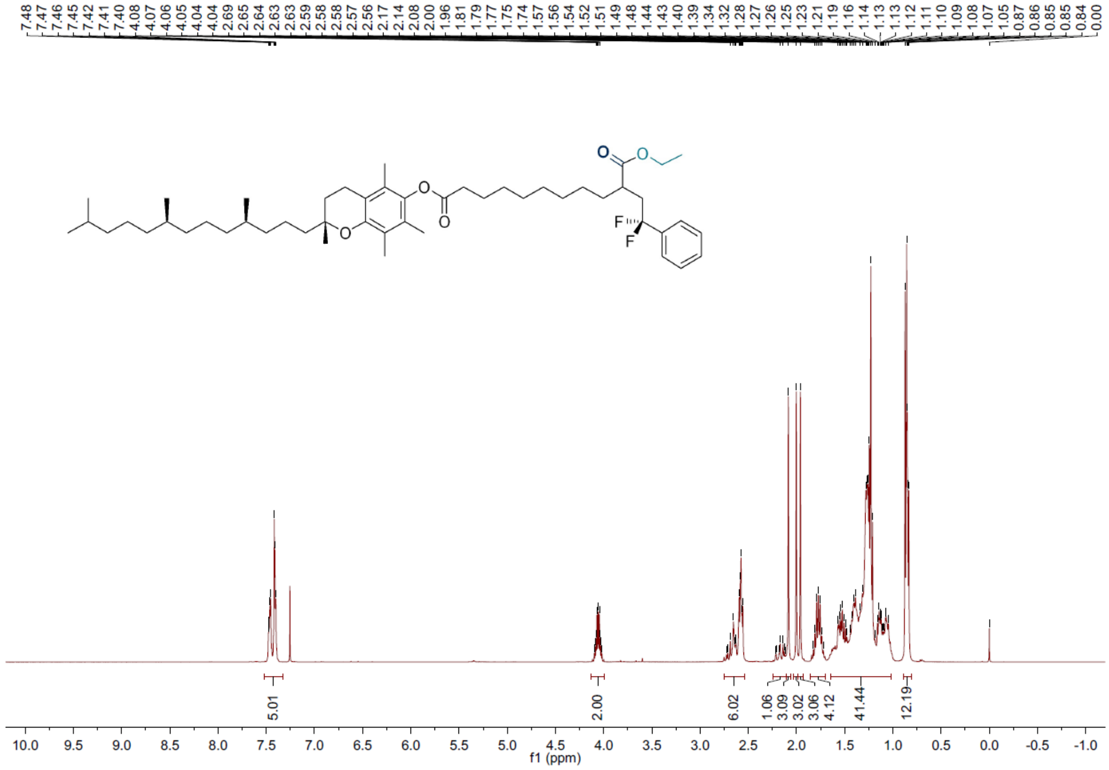

^{1}H NMR spectrum of **7g** in $CDCl_3$ (400 MHz)

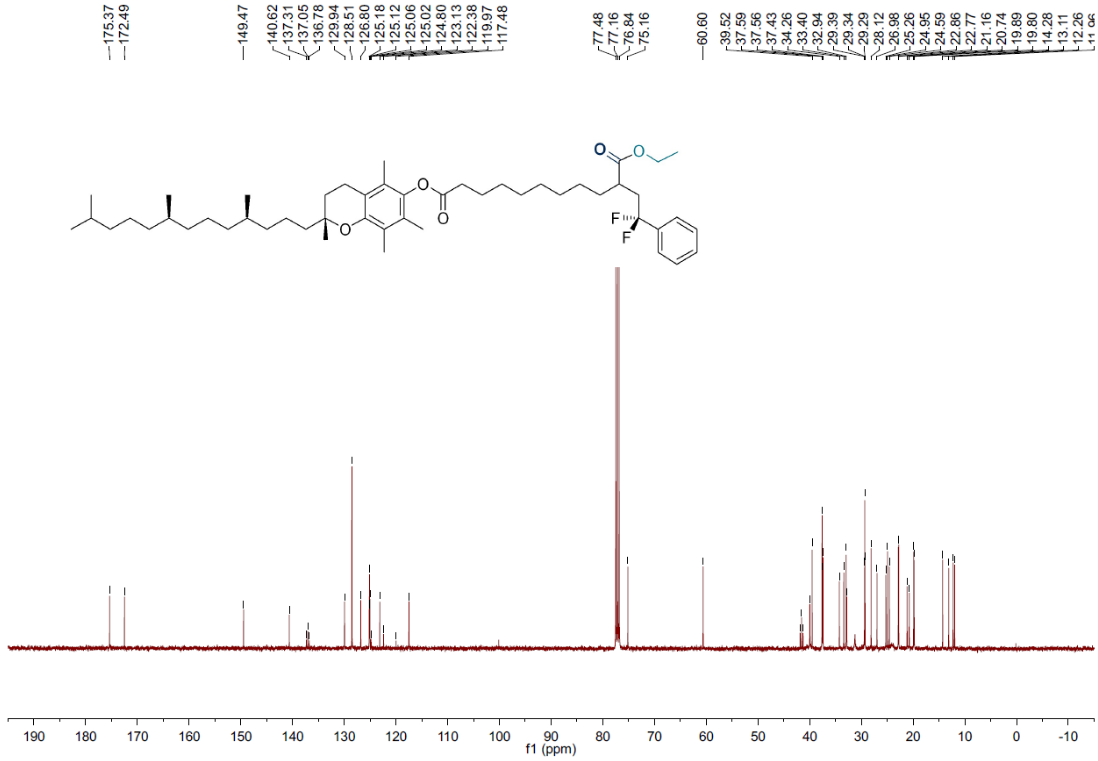

^{13}C NMR spectrum of **7g** in $CDCl_3$ (101 MHz)

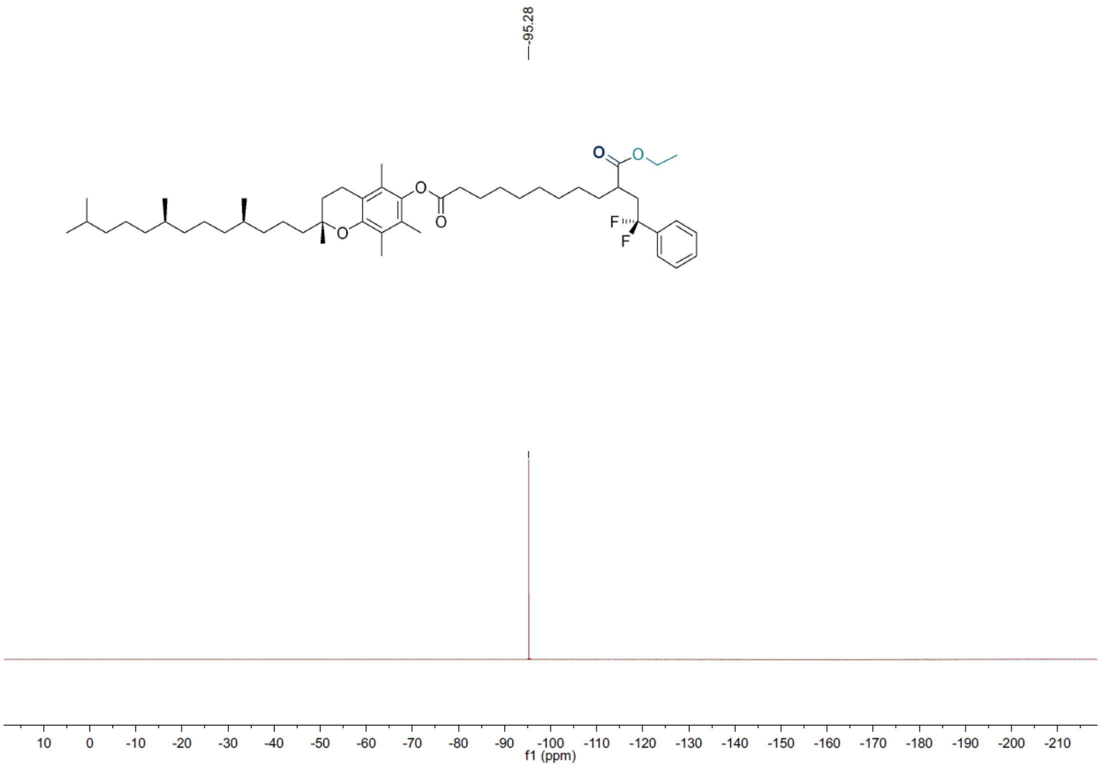

^{19}F NMR spectrum of **7g** in $CDCl_3$ (376 MHz)

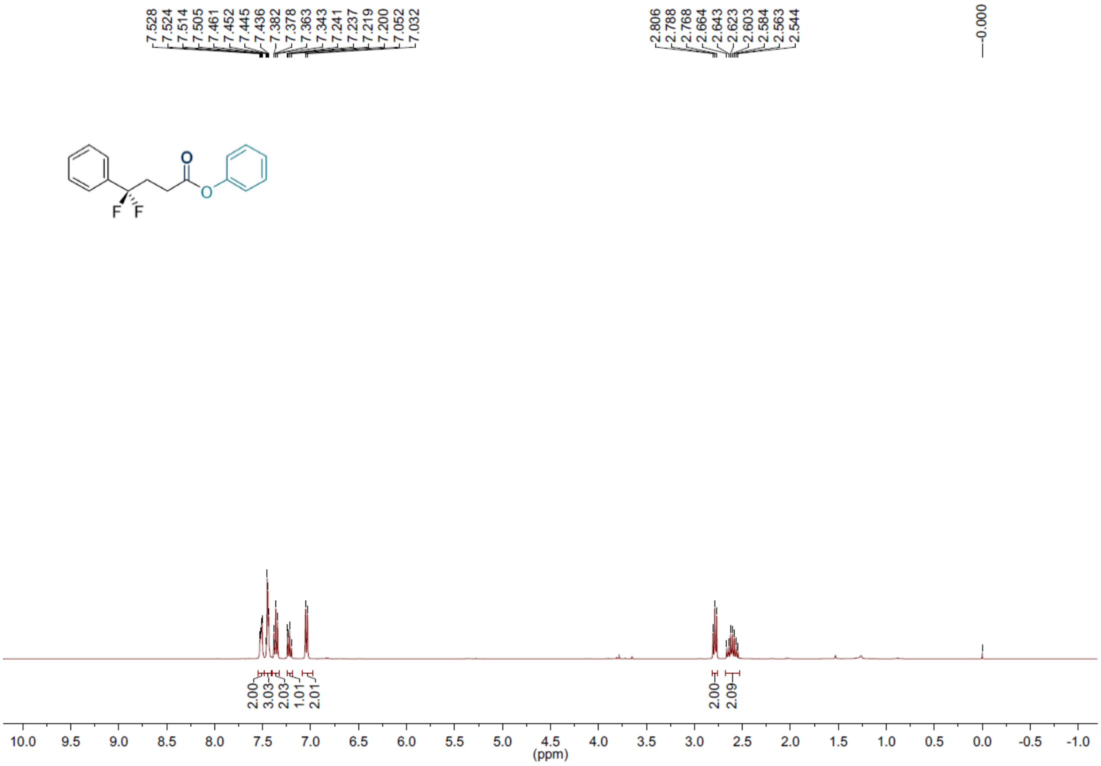

^{1}H NMR spectrum of **8b** in $CDCl_3$ (400 MHz)

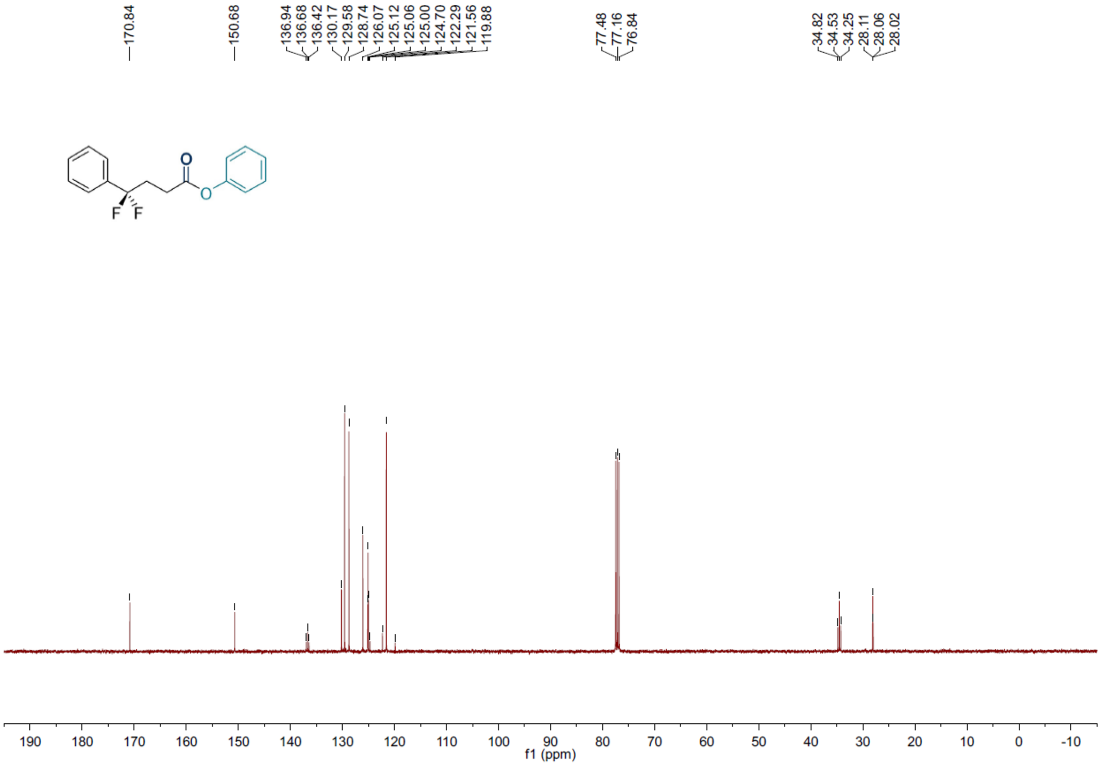

^{13}C NMR spectrum of **8b** in $CDCl_3$ (101 MHz)

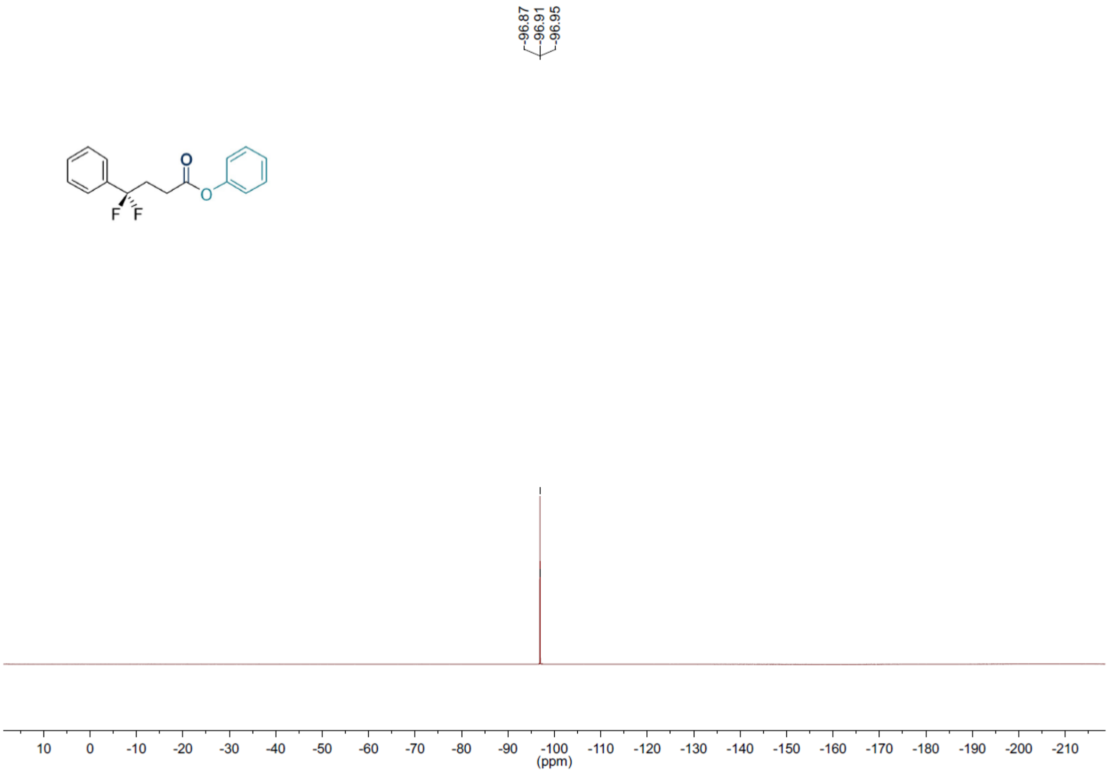

^{19}F NMR spectrum of **8b** in $CDCl_3$ (376 MHz)

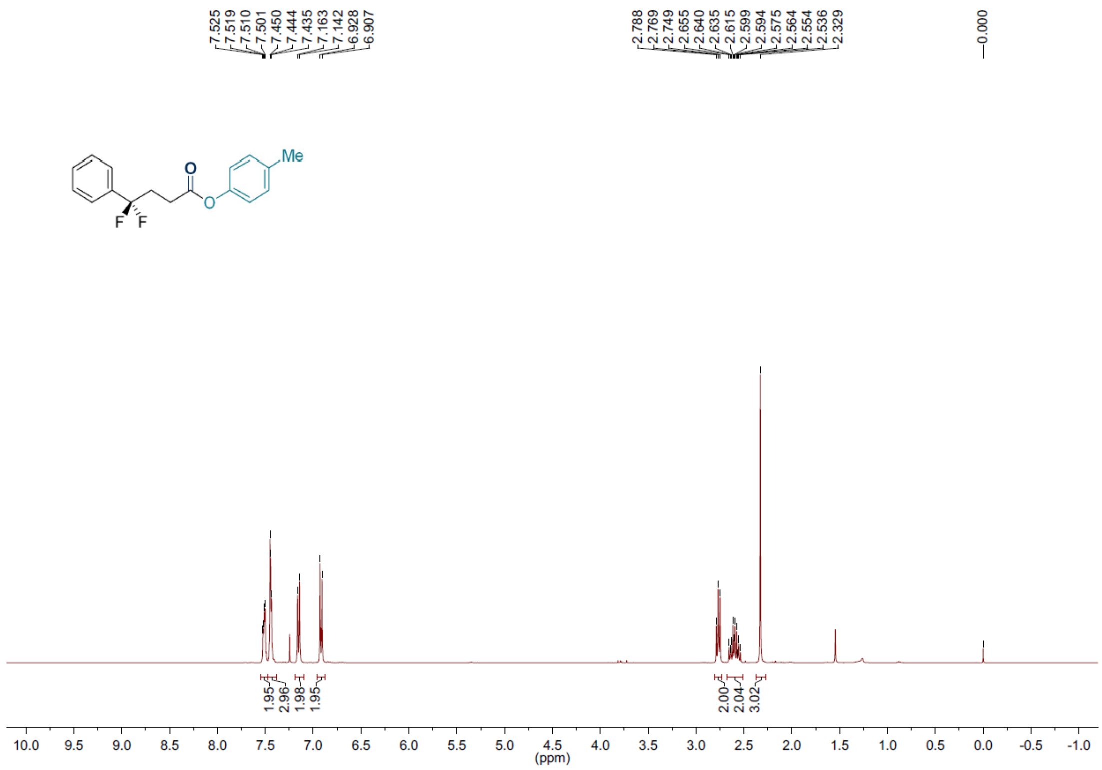

^{1}H NMR spectrum of **8c** in $CDCl_3$ (400 MHz)

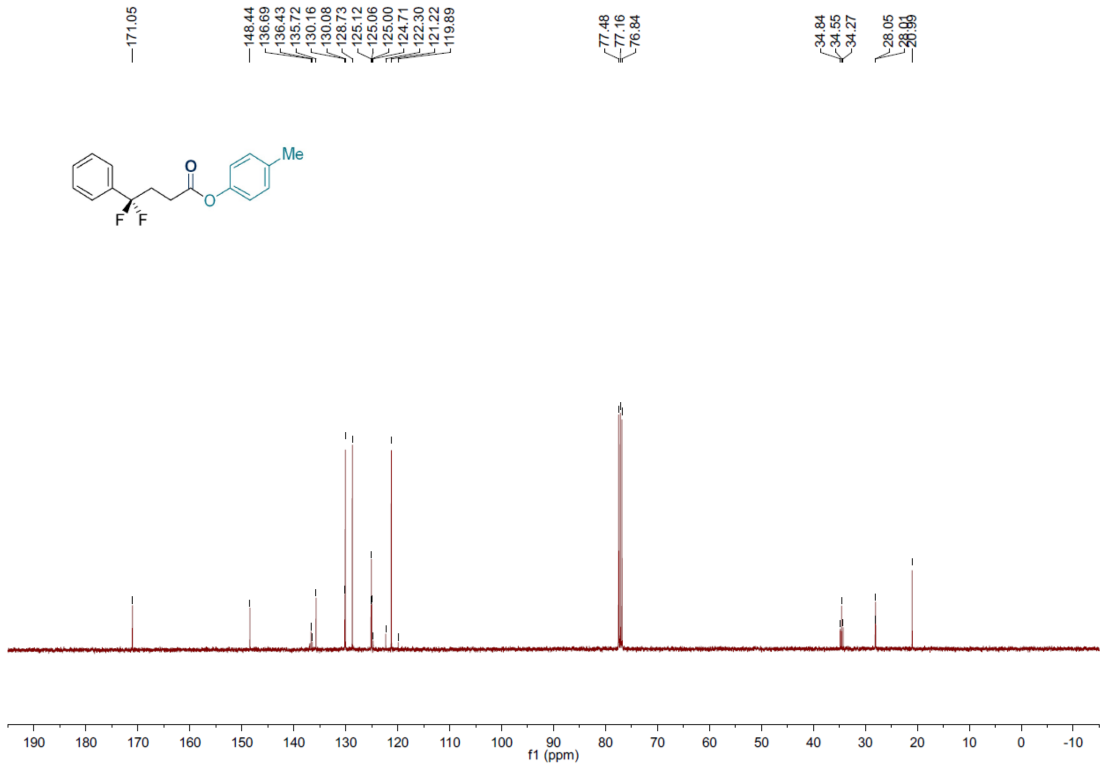

^{13}C NMR spectrum of **8c** in $CDCl_3$ (101 MHz)

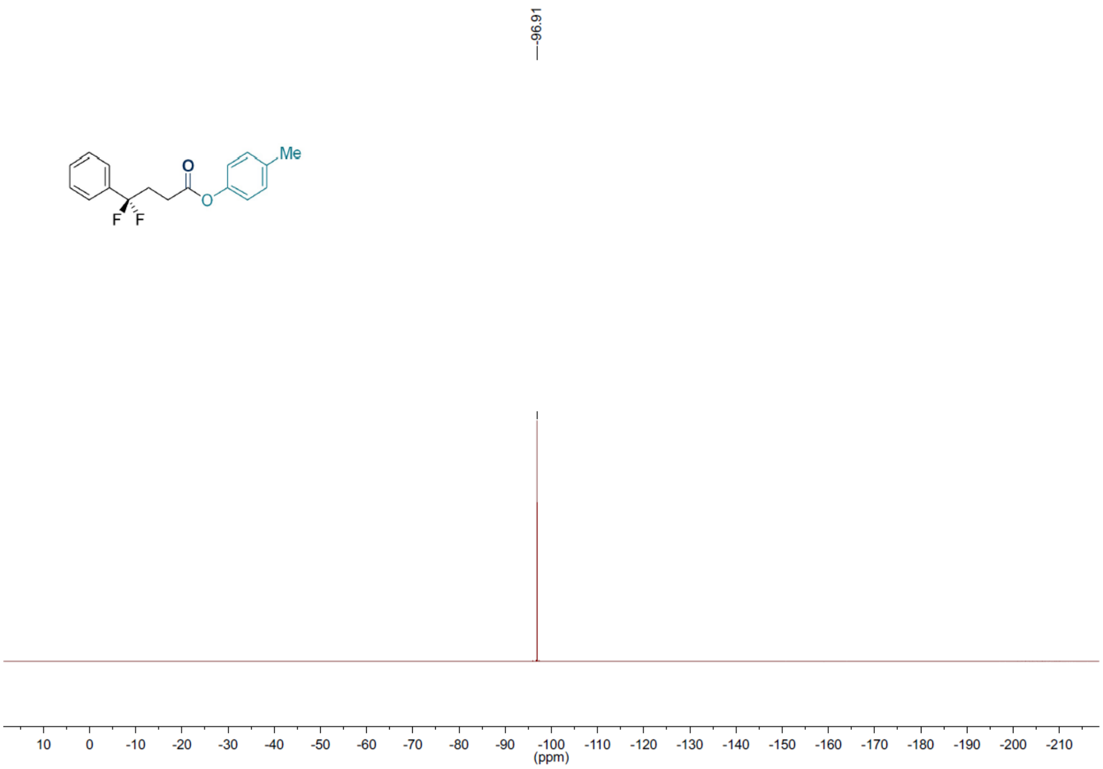

^{19}F NMR spectrum of **8c** in $CDCl_3$ (376 MHz)

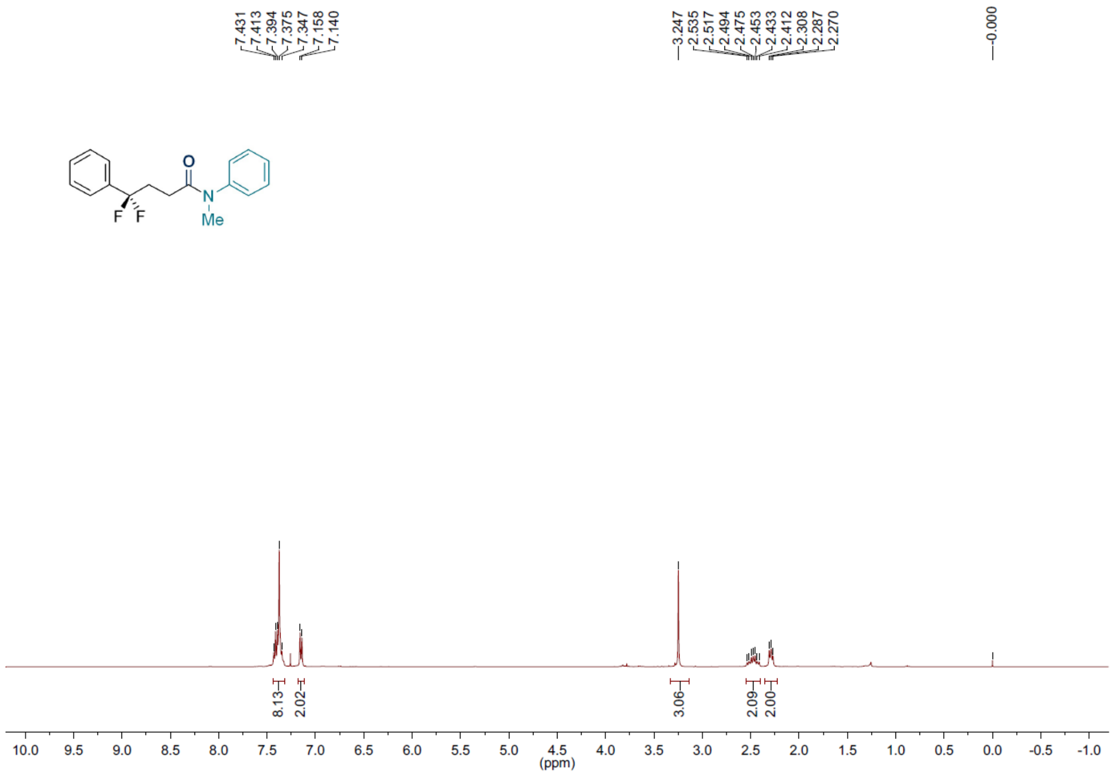

^{1}H NMR spectrum of **9c** in $CDCl_3$ (400 MHz)

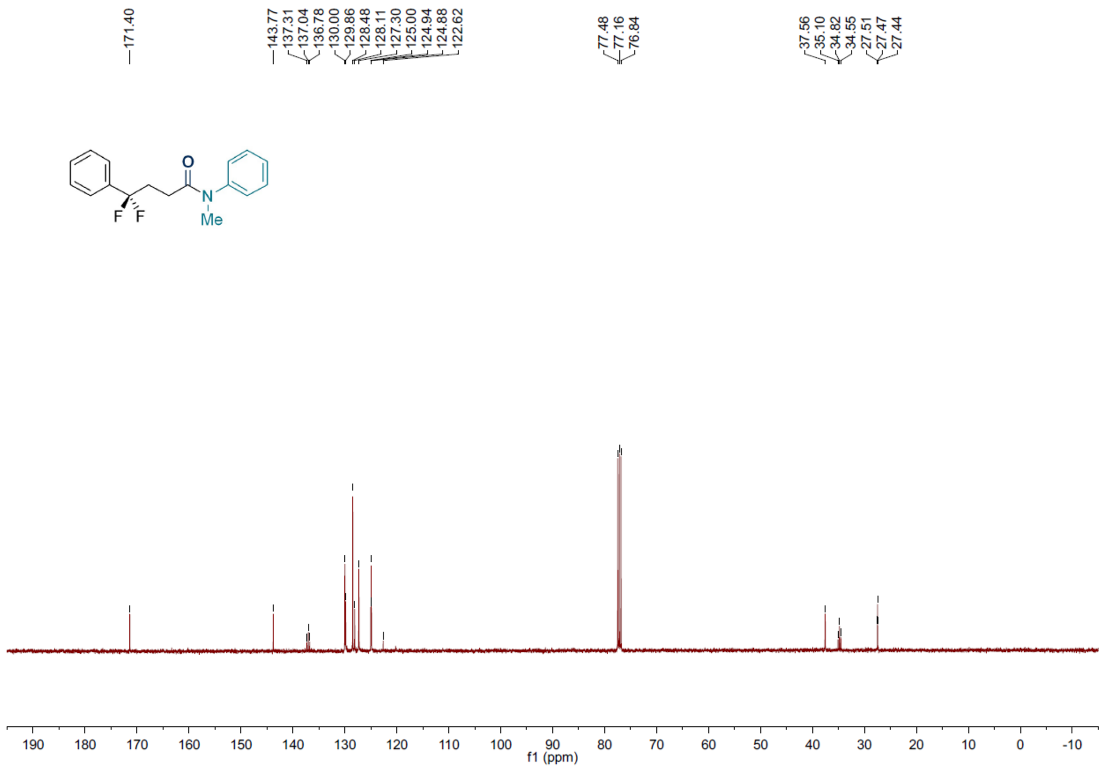

^{13}C NMR spectrum of **9c** in $CDCl_3$ (101 MHz)

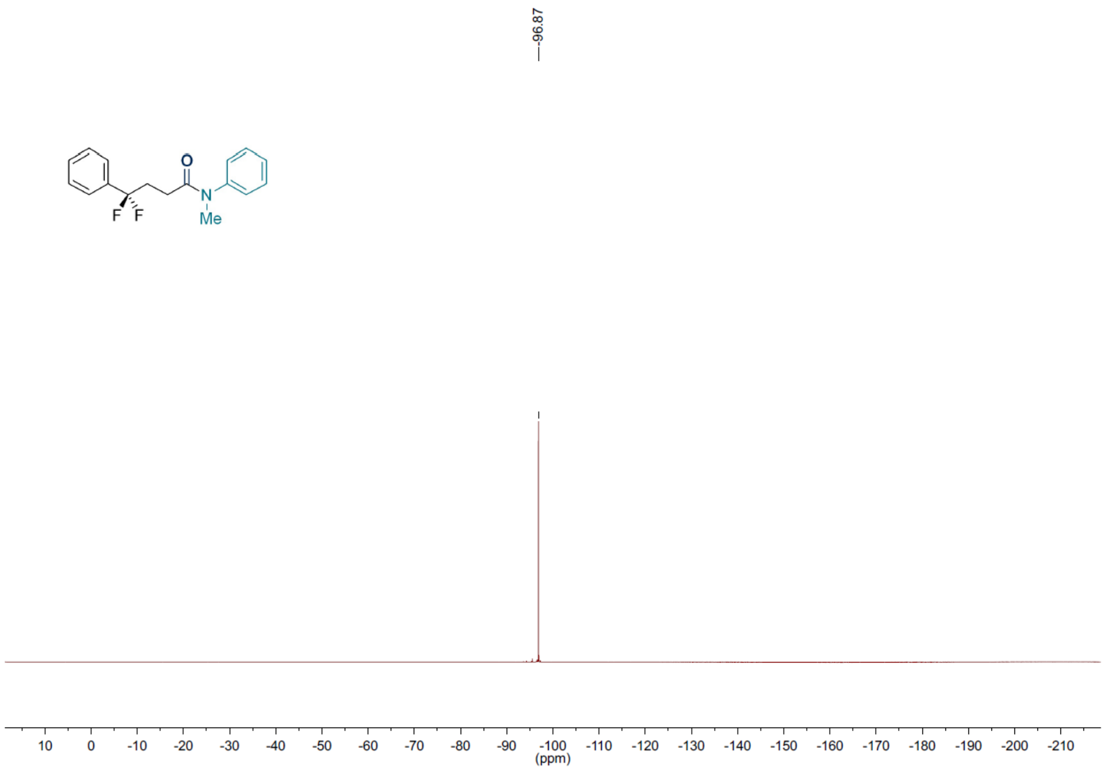

^{19}F NMR spectrum of **9c** in $CDCl_3$ (376 MHz)

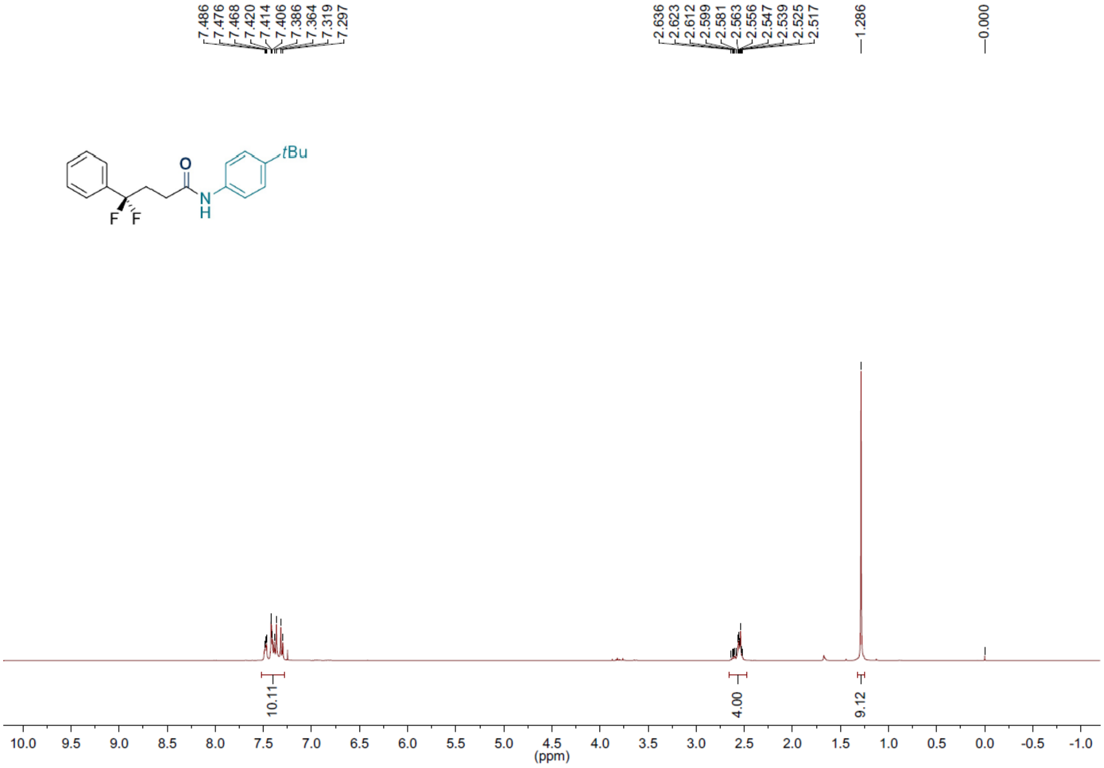

^{1}H NMR spectrum of **9d** in $CDCl_3$ (400 MHz)

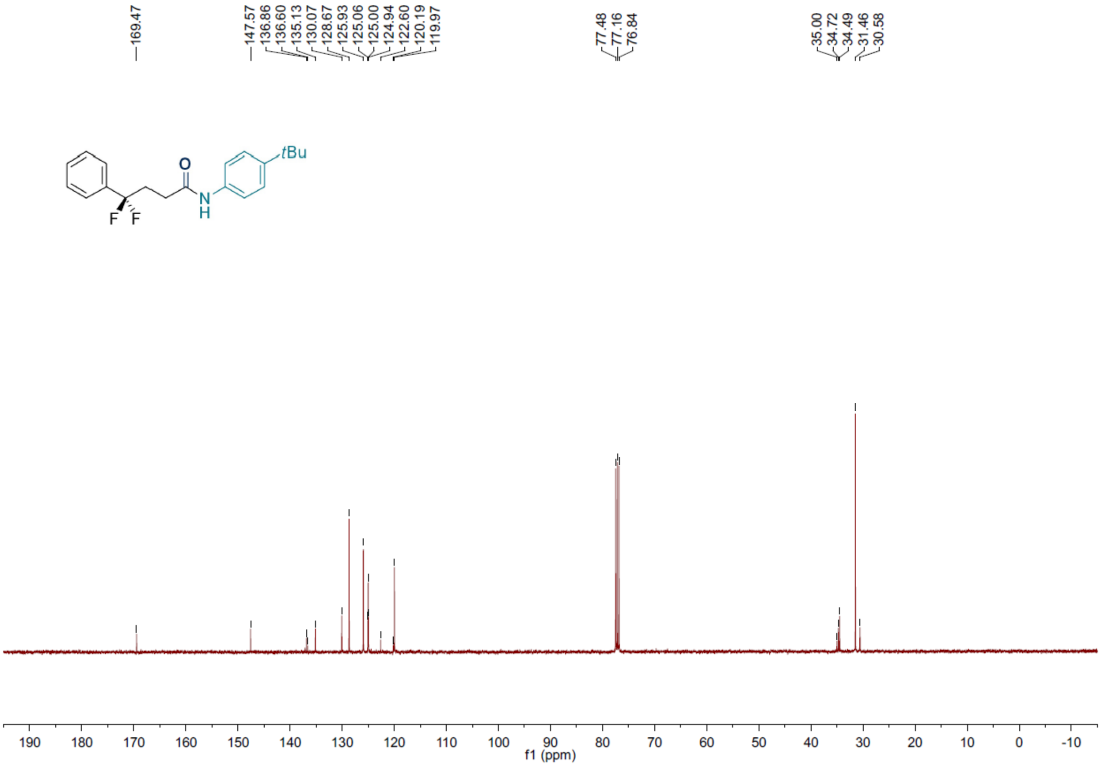

^{13}C NMR spectrum of **9d** in $CDCl_3$ (101 MHz)

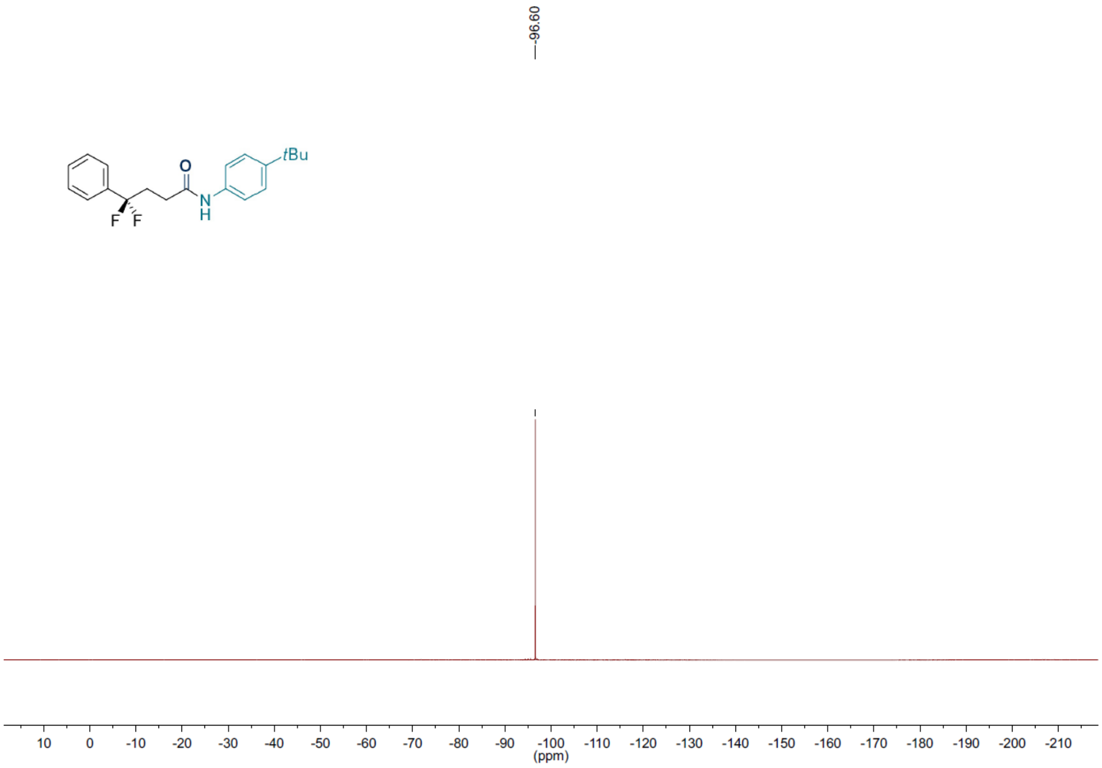

^{19}F NMR spectrum of **9d** in $CDCl_3$ (376 MHz)

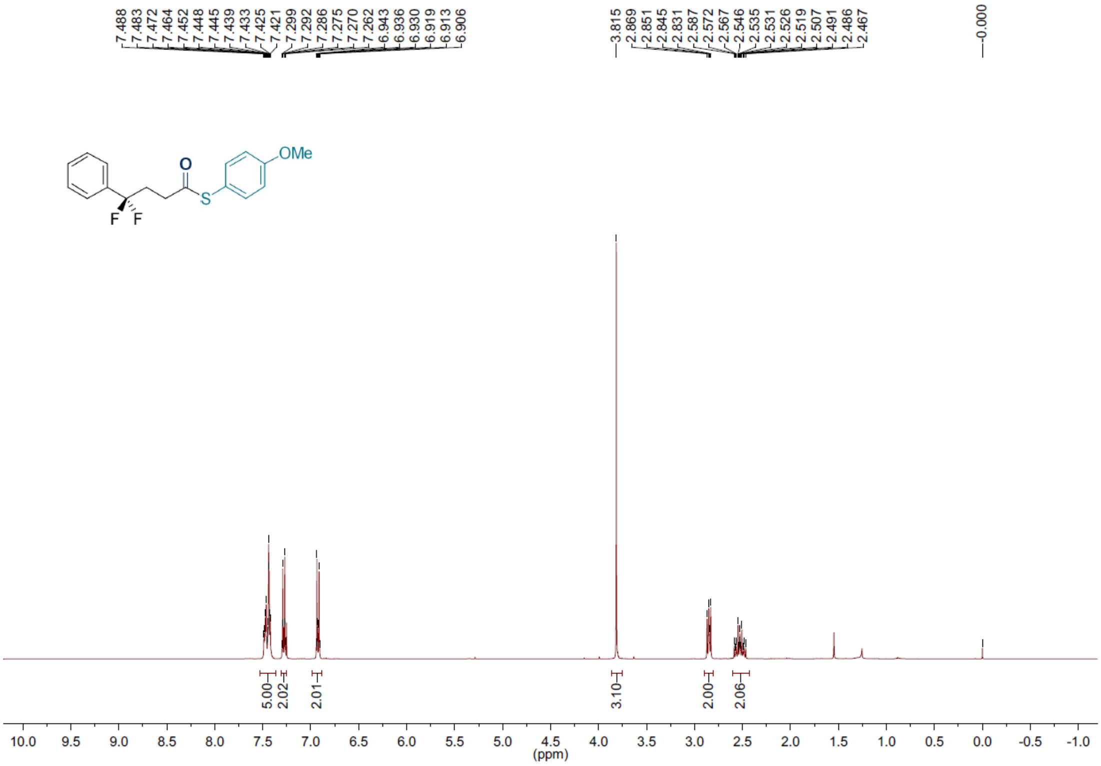

^{1}H NMR spectrum of **16** in $CDCl_3$ (400 MHz)

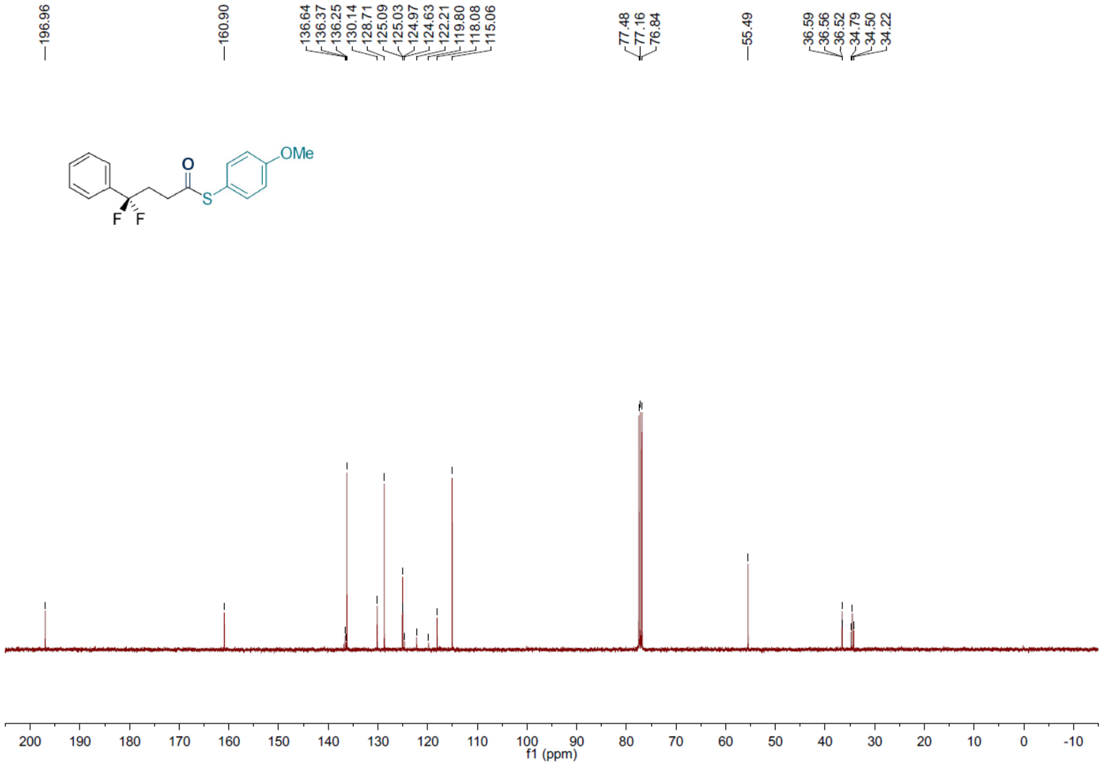

^{13}C NMR spectrum of **16** in $CDCl_3$ (101 MHz)

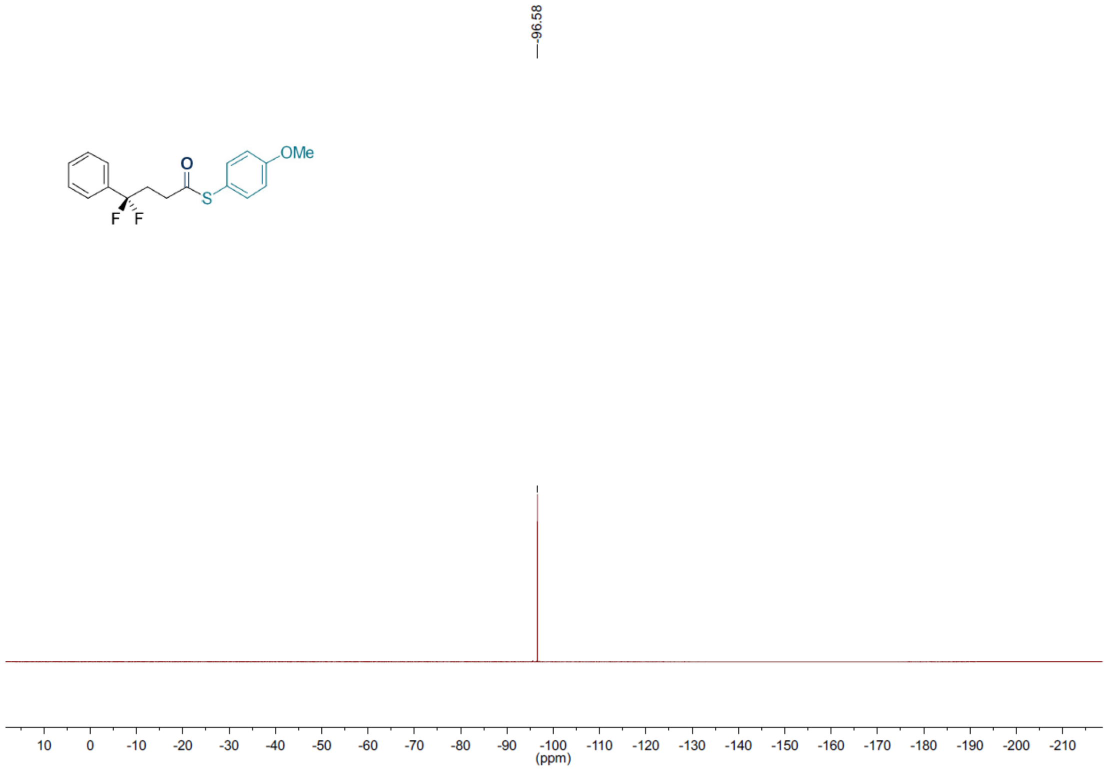

^{19}F NMR spectrum of **16** in $CDCl_3$ (376 MHz)

8. Supplementary References

1. Wei, D.; Liu, T.; He, Y.; Wei, B.; Pan, J.; Zhang, J.; Jiao, N.; Han, B. Radical 1,4/5-Amino Shift Enables Access to Fluoroalkyl-Containing Primary $\beta(\gamma)$-Aminoketones under Metal-Free Conditions. *Angew. Chem., Int. Ed.* **2021**, *60*, 26308-26313.
2. Liu, H.; Fu, Z.; Li, X.; Yu, S. Halogen-Bond-Assisted Radical Remote Difunctionalization of Bicyclo[1.1.1]Butane Skeletons. *Green Chemistry* **2025**, *27*, 256-263.
3. Cheng, Y.; Yu, S. Hydrotrifluoromethylation of Unactivated Alkenes and Alkynes Enabled by an Electron-Donor-Acceptor Complex of Togni's Reagent with a Tertiary Amine. *Org. Lett.* **2016**, *18*, 2962-2965.
4. Qin, Q.; Zhang, L.; Wei, J.; Qiu, X.; Hao, S.; An, X.-D.; Jiao, N. Direct Oxygen Insertion into C-C Bond of Styrenes with Air. *Nat. Commun.* **2024**, *15*, 9015.
